# Bioinformatic Inference of Specific and General Transcription Factor Binding Sites in the Plant Pathogen *Phytophthora infestans*

**DOI:** 10.1371/journal.pone.0051295

**Published:** 2012-12-12

**Authors:** Michael F Seidl, Rui-Peng Wang, Guido Van den Ackerveken, Francine Govers, Berend Snel

**Affiliations:** 1 Theoretical Biology and Bioinformatics, Department of Biology, Utrecht University, Utrecht, The Netherlands; 2 Centre for BioSystems Genomics, Wageningen, The Netherlands; 3 Centre for Integrative Bioinformatics VU (IBIVU), VU University Amsterdam, Amsterdam, The Netherlands; 4 Plant-Microbe Interactions, Department of Biology, Utrecht University, Utrecht, The Netherlands; 5 Laboratory of Phytopathology, Wageningen University, Wageningen, The Netherlands; Friedrich-Alexander-University Erlangen-Nurenberg, Germany

## Abstract

Plant infection by oomycete pathogens is a complex process. It requires precise expression of a plethora of genes in the pathogen that contribute to a successful interaction with the host. Whereas much effort has been made to uncover the molecular systems underlying this infection process, mechanisms of transcriptional regulation of the genes involved remain largely unknown. We performed the first systematic *de-novo* DNA motif discovery analysis in *Phytophthora*. To this end, we utilized the genome sequence of the late blight pathogen *Phytophthora infestans* and two related *Phytophthora* species (*P. ramorum* and *P. sojae*), as well as genome-wide *in planta* gene expression data to systematically predict 19 conserved DNA motifs. This catalog describes common eukaryotic promoter elements whose functionality is supported by the presence of orthologs of known general transcription factors. Together with strong functional enrichment of the common promoter elements towards effector genes involved in pathogenicity, we obtained a new and expanded picture of the promoter structure in *P. infestans*. More intriguingly, we identified specific DNA motifs that are either highly abundant or whose presence is significantly correlated with gene expression levels during infection. Several of these motifs are observed upstream of genes encoding transporters, RXLR effectors, but also transcriptional regulators. Motifs that are observed upstream of known pathogenicity-related genes are potentially important binding sites for transcription factors. Our analyses add substantial knowledge to the as of yet virtually unexplored question regarding general and specific gene regulation in this important class of pathogens. We propose hypotheses on the effects of *cis*-regulatory motifs on the gene regulation of pathogenicity-related genes and pinpoint motifs that are prime targets for further experimental validation.

## Introduction

Oomycetes are an important class of eukaryotic pathogens that have severe ecological and economic impact [1], which only recently entered the genomic era [2], [3]. The genus *Phytophthora* contains several well-known species such as the potato and tomato late blight pathogen *Phytophthora infestans* [4], the stem and root pathogen of soybean *Phytophthora sojae* [5], [6] and the sudden oak death pathogen *Phytophthora ramorum* [5], [7]. The genome sequence of these pathogens facilitated insights into the large repertoire of proteins involved in the interaction with the host [8]. For example, proteins containing the amino-acid motifs RXLR and LXLFLAK (Crinkler) belong to two distinct classes of effectors that are targeted to the inside of the plant cell presumably to promote infection of the host [4], [9], [10]. Elicitins (ELIs) are proteins that elicit defense responses and induce necrosis whereas the related elicitin-like proteins (ELLs) do not exhibit such an activity [11]. Present hypotheses on the functions of ELLs are still inconclusive, but some members seem to be associated with the cell wall or the cell membrane [11]. Genes encoding effectors and also other proteins that are involved in the host-pathogen interaction require a precise spatial and temporal expression to facilitate the successful colonization of the host.

There is rich and continuously expanding knowledge on the regulation of the spatio-temporal expression of genes in human and in eukaryotic model organisms such as yeast and fruit fly (e.g. [12]–[18]). In eukaryotes, regulation of transcription is accomplished by the complex interplay of several elements. These include DNA motifs in the upstream regions of genes (*cis*-regulatory elements), which are bound by diverse transcription factors, and the remodeling of the chromatin structure. Elements in proximity to the transcription start site include the eukaryotic core promoter elements as well as specific regulatory elements. The basic transcriptional activity is determined by the eukaryotic core promoter, which is typically present within 70 nucleotides (nt) surrounding the transcription start site (TSS) and directs the mediator complex, general transcription factors, and the RNA polymerase II (RNA Pol II) into a functional pre-initiation complex [19]–[21]. The core promoter in many eukaryotes consists of different combinations of functional DNA motifs: the transcription factor-B recognition element (BRE), followed by the TATA-box, the initiator (Inr) (located at or around the TSS), and the downstream promoter elements (DPE). The CCAAT-box, another common eukaryotic promoter element, mainly occurs upstream of the core promoter elements. In contrast to other eukaryotes, oomycetes seem to lack canonical TATA-box elements [22]. However, many genes have an Inr-element that resembles the general eukaryotic Inr-element [23], [24]; an element that is sufficient to direct the accurate transcription in the absence of other elements [24]–[26]. Interestingly, oomycetes have a flanking promoter region (FPR) downstream of the Inr-element that has not yet been described as an important functional region in other eukaryotes [24]. Our knowledge on specific promoter elements in oomycetes is limited: The upstream regions of the sporulation-specific genes *Cdc14* and *Pks1* contain, next to the Inr- or Inr/FPR- element, specific but distinct elements that are required for correct gene expression [27], [28]. Additionally, a short (7nt) motif named cold-box mediates temperature-induced expression of zoosporogenesis-specific genes [29]. This small number of experimentally characterized *cis*-regulatory elements in *Phytophthora* is in sharp contrast to the abundance of the predicted genes encoding the diversity of transcription factors in *Phytophthora* and related non-pathogenic species (Table S1A) [30]. This raises questions about the nature and abundance of the accompanying and not yet described *cis*-regulatory elements in the genomes of *Phytophthora* spp.

To expand our knowledge on the transcriptional regulation in *Phytophthora* spp., we systematically inferred and analyzed DNA motifs. We adopted *in silico* methodologies that have been successfully applied to other eukaryotic pathogens, such as the malaria parasite *Plasmodium falciparum* [31], and plants [32]. It is assumed that co-expressed genes share similar *cis*-regulatory motifs [33] and that functional motifs are conserved both within and between species to a higher extent than non-functional DNA. With the availability of genomic and transcriptomic data of several *Phytophthora* spp. [4], [5] similar methodologies can now also be applied to analyze *cis*-regulatory motifs in these important plant pathogens. We combined the upstream regions of co-expressed genes in *P. infestans* with the upstream regions of their orthologs in *P. sojae* and *P. ramorum* and predicted in total 19 motifs. The analysis of this repertoire revealed a complex picture of the *Phytophthora* promoter and allowed the identification of biologically relevant motifs. Several of these motifs are predicted upstream of genes encoding known effector genes or transcriptional regulators, e.g. Myb-like transcription factors. These motifs thus represent interesting candidates for further experimental validation. Hence, our study represents the first systematic characterization of *cis*-regulatory elements in *Phytophthora* spp. and expands our knowledge on the regulation of gene expression in this important class of pathogens.

## Materials and Methods

### Identification of Co-expressed *P. infestans* Genes

We retrieved NimbleGen microarray data of *P. infestans* containing three *in vitro* stages (different media types) and four *in planta* stages [4] from GEO [34]. The initial analysis and summary of the NimbleGen data has been described by Haas *et al*. [4]. Differentially expressed genes during *in planta* growth were identified using t-tests between two groups (group A, different media types; group B replicates for a single data point post inoculation). The tests were independently applied for each day after inoculation and genes were deemed significantly differentially expressed (up- and down-regulated) with a p-value cutoff of 0.05. False discovery rates were assessed by computing q-values (q-value cutoff of 0.05) for each comparison. Subsequently, the identified significantly differentially expressed genes were clustered based on their expression profiles, i.e. intensities relative to the average expression intensity in growth media, using Spearman correlation coefficient utilizing the Markov clustering algorithm (version 09–308, 1.008, inflation 5) [35], [36]. The cutoff for the correlation coefficient was empirically determined by computing the distribution of Spearman correlation coefficients between 1,000 randomly drawn *P. infestans* genes. The correlation coefficient cutoff was determined by the 95 percent quantile, corresponding to value of 0.86. Single, non-clustered genes were discarded before further analysis.

### Identification of Orthologs and Extraction of the 1 kb Upstream Regions in *Phytophthora* spp

We identified orthologs (exclusive in-paralogs within *P. infestans*) of all predicted proteins in the analyzed *Phytophthora* spp. using OrthoMCL (version 2.0; default settings; e-value cutoff 1e-5) [37]. OrthoMCL covers the vast majority of the predicted proteome by grouping on average 84 percent of the predicted proteins into orthologous groups, ranging from 77 percent for *P. infestans* to 91 percent in *P. ramorum*. Subsequently, we combined the upstream regions of co-expressed *P. infestans* genes (clusters with size > = 2) with their orthologs in *P. ramorum* and *P. sojae* (inclusive in-paralogs) and used these to identify conserved DNA motifs. The upstream region per gene was defined as the 1,000 nt upstream of the translation start site ‘ATG’ as annotated by the coding sequence. Upstream sequences without an associated annotated coding gene were discarded. If a coding gene occurred within the 1,000 nt, the upstream region was truncated. For genes located on the negative strand the extracted DNA sequence was converted to its reverse complement. The upstream regions were filtered for the remnants of non-annotated genes by similarity search against the NCBI nr database (downloaded 24.10.2011, blastx [38]; e-value cutoff 1e-3) and the presence of transposable elements identified by TransposonPSI and against the Repbase database [39] (downloaded 19.01.2012, blastn; evalue cutoff 1e-3). Subsequently, all significant hits within the sequences were masked for all further analysis. Furthermore, we tried to reduce the number of false positives during motif prediction by removing highly similar upstream regions as defined by 95 percent identity over an area of at least 50 percent of the length of the informative sequences (one of the sequences was retained).

### Identification of DNA Motifs within Clusters of Co-expressed *P. infestans* Genes and their Orthologs in *P. ramorum* and *P. sojae*

DNA motifs in the upstream regions of different clusters of co-expressed *P. infestans* genes and their orthologs (clusters with size > = 5) were identified using the expectation maximization algorithm implemented in MEME (version 4.6.1; e-value cutoff 1) [40]. We applied the zoops model allowing for zero or single occurrence of a motif per upstream region, inclusion of the reverse compliment DNA strand in the motif identification, a motif length between 4–16 nt, maximally 30 distinct motifs per cluster of co-expressed genes and an empirical 3^rd^ order background Markov model based on the upstream region of all *Phytophthora* spp. genes (this background model is also used for all other analyses).

Similar motifs were clustered into families based on their pairwise similarity using the Markov clustering algorithm (inflation 2). Combined motif logos were produced using Weblogo 3 [41]. The genome-wide abundance of each motif-family was predicted per individual motif constituting the motif-family and the combined motif using FIMO (part of the MEME/MAST package) [42]. FIMO calculates a score for each position within the searched sequence based on the position-specific frequency matrix of the *ab initio* determined motifs. These scores are transformed to p-values and subsequently to q-values to address false discovery rates due to multiple testing. We applied a q-value cutoff 0.1 to define the genome-wide abundance for each motif. The location of the motifs in the upstream regions is displayed for the first 1,000 nt using bins of the size 50. To account for shorter upstream regions due to coding genes within the first 1,000 nt, the abundance was weighted accordingly. Similarity to known motifs was assessed using Tomtom (e-value 0.5; min overlap between motifs 3) [43] against the JASPAR Core and JASPAR PolII database.

To estimate the evolutionary conservation of the identified motifs, we calculated a conservation score that is based on the network-level conservation principle [44], [45]. Assuming that the global gene expression between two closely related species is largely conserved, the network-level conservation principle requires that most of the target sites, i.e. the DNA motifs, are retained. Therefore, we identified the presence of each motif in the upstream regions of orthologous between two of the *Phytophthora* spp. (as determined by OrthoMCL groups, see above). We subsequently calculated the number of cases where both orthologous groups maintained the motif and assessed the significance of the overlap (Fisher exact test, conservation scores are reported as the –log_e_). The values were compared to a set of randomized motifs (the column of each identified motif was shuffled twenty times); the poly-C motif-6 was excluded for this and all subsequent analyses. As expected, the majority of these motifs did not yield any significant hits against the *Phytophthora* upstream regions. Based on the motifs with significant hits we chose the 95 percent quantile as a conservation cutoff, corresponding to a p-value of 0.04. Applying this cutoff to the set of motif families yield a conserved subset that exceed this score between *P. infestans* and at least one of the other *Phytophthora*.

The identified motifs, their genome wide abundance, their conservation score and location (global as well as per individual gene) are accessible as ‘Supplementary data 1’.

### Correlation of Conserved DNA Motifs with Gene Expression Levels upon Infection

Functional *cis*-regulatory motifs are DNA elements that modulate the expression of genes upon binding of a transcription factor. They were identified in *P. infestans* by searching for motifs where their presence within the upstream regions significantly correlates with expression levels of the downstream genes similar to the approach outlined by Bussemaker and colleagues [46]. We searched the upstream region of each of the differentially expressed genes for the binding of one of the individual members of the motif-family using FIMO (default settings, no q-value computation) [42]. For each motif, we retrieved the maximum score per motif-family; the score per hit is defined by the sum of the entries of the position specific scoring matrix. Subsequently, the maximum score is scaled based on the length of the highest scoring motif and the scores for each motif was rescaled in the range [0,10] resulting in a scoring matrix with the dimensions of the number of differentially expressed genes times the number of motifs. Significantly correlated motif scores with the expression level at one of the three different time points (2–4 dpi), expressed as the log_2_-fold change compared to the growth media, were identified by forward variable selection as implemented in R and multiple testing correction was applied to the p-values by computing q-values (false discovery rate). Motifs with a q-value <0.01 were deemed significant. For each motif in each condition a ‘time course value’ (T-value) was calculated: the correlation between the motif score and the expression level at each time point (growth media +2–5 dpi) was transformed into a T-value by multiplying the correlation (r) with the square root of the number of genes (G) (T = r*sqrt(G)) [31].

### Functional Annotation of Genes in the Three Analyzed *Phytophthora* spp

Genes in the analyzed *Phytophthora* spp. were functionally annotated using BLAST2GO algorithm (default parameters) [47]. Functional enrichment of GO terms of genes sharing predicted motifs was conducted with the BiNGO package 2.44 (default parameters) [48] included in Cytoscape 2.8.1 [49]. Significantly enriched GO terms were summarized by removing redundancies using REVIGO (similarity cutoff 0.5) [50]. Moreover, additional annotation for genes such as RXLRs, Crinklers, elicitins, and elicitin-likes was added based on the annotation provided by Haas et al. [4], Jiang et al. [11] and the BROAD website (http://www.broadinstitute.org/). Significance of this overrepresentation was assessed using Fisher exact test (p-value cutoff 0.05).

### Identification of Known Transcription Factors Binding Common Eukaryotic DNA Elements

Known transcription factors that bind to common eukaryotic promoter elements were identified by determining orthologs of the human genes (proteins) in oomycetes using OrthoMCL (version 2.0; default settings; e-value cutoff 1e−5) [37]. The version and source of the nineteen proteomes included in this analysis are shown in Table S1C. In the case of *CBF-B*, OrthoMCL clustered the human gene solitarily and the orthologs of the *Arabidopsis thaliana CBF-B* gene were reported.

### Description of the Transcription Factor Repertoire in *Phytophthora* spp

We predicted the repertoire of potential transcription factors in the proteomes of the three analyzed *Phytophthora* spp. and four non-pathogenic sister taxa (Table S1C) using Pfam models that describe DNA binding sites. The majority of models have been obtained from DBD [51] and some, e.g. Myb-like DNA binding domain, have been added manually (see Table S1A for details). Domains were identified using HMMER3 applying the gathering cutoff [52].

## Results

### Identification of Conserved DNA Motifs in Promoters of *Phytophthora* Genes

**Figure 1:**
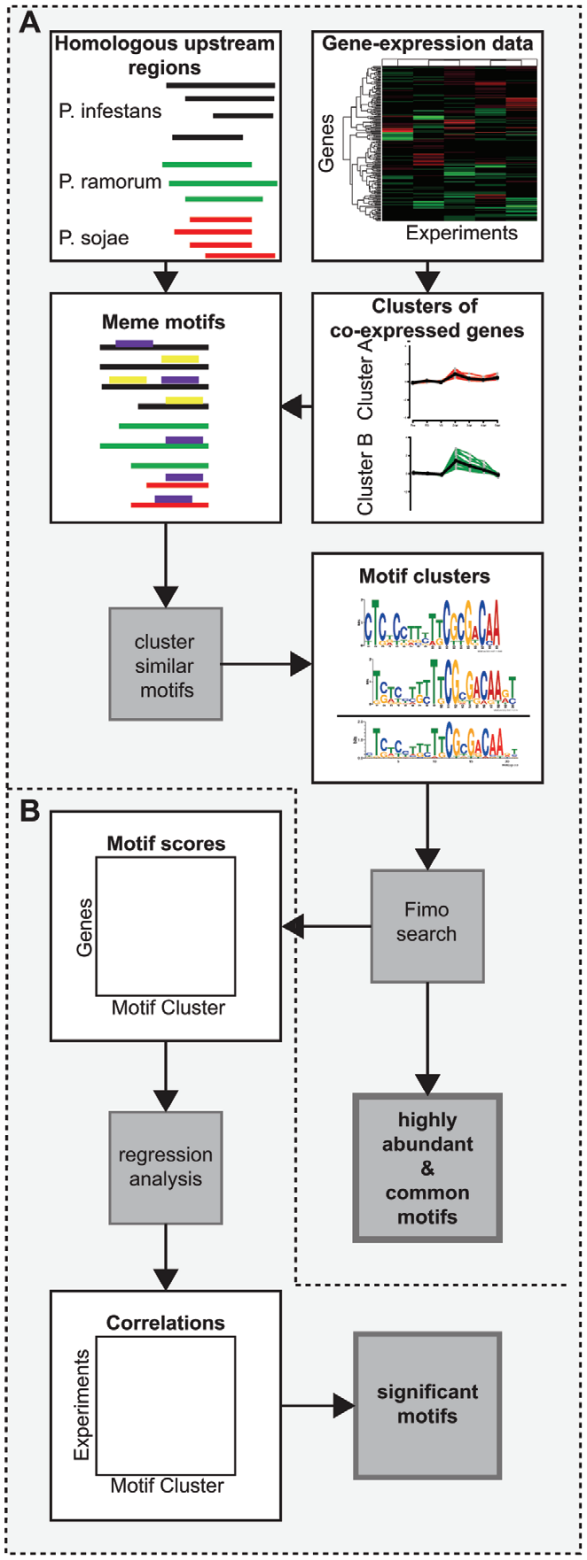
Analysis pipeline used to identify conserved DNA motifs in the three analyzed *Phytophthora* spp. (A) Co-expressed *P. infestans* genes were identified and their upstream regions were combined with the ones from orthologous genes in *P. sojae* and *P. ramorum*. In total 80 motifs were identified and similar motifs were grouped into 24 motif families of which 19 remained after conservation filtering. These were automatically and manually inspected for similarity to known eukaryotic promoter elements. (B) To further assess the biological relevance of the motifs, scores describing the occurrence of motifs in each individual upstream region were assigned. The motif score was correlated with the gene expression level of the downstream genes; an approach similar to the one applied by Bussemaker and colleagues. Subsequently, motifs that have a significant correlation with the expression level of genes during infection were identified (q<0.01).

To predict potential *cis*-regulatory elements in the upstream regions of *Phytophthora* genes, we assumed that co-expressed genes are co-regulated by shared *cis*-regulatory elements [33]. In total, 1,667 differentially expressed *P. infestans* genes were selected from NimbleGen microarray data of *in vitro* growth (three plant extract media) and *in planta* growth (four conditions) 2–5 days post inoculation (dpi) of potato plants [4]. The first three conditions (2–4 dpi) coincide with the formation of haustoria, specialized infection structures that are formed inside the plant cells. The later stage of infection (5 dpi) corresponds to necrotrophic growth on dead plant material where the expression of many genes show similar expression profiles to growth in plant extract media [4]. By clustering the expression profiles of the differentially expressed *P. infestans* genes using Spearman correlation and a graph based clustering algorithm (MCL) [35], we obtained 159 groups of co-expressed genes (Figure 1A; Material & Methods). For each gene within the co-expressed cluster we identified orthologs in two related species (*P. sojae* and *P. ramorum*) and filtered the upstream regions for remnants of transposable elements (see Material & Methods).

Within 136 co-expressed clusters, we identified 80 motifs representing putative regulatory DNA elements in the upstream regions of co-expressed *P. infestans* genes and their orthologs. Similar motifs, especially common eukaryotic DNA elements, were identified in different clusters of co-expressed genes. Hence, we grouped the total of 80 motifs into 24 distinct motif families (called ‘motifs’ throughout the remainder of the manuscript), based on the assumption that all motifs within a family represent a binding site for a specific DNA binding protein or complex. To enrich our results for conserved functional DNA motifs, these were filtered by applying an evolutionary conservation filter between *P. infestans* and at least one of the other *Phytophthora* yielding 19 conserved DNA motifs for which the genome-wide abundance was determined using FIMO (Data S1; Material & Methods).

### Promoters of *Phytophthora* Contain Common Eukaryotic Promoter Elements in High Abundance

**Figure 2:**
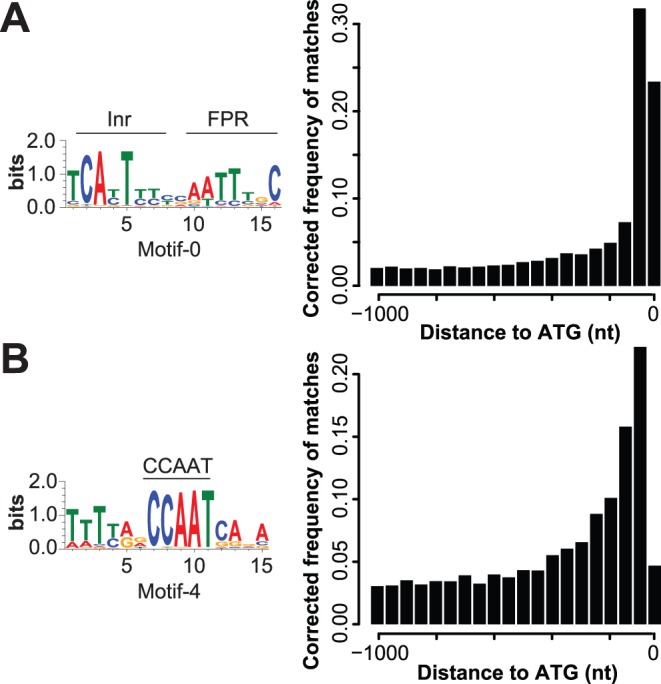
Common eukaryotic promoter elements in *Phytophthora*. Sequence motif of the (A) Inr/FPR-elements and the (B) CCAAT-box identified in the upstream regions of *Phytophthora* spp. genes. The location of the motif in relation to the TLS is indicated by bar charts (bin size 50 nt). The frequency of the motif per bin was weighted according to the underlying length distribution of the upstream regions.

To validate our method, we first surveyed the 19 obtained motifs for similarity to known eukaryotic promoter elements. Pre-genome analyses of the upstream regions of a small set of oomycete genes have identified a Inr/FPR-element as a core promoter element [23], [24]. Indeed, our *in silico* approach recovered the previously described oomycete-specific Inr/FPR element (motif-0). In the set of 1,493 *P. infestans* genes included in the motif search it occurs 652 times. Genome-wide, the Inr/FPR-element is the most abundant motif (Material & Methods): It is predicted in 18,138 upstream regions of all annotated genes in the three analyzed *Phytophthora* spp., and in 6,511 or 37 percent of all *P. infestans*. It has a distinct localization at a median of 81 nt upstream of the translation start site (TLS) (Figure 2A). In other eukaryotes, the transcription factors TAF1 and TAF2 are associated with the transcription factor II D complex during the initiation of transcription at the Inr-element [15], [53]. *Phytophthora* spp. and also other oomycetes have *TAF1* and *TAF2* orthologs (Table S1B), suggesting the association of these transcription factors with the Inr/FPR-element and further supporting its role as a ubiquitous core promoter element in oomycetes.

Among the 19 automatically derived motifs, motif-4 is significantly similar to the eukaryotic CCAAT-box, also named NFYA- or CBF-B binding box (Figure 2B & Tab S2). This common eukaryotic DNA element was so far only reported for a few *Phytophthora* genes [54]. In the three *Phytophthora* spp., we predicted the CCAAT-box in the upstream regions of 8,225 genes, 3,418 of which are from *P. infestans*. It is primarily localized at 192 nt upstream of the TLS. When the CCAAT-box co-occurs with the Inr/FPR-elements (3,321 genes), these motifs are approximately 180 nt apart (80nt for the 25th percentile). Interestingly, we found more occurrences of the CCAAT-box on the negative strand than on the positive strand (4,310 vs. 3,915), consistent with the observation that the CCAAT-box is found in both orientations [55], [56]. The CCAAT-box binding factor is a heterotrimeric protein complex composed of CBF-A, CBF-B and CBF-C [57]. We found orthologs of all three CBF-encoding genes in all *Phytophthora* and in other oomycetes species analyzed (Table S1B), showing additional support for a function of this motif in the regulation of gene expression in oomycetes.

### Enrichment of Distinct Functional Classes in the Sets of Genes with Common Eukaryotic Promoter Elements

**Figure 3:**
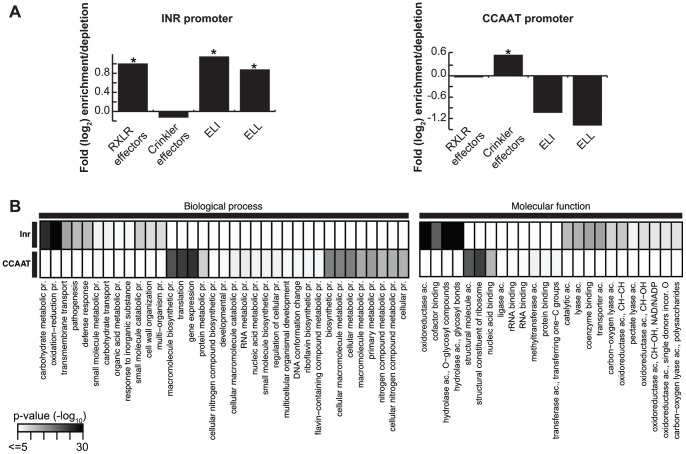
Enrichment/depletion of functional classes in the set of genes with Inr/FPR- or CCAAT-box elements. (A) Log_2_-fold enrichment/depletion displayed for four classes of genes (RXLR, Crinkler, ELI and ELL) predicted to contain the Inr/FPR- or the CCAAT-box in their promoter sequence. (B) Overrepresentation of GO functional annotation of genes that contain the Inr/FPR- or the CCAAT-box elements in their promoter sequence. Heat map shows the -log_10_(p-value) of the significant enrichments detected by BiNGO [48]. Non-redundant GO terms (see Material & Methods) with a −log_10_(p-value) >5 are displayed (see Table S3A for the full list).

To assess whether the described common eukaryotic promoter elements are observed upstream of distinct set of genes, we searched for enrichment of functional Gene Ontology categories as well as other classes associated with host-pathogen interaction, e.g. RXLR effector genes. The sets of genes of which the upstream region contains either the Inr/FPR-element or the CCAAT-box are enriched for different functional categories (Figure 3 & Table S3A). The set with the Inr/FPR-element is highly enriched for RXLR effector, ELI- and ELL genes, and also other genes with predicted functions in pathogenesis, carbohydrate metabolism, glycoside hydrolysis-, oxidoreductase, lyase- or transporter activity; many have a predicted extracellular localization (Table S3A). Strikingly, 869 of the 1107 predicted RXLR effector genes in the three *Phytophthora* spp. contain the Inr/FPR-element in their upstream regions including several up-regulated RXLR effectors (Table 1). In contrast, the set of genes with promoters that exclusively contain the CCAAT-box is depleted of RXLR effector, ELI- and ELL genes and enriched for Crinkler genes (160 out of 600 Crinkler genes). Furthermore, the CCAAT-box set is enriched for genes encoding proteins with a predicted intracellular localization, as well as gene products involved in gene expression, translation, reproduction and developmental- or metabolic processes. The surprisingly strong adjustment of common eukaryotic promoter elements, such as the Inr/FPR, towards pathogenicity and the strong, opposing functional enrichment of genes regulated by either CCAAT-box or Inr/FPR-element is yet another striking example of successful genome adaptation towards pathogenicity within *Phytophthora*.

**Table 1:** Motifs in the upstream regions of a subset of differentially expressed *P. infestans* genes.

					Fold (log_2_)			
Motif	Gene Description	Gene ID	Position$	Sequence*	2dpi	3dpi	4dpi	5dpi
Motif-0	*avr4*	PITG_07387	−36	cgagc**TCAGTCTTCAATTCTC**ccttt	2.06	1.42	0.90	0.37
Motif-0	Pred. RXLR effector	PITG_00821	−62	cagca**TCATTCTTCAACTCGC**aacac	0.80	0.23	−0.002	0.12
Motif-0	Pred. RXLR effector	PITG_02860	−49	caccc**TCATTCTTCAATTCTT**cgact	1.68	1.97	1.15	0.26
Motif-0	Pred. RXLR effector	PITG_12057	−24	agtag**TCATTTCGCTTCTTGC**aggtg	2.27	1.65	1.15	0.30
								
Motif-1	*avr1* family	PITG_16663	−389	cgaac**TACATGTATAT**cccgc	1.42	0.99	0.38	0.07
Motif-1	*avr2* family	PITG_08278	−119	ctcag**TACATGTAA**ccccg	0.98	0.96	0.42	0.39
Motif-1	Mannitol dehydrogenase	PITG_00972	−352	acatg**TACATGTAt**taat	2.06	2.67	0.85	−0.11
Motif-1	Polygalacturonase	PITG_21247	−108	gatgg**TACATGTAC**acggg	0.67	0.01	−0.21	0.13
								
Motif-3	Pred. RXLR effector	PITG_09218	−108	ttgaa**TGCAAATACTAAGTCA**aactg	2.48	1.89	1.15	−0.59
Motif-3	*avrblb2* family	PITG_20303	−181	aagtc**TTACTAATATCAAGTC**gattt	2.02	2.03	1.34	0.23
Motif-3	Myb-like transcription factor	PITG_00513	−709	agtta**ACTTGTTTTATGTAAG**tccca	0.73	0.58	0.33	0.32
Motif-3	Aldose1-epimerase	PITG_14720	−279	aagta**TACAGAAGTCAAGTCA**aatga	1.86	0.96	0.55	−0.50

Predicted motifs in the upstream region of a selected subset of differentially expressed *P. infestans* genes. The fold (log_2_) expression change is compared to the average expression level in growth media.

$ Position of the start of the motif relative to the translation start site (TLS) of the gene.

* Sequence of the motif (capital letters and bold) and 5 nt upstream and downstream of the motif (small letters) are shown for *P. infestans* genes.

### Candidate *Cis*-regulatory Elements that Correlate with Gene Expression Levels upon Infection

To further assess the functional significance of the 19 motifs, we correlated the gene expression levels of the differentially expressed genes with the occurrence of the motifs with a regression-based approach similar to the one described by Bussemaker and colleagues [46] (Figure 1B; Material & Methods). Four motifs show significant positive correlation between the level of motif occurrence and expression levels at one or more of the three time points post inoculation (2–4 dpi; q<0.01). Hence, these four motifs are likely functional binding sites for transcription factors and involved in the regulation of expression of the upstream genes during infection.

**Figure 4:**
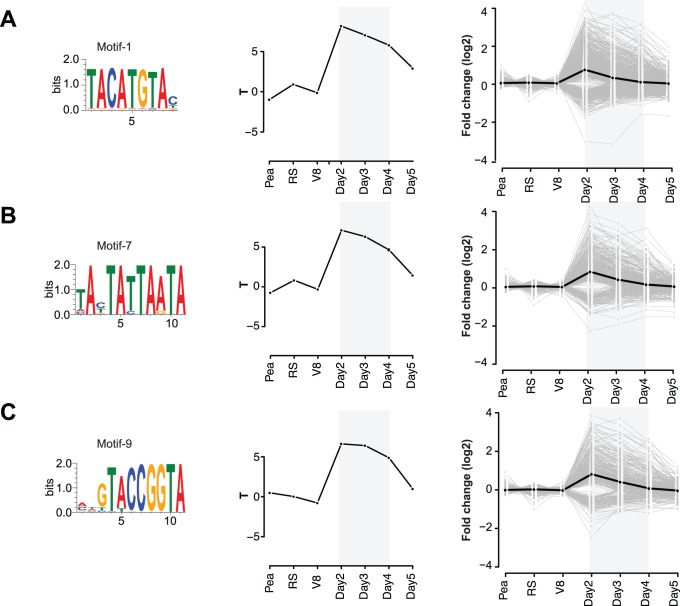
*Cis*-regulatory elements that correlate with gene expression levels during infection. Nucleotide conservation of (A) motif-1, (B) motif-7 and (C) motif-9 is displayed as sequence logos. The T-values for each motif are displayed for each data point as well as gene expression of all differentially expressed genes that contributed to the correlation (see Material & Methods) are displayed.

We identified a novel DNA motif (motif-1) that does not show any significant similarity to known motifs as determined by a Tomtom search against the JASPAR database (Table S2). Motif-1 is a highly abundant and conserved motif that is characterized by the consensus inverted repeat sequence TACATGTA and is identified in total in the upstream regions of 12,070 *Phytophthora* genes, 44% of which are from *P. infestans* (Figure 4A). The inverted repeat structure is suggestive of a binding site for a homodimeric transcription factor. The presence of motif-1 is significantly correlated with the up-regulation of *P. infestans* genes at 2–4 dpi. Interestingly, the set of genes that contain this motif in their upstream region is enriched in genes encoding RXLR effectors and genes involved in cell wall organization, carbohydrate metabolism as well as for genes that encode catalytically active proteins, e.g. glycosyl-hydrolases and oxidoreductases (Figure 4 & Table 1 & Table S3). The differentially expressed mannitol-dehydrogenase gene (PITG_00972) is an example of an oxidoreductase within the enriched class of catalytic enzymes that is up-regulated early during infection (Table 1). Mannitol can suppress of ROS-related plant responses upon secretion in the apoplast and could act as a carbohydrate reservoir [58]–[61]. It has been suggested that mannitol-dehydrogenases (e.g. *MAD1*) in the biotrophic fungal plant pathogen *Uromyces fabae* are responsible for the production of mannitol in haustoria [61], an activity that could also occur in oomycete pathogens. Another example of a stress response gene is a highly expressed (11-fold increased expression at 2 dpi) secreted catalase-peroxidase (PITG_07143) that could act in counteracting the burst of reactive oxygen species (ROS) by the plant as a defense mechanism upon pathogen infection [62].

We also identified an inverted repeat, AT-rich motif (motif-7) in 1,388 *Phytophthora* genes, 940 of which are from *P. infestans* (Figure 4B). This motif shows remote similarity to the eukaryotic TATA-box; a eukaryotic core promoter element that is found in the upstream regions of a quarter of all genes in yeast and human [16]. Previous analyses of the transcriptional regulation of oomycetes have indicated that oomycete promoters do not contain a canonical TATA-box [22], however non-canonical TATA-box elements that resemble functional TATA-box elements have been discovered in oomycetes before [54], [63]. Unlike the Inr/FPR element and CCAAT-box, the TATA-like motif does not have a strong positional preference compared to the canonical TATA-box observed in other eukaryotes or the Inr/FPR-element and CCAAT-box in oomycetes. The set of genes with the TATA-like motif in their upstream regions is enriched for genes encoding RXLRs and ELIs and otherwise do not show any significant enrichment for Gene Ontology categories.

Another novel and abundant conserved DNA motif that shows correlation with gene expression during the infection is motif-9. This inverted repeat motif occurs upstream of 1,284 genes and the set of genes is enriched for RXLR effectors (3 fold). These conserved DNA motifs (motif-1, motif-7 and motif-9) are highly abundant in *Phytophthora* genomes, are correlated with the infection-related gene expression levels and are enriched in specific functional categories. Moreover, one of the four positively correlated motifs is the Inr/FPR-element, further emphasizing the adaptation of basic cellular machinery towards pathogenicity (Figure S1). Hence, the four DNA motifs are relevant candidates for *cis*-acting transcriptional regulatory DNA motifs in pathogenic oomycetes.

### Highly Abundant Motifs in the Genomes of *Phytophthora* spp. are Candidate Binding-sites for Transcriptional Regulators

**Figure 5:**
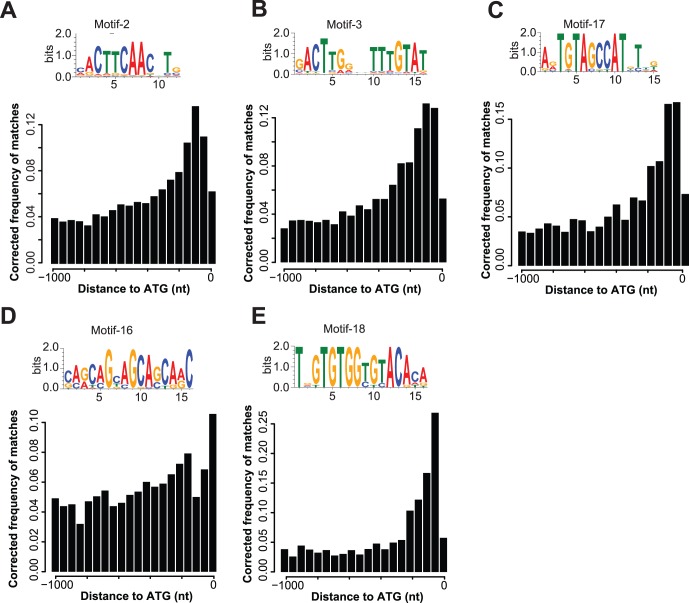
Highly abundant *cis*-regulatory elements. Nucleotide conservation of the five (A-E) highly abundant *cis*-regulatory motifs is displayed as sequence logos. The location of the motif in relation to the TLS is indicated by bar charts (bin size 50 nt). The frequency of the motif per bin was weighted according to the underlying length distribution of the upstream regions.

We expanded the number of candidate motifs by focusing on the ten most abundant motifs within the set of the 19 automatically derived conserved DNA motifs in the upstream regions of the three *Phytophthora* species. These ten motifs include the two common promoter elements (Inr/FPR and CCAAT-box) earlier described, three motifs whose presence is correlated with gene expression levels during infection (motif-1, motif-7 and motif-9) and five additional candidate motifs (Figure 5). Whereas the remaining nine motifs occur in less than 100 different upstream regions, these five motifs occur in high abundance in the upstream regions of *Phytophthora* spp., ranging from 12,034 for motif-2 down to 1,397 occurrences for motif-18.

The most abundant of the five motifs is motif-2 which occurs upstream of 12,034 genes. It is a highly conserved CTTCAAC nucleotide motif that is shows localization preference at 260 nt upstream of the translation start site (Figure 5). The set of genes with motif-2 in their upstream region is significantly enriched in proteins with acyl-CoA dehydrogenase and transporter activity (Tab S3C). In total, 606 of the 12,034 genes encode proteins involved in transporter activity, including e.g. a MOP flippase (PITG_00021) as well as a potential sugar transporter (PITG_00917).

Motif-3, the second most abundant motif, is identified upstream of 5,249 genes, 1,767 of which belong to *P. infestans* (Figure 5B). Twelve of the 105 differentially expressed RXLR effector genes in *P. infestans* have this motif in their upstream region, including a member of the *Avrblbl2* family (Table 1). In contrast to motif-2, the set of genes containing this motif in their promoter is enriched for genes encoding proteins with DNA binding functions (GO: 0003677), many of which are targeted to intracellular organelles (GO: 0043229) such as the nucleus. Of the total of 5,249 genes, 727 genes have either of the two functional annotations and 119 genes share both. These 119 genes include members of the Myb-like transcription factor family (e.g. PITG_00513 that is significantly up-regulated during infection; see Table 1), genes encoding for transcription factors with basic leucine zipper domains (e.g. PITG_00964), but also genes encoding chromatin remodelers such as histone deacetylases (e.g. PITG_01897). However, the majority of these genes in *P. infestans* does not show differential expression during the infection process. The high abundance of motif-3 in the genomes of *Phytophthora* spp. and the highly significant enrichment of genes with predicted functions as transcriptional regulators highlight the prominent role of motif-3 as a protein-binding site. Hence, the transcription factor binding motif-3 is a central regulator and an important target for identification of the binding transcription factors and further experimental studies.

Moreover, we identified three additional highly abundant motifs, motif-17, motif-16 and motif-18 (Figure 5C–E, Table S3C). These motifs are found upstream of 1,938, 1,724 and 1,397 genes, respectively. The set of genes containing motif-17 in their upstream region is enriched for genes encoding proteins involved in transferase-activity and amino-acid metabolism, whereas the sets containing motif-16 and motif18 are enriched for functions such as ATPase activity or intracellular transport, respectively (Tab S3C). Even though the presence of these five motifs is not correlated with the expression levels at 2–4 dpi, they are interesting candidates because of their high abundance and the functional enrichment within the set of genes that have these motifs in their promoter.

## Discussion

Infection of host plants by an oomycete is a complex process that requires the precise expression of proteins encoded in the pathogen’s genome. Infection-related proteins directly or indirectly facilitate the tight interaction with the host by suppressing immune responses triggered by the pathogen. How the expression of this complex arsenal of genes, but also other genes encoded in their genomes, is precisely regulated is still largely unknown. To identify *cis*-regulatory motifs that characterize the promoter regions in *Phytophthora* genes, we adopted methods that utilize genome and transcriptome data to systematically predict conserved DNA elements in genes co-expressed during infection. Our approach yielded 19 potentially active *cis*-regulatory elements, a number that is comparable to similar studies conducted in plants that yielded 34 potential DNA binding sites [32]. Only very few of the 19 motifs show significant similarity to known *cis*-regulatory motifs (Table S2). The discovery of novel *cis*-regulatory elements is an important first step towards understanding the regulation of gene expression in oomycetes.

**Figure 6:**
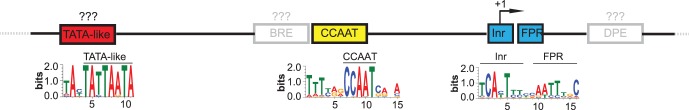
Architecture of *Phytophthora* promoter. Structure of the *Phytophthora* promoter defined by identified DNA motifs determined by our analysis or proposed to be present by indirect evidence. Identified consensus motifs are displayed below for the CCAAT-, the AT-rich TATA-like- and the Inr/FPR-element. Gene ids of the orthologs of the TFs described to bind BRE and DPE are in Table S1C.

We identified a complex promoter structure that expands our view of the central transcriptional regulation machinery of oomycetes (Figure 6). Next to the Inr/FPR-element, we identified and quantified other known eukaryotic elements such as the highly abundant CCAAT-box (Figure 2). We identified an AT-rich motif (motif-7) that could represent a functional TATA-like element. Whether the observed AT-rich element is in fact functionally equivalent to the TATA-box element observed in other eukaryotes is unknown. We identified an ortholog of the TATA-box binding protein (TBP) that is encoded in the genomes of all analyzed *Phytophthora* spp. (Table S1B). The presence of the TBP suggests that AT-rich promoter elements can be bound by the TBP, thereby recruiting the RNA Pol II to the TSS to initiate transcription, especially since it has been shown that TBP binds to a huge variety of AT-rich sequences. Hence, the AT-rich TATA-like box together with the TBP suggests that oomycetes contain functional TATA-box-like elements similar to that of other eukaryotes.

Our analysis did not identify motifs with similarity to the eukaryotic BRE-element or downstream promoter element (DPE), two motifs that are frequently observed as core elements in eukaryotic promoters. However, we found orthologs of the transcription factor II B (TFIIB) as well as the binding factors for the DPE-elements in all *Phytophthora* spp. and in the other oomycetes (Table S1B). Hence, the presence of the necessary molecular factors encoded in the genomes of oomycetes is an indication for the presence of these elements or of non-canonical, functional replacements in the promoter of oomycete genes.

Only 37% of all genes have an Inr/FPR-element; a percentage that is lower than reported for the eukaryotic Inr-element present in various eukaryotes such as human and yeast (46% and 40%, respectively) [16]. It is possible that our pipeline did not automatically predict core promoter elements (e.g. BRE/DPE) or that we underestimated the overall abundance of other motifs, since we searched for motifs within the upstream regions of genes co-expressed under a distinct biological condition. The thereby derived motifs might be biased towards certain nucleotide conservation at positions that do not necessary reflect the consensus. Hence, in combination with a stringent significance cutoff, the biased motifs would not be able to identify all occurrences in the genome and consequently underestimate the true abundance. This is indeed the case for the upstream region of *ipiO1* gene in which the Inr/FPR-element was initially described [23]. If we specifically searched for the occurrence of this motif in the upstream region, we could identify its occurrence at 28 nt upstream of the TLS. However, on a genome-wide search, the occurrence is not significant due to multiple-testing corrections. For a more elaborate unbiased quantification of the core promoter and also other DNA motifs, the identification of these elements under biological conditions other than the infection process is necessary. Currently, the number of different microarray experiments that monitor the changes in gene expression genome-wide is limited. Additional experiments probing different biological conditions would help to reduce the number of false negatives as well as false positives and provide a concise set of differentially expressed genes that could be used for the identification of stage-specific regulatory elements.

Interestingly, the set of genes that are regulated by different combinations of common eukaryotic promoter elements is enriched for distinct functional classes ranging from metabolism to effector genes (Figure 3). This functional adaptation of common eukaryotic promoter elements has been observed for yeast: TATA-box containing genes are stress-induced and expressed in extremely high or low levels, linking the TATA-box to transcriptional plasticity [64]. Moreover, in plants and humans the CCAAT-box has been reported upstream of genes involved in development, gene expression, translation and general metabolism [32], [65], [66], corroborating our observed enrichments in *Phytophthora* (Fig. 3). Many of the studied gene families, but especially RXLR as well as Crinklers effectors, underwent recent expansions in *Phytophthora* [4], [11], [67], [68]. Identical upstream regions due to very recent duplications could influence the observed opposing enrichment of these classes in the set of genes containing either the Inr/FPR or the CCAAT-box. To test this hypothesis, we removed upstream regions that exceed similarity that could be expected due to functional DNA elements before assessing the enrichment (95% identity over 50 percent of the sequence). Even though quantitatively the results vary slightly, we overall still observed the opposing patterns of enrichment in GO categories and RXLRs as before, indicating the independence of our observation to bias due to very recent duplications.

We identified 17 additional conserved DNA motifs next to the two common eukaryotic promoter elements (Data S1). Several of these motifs are candidates for functional active *cis*-regulatory elements because: (i) they are highly abundant in the analyzed *Phytophthora* spp., (ii) their presence in the promoter of genes significantly correlate with the gene expression level during infection and (iii) the set of corresponding proteins is enriched for interesting functions. Within the four motifs whose presence significantly correlate with up-regulation during infection, we revealed, next to the Inr/FPR and the putative AT-rich TATA-like element, two novel abundant motifs. This number is slightly lower, most likely due to limitations in the gene expression data, but still comparable to a study in *Plasmodium falciparum* that identified twelve motifs which are significantly correlated with gene expression levels [31]. Notably, motifs that are positively correlated occur in a high number of different upstream regions and are inverted repeats, suggestive of a possible binding by a homodimeric transcription factor.

Motif-1 is highly abundant and correlates with up-regulation of genes expression levels during infection. The set of genes containing this motif in their upstream region is enriched for RXLR effector genes as well as genes with catalytic activity such as glycosyl-hydrolases. In *Caenorhabditis elegans,* taCATGta motifs are rare footprints of *Tc1* transposable elements excision [69]. Given the strong conservation of this motif and the high abundance in the analyzed of *Phytophthora* genomes, we do not expect motif-1 to be a transposon footprint. Moreover, a recent analysis of the binding preference of homeodomain DNA-binding domains has identified TACATGTA as the preferred binding site for Irx family transcription factors [70]; a group of transcription factors that is observed in *Drosophila* as well as in vertebrates and containing the Homeobox KN domain (PF05920). Interestingly, this domain is also present in several predicted transcription factors in the analyzed *Phytophthora* (Tab S1A); hence, these might be interesting candidate transcription factors for motif-1 binding.

Like motif-1, motif-3 is highly abundant. Notably, it is also present upstream of ∼700 genes that encode proteins with predicted organelle localization such as the nucleus or DNA binding activity. Of these, 119 have both predicted functional annotations and include several transcription factors of the Myb-like family. The majority of *P. infestans* genes in this set is not differentially expressed during infection. Nevertheless, the high abundance of this motif in the *Phytophthora* genomes and its enrichment in genes encoding nuclear and DNA binding proteins suggests that motif-3 is a functional binding site for an unknown transcription factor that in turn regulates many other transcription factors.

The identification of potential biologically relevant motifs solely by correlating their presence with the gene expression levels is a simplified approach. This is especially apparent in the high variability of expression levels between genes that have one of the correlated motifs in their upstream region (Figure 4). *In vivo* there are many factors that influence the transcription of genes such as the chromatin state, the availability of the binding transcription factors and also the presence of other motifs in the proximity that may act together or antagonistic in a regulatory module. Nevertheless, the combination of different criteria, including significant correlation of motif presence with the gene expression level, allows us to generate a concise list of interesting candidates for pending experimental validation; both of the motif itself as well as of the binding transcription factor.

This analysis provides the first systematic insights in the transcriptional regulation of the late blight pathogen *P. infestans* and two closely related *Phytophthora* species. The identified *cis*-regulatory elements are promising candidates for further experimental validation and identification of the binding transcription factors. In general, biochemical and genetic approaches such as ChIP-Seq are lagging in oomycetes and pathogenic fungi. However, whole genome transcriptomics and thereby derived gene expression data as well as genomic sequences of close relatives will be available in the close future. *In silico* methods such as the one outlined in this study are in an exceptional position to take advantage of these data to gradually close the knowledge gap between well-established model organisms and these important and intriguing groups of pathogens.

## Supporting Information

Figure S1 **Positively correlated core promoter motif with gene expression levels.** The Inr/FPR-element is positively correlated with the gene expression levels during infection. The motif logo, a graph displaying the T-value per data point and the gene expression of all differentially expressed genes that contributed to the correlation are displayed.
(EPS)

Table S1 **Predicted DNA binding domains, orthologs of known transcription factors and analyzed genomes in this study.** (A) List of the identified DNA-binding domains in the predicted proteomes of the three analyzed *Phytophthora* spp. and four non-pathogenic sister taxa and their relative abundances. Domains were either derived from DBD (D) or manually added (M). (B) List of known transcription factors binding eukaryotic core promoter elements and their orthologs in eighteen species including five oomycetes. (C) Names, versions and sources of different eukaryotic proteomes that were used to define the orthologs of known transcription factors as well as the predicted transcription factor repertoire in *Phytophthora* spp.
(XLS)

Table S2 **Tabular overview of the Tomtom results for the analyzed motifs.** Tabular overview of all results retrieved by the Tomtom search against the JASPAR motif database. Significant hits (evalue cutoff 0.01) are highlighted in orange.
(XLS)

Table S3 **Tabular overview of enriched GO terms.** Tabular overview of all significantly enriched GO terms for the set of genes that contain the Inr/FPR element or the CCAAT-box. Redundant GO terms as defined by REVIGO are also indicated.
(XLS)

Data S1 **Overview of the identified motif and their occurrence per gene.**
(ZIP)
