# Supplementary material for: Bioinformatic Inference of Specific and General Transcription Factor Binding Sites in the Plant Pathogen Phytophthora infestans
Source: PLoS One. 2012 Dec 12;7(12):e51295. doi: 10.1371/journal.pone.0051295 (PMC3520976; doi:10.1371/journal.pone.0051295)
Supplement: Data S1 — Overview of the identified motif and their occurrence per gene. (ZIP) [file pone.0051295.s005.zip › Supplementary Data1/overview_per_gene.html]

Overview per Gene


| Species | Gene ID | Length Upstream | Motif ID | Position | Strand | Match |
| --- | --- | --- | --- | --- | --- | --- |
| P. infestans | PITG\_00002 | 1000 | Motif-0 | -574 | - | TCAGTCTTACATCTAC |
|  |  |  | Motif-1 | -430 | - | GTTACATGAAG |
| P. infestans | PITG\_00004 | 454 | Motif-1 | -264 | + | TACATGTAA |
| P. infestans | PITG\_00006 | 1000 | Motif-0 | -55 | + | CATTCCTAATTTCGCC |
|  |  |  | Motif-1 | -326 | + | CATATATGTATGG |
| P. infestans | PITG\_00009 | 1000 | Motif-0 | -413 | - | TCACTTCTCTACTTTG |
|  |  |  | Motif-1 | -31 | + | TACATGTAC |
|  |  |  | Motif-3 | -271 | - | TACTTGGAATTTGTAT |
| P. infestans | PITG\_00010 | 825 | Motif-4 | -315 | + | TCTGATTGGTGAATT |
|  |  |  | Motif-7 | -806 | - | TACTATTAGTA |
|  |  |  | Motif-8 | -235 | - | TATTATTAAATATCA |
| P. infestans | PITG\_00011 | 1000 | Motif-0 | -241 | - | GGAACTGGGAGAATGA |
|  |  |  | Motif-8 | -368 | + | TTTTATTTTTTAAAA |
| P. infestans | PITG\_00013 | 779 | Motif-0 | -81 | - | TCAAATTGGGGAATGA |
| P. infestans | PITG\_00014 | 456 | Motif-7 | -177 | + | TATTACTAATA |
| P. infestans | PITG\_00019 | 272 | Motif-4 | -198 | + | CGCGATTGGACAAAA |
| P. infestans | PITG\_00020 | 272 | Motif-4 | -89 | - | CGCGATTGGACAAAA |
| P. infestans | PITG\_00021 | 861 | Motif-1 | -353 | + | TACATGTAC |
|  |  |  | Motif-2 | -539 | + | CCTTCAACGTGCCTCA |
|  |  |  | Motif-4 | -208 | - | CATGATTGGTTAAAA |
| P. infestans | PITG\_00022 | 1000 | Motif-0 | -41 | + | TCATTTCTCTTTCCGA |
|  |  |  | Motif-1 | -24 | - | GATACGTGTAG |
|  |  |  | Motif-2 | -188 | + | CAGCTTCAACAAG |
|  |  |  | Motif-7 | -774 | - | TACTATTAATA |
| P. infestans | PITG\_00023 | 1000 | Motif-0 | -426 | - | TCATTCTTGCTTTCCA |
|  |  |  | Motif-4 | -178 | + | TTTGATTGGCTGAAA |
| P. infestans | PITG\_00025 | 573 | Motif-1 | -377 | + | TACATGTAT |
|  |  |  | Motif-4 | -298 | + | TTTGATTGGATGAAT |
| P. infestans | PITG\_00026 | 573 | Motif-1 | -204 | + | TACATGTAT |
|  |  |  | Motif-4 | -290 | - | TTTGATTGGATGAAT |
| P. infestans | PITG\_00029 | 222 | Motif-1 | -85 | + | TACGTGTAC |
| P. infestans | PITG\_00030 | 656 | Motif-2 | -511 | - | AGCTTCAACCT |
| P. infestans | PITG\_00031 | 864 | Motif-1 | -272 | + | TGCATGTATTTATTC |
|  |  |  | Motif-4 | -46 | - | TTCGATTGGATAAAA |
| P. infestans | PITG\_00032 | 864 | Motif-1 | -607 | - | TGCATGTATTTATTC |
|  |  |  | Motif-4 | -46 | - | TATGATTGGCTGAAA |
| P. infestans | PITG\_00033 | 879 | Motif-1 | -348 | + | TACATGTAA |
| P. infestans | PITG\_00034 | 879 | Motif-1 | -55 | + | TACATGTAC |
| P. infestans | PITG\_00035 | 642 | Motif-0 | -556 | + | TCATTCTTCAGCTTCT |
|  |  |  | Motif-1 | -622 | + | TACATGTAG |
|  |  |  | Motif-4 | -84 | - | TGTAGGCCAATGGAA |
| P. infestans | PITG\_00038 | 347 | Motif-18 | -265 | - | TTGTGTGGTACACAAA |
|  |  |  | Motif-2 | -184 | + | TCAACCTCAAGCTGCC |
| P. infestans | PITG\_00039 | 1000 | Motif-16 | -518 | - | CCGCAGTCGCTGCAGC |
|  |  |  | Motif-6 | -982 | + | CCCTCCCCCCCCTTAA |
| P. infestans | PITG\_00040 | 1000 | Motif-0 | -96 | + | CACTTTTAAATTGGCC |
|  |  |  | Motif-3 | -205 | + | AATTAGTATTTGTAT |
| P. infestans | PITG\_00041 | 1000 | Motif-0 | -948 | + | TCATTCCCCCAGTGGC |
|  |  |  | Motif-16 | -135 | + | TAACAGCAGCACCGAC |
| P. infestans | PITG\_00042 | 1000 | Motif-1 | -112 | - | CCCTTTTTCATGTAT |
|  |  |  | Motif-3 | -232 | - | AACTTGGTATTAGTAT |
|  |  |  | Motif-7 | -313 | + | TATTACTAATA |
| P. infestans | PITG\_00043 | 470 | Motif-1 | -119 | + | TACATGTTCTTCGAA |
|  |  |  | Motif-8 | -270 | - | TATTATTTTTTATAT |
| P. infestans | PITG\_00044 | 1000 | Motif-0 | -502 | + | GAAAATAGGAAAGTGG |
|  |  |  | Motif-7 | -303 | + | TACTATTAATA |
| P. infestans | PITG\_00045 | 1000 | Motif-0 | -115 | + | GCACTCTCAATTCCGC |
|  |  |  | Motif-7 | -880 | - | TACTATTAATA |
| P. infestans | PITG\_00048 | 224 | Motif-4 | -79 | - | TCGTATTGGATAAAA |
| P. infestans | PITG\_00049 | 224 | Motif-4 | -95 | + | TCGGATTGGTAGAAA |
| P. infestans | PITG\_00050 | 133 | Motif-4 | -64 | - | TTTGATTGGACATAA |
| P. infestans | PITG\_00051 | 156 | Motif-4 | -72 | - | TGTGATTGGATGAAA |
| P. infestans | PITG\_00052 | 156 | Motif-4 | -69 | + | TCTCATTGGCCATTA |
| P. infestans | PITG\_00053 | 130 | Motif-0 | -122 | + | GAAAATATCAAAGTGA |
| P. infestans | PITG\_00054 | 130 | Motif-0 | -24 | - | GAAAATATCAAAGTGA |
| P. infestans | PITG\_00056 | 1000 | Motif-0 | -100 | + | CATTCCAGCTTTTGCC |
|  |  |  | Motif-1 | -476 | - | TACATATAC |
| P. infestans | PITG\_00058 | 1000 | Motif-0 | -412 | - | TCACTTCTCGATTTC |
| P. infestans | PITG\_00059 | 1000 | Motif-1 | -289 | + | TACATGTAG |
| P. infestans | PITG\_00060 | 915 | Motif-2 | -106 | - | GATATTGAAGCTGA |
| P. infestans | PITG\_00061 | 428 | Motif-0 | -20 | + | GCATTCTTCGATTTAT |
|  |  |  | Motif-17 | -310 | + | AGTGTAGCCATGCGA |
| P. infestans | PITG\_00062 | 428 | Motif-0 | -424 | - | GCATTCTTCGATTTAT |
|  |  |  | Motif-17 | -133 | - | AGTGTAGCCATGCGA |
| P. infestans | PITG\_00065 | 406 | Motif-4 | -55 | - | CGTGGTTGGCTGAAA |
| P. infestans | PITG\_00066 | 406 | Motif-4 | -313 | - | TCCCATTTGCTAAAA |
| P. infestans | PITG\_00067 | 1000 | Motif-1 | -760 | + | TACATGCAC |
|  |  |  | Motif-7 | -749 | + | CACTATTAATA |
| P. infestans | PITG\_00068 | 1000 | Motif-1 | -384 | + | TGCATGTAC |
|  |  |  | Motif-2 | -136 | - | TATGTTGAAGTT |
|  |  |  | Motif-7 | -398 | - | CACTATTAATA |
| P. infestans | PITG\_00069 | 558 | Motif-1 | -538 | + | TACATGTAA |
| P. infestans | PITG\_00073 | 449 | Motif-9 | -259 | - | AAGTACCGGTA |
| P. infestans | PITG\_00074 | 449 | Motif-9 | -198 | - | AAGTACCGGTA |
| P. infestans | PITG\_00076 | 1000 | Motif-0 | -737 | - | TCATTTTGCAATCCCT |
| P. infestans | PITG\_00077 | 1000 | Motif-0 | -551 | - | TCACTCTTGGATTCCG |
| P. infestans | PITG\_00078 | 1000 | Motif-0 | -852 | - | GCCGAATTGAAAATGA |
| P. infestans | PITG\_00079 | 1000 | Motif-0 | -330 | - | TCATTCTGGAAATTAA |
|  |  |  | Motif-2 | -399 | - | CACTTCAACCT |
| P. infestans | PITG\_00080 | 175 | Motif-4 | -31 | + | TTTGATAGGCTAAAA |
| P. infestans | PITG\_00081 | 451 | Motif-1 | -370 | - | TGCATGTAC |
| P. infestans | PITG\_00082 | 403 | Motif-0 | -394 | + | GGAGAATGAGAAATGG |
|  |  |  | Motif-1 | -241 | - | GATGCATGTAG |
| P. infestans | PITG\_00083 | 403 | Motif-0 | -25 | - | GGAGAATGAGAAATGG |
|  |  |  | Motif-1 | -173 | + | GATGCATGTAG |
| P. infestans | PITG\_00088 | 1000 | Motif-17 | -966 | + | AGTGAAGCCATCTTT |
|  |  |  | Motif-2 | -718 | - | GTCGTTGAAGTG |
|  |  |  | Motif-4 | -612 | + | TCTGATTGGACCAAA |
| P. infestans | PITG\_00089 | 377 | Motif-0 | -74 | - | GCAAACCGCGAAATGA |
|  |  |  | Motif-4 | -220 | + | TAGGATTGGTTTAAA |
| P. infestans | PITG\_00090 | 377 | Motif-0 | -319 | + | GCAAACCGCGAAATGA |
|  |  |  | Motif-4 | -172 | - | TAGGATTGGTTTAAA |
| P. infestans | PITG\_00095 | 1000 | Motif-1 | -321 | + | TACATGTAA |
| P. infestans | PITG\_00096 | 664 | Motif-0 | -41 | - | GTAAATTGTTAAGTGA |
| P. infestans | PITG\_00097 | 664 | Motif-0 | -639 | + | GTAAATTGTTAAGTGA |
| P. infestans | PITG\_00102 | 1000 | Motif-0 | -41 | - | GTAAATTGTTAAGTGA |
| P. infestans | PITG\_00103 | 1000 | Motif-1 | -453 | + | TACATGTAA |
| P. infestans | PITG\_00104 | 760 | Motif-1 | -61 | + | TACATGTAA |
|  |  |  | Motif-8 | -104 | - | TTTTTTTTTATAGAA |
| P. infestans | PITG\_00108 | 1000 | Motif-0 | -788 | - | GCAAATTGCAATGTGG |
| P. infestans | PITG\_00109 | 1000 | Motif-0 | -25 | - | TTGAGTTGAGGAATGA |
|  |  |  | Motif-8 | -291 | + | TATTTTTAAATATGT |
| P. infestans | PITG\_00110 | 375 | Motif-17 | -367 | - | AATGTAGCCATGTTA |
| P. infestans | PITG\_00111 | 158 | Motif-8 | -67 | + | TATTTTTTAATTGCA |
| P. infestans | PITG\_00112 | 299 | Motif-2 | -278 | + | GAGCTTCAACAAG |
|  |  |  | Motif-8 | -189 | - | TTATTTTTATTTTAA |
| P. infestans | PITG\_00113 | 299 | Motif-2 | -34 | - | GAGCTTCAACAAG |
|  |  |  | Motif-8 | -124 | + | TATTTTTATTTTAAA |
| P. infestans | PITG\_00114 | 1000 | Motif-0 | -358 | - | TCACTCTCCATTTTG |
|  |  |  | Motif-1 | -571 | + | ATTATATGTAC |
| P. infestans | PITG\_00115 | 1000 | Motif-16 | -20 | + | CACCAGCAGCCCCAGC |
| P. infestans | PITG\_00117 | 192 | Motif-17 | -93 | + | AGTGTAGCCATGTTG |
| P. infestans | PITG\_00118 | 419 | Motif-4 | -299 | + | TCTAATTGGCTAAAA |
| P. infestans | PITG\_00119 | 368 | Motif-1 | -140 | + | TACATGTAC |
|  |  |  | Motif-8 | -196 | + | TTTTTTTATTTAAAA |
| P. infestans | PITG\_00121 | 327 | Motif-0 | -64 | - | GCCAATTGGCGAATGA |
|  |  |  | Motif-1 | -235 | + | TACATGTAC |
| P. infestans | PITG\_00122 | 171 | Motif-0 | -145 | + | TCACTCCACTATTTTA |
| P. infestans | PITG\_00123 | 622 | Motif-18 | -488 | + | TAGAGTGGTGCACGCG |
| P. infestans | PITG\_00124 | 622 | Motif-18 | -150 | - | TAGAGTGGTGCACGCG |
| P. infestans | PITG\_00125 | 1000 | Motif-1 | -454 | - | AGCATGTACTGATGC |
|  |  |  | Motif-16 | -631 | - | CCGACGTCGCAGCGGC |
|  |  |  | Motif-2 | -778 | + | CATTTTGAAGTTGA |
| P. infestans | PITG\_00126 | 1000 | Motif-0 | -40 | - | GAGAATTGCTAAATGA |
|  |  |  | Motif-1 | -588 | + | AGCATGTACTGATGC |
|  |  |  | Motif-16 | -412 | + | CCGACGTCGCAGCGGC |
|  |  |  | Motif-2 | -261 | + | AACTTCAAAATGGC |
| P. infestans | PITG\_00129 | 808 | Motif-1 | -166 | + | TACATGTAC |
|  |  |  | Motif-2 | -747 | + | GAAATTGAAGCG |
|  |  |  | Motif-3 | -456 | + | ATTTGACTATTGTAT |
|  |  |  | Motif-8 | -249 | + | TTTAAATAAATTAAT |
| P. infestans | PITG\_00130 | 858 | Motif-1 | -685 | + | TACATGCATT |
| P. infestans | PITG\_00131 | 858 | Motif-1 | -181 | + | TGCATGTATTAACTT |
| P. infestans | PITG\_00132 | 1000 | Motif-1 | -346 | + | TACATGTAG |
|  |  |  | Motif-3 | -671 | + | ACTCGTGCTTTGTAT |
| P. infestans | PITG\_00133 | 1000 | Motif-2 | -990 | - | AGCTTCAACGT |
|  |  |  | Motif-3 | -115 | + | TACAAACATCAAGTAA |
|  |  |  | Motif-4 | -191 | + | GGTGACTGGCTCAAA |
| P. infestans | PITG\_00134 | 202 | Motif-1 | -139 | + | TACATGAAC |
| P. infestans | PITG\_00135 | 202 | Motif-1 | -72 | - | TACATGAAC |
| P. infestans | PITG\_00136 | 1000 | Motif-0 | -294 | + | CACTTCCCAATTTGCC |
| P. infestans | PITG\_00139 | 905 | Motif-6 | -894 | - | TCCCCTTCCTCCCCCC |
| P. infestans | PITG\_00140 | 905 | Motif-6 | -27 | + | TCCCCTTCCTCCCCCC |
| P. infestans | PITG\_00143 | 140 | Motif-4 | -36 | - | TATCATTGGATAAAA |
| P. infestans | PITG\_00144 | 140 | Motif-4 | -81 | - | TTGGATTGGCGGAAA |
| P. infestans | PITG\_00146 | 1000 | Motif-1 | -337 | - | AGTGCTTGTACATATA |
| P. infestans | PITG\_00147 | 1000 | Motif-0 | -58 | + | CATTTCAGAATTTGCC |
|  |  |  | Motif-1 | -260 | + | TACATGTAC |
|  |  |  | Motif-3 | -180 | + | TACAAACTGCAAGACA |
|  |  |  | Motif-9 | -621 | + | CTGTACCGGTA |
| P. infestans | PITG\_00148 | 1000 | Motif-4 | -203 | + | TCGGATTGGTCAATA |
| P. infestans | PITG\_00149 | 1000 | Motif-0 | -286 | + | CAGTTGTGAATTTGCC |
| P. infestans | PITG\_00150 | 1000 | Motif-0 | -84 | + | TCACAATCGAACTCGC |
|  |  |  | Motif-1 | -443 | + | TACATGCAC |
| P. infestans | PITG\_00152 | 1000 | Motif-3 | -289 | + | ATACAAATGTCAATAT |
| P. infestans | PITG\_00153 | 1000 | Motif-6 | -383 | + | CCCCCCTCCCCCTTAG |
| P. infestans | PITG\_00154 | 1000 | Motif-4 | -330 | + | TCTGATTGGTTCAAT |
| P. infestans | PITG\_00155 | 1000 | Motif-0 | -476 | + | CACTTGTCAATTGACG |
|  |  |  | Motif-1 | -405 | + | TACATGTAA |
| P. infestans | PITG\_00156 | 1000 | Motif-0 | -673 | - | CCACTTCCCAATGTCT |
|  |  |  | Motif-3 | -421 | + | TCTTGGCATTTGTAA |
|  |  |  | Motif-4 | -295 | + | CTTCATTCGCCAAAA |
| P. infestans | PITG\_00157 | 287 | Motif-2 | -36 | - | AACTTCAACTT |
| P. infestans | PITG\_00158 | 114 | Motif-4 | -24 | - | TTTCGACCAATTCAC |
| P. infestans | PITG\_00159 | 114 | Motif-4 | -105 | + | TTTCGACCAATTCAC |
| P. infestans | PITG\_00162 | 1000 | Motif-0 | -298 | + | GCGAATTCCGGAGTGA |
| P. infestans | PITG\_00163 | 1000 | Motif-0 | -138 | - | AAAAAATTGGAAGTGA |
|  |  |  | Motif-1 | -219 | + | TACATGTAC |
| P. infestans | PITG\_00164 | 1000 | Motif-1 | -199 | + | GCTACATGAAG |
|  |  |  | Motif-7 | -677 | + | AAGTATTAATA |
| P. infestans | PITG\_00165 | 1000 | Motif-1 | -726 | + | AAAACATGTAAAGAA |
|  |  |  | Motif-4 | -73 | - | TGTGATTGGCCACTT |
|  |  |  | Motif-7 | -414 | - | AAGTATTAATA |
| P. infestans | PITG\_00166 | 493 | Motif-2 | -23 | - | AGGGAGGTCGAAGTTG |
| P. infestans | PITG\_00167 | 1000 | Motif-2 | -715 | + | CACGTTGCAGTT |
| P. infestans | PITG\_00169 | 1000 | Motif-1 | -138 | + | TACATGTAG |
| P. infestans | PITG\_00170 | 483 | Motif-0 | -43 | + | TCACTCGACATTTCAC |
|  |  |  | Motif-2 | -247 | + | CAGCTTCAACGAG |
| P. infestans | PITG\_00171 | 678 | Motif-0 | -334 | + | ACATTTCTCAACTCTA |
|  |  |  | Motif-1 | -233 | + | TACATGTAC |
|  |  |  | Motif-3 | -285 | + | GCTTGTTTTTAGTAT |
|  |  |  | Motif-7 | -52 | - | TAGTATTAATA |
| P. infestans | PITG\_00172 | 119 | Motif-4 | -114 | + | TATTATTGGTCAAAA |
| P. infestans | PITG\_00173 | 382 | Motif-4 | -121 | + | TATCATTGGACAAAT |
| P. infestans | PITG\_00174 | 382 | Motif-4 | -276 | - | TATCATTGGACAAAT |
| P. infestans | PITG\_00175 | 118 | Motif-0 | -20 | + | TCATTCAAGAATCTGC |
|  |  |  | Motif-2 | -45 | - | AACTTCAACAT |
| P. infestans | PITG\_00176 | 1000 | Motif-0 | -118 | - | CAGAAATGAAAAGTGA |
| P. infestans | PITG\_00177 | 1000 | Motif-0 | -42 | - | GAAGATCGGGGAATGA |
|  |  |  | Motif-1 | -347 | - | GATACCGGTACTGTA |
|  |  |  | Motif-2 | -639 | - | CGCTTCAACTT |
|  |  |  | Motif-3 | -687 | - | TCTGAGATTTAGTAT |
|  |  |  | Motif-9 | -345 | + | CAGTACCGGTA |
| P. infestans | PITG\_00178 | 1000 | Motif-0 | -204 | - | GACAGTTGGGAAGTGG |
|  |  |  | Motif-1 | -729 | + | GATACCGGTACTGTA |
|  |  |  | Motif-2 | -432 | + | GCTTCAACTTG |
|  |  |  | Motif-3 | -389 | + | TCTGAGATTTAGTAT |
|  |  |  | Motif-9 | -727 | - | CAGTACCGGTA |
| P. infestans | PITG\_00179 | 219 | Motif-4 | -99 | - | TTTGACTGGTTCAAA |
| P. infestans | PITG\_00180 | 219 | Motif-4 | -135 | + | TTTGACTGGTTCAAA |
| P. infestans | PITG\_00181 | 621 | Motif-4 | -569 | + | GGTGATTGGCTAGAA |
| P. infestans | PITG\_00182 | 621 | Motif-4 | -67 | - | GGTGATTGGCTAGAA |
| P. infestans | PITG\_00185 | 963 | Motif-0 | -22 | - | GCGAAGTGCGGAGTGA |
|  |  |  | Motif-1 | -204 | + | TGCATGTAC |
| P. infestans | PITG\_00186 | 963 | Motif-0 | -81 | + | TCATTATGCGTTTTGA |
|  |  |  | Motif-1 | -548 | + | TACAAGTACT |
| P. infestans | PITG\_00187 | 94 | Motif-4 | -77 | + | AATCAACCAATAGAA |
| P. infestans | PITG\_00190 | 333 | Motif-2 | -235 | - | TCCGTTGAAGTGAC |
| P. infestans | PITG\_00191 | 1000 | Motif-0 | -47 | - | GCAAAGTAGAAAATGG |
|  |  |  | Motif-2 | -567 | - | AAAATTGAAGTTGC |
| P. infestans | PITG\_00192 | 474 | Motif-1 | -340 | + | TACATGTAT |
|  |  |  | Motif-4 | -139 | + | GTTGATTGGCTAATT |
| P. infestans | PITG\_00193 | 771 | Motif-3 | -90 | + | TACTCAGTTCAAGTGA |
| P. infestans | PITG\_00194 | 1000 | Motif-1 | -376 | + | TACATGTAT |
|  |  |  | Motif-3 | -64 | - | GACTTGAGCTTTCTTT |
|  |  |  | Motif-8 | -113 | + | TTTTACTTAATATTT |
| P. infestans | PITG\_00195 | 1000 | Motif-1 | -923 | - | TGTACATGTCAC |
|  |  |  | Motif-2 | -352 | - | AAGGTTGAAGGGGA |
|  |  |  | Motif-8 | -945 | - | TATTTTTTAGCAGAA |
| P. infestans | PITG\_00196 | 265 | Motif-1 | -147 | - | ATGACATGTAT |
|  |  |  | Motif-4 | -190 | + | TGTCATTGGCTAGAA |
| P. infestans | PITG\_00197 | 265 | Motif-1 | -129 | + | ATGACATGTAT |
|  |  |  | Motif-4 | -90 | - | TGTCATTGGCTAGAA |
| P. infestans | PITG\_00198 | 1000 | Motif-0 | -395 | + | GTATATTGAGAAGTGA |
| P. infestans | PITG\_00200 | 1000 | Motif-1 | -565 | + | ACTCTACGTGTAGC |
| P. infestans | PITG\_00201 | 584 | Motif-1 | -158 | + | TACACGTACAAGAAT |
| P. infestans | PITG\_00202 | 584 | Motif-1 | -435 | - | ACTACACGTAC |
| P. infestans | PITG\_00203 | 813 | Motif-1 | -333 | - | ACTACGTGTAG |
| P. infestans | PITG\_00204 | 813 | Motif-1 | -433 | - | TACATGCATT |
| P. infestans | PITG\_00205 | 1000 | Motif-1 | -692 | + | TGCATGTAC |
|  |  |  | Motif-3 | -375 | + | ACTTGACTTCTGTAT |
|  |  |  | Motif-4 | -23 | + | CGTGATTGGTCGAGA |
| P. infestans | PITG\_00206 | 1000 | Motif-1 | -796 | - | TGCATGTAC |
|  |  |  | Motif-2 | -407 | + | GCCGTTGAAGTTGA |
| P. infestans | PITG\_00208 | 479 | Motif-17 | -468 | + | GATGTAGCCATTCGA |
|  |  |  | Motif-2 | -425 | + | TCAGCTTCAAGTCGAC |
|  |  |  | Motif-6 | -223 | + | TCTCCCTCCCCCCACT |
| P. infestans | PITG\_00209 | 1000 | Motif-2 | -87 | - | CACTTCAACTT |
| P. infestans | PITG\_00211 | 1000 | Motif-16 | -566 | - | GCATCGTAGCCGCAGC |
| P. infestans | PITG\_00212 | 578 | Motif-0 | -359 | - | GCAAAATAAAAACTGA |
|  |  |  | Motif-1 | -453 | + | TACATGTAC |
| P. infestans | PITG\_00214 | 655 | Motif-0 | -108 | + | ACACTTTGAAATTCTC |
|  |  |  | Motif-1 | -566 | + | TACATGTAG |
|  |  |  | Motif-2 | -91 | + | CATATTGAAGTG |
| P. infestans | PITG\_00215 | 589 | Motif-1 | -195 | + | TACATGTAT |
| P. infestans | PITG\_00216 | 1000 | Motif-8 | -97 | + | TTTTTTTAAATATTT |
| P. infestans | PITG\_00217 | 469 | Motif-3 | -422 | - | ATTTGCTCTTTCTAT |
| P. infestans | PITG\_00218 | 469 | Motif-3 | -62 | + | ATTTGCTCTTTCTAT |
| P. infestans | PITG\_00219 | 1000 | Motif-0 | -80 | - | GCAAATCGGTGAATGG |
|  |  |  | Motif-1 | -301 | + | TACATGTGACAGTAAA |
|  |  |  | Motif-2 | -836 | + | CATTTTGAAGTG |
|  |  |  | Motif-9 | -401 | + | ACGTACCGGTA |
| P. infestans | PITG\_00222 | 465 | Motif-0 | -385 | - | TCATTATTAAGCTTGC |
|  |  |  | Motif-17 | -326 | - | ACTGTAGCCATATGA |
|  |  |  | Motif-2 | -15 | + | GCTTCAACTCG |
|  |  |  | Motif-3 | -236 | + | ACTTAGGATTTGTAA |
|  |  |  | Motif-4 | -114 | + | GGTGATTGGCTAAAA |
| P. infestans | PITG\_00223 | 175 | Motif-4 | -61 | - | TTTGATTGGACAAAT |
| P. infestans | PITG\_00224 | 175 | Motif-4 | -72 | + | TTCGATTGGATCAAA |
| P. infestans | PITG\_00225 | 108 | Motif-2 | -42 | - | CACTTCAACTT |
|  |  |  | Motif-4 | -23 | + | TGTGATTGGTAGAAA |
| P. infestans | PITG\_00228 | 274 | Motif-4 | -228 | - | TATGATTGGATGAAA |
| P. infestans | PITG\_00229 | 274 | Motif-4 | -61 | + | TATGATTGGATGAAA |
| P. infestans | PITG\_00230 | 215 | Motif-0 | -153 | - | TTATTTTCCCTTTTTC |
|  |  |  | Motif-1 | -159 | - | CCCTTTTTCATGTAT |
| P. infestans | PITG\_00231 | 1000 | Motif-2 | -444 | + | CTCTTCAACGT |
| P. infestans | PITG\_00232 | 1000 | Motif-2 | -895 | - | CTCTTCAACGT |
| P. infestans | PITG\_00233 | 1000 | Motif-0 | -258 | + | TCAGTACTGTACTTAC |
|  |  |  | Motif-1 | -302 | + | TACATGTAC |
|  |  |  | Motif-2 | -27 | - | TGGCTTCAAGATGA |
|  |  |  | Motif-8 | -409 | + | TATGTATAATTTATA |
| P. infestans | PITG\_00234 | 1000 | Motif-0 | -134 | - | GCACTTTGCGTTCTAC |
|  |  |  | Motif-2 | -433 | + | CCAGTTGAAGCGGA |
|  |  |  | Motif-3 | -289 | + | TACTGAAAGCAAGTCA |
| P. infestans | PITG\_00235 | 903 | Motif-0 | -771 | - | CCACTTCCTCATCTCC |
| P. infestans | PITG\_00236 | 133 | Motif-2 | -99 | + | GCTTCAACATG |
| P. infestans | PITG\_00237 | 133 | Motif-2 | -44 | - | AGCTTCAACAT |
| P. infestans | PITG\_00238 | 1000 | Motif-1 | -55 | + | TACATGTAT |
|  |  |  | Motif-7 | -49 | - | TAATATTAATA |
|  |  |  | Motif-9 | -91 | - | CAGTACCGGTA |
| P. infestans | PITG\_00244 | 1000 | Motif-8 | -308 | - | TTTTTTTTAGCAAAA |
| P. infestans | PITG\_00245 | 1000 | Motif-1 | -205 | + | TACATGTAT |
| P. infestans | PITG\_00248 | 674 | Motif-2 | -545 | - | ACTTCAAGGTG |
| P. infestans | PITG\_00249 | 1000 | Motif-3 | -231 | - | TTTTGGCGTTAGTAT |
| P. infestans | PITG\_00251 | 358 | Motif-17 | -350 | + | AATGTAGGCATGTGG |
| P. infestans | PITG\_00252 | 296 | Motif-0 | -181 | + | CCATTCTTCGATCAGC |
| P. infestans | PITG\_00253 | 699 | Motif-0 | -150 | + | GTTTTATTTTTGCTTT |
| P. infestans | PITG\_00254 | 945 | Motif-1 | -547 | + | ATTAAATGTA |
|  |  |  | Motif-2 | -608 | + | GCACTTCAACAGCA |
|  |  |  | Motif-4 | -207 | + | GTCTATTGGATAAAA |
| P. infestans | PITG\_00257 | 418 | Motif-2 | -393 | - | CACATTGAAGTG |
| P. infestans | PITG\_00258 | 418 | Motif-2 | -37 | + | CACATTGAAGTG |
| P. infestans | PITG\_00259 | 206 | Motif-1 | -172 | + | TACATGTAT |
| P. infestans | PITG\_00260 | 298 | Motif-0 | -193 | + | CCATTTTTCAAATTCA |
|  |  |  | Motif-2 | -112 | - | CACTTCAACTT |
| P. infestans | PITG\_00261 | 298 | Motif-0 | -62 | + | TCACTGTCACTTCCTC |
|  |  |  | Motif-2 | -196 | + | ACTTCAACTTG |
| P. infestans | PITG\_00262 | 1000 | Motif-2 | -168 | + | TTGGTTGAAGTG |
|  |  |  | Motif-4 | -662 | - | TCCGATTCGTTGAAT |
| P. infestans | PITG\_00263 | 1000 | Motif-4 | -724 | + | TCCGATTCGTTGAAT |
| P. infestans | PITG\_00264 | 150 | Motif-0 | -142 | - | GCAGTTTCTAATTGGC |
|  |  |  | Motif-4 | -147 | - | TCTAATTGGCAAAAA |
| P. infestans | PITG\_00265 | 290 | Motif-4 | -88 | - | TGCGATTGGCTTAAA |
| P. infestans | PITG\_00266 | 290 | Motif-4 | -217 | + | TGCGATTGGCTTAAA |
| P. infestans | PITG\_00267 | 325 | Motif-1 | -204 | + | TACATGTAG |
| P. infestans | PITG\_00268 | 325 | Motif-1 | -62 | - | TCTATATGTAT |
| P. infestans | PITG\_00269 | 646 | Motif-2 | -311 | - | GACGGCAACTTGCC |
|  |  |  | Motif-4 | -527 | - | GTTGATTGGCTTAAT |
| P. infestans | PITG\_00270 | 440 | Motif-4 | -61 | + | TCTGGTTGGCTGAAA |
| P. infestans | PITG\_00271 | 1000 | Motif-0 | -620 | - | CCACTTTTCTTTCTCC |
|  |  |  | Motif-4 | -242 | - | CCTCATTGGTGGATA |
| P. infestans | PITG\_00272 | 1000 | Motif-0 | -235 | - | GCAAATTGCGGAATGG |
| P. infestans | PITG\_00273 | 707 | Motif-2 | -296 | + | GTCGTTGAAGTT |
|  |  |  | Motif-3 | -98 | - | ACTTATACTTTCTAT |
| P. infestans | PITG\_00274 | 261 | Motif-0 | -98 | + | GAAAAACTAGGACTGA |
| P. infestans | PITG\_00275 | 261 | Motif-0 | -179 | - | GAAAAACTAGGACTGA |
| P. infestans | PITG\_00276 | 1000 | Motif-1 | -760 | + | TACATGTAC |
|  |  |  | Motif-4 | -861 | + | TTTCATTGGCTCATA |
| P. infestans | PITG\_00281 | 279 | Motif-1 | -151 | - | ATCATACAAGTAGC |
| P. infestans | PITG\_00282 | 279 | Motif-1 | -46 | + | ACAATTCATGTACT |
| P. infestans | PITG\_00287 | 915 | Motif-0 | -64 | + | TCACTTCAGTTTCCCC |
| P. infestans | PITG\_00288 | 640 | Motif-0 | -512 | + | TCATTCTTTAATCGGA |
|  |  |  | Motif-2 | -577 | - | AGCTTCAACTT |
|  |  |  | Motif-4 | -553 | - | CCGTATTGGATGAAA |
| P. infestans | PITG\_00289 | 721 | Motif-0 | -242 | - | CACTCCTGCATGTGCC |
|  |  |  | Motif-1 | -386 | - | CGTACCGGTACCAGCA |
|  |  |  | Motif-4 | -136 | - | GGTGATTGGATGAAA |
|  |  |  | Motif-9 | -380 | - | CCGTACCGGTA |
| P. infestans | PITG\_00290 | 330 | Motif-1 | -225 | + | AGTGTACATGAAGT |
|  |  |  | Motif-16 | -296 | + | GCGTCGCAGCACCAGC |
|  |  |  | Motif-3 | -143 | - | GACTTAACTTTTGTAT |
| P. infestans | PITG\_00291 | 294 | Motif-3 | -221 | + | TAGAAACATCCAATCA |
|  |  |  | Motif-4 | -218 | - | TCTGATTGGATGTTT |
| P. infestans | PITG\_00292 | 294 | Motif-3 | -89 | - | TAGAAACATCCAATCA |
|  |  |  | Motif-4 | -91 | + | TCTGATTGGATGTTT |
| P. infestans | PITG\_00293 | 349 | Motif-0 | -125 | - | GCAAAATGCGCAATGC |
|  |  |  | Motif-2 | -15 | + | GAAGTTAAAGTGAA |
|  |  |  | Motif-3 | -30 | + | ATACAAAGTCGAAGAG |
| P. infestans | PITG\_00294 | 259 | Motif-0 | -66 | + | TCATTGACGAATCGTC |
|  |  |  | Motif-8 | -108 | + | TATTTGTAAATTAAA |
| P. infestans | PITG\_00295 | 259 | Motif-0 | -209 | - | TCATTGACGAATCGTC |
|  |  |  | Motif-8 | -106 | + | TTTTTTTAATTTTTA |
| P. infestans | PITG\_00296 | 544 | Motif-1 | -149 | - | AGTACAGGTAC |
|  |  |  | Motif-2 | -59 | + | ACTTCAACATG |
|  |  |  | Motif-3 | -409 | - | AATTCAAAGCTCAAGT |
| P. infestans | PITG\_00297 | 544 | Motif-1 | -405 | - | GTTATGTACCTGTAC |
|  |  |  | Motif-2 | -495 | - | CACTTCAACAT |
|  |  |  | Motif-3 | -150 | - | TACTTGAGCTTTGAAT |
| P. infestans | PITG\_00298 | 359 | Motif-2 | -111 | + | CTCGTTGAAGTG |
|  |  |  | Motif-4 | -329 | - | TGTCATTGGTCAATA |
| P. infestans | PITG\_00299 | 381 | Motif-1 | -192 | + | TACATGTAT |
|  |  |  | Motif-7 | -229 | - | TACTATTAATA |
| P. infestans | PITG\_00300 | 381 | Motif-1 | -78 | + | TACATGTAT |
|  |  |  | Motif-7 | -163 | + | TACTATTAATA |
| P. infestans | PITG\_00307 | 164 | Motif-0 | -139 | + | GTGAATTCGAAACTGG |
| P. infestans | PITG\_00308 | 243 | Motif-0 | -20 | + | CACTCCTCAATTTACG |
| P. infestans | PITG\_00309 | 243 | Motif-0 | -238 | + | GTAAATTGAGGAGTGA |
| P. infestans | PITG\_00310 | 296 | Motif-0 | -122 | - | GCACTGTCGTTTTTGC |
| P. infestans | PITG\_00311 | 289 | Motif-18 | -51 | - | TGGTGTGGTGCACGCA |
| P. infestans | PITG\_00312 | 289 | Motif-18 | -254 | + | TGGTGTGGTGCACGCA |
| P. infestans | PITG\_00313 | 195 | Motif-2 | -36 | + | ACTTCAACTTG |
| P. infestans | PITG\_00314 | 195 | Motif-2 | -169 | - | AACTTCAACTT |
| P. infestans | PITG\_00320 | 1000 | Motif-1 | -895 | + | TACATGTAT |
| P. infestans | PITG\_00321 | 570 | Motif-1 | -356 | - | TACATGAAC |
|  |  |  | Motif-4 | -548 | + | TTCCATTGGAAGAAA |
| P. infestans | PITG\_00322 | 1000 | Motif-0 | -57 | + | TCAGTTTGCGTTTTCA |
|  |  |  | Motif-3 | -595 | + | TACAAAATTCTAGTCA |
| P. infestans | PITG\_00323 | 372 | Motif-2 | -136 | - | CCATTTCAACTCCA |
| P. infestans | PITG\_00324 | 1000 | Motif-8 | -206 | + | TATTTTTTAATATCA |
| P. infestans | PITG\_00327 | 1000 | Motif-0 | -354 | + | GTGAATCGAAAAATGA |
| P. infestans | PITG\_00329 | 1000 | Motif-0 | -69 | - | ACAAAAAGGAAAATGA |
|  |  |  | Motif-1 | -235 | + | TACATGTAC |
|  |  |  | Motif-2 | -38 | - | GTCGAAGCTGAAGGTG |
| P. infestans | PITG\_00330 | 1000 | Motif-1 | -626 | + | TACATGTAC |
|  |  |  | Motif-4 | -220 | + | ATTCATTGGCTGAAA |
| P. infestans | PITG\_00332 | 951 | Motif-1 | -622 | + | TACATGTAC |
|  |  |  | Motif-2 | -672 | + | AATCACGCTGAAGTGG |
| P. infestans | PITG\_00333 | 951 | Motif-1 | -331 | + | TACATGCAC |
|  |  |  | Motif-2 | -295 | - | AATCACGCTGAAGTGG |
| P. infestans | PITG\_00334 | 356 | Motif-1 | -268 | + | TACATGTAT |
| P. infestans | PITG\_00336 | 758 | Motif-2 | -203 | + | CAAATTGAAGTT |
| P. infestans | PITG\_00337 | 758 | Motif-2 | -567 | - | CAAATTGAAGTT |
| P. infestans | PITG\_00338 | 1000 | Motif-1 | -75 | + | TCTACGTGTATAATT |
| P. infestans | PITG\_00340 | 1000 | Motif-0 | -341 | - | TTATTTCTGACTTTGC |
| P. infestans | PITG\_00343 | 1000 | Motif-0 | -511 | + | CCAGTTCCAAAGTGGC |
|  |  |  | Motif-1 | -382 | + | TACATGTAC |
|  |  |  | Motif-2 | -281 | + | GTTCAAGATGAAGCTG |
| P. infestans | PITG\_00347 | 1000 | Motif-3 | -728 | - | TCTTGGAATAAGTAT |
|  |  |  | Motif-7 | -620 | - | AACTATTAATA |
| P. infestans | PITG\_00348 | 1000 | Motif-0 | -116 | - | GCGGAACGCGAAGTGA |
|  |  |  | Motif-1 | -343 | + | TACATGTAC |
|  |  |  | Motif-16 | -908 | - | GAAAAGCAGCCGTAGC |
|  |  |  | Motif-2 | -625 | + | ACTTCAACTTG |
|  |  |  | Motif-3 | -187 | - | ACTTGCGTTCAGTAT |
|  |  |  | Motif-4 | -218 | - | TCTGATTGGCTACTA |
| P. infestans | PITG\_00349 | 147 | Motif-4 | -67 | + | TTTAATTGGCTGATT |
| P. infestans | PITG\_00350 | 147 | Motif-4 | -95 | - | TTTAATTGGCTGATT |
| P. infestans | PITG\_00352 | 357 | Motif-3 | -54 | + | ACTTAATATTTGTAT |
| P. infestans | PITG\_00353 | 357 | Motif-3 | -317 | + | TACAAATATTAAGTCA |
| P. infestans | PITG\_00354 | 446 | Motif-4 | -247 | + | CTTGATTGGCCGAAA |
|  |  |  | Motif-9 | -344 | - | GTGTACCGGTA |
| P. infestans | PITG\_00358 | 1000 | Motif-1 | -330 | - | TGAGCATGTACAAGTG |
| P. infestans | PITG\_00359 | 1000 | Motif-2 | -558 | + | GGACATCAACATCG |
|  |  |  | Motif-7 | -514 | - | TATTATTAATA |
|  |  |  | Motif-8 | -427 | - | TATTTCTAAATAGTA |
| P. infestans | PITG\_00360 | 1000 | Motif-0 | -98 | - | GAAAATCGAAGAACGA |
|  |  |  | Motif-2 | -715 | - | GGACATCAACATCG |
|  |  |  | Motif-7 | -756 | + | TATTATTAATA |
|  |  |  | Motif-8 | -847 | + | TATTTCTAAATAGTA |
| P. infestans | PITG\_00361 | 60 | Motif-4 | -39 | + | TTCTATTGGTTGAAA |
| P. infestans | PITG\_00362 | 178 | Motif-0 | -35 | - | GTAAACTGGGAAATGA |
| P. infestans | PITG\_00363 | 178 | Motif-0 | -159 | + | GTAAACTGGGAAATGA |
| P. infestans | PITG\_00366 | 1000 | Motif-0 | -41 | + | CATTTCTCAATTCACC |
|  |  |  | Motif-1 | -97 | + | TACATGTAT |
| P. infestans | PITG\_00368 | 1000 | Motif-0 | -331 | - | TCACTTCCCAGTTGG |
|  |  |  | Motif-6 | -603 | - | ACCCCCCCCCACCCCA |
|  |  |  | Motif-8 | -557 | + | TTTTATTAAGCTATA |
| P. infestans | PITG\_00369 | 1000 | Motif-4 | -517 | - | CTTTATTGGTTATTT |
|  |  |  | Motif-7 | -309 | - | CAGTATTAATA |
|  |  |  | Motif-8 | -317 | + | TAATTTTATATTAAT |
| P. infestans | PITG\_00375 | 1000 | Motif-0 | -57 | + | CCAGTTTGCTATTTTC |
|  |  |  | Motif-1 | -306 | - | AACATGTATTACGCT |
|  |  |  | Motif-3 | -166 | + | GCTTGGTTTAAGTAT |
|  |  |  | Motif-9 | -326 | + | CGGTACCGGTA |
| P. infestans | PITG\_00377 | 459 | Motif-3 | -116 | + | TACAAAAAGCAAGTTA |
| P. infestans | PITG\_00378 | 459 | Motif-3 | -357 | + | ACTTGCTTTTTGTAT |
| P. infestans | PITG\_00380 | 626 | Motif-0 | -60 | - | GATAATTGAGAAATGA |
|  |  |  | Motif-1 | -79 | + | TACATGCAC |
|  |  |  | Motif-2 | -561 | + | ACTTCAACTTTCTCGA |
| P. infestans | PITG\_00381 | 626 | Motif-0 | -581 | - | TCATTTCTCAATTAT |
|  |  |  | Motif-1 | -555 | + | TGCATGTAC |
|  |  |  | Motif-2 | -75 | - | AACTTCAACTT |
| P. infestans | PITG\_00388 | 334 | Motif-4 | -52 | - | TGTGATTGGCTGAAA |
| P. infestans | PITG\_00389 | 334 | Motif-4 | -277 | - | TCTGATTGGTAGAAA |
| P. infestans | PITG\_00390 | 1000 | Motif-0 | -236 | + | GTAAATTTCGAAATGA |
| P. infestans | PITG\_00391 | 1000 | Motif-0 | -679 | + | TCATTTCCGATTCCGT |
|  |  |  | Motif-2 | -198 | + | TCTACTTCAATTTGGA |
| P. infestans | PITG\_00392 | 517 | Motif-0 | -186 | + | GAAGTTTGCAGAATGA |
|  |  |  | Motif-2 | -192 | + | GTGGTTGAAGTT |
| P. infestans | PITG\_00393 | 311 | Motif-8 | -146 | - | TTATTTTTATTTATT |
| P. infestans | PITG\_00394 | 1000 | Motif-1 | -861 | + | TACATGTAC |
|  |  |  | Motif-3 | -481 | - | TTGCAAATTGCAAGTT |
| P. infestans | PITG\_00395 | 588 | Motif-0 | -204 | - | GCGGATTCGAGAATGA |
|  |  |  | Motif-3 | -326 | - | CACTTGGTGCTTGTAT |
| P. infestans | PITG\_00396 | 588 | Motif-0 | -400 | + | GCGGATTCGAGAATGA |
|  |  |  | Motif-3 | -277 | + | ACTTGGTGCTTGTAT |
| P. infestans | PITG\_00397 | 278 | Motif-17 | -202 | - | AGTTTAGCTATCTTT |
| P. infestans | PITG\_00398 | 929 | Motif-1 | -62 | - | TGCATGTAC |
|  |  |  | Motif-8 | -441 | + | TAATTTTTAATAATA |
| P. infestans | PITG\_00399 | 929 | Motif-1 | -105 | - | TACGTGTAC |
|  |  |  | Motif-8 | -503 | - | TAATTTTTAATAATA |
| P. infestans | PITG\_00400 | 1000 | Motif-0 | -124 | - | GCAAGTCTGGTAATGA |
|  |  |  | Motif-1 | -593 | + | TACATGTAT |
| P. infestans | PITG\_00401 | 1000 | Motif-1 | -868 | + | TACATGTAC |
|  |  |  | Motif-2 | -636 | + | TCGCGACTTGCAGTC |
|  |  |  | Motif-4 | -39 | - | TCTTATGGGTTAAAA |
|  |  |  | Motif-9 | -50 | + | CCGTACCGGTA |
| P. infestans | PITG\_00405 | 1000 | Motif-0 | -691 | - | CTACTTTTCAATTTG |
|  |  |  | Motif-2 | -487 | + | ACTGCAACTTG |
| P. infestans | PITG\_00406 | 1000 | Motif-0 | -853 | + | CTACTTTTCAATTTGT |
| P. infestans | PITG\_00407 | 308 | Motif-4 | -122 | + | TACGATTGGTTGAAA |
| P. infestans | PITG\_00411 | 274 | Motif-1 | -235 | - | CGTGGATGTATCC |
| P. infestans | PITG\_00412 | 1000 | Motif-1 | -152 | + | CGTACCGGTACAGTA |
|  |  |  | Motif-9 | -150 | - | CTGTACCGGTA |
| P. infestans | PITG\_00413 | 1000 | Motif-0 | -384 | + | GCGATTTGGCGAGTGA |
|  |  |  | Motif-1 | -853 | + | TACATGTAG |
|  |  |  | Motif-3 | -292 | + | TACATACAGTAAGTCA |
|  |  |  | Motif-4 | -373 | + | AGTGATTGGTTGAAA |
|  |  |  | Motif-8 | -408 | - | TATTTATAATTTTCA |
| P. infestans | PITG\_00416 | 1000 | Motif-0 | -92 | + | CATTCCGCAATTCCCT |
|  |  |  | Motif-1 | -219 | + | TACATGTAA |
|  |  |  | Motif-2 | -454 | + | CACTTTGAAGTT |
| P. infestans | PITG\_00417 | 392 | Motif-0 | -89 | - | GCACTCTGCCACTTGC |
| P. infestans | PITG\_00418 | 392 | Motif-0 | -115 | + | ACATTTGTCAATTTTC |
| P. infestans | PITG\_00419 | 309 | Motif-18 | -227 | - | TCATGTGGCGCACATA |
| P. infestans | PITG\_00420 | 309 | Motif-18 | -98 | + | TCATGTGGCGCACATA |
| P. infestans | PITG\_00421 | 1000 | Motif-7 | -351 | - | TACTATTAGTA |
| P. infestans | PITG\_00422 | 1000 | Motif-4 | -427 | + | TTCTATTGGTTTAAA |
| P. infestans | PITG\_00423 | 1000 | Motif-1 | -352 | + | ACAATACGAGTAGT |
|  |  |  | Motif-4 | -446 | + | TCCCATTAGCTAAAA |
|  |  |  | Motif-8 | -171 | - | TATTTATATATTTAA |
| P. infestans | PITG\_00426 | 1000 | Motif-2 | -415 | + | CACCTCAACACGCA |
|  |  |  | Motif-4 | -527 | - | TCTCATTGGTGAATA |
| P. infestans | PITG\_00427 | 1000 | Motif-1 | -270 | + | AACATGTATAGTTCC |
|  |  |  | Motif-8 | -113 | - | TTTTTTTTTGTTAAA |
| P. infestans | PITG\_00430 | 1000 | Motif-1 | -696 | + | TGCATGTAC |
|  |  |  | Motif-6 | -484 | - | ACACGCCCCCCCCCAC |
|  |  |  | Motif-8 | -197 | + | TTTTTTTAAATTACA |
| P. infestans | PITG\_00431 | 517 | Motif-0 | -115 | - | GCAAAACTGGAAATGG |
| P. infestans | PITG\_00432 | 645 | Motif-0 | -615 | + | GCAAAAGGTGGAATGA |
|  |  |  | Motif-4 | -449 | - | CTTGATTGGTAAAAA |
|  |  |  | Motif-7 | -216 | - | TATTATTAATA |
|  |  |  | Motif-8 | -220 | + | TTATTATTAATAATA |
| P. infestans | PITG\_00433 | 645 | Motif-0 | -46 | - | GCAAAAGGTGGAATGA |
|  |  |  | Motif-4 | -211 | + | CTTGATTGGTAAAAA |
|  |  |  | Motif-7 | -437 | - | TATTATTAATA |
|  |  |  | Motif-8 | -440 | - | TTATTATTAATAATA |
| P. infestans | PITG\_00436 | 1000 | Motif-0 | -62 | + | CACTTCAGCTTTTGCC |
|  |  |  | Motif-1 | -304 | + | TACATGTAC |
|  |  |  | Motif-3 | -609 | - | CAGTTGACTTTCGTAT |
| P. infestans | PITG\_00437 | 1000 | Motif-1 | -232 | + | TACATGTAG |
|  |  |  | Motif-2 | -595 | - | TCGCTTCAAGTTCA |
|  |  |  | Motif-4 | -93 | - | TGTGATTGGTGGATT |
|  |  |  | Motif-7 | -257 | + | TATTATTAATA |
| P. infestans | PITG\_00438 | 1000 | Motif-0 | -75 | + | CACTCTTGGATTTGCC |
|  |  |  | Motif-1 | -359 | + | TACATGTAC |
|  |  |  | Motif-8 | -384 | + | TTATATTAAATTACA |
|  |  |  | Motif-9 | -356 | + | ATGTACCGGTA |
| P. infestans | PITG\_00439 | 1000 | Motif-0 | -930 | + | GTACATGGGAAAATGA |
|  |  |  | Motif-1 | -475 | + | TACATGTAA |
| P. infestans | PITG\_00440 | 407 | Motif-2 | -138 | + | CACGTTGAAGTA |
| P. infestans | PITG\_00441 | 407 | Motif-2 | -280 | + | ACTTCAACGTG |
| P. infestans | PITG\_00444 | 1000 | Motif-0 | -383 | + | TCACTACACATTTGCC |
|  |  |  | Motif-2 | -951 | + | ACAACTTCAATTCATA |
| P. infestans | PITG\_00445 | 231 | Motif-2 | -218 | - | TCACTTCAACTGTA |
| P. infestans | PITG\_00446 | 231 | Motif-2 | -27 | + | TCACTTCAACTGTA |
| P. infestans | PITG\_00449 | 864 | Motif-1 | -203 | - | GTACTGTACATTTAC |
|  |  |  | Motif-9 | -208 | - | ATTTACCGGTA |
| P. infestans | PITG\_00450 | 864 | Motif-1 | -320 | + | TGCATGTAC |
|  |  |  | Motif-9 | -667 | + | ATTTACCGGTA |
| P. infestans | PITG\_00451 | 525 | Motif-0 | -183 | + | TCATTTTTGTATTCGG |
|  |  |  | Motif-1 | -436 | + | GGAGCATGTACAGCTC |
|  |  |  | Motif-2 | -405 | + | GCTTCAACGTG |
|  |  |  | Motif-3 | -187 | - | ATACAAAAATGAAAAG |
| P. infestans | PITG\_00452 | 525 | Motif-0 | -39 | + | CATTTTGCCATTTCTC |
|  |  |  | Motif-1 | -105 | - | GGAGCATGTACAGCTC |
|  |  |  | Motif-2 | -130 | - | CGCTTCAACGT |
|  |  |  | Motif-3 | -354 | + | ATACAAAAATGAAAAG |
| P. infestans | PITG\_00453 | 399 | Motif-0 | -17 | + | TCACTTTCAGTCTGTC |
|  |  |  | Motif-8 | -206 | - | TAATTTTTAATAGTA |
| P. infestans | PITG\_00454 | 1000 | Motif-0 | -47 | - | TCAAGACGAAAAGTGA |
|  |  |  | Motif-1 | -269 | + | TACATGCAC |
|  |  |  | Motif-2 | -701 | + | GATATTGAAGCG |
| P. infestans | PITG\_00455 | 1000 | Motif-0 | -974 | - | TTCACTTTTCGTCTT |
|  |  |  | Motif-1 | -719 | - | TACATGCAC |
|  |  |  | Motif-2 | -318 | - | GATATTGAAGCG |
| P. infestans | PITG\_00458 | 1000 | Motif-0 | -37 | + | CCATTTTGCGACTCAC |
|  |  |  | Motif-4 | -116 | - | GCTGATTGGTAGATA |
| P. infestans | PITG\_00459 | 200 | Motif-4 | -70 | - | TCTGATTGGGTAAAT |
| P. infestans | PITG\_00461 | 1000 | Motif-0 | -53 | - | GTAAATCGAGATGTGA |
|  |  |  | Motif-2 | -355 | + | CAACTTCAACAAA |
| P. infestans | PITG\_00464 | 131 | Motif-9 | -74 | + | GTGTACCGGTA |
| P. infestans | PITG\_00465 | 131 | Motif-9 | -68 | - | GTGTACCGGTA |
| P. infestans | PITG\_00466 | 1000 | Motif-0 | -670 | + | CCATTCCGCCTTTTTC |
|  |  |  | Motif-1 | -436 | + | TACATGTAC |
|  |  |  | Motif-3 | -109 | - | ATCTTGGATTTTGTAA |
|  |  |  | Motif-8 | -225 | + | TATTTTTAAATAACA |
|  |  |  | Motif-9 | -442 | - | ATGTACCGGTA |
| P. infestans | PITG\_00467 | 1000 | Motif-0 | -67 | + | GCACTTTGTATTTGAC |
| P. infestans | PITG\_00469 | 1000 | Motif-0 | -28 | + | GCACTTCACGATTCGC |
|  |  |  | Motif-2 | -524 | - | CACTTCAACAT |
| P. infestans | PITG\_00471 | 401 | Motif-0 | -50 | + | TCATTCCCGGTTTAAC |
|  |  |  | Motif-4 | -185 | - | GATGATTGGCCAAAA |
| P. infestans | PITG\_00472 | 1000 | Motif-8 | -115 | - | TTTTTCTAAATTAAA |
| P. infestans | PITG\_00473 | 1000 | Motif-0 | -19 | + | TCACTTTCAAATCGAA |
| P. infestans | PITG\_00474 | 284 | Motif-3 | -105 | + | TACAAACTTCAAGTCA |
|  |  |  | Motif-4 | -172 | - | AGTGATTGGATGAAA |
| P. infestans | PITG\_00476 | 1000 | Motif-4 | -92 | - | TATGATTGGCCAAAA |
| P. infestans | PITG\_00477 | 1000 | Motif-4 | -71 | + | TGTGATTGGTAAAAA |
| P. infestans | PITG\_00478 | 391 | Motif-16 | -43 | - | CAGCATCAGCACTAGC |
| P. infestans | PITG\_00479 | 1000 | Motif-0 | -665 | - | GCAAATTCTCTAATGA |
| P. infestans | PITG\_00480 | 1000 | Motif-1 | -368 | + | TGTAGATGTAACT |
|  |  |  | Motif-2 | -172 | - | GACTTCAACAT |
|  |  |  | Motif-4 | -676 | - | GATCATTGGATAAAA |
| P. infestans | PITG\_00483 | 849 | Motif-0 | -466 | + | GCAATACGGAGACTGA |
|  |  |  | Motif-2 | -155 | - | AAAGTTGAAGCG |
| P. infestans | PITG\_00485 | 537 | Motif-3 | -166 | + | TACTAAATCTAAGTCA |
| P. infestans | PITG\_00486 | 537 | Motif-3 | -385 | + | ACTTAGATTTAGTAA |
| P. infestans | PITG\_00488 | 1000 | Motif-0 | -67 | + | TCATTCTCCATTTACT |
|  |  |  | Motif-1 | -413 | + | TACATGTAT |
|  |  |  | Motif-2 | -510 | - | ACTGCAACTCG |
|  |  |  | Motif-3 | -135 | + | ACTTGGATTTAGTAA |
|  |  |  | Motif-8 | -591 | + | TTTTATTTAGCAAAT |
| P. infestans | PITG\_00489 | 1000 | Motif-1 | -925 | + | TACATGTAC |
|  |  |  | Motif-3 | -18 | + | TAAAAACACCGAGTCA |
| P. infestans | PITG\_00490 | 1000 | Motif-1 | -358 | + | TACATGTAC |
|  |  |  | Motif-2 | -87 | + | ACTTCAACGTAGCAAC |
|  |  |  | Motif-4 | -64 | - | GATCATTGGTCAAAA |
| P. infestans | PITG\_00491 | 1000 | Motif-0 | -392 | + | CCATTTTTCTTCTTCC |
|  |  |  | Motif-2 | -499 | + | ACTTCAACCTGGGTCT |
| P. infestans | PITG\_00492 | 1000 | Motif-0 | -673 | - | TCACTTCCATTTTTCT |
|  |  |  | Motif-2 | -566 | - | CACTTCAACCT |
| P. infestans | PITG\_00494 | 145 | Motif-4 | -76 | + | TTTGATTGGTGAAAA |
| P. infestans | PITG\_00495 | 145 | Motif-4 | -54 | - | TTGGATTGGTTGAAA |
| P. infestans | PITG\_00496 | 1000 | Motif-2 | -42 | + | ACTTCAACATC |
|  |  |  | Motif-3 | -748 | + | TTTTGATTATTGTAT |
|  |  |  | Motif-4 | -632 | + | GGTGATTGGCTAATA |
| P. infestans | PITG\_00499 | 1000 | Motif-1 | -623 | + | TACTGATACACGTAC |
| P. infestans | PITG\_00501 | 125 | Motif-0 | -32 | - | TTAAAATGTAGAATGA |
| P. infestans | PITG\_00502 | 223 | Motif-18 | -165 | - | TGGTGTGATGTACATT |
| P. infestans | PITG\_00503 | 223 | Motif-18 | -74 | + | TGGTGTGATGTACATT |
| P. infestans | PITG\_00505 | 1000 | Motif-0 | -64 | + | TCATTTTCCTTTCGGA |
|  |  |  | Motif-2 | -704 | - | CGCTTCAACGT |
|  |  |  | Motif-4 | -271 | - | TACAATTGGCCAAAA |
|  |  |  | Motif-6 | -89 | + | CCCCCTCGCTCCCCCT |
|  |  |  | Motif-7 | -957 | + | TACTACTAATA |
| P. infestans | PITG\_00506 | 1000 | Motif-0 | -83 | - | GCAAGAGCAAGAGTGA |
|  |  |  | Motif-1 | -222 | - | AGTAAATGTAT |
|  |  |  | Motif-2 | -634 | + | GCTTCAACGTG |
|  |  |  | Motif-7 | -376 | + | TAGTATTAATA |
| P. infestans | PITG\_00507 | 1000 | Motif-1 | -160 | + | ACAACATGTA |
|  |  |  | Motif-3 | -755 | + | TTCGACAGCCGAGTGA |
| P. infestans | PITG\_00508 | 1000 | Motif-17 | -55 | - | ACTGTAGCCATTTCT |
|  |  |  | Motif-2 | -781 | - | CGACTTCAAGTTCA |
|  |  |  | Motif-4 | -31 | - | GACGATTGGCTGAAA |
| P. infestans | PITG\_00513 | 1000 | Motif-0 | -113 | - | GCAAATTTGAGAATGA |
|  |  |  | Motif-1 | -352 | - | AGTACAGGTAC |
|  |  |  | Motif-3 | -709 | + | ACTTGTTTTATGTAA |
| P. infestans | PITG\_00514 | 1000 | Motif-0 | -68 | + | CCATTTCCGATTTTTA |
|  |  |  | Motif-1 | -816 | + | TACGTGTAC |
|  |  |  | Motif-2 | -740 | + | GCTGCAACTTG |
| P. infestans | PITG\_00515 | 1000 | Motif-1 | -149 | - | CATATATGTATC |
|  |  |  | Motif-2 | -681 | - | GCTGCAACTTG |
| P. infestans | PITG\_00516 | 143 | Motif-3 | -18 | + | ACTTATTATTTGTAT |
|  |  |  | Motif-4 | -56 | + | TTAGATTGGTAAAAA |
|  |  |  | Motif-7 | -68 | - | AATTATTAATA |
|  |  |  | Motif-8 | -35 | - | TTTTTTTAAATGAAA |
| P. infestans | PITG\_00517 | 143 | Motif-3 | -140 | - | GACTTATTATTTGTAT |
|  |  |  | Motif-4 | -102 | - | TTAGATTGGTAAAAA |
|  |  |  | Motif-7 | -86 | + | AATTATTAATA |
|  |  |  | Motif-8 | -123 | + | TTTTTTTAAATGAAA |
| P. infestans | PITG\_00518 | 1000 | Motif-0 | -107 | + | TCATTTCATAATATTC |
| P. infestans | PITG\_00523 | 806 | Motif-17 | -124 | + | TATGTAGCCATTTCT |
| P. infestans | PITG\_00524 | 695 | Motif-2 | -547 | - | AGCTTCAACTT |
|  |  |  | Motif-3 | -54 | - | CACTTGCGTTTAGTAA |
| P. infestans | PITG\_00525 | 118 | Motif-2 | -107 | - | AATGTTGAAGTT |
| P. infestans | PITG\_00527 | 1000 | Motif-0 | -44 | + | TCATTATCGTTTTCGC |
|  |  |  | Motif-1 | -795 | + | TACATGTAT |
|  |  |  | Motif-7 | -464 | + | AACTATTAATA |
| P. infestans | PITG\_00528 | 1000 | Motif-1 | -71 | - | GGTACATAAAC |
|  |  |  | Motif-17 | -96 | + | ACTTTAGCCATTTCG |
| P. infestans | PITG\_00529 | 338 | Motif-1 | -213 | + | TACATGTAG |
| P. infestans | PITG\_00530 | 338 | Motif-1 | -47 | + | TACATGTAT |
| P. infestans | PITG\_00531 | 1000 | Motif-0 | -339 | + | ATTTAATTTTCACAAT |
|  |  |  | Motif-4 | -444 | + | CCTGATTGGGTAGTA |
| P. infestans | PITG\_00535 | 573 | Motif-0 | -19 | + | CACTATTAAATTTGTC |
|  |  |  | Motif-1 | -165 | - | TACTTGTACT |
|  |  |  | Motif-2 | -120 | - | GACTTCAAATTGGT |
|  |  |  | Motif-8 | -495 | - | TACTTCTAATTTTAA |
| P. infestans | PITG\_00536 | 573 | Motif-0 | -33 | - | GCAGTGCTCAATCTAC |
|  |  |  | Motif-1 | -42 | + | ACACTACAAGTAGA |
|  |  |  | Motif-2 | -467 | + | GACTTCAAATTGGT |
|  |  |  | Motif-8 | -93 | + | TACTTCTAATTTTAA |
| P. infestans | PITG\_00537 | 530 | Motif-18 | -423 | - | TCGTGTGGTTTACATA |
|  |  |  | Motif-3 | -149 | - | TACTTGAGATTTGTAA |
| P. infestans | PITG\_00538 | 1000 | Motif-0 | -635 | - | CCATTGTTGCATCGGC |
|  |  |  | Motif-4 | -51 | + | TCGGATTGGCTGAAA |
| P. infestans | PITG\_00539 | 1000 | Motif-0 | -441 | + | CCATTGTTGCATCGGC |
| P. infestans | PITG\_00540 | 1000 | Motif-0 | -24 | - | GCCAGATCAAAACTGA |
|  |  |  | Motif-3 | -257 | - | ATTTGATTATAGTAT |
|  |  |  | Motif-4 | -70 | + | TCTGATTGGTTATTT |
|  |  |  | Motif-8 | -563 | + | TTTTTTTTAGCATAA |
| P. infestans | PITG\_00542 | 1000 | Motif-1 | -146 | - | TACGTGTAC |
| P. infestans | PITG\_00543 | 974 | Motif-0 | -921 | + | GCAAATAGCGGAATGG |
|  |  |  | Motif-1 | -526 | - | AACATGTAC |
|  |  |  | Motif-8 | -500 | + | TATTTTTTTTTTAAT |
| P. infestans | PITG\_00544 | 974 | Motif-0 | -69 | - | GCAAATAGCGGAATGG |
|  |  |  | Motif-1 | -457 | + | AACATGTAC |
|  |  |  | Motif-8 | -489 | - | TATTTTTTTTTTAAT |
| P. infestans | PITG\_00545 | 502 | Motif-0 | -47 | + | TCATTCCCACATCGAC |
|  |  |  | Motif-17 | -239 | + | AATGTAGCCATATCG |
|  |  |  | Motif-4 | -156 | - | TGGGATTGGGTGATA |
| P. infestans | PITG\_00546 | 365 | Motif-4 | -306 | + | GTGTATTGGTTGAAA |
| P. infestans | PITG\_00547 | 365 | Motif-4 | -74 | - | GTGTATTGGTTGAAA |
| P. infestans | PITG\_00551 | 1000 | Motif-0 | -373 | + | CACTTTGAAATGCACC |
|  |  |  | Motif-1 | -788 | - | AGTACCGGTACTTTA |
|  |  |  | Motif-2 | -856 | + | CAAGTGGAAGTG |
|  |  |  | Motif-9 | -783 | - | AAGTACCGGTA |
| P. infestans | PITG\_00552 | 1000 | Motif-0 | -685 | - | CACTTTGAAATGCACC |
|  |  |  | Motif-1 | -269 | + | AGTACCGGTACTTTA |
|  |  |  | Motif-2 | -162 | - | ATAGTTGAAGTTGA |
|  |  |  | Motif-9 | -267 | - | AAGTACCGGTA |
| P. infestans | PITG\_00553 | 284 | Motif-0 | -39 | + | TCATTCGGCAATCGGT |
|  |  |  | Motif-3 | -127 | + | TCTTGGTTTTTGTAT |
| P. infestans | PITG\_00556 | 259 | Motif-8 | -141 | - | TATTTTTAATTAGGA |
| P. infestans | PITG\_00557 | 259 | Motif-8 | -133 | + | TATTTTTAATTAGGA |
| P. infestans | PITG\_00558 | 124 | Motif-0 | -26 | - | GACAAGTTGAAAATGA |
|  |  |  | Motif-1 | -66 | + | TACATGTAC |
|  |  |  | Motif-4 | -79 | + | TTCGATTGGCTAATA |
| P. infestans | PITG\_00559 | 1000 | Motif-0 | -178 | - | GCACTCTGGATTCTAC |
|  |  |  | Motif-1 | -923 | + | TGCATGTAGAAGTAC |
|  |  |  | Motif-3 | -434 | + | AGCTTGGGTTTTGTAA |
|  |  |  | Motif-4 | -165 | - | GCCGATTGGCTAGCA |
|  |  |  | Motif-8 | -824 | - | TATATATAAATATAA |
| P. infestans | PITG\_00560 | 1000 | Motif-0 | -472 | + | CCATTCCCCTTTTCCA |
|  |  |  | Motif-1 | -685 | + | TACATGCAC |
|  |  |  | Motif-4 | -563 | + | TCTGATTGGCCAATC |
|  |  |  | Motif-8 | -791 | + | TATATATAAATATAA |
| P. infestans | PITG\_00561 | 639 | Motif-1 | -492 | + | TACATGTAC |
|  |  |  | Motif-2 | -209 | - | TGTGTTGAAGTTGA |
|  |  |  | Motif-9 | -151 | + | ATTTACCGGTA |
| P. infestans | PITG\_00562 | 639 | Motif-1 | -155 | + | TACATGTAC |
|  |  |  | Motif-2 | -437 | - | AAATTCAACTT |
|  |  |  | Motif-9 | -499 | - | ATTTACCGGTA |
| P. infestans | PITG\_00563 | 1000 | Motif-1 | -76 | - | AGTACAGGTAT |
|  |  |  | Motif-16 | -477 | - | CAGCAATAGCAGCAGC |
| P. infestans | PITG\_00564 | 1000 | Motif-0 | -90 | + | TCACAACGGAATTTGC |
|  |  |  | Motif-16 | -662 | + | CAATAGCAGCAGCAGT |
| P. infestans | PITG\_00566 | 1000 | Motif-0 | -866 | + | CATTTTACAATTTGCC |
|  |  |  | Motif-2 | -165 | + | GTAGTTGAAGTG |
|  |  |  | Motif-4 | -92 | - | TTTGATTGGATGAAG |
| P. infestans | PITG\_00570 | 263 | Motif-0 | -34 | + | TCACTTCTCAACCGCA |
|  |  |  | Motif-1 | -227 | + | TACATGAAC |
| P. infestans | PITG\_00571 | 544 | Motif-2 | -225 | - | AAAGTTGAAGTT |
|  |  |  | Motif-3 | -222 | + | TTCAACTTTCAACTCA |
|  |  |  | Motif-4 | -115 | - | TCTAATTGGCTACAA |
| P. infestans | PITG\_00572 | 1000 | Motif-0 | -362 | + | TCAATCTACAATTGGC |
| P. infestans | PITG\_00573 | 1000 | Motif-4 | -354 | - | TTTTATTGGATTAAA |
| P. infestans | PITG\_00574 | 236 | Motif-9 | -135 | + | CAGTACCGGTA |
| P. infestans | PITG\_00575 | 258 | Motif-1 | -81 | + | AACATGTAC |
| P. infestans | PITG\_00576 | 258 | Motif-1 | -186 | - | AACATGTAC |
| P. infestans | PITG\_00577 | 355 | Motif-0 | -19 | + | CACTCTTGCATTTGTG |
|  |  |  | Motif-2 | -59 | + | GACGTTGCAGTT |
|  |  |  | Motif-4 | -231 | - | TTTTATTCGCTAAAA |
| P. infestans | PITG\_00578 | 355 | Motif-0 | -352 | - | CACTCTTGCATTTGTG |
|  |  |  | Motif-2 | -93 | - | GATGTTGAAGCG |
|  |  |  | Motif-4 | -110 | + | GCTGATTGGACAGAT |
| P. infestans | PITG\_00579 | 1000 | Motif-0 | -49 | - | GCGAATGGAGGAGTAA |
|  |  |  | Motif-1 | -121 | - | TACATGTGTA |
| P. infestans | PITG\_00580 | 1000 | Motif-0 | -924 | + | CTATTTCTCAATTTG |
|  |  |  | Motif-4 | -785 | + | GGTGATTGGTCAAAA |
| P. infestans | PITG\_00582 | 1000 | Motif-0 | -71 | - | GAAAAAGGGAAAATGA |
|  |  |  | Motif-1 | -441 | + | TACTTGTACT |
|  |  |  | Motif-6 | -699 | + | CCCCCCTCCACCTCCC |
| P. infestans | PITG\_00584 | 403 | Motif-0 | -44 | - | GCAAATCGCCAACTGA |
| P. infestans | PITG\_00585 | 1000 | Motif-7 | -192 | - | TATTATTAATA |
| P. infestans | PITG\_00586 | 1000 | Motif-0 | -382 | + | CACTTTGAAATTTCTG |
| P. infestans | PITG\_00587 | 1000 | Motif-0 | -628 | - | TCATTTCGAAATAGGC |
|  |  |  | Motif-1 | -151 | + | TACATGTAG |
|  |  |  | Motif-18 | -231 | + | TTGTGTGGTCCACACT |
| P. infestans | PITG\_00588 | 155 | Motif-3 | -144 | - | ATGCAAAAAGCAATTG |
|  |  |  | Motif-4 | -71 | + | TGGGATTGGTCAAAA |
| P. infestans | PITG\_00589 | 155 | Motif-3 | -27 | + | ATGCAAAAAGCAATTG |
|  |  |  | Motif-4 | -99 | - | TGGGATTGGTCAAAA |
| P. infestans | PITG\_00590 | 355 | Motif-3 | -70 | + | TCTTGGCTTTTGTAA |
| P. infestans | PITG\_00591 | 1000 | Motif-1 | -893 | + | TACATGTAC |
|  |  |  | Motif-2 | -11 | + | ACTTCAACATC |
|  |  |  | Motif-4 | -88 | + | GCTCATTCGTCAAAA |
| P. infestans | PITG\_00592 | 1000 | Motif-1 | -316 | + | TACATGTAG |
| P. infestans | PITG\_00594 | 314 | Motif-0 | -25 | + | CATTCCCTAATTTGCT |
|  |  |  | Motif-4 | -86 | + | CGTGATTGGTCAAAA |
| P. infestans | PITG\_00595 | 314 | Motif-0 | -304 | + | GCAAATTAGGGAATGG |
|  |  |  | Motif-4 | -243 | - | CGTGATTGGTCAAAA |
| P. infestans | PITG\_00596 | 115 | Motif-6 | -42 | + | CCCCCCCCCCCCTCAC |
| P. infestans | PITG\_00597 | 730 | Motif-1 | -125 | + | TACGTGTAC |
|  |  |  | Motif-2 | -164 | + | CACCTTCAACATA |
|  |  |  | Motif-4 | -597 | - | TGTGATAGGCTAAAA |
| P. infestans | PITG\_00598 | 730 | Motif-1 | -47 | - | CAAACATTCATGTAC |
|  |  |  | Motif-2 | -561 | + | GGTGTTGAAGTT |
|  |  |  | Motif-4 | -148 | + | TGTGATAGGCTAAAA |
| P. infestans | PITG\_00601 | 1000 | Motif-0 | -25 | + | TCATTCGAGAACTTTC |
|  |  |  | Motif-1 | -111 | + | TACATGTAA |
|  |  |  | Motif-4 | -73 | + | TTGGATTGGTTAAAA |
| P. infestans | PITG\_00607 | 1000 | Motif-1 | -38 | + | TACATGTAC |
|  |  |  | Motif-4 | -791 | + | TGTGATTAGCCTAAA |
|  |  |  | Motif-8 | -204 | + | TTCTATTAAATTAAT |
| P. infestans | PITG\_00611 | 1000 | Motif-0 | -25 | + | TCAGTCAAGAATTTTC |
|  |  |  | Motif-1 | -257 | + | TACATGTAT |
|  |  |  | Motif-2 | -335 | + | TAACTTCAACAAA |
|  |  |  | Motif-4 | -74 | + | TTGGATTGGCTAGAA |
| P. infestans | PITG\_00613 | 1000 | Motif-17 | -185 | - | GCTGTAGCCATCTTT |
| P. infestans | PITG\_00615 | 1000 | Motif-1 | -275 | + | ACCACATGTACTACT |
|  |  |  | Motif-4 | -935 | + | TGCGATTGGACAGTA |
|  |  |  | Motif-8 | -162 | + | TATTTATTAATAAAA |
| P. infestans | PITG\_00616 | 1000 | Motif-1 | -190 | - | TACGTGTAC |
|  |  |  | Motif-4 | -951 | - | TGCGATTGGACAGTA |
| P. infestans | PITG\_00617 | 1000 | Motif-1 | -361 | - | TGCATGTAC |
|  |  |  | Motif-7 | -277 | - | AAGTATTAATA |
|  |  |  | Motif-8 | -767 | - | TTTTACTTAACAAAA |
| P. infestans | PITG\_00618 | 347 | Motif-0 | -308 | + | GCGAAAAGCGAAGTGA |
|  |  |  | Motif-9 | -172 | - | CAGTACCGGTA |
| P. infestans | PITG\_00619 | 347 | Motif-0 | -55 | - | GCGAAAAGCGAAGTGA |
|  |  |  | Motif-9 | -186 | + | CAGTACCGGTA |
| P. infestans | PITG\_00620 | 1000 | Motif-0 | -227 | + | ATTTAATTATTAATTT |
|  |  |  | Motif-6 | -673 | + | GCCCCCCCCAACCCAC |
| P. infestans | PITG\_00624 | 1000 | Motif-2 | -182 | - | GAGATTGAAGTG |
| P. infestans | PITG\_00625 | 1000 | Motif-1 | -293 | + | TACATGTAG |
|  |  |  | Motif-2 | -91 | - | ACTGCAACTCG |
| P. infestans | PITG\_00626 | 884 | Motif-1 | -380 | + | TACATGTAT |
|  |  |  | Motif-4 | -280 | - | GAGGATTGGATAAAA |
| P. infestans | PITG\_00629 | 1000 | Motif-0 | -992 | + | TCATTTTTAATCTCT |
| P. infestans | PITG\_00630 | 720 | Motif-17 | -420 | + | GCTGTAGCCATTTCA |
| P. infestans | PITG\_00631 | 1000 | Motif-1 | -514 | + | TACATGTAG |
|  |  |  | Motif-2 | -969 | + | GATGTTGAAGTA |
|  |  |  | Motif-3 | -986 | - | ATCTTGATATTTGTAG |
|  |  |  | Motif-4 | -421 | + | GTTCATTCGTCAAAA |
| P. infestans | PITG\_00632 | 1000 | Motif-1 | -659 | + | TACATGTAA |
|  |  |  | Motif-2 | -207 | + | ACTTCAACATC |
|  |  |  | Motif-3 | -194 | + | TCTTGATATTTGTAG |
|  |  |  | Motif-4 | -759 | - | GTTCATTCGTCAAAA |
|  |  |  | Motif-7 | -43 | + | TACTATTAATA |
| P. infestans | PITG\_00633 | 154 | Motif-4 | -88 | - | TCGGATTGGTAAAAA |
| P. infestans | PITG\_00634 | 154 | Motif-4 | -81 | + | TCGGATTGGTAAAAA |
| P. infestans | PITG\_00635 | 740 | Motif-0 | -285 | - | GCGAGTCCGCAAATGA |
| P. infestans | PITG\_00636 | 204 | Motif-2 | -125 | + | GCTTCAACTCG |
| P. infestans | PITG\_00637 | 1000 | Motif-0 | -567 | + | CACTTTTAATTTTGTT |
|  |  |  | Motif-3 | -681 | + | TACAAAAATTAAGTGA |
| P. infestans | PITG\_00643 | 782 | Motif-2 | -483 | + | GCACTTCAACTGCA |
| P. infestans | PITG\_00644 | 1000 | Motif-0 | -186 | + | TTCACTTTTTTTTTT |
|  |  |  | Motif-1 | -383 | - | TACATGTGCG |
|  |  |  | Motif-8 | -181 | + | TTTTTTTTTTCAAAA |
| P. infestans | PITG\_00645 | 1000 | Motif-2 | -317 | - | CCACTTCACCATG |
|  |  |  | Motif-7 | -549 | - | TATTATTAATA |
| P. infestans | PITG\_00646 | 1000 | Motif-1 | -687 | + | TACATGTAT |
|  |  |  | Motif-4 | -240 | + | CTTGATTGCTTGAAA |
| P. infestans | PITG\_00647 | 1000 | Motif-0 | -125 | + | TCATTTTCAAATCCTT |
| P. infestans | PITG\_00648 | 1000 | Motif-2 | -275 | + | CATGTTGCAGTT |
|  |  |  | Motif-7 | -459 | - | AAGTATTAATA |
|  |  |  | Motif-8 | -456 | - | TTTTTTTAAGTATTA |
| P. infestans | PITG\_00651 | 1000 | Motif-0 | -344 | - | TTATTTCTGACTTTGC |
| P. infestans | PITG\_00652 | 488 | Motif-2 | -172 | + | CTTGTTGAAGTT |
|  |  |  | Motif-4 | -116 | - | ATTGATTGGCCAAAA |
| P. infestans | PITG\_00653 | 446 | Motif-0 | -62 | + | TCATTTCTCGACTTCG |
|  |  |  | Motif-3 | -376 | - | GTCTTGGCGTTCGTAT |
| P. infestans | PITG\_00654 | 446 | Motif-0 | -399 | - | TCATTTCTCGACTTC |
|  |  |  | Motif-3 | -86 | + | GTCTTGGCGTTCGTAT |
| P. infestans | PITG\_00655 | 96 | Motif-4 | -57 | - | TGTGATTCGGCAAAA |
| P. infestans | PITG\_00656 | 681 | Motif-0 | -140 | + | CAAAATTCAAAAATGA |
|  |  |  | Motif-1 | -119 | + | AATACATAAAC |
| P. infestans | PITG\_00658 | 377 | Motif-0 | -37 | + | TCACTCCGCATTCAGA |
|  |  |  | Motif-2 | -86 | + | CAGCTTCAACAAA |
|  |  |  | Motif-4 | -64 | - | TATGGTTGGATGAAA |
| P. infestans | PITG\_00659 | 1000 | Motif-3 | -613 | + | ACTTGACTTCAGTAT |
|  |  |  | Motif-4 | -686 | + | TGTGATTGGTTAAAA |
|  |  |  | Motif-8 | -208 | - | TTTATATAATTTATA |
| P. infestans | PITG\_00660 | 1000 | Motif-2 | -286 | - | GCGGTTGAAGTGGC |
|  |  |  | Motif-3 | -796 | - | ACTTGACTTCAGTAT |
|  |  |  | Motif-4 | -723 | - | TGTGATTGGTTAAAA |
| P. infestans | PITG\_00663 | 251 | Motif-0 | -45 | + | TCATTCGCGAATTGGT |
|  |  |  | Motif-17 | -233 | - | ATTGTAGCCATTTTT |
|  |  |  | Motif-2 | -247 | + | GCTTCAACTTG |
|  |  |  | Motif-3 | -110 | + | ACTTGGACTCAGTAT |
| P. infestans | PITG\_00666 | 278 | Motif-2 | -181 | + | CACCTTGAAGTG |
| P. infestans | PITG\_00668 | 1000 | Motif-0 | -62 | - | GCCGATTCAAAAGTGA |
|  |  |  | Motif-2 | -351 | - | TGGCTTCAAGGTGG |
| P. infestans | PITG\_00669 | 1000 | Motif-0 | -87 | - | GGAAAAGCGATAATGA |
|  |  |  | Motif-1 | -476 | + | TACATGTAC |
| P. infestans | PITG\_00670 | 738 | Motif-18 | -35 | - | TGATGTGTTGTACACA |
| P. infestans | PITG\_00671 | 1000 | Motif-8 | -435 | - | TTTTTTTTTATTTTA |
| P. infestans | PITG\_00672 | 1000 | Motif-8 | -690 | + | TTTTTTTATTTTAAA |
| P. infestans | PITG\_00673 | 1000 | Motif-7 | -354 | + | AACTATTAATA |
| P. infestans | PITG\_00675 | 1000 | Motif-2 | -49 | - | CGCTTCAACTT |
|  |  |  | Motif-4 | -77 | - | TCCGATTGGTCAGAT |
| P. infestans | PITG\_00679 | 1000 | Motif-4 | -326 | - | TCTAATTGACTAAAA |
|  |  |  | Motif-9 | -205 | - | ATTTACCGGTA |
| P. infestans | PITG\_00682 | 842 | Motif-0 | -56 | + | CACTTTGAACTTCGCC |
|  |  |  | Motif-18 | -204 | - | TTGCGTGGTGCACACA |
|  |  |  | Motif-2 | -776 | - | TTCGTTGAAGTG |
|  |  |  | Motif-4 | -180 | + | GACGATTGGATTAAA |
| P. infestans | PITG\_00683 | 842 | Motif-0 | -720 | + | TCATTGCCCATTTTCT |
|  |  |  | Motif-18 | -654 | + | TTGCGTGGTGCACACA |
|  |  |  | Motif-2 | -78 | + | TTCGTTGAAGTG |
|  |  |  | Motif-4 | -55 | + | TTCTATTGGTCAAAA |
| P. infestans | PITG\_00685 | 1000 | Motif-1 | -388 | + | TACACGTATAAATGT |
|  |  |  | Motif-2 | -261 | - | ACTTCAACGTAGTACT |
|  |  |  | Motif-7 | -49 | - | TAGTATTAATA |
| P. infestans | PITG\_00686 | 1000 | Motif-1 | -38 | - | TACACGTAGCAATAG |
|  |  |  | Motif-2 | -15 | - | GACTTCAACTT |
|  |  |  | Motif-7 | -145 | + | TACTATTAATA |
|  |  |  | Motif-8 | -453 | + | TTTTATTTAACTATT |
| P. infestans | PITG\_00688 | 414 | Motif-3 | -199 | + | TACTGAGACCAAGTCA |
| P. infestans | PITG\_00689 | 1000 | Motif-0 | -44 | + | CACTTGCCAAGTTGCC |
| P. infestans | PITG\_00691 | 1000 | Motif-1 | -933 | + | TACATGTTTCGTGAC |
|  |  |  | Motif-17 | -770 | + | AATGTAGCCATATTA |
|  |  |  | Motif-3 | -141 | - | ACCACAAAGCCCTAGT |
|  |  |  | Motif-7 | -281 | + | CACTATTAATA |
| P. infestans | PITG\_00693 | 973 | Motif-0 | -49 | - | GGGAAACGAGAAGTGA |
|  |  |  | Motif-1 | -238 | + | TGCATGTAC |
| P. infestans | PITG\_00694 | 376 | Motif-0 | -197 | + | TCCGTCTTTAACTGGC |
|  |  |  | Motif-2 | -282 | - | CACTGCAACTCCGA |
|  |  |  | Motif-9 | -302 | + | GAGTACCGGTA |
| P. infestans | PITG\_00695 | 683 | Motif-0 | -16 | - | CCTCTCTCGAATTCGC |
| P. infestans | PITG\_00697 | 1000 | Motif-0 | -59 | + | TCACTTCCAGATCCGA |
|  |  |  | Motif-2 | -799 | - | GCCGAAGCTGAAGTGA |
|  |  |  | Motif-4 | -693 | - | TTGAAACCAATTAGA |
| P. infestans | PITG\_00698 | 1000 | Motif-2 | -354 | + | GCCGAAGCTGAAGTGA |
|  |  |  | Motif-4 | -459 | + | TTGAAACCAATTAGA |
| P. infestans | PITG\_00699 | 378 | Motif-0 | -319 | - | TCACTTTTCATTGTCT |
| P. infestans | PITG\_00702 | 299 | Motif-0 | -162 | + | CATTCTGCAATTGGTC |
|  |  |  | Motif-4 | -91 | - | GACGATTGGTTGAAT |
| P. infestans | PITG\_00703 | 299 | Motif-0 | -152 | - | GCATTCTGCAATTGGT |
|  |  |  | Motif-4 | -157 | - | TGCAATTGGTCAATA |
| P. infestans | PITG\_00704 | 272 | Motif-0 | -137 | - | GTAGATCAGAAAGTGA |
|  |  |  | Motif-4 | -180 | - | GGTGATTGGCTCATA |
| P. infestans | PITG\_00705 | 272 | Motif-0 | -151 | + | GTAGATCAGAAAGTGA |
|  |  |  | Motif-4 | -107 | + | GGTGATTGGCTCATA |
| P. infestans | PITG\_00708 | 412 | Motif-3 | -60 | + | ACTTGGAGTTTGTAT |
| P. infestans | PITG\_00709 | 412 | Motif-3 | -366 | + | TACAAACTCCAAGTCC |
| P. infestans | PITG\_00710 | 346 | Motif-0 | -125 | - | GGCAAATGGAGAGTGC |
|  |  |  | Motif-1 | -151 | + | TACATGTAC |
| P. infestans | PITG\_00711 | 238 | Motif-9 | -35 | + | GAGTACCGGTA |
| P. infestans | PITG\_00712 | 1000 | Motif-0 | -65 | + | TCACTACCGGAATTGC |
|  |  |  | Motif-1 | -276 | + | AGTACCGGTACAAGAG |
|  |  |  | Motif-9 | -277 | + | AAGTACCGGTA |
| P. infestans | PITG\_00715 | 446 | Motif-4 | -192 | + | ATTCAACGAATGGGA |
| P. infestans | PITG\_00716 | 730 | Motif-4 | -202 | - | TCCCATTCGTCAAAA |
| P. infestans | PITG\_00717 | 730 | Motif-4 | -58 | - | GCCGATTGGCAGAAA |
| P. infestans | PITG\_00718 | 904 | Motif-1 | -414 | - | TACAAGTACT |
|  |  |  | Motif-2 | -151 | - | AAAGTTGAAGCT |
|  |  |  | Motif-7 | -478 | + | TACTACTAATA |
| P. infestans | PITG\_00719 | 1000 | Motif-0 | -48 | + | CATTTCGGAATTTGCA |
|  |  |  | Motif-1 | -472 | + | TACATGTAT |
|  |  |  | Motif-2 | -204 | + | CTCGTTGAAGTT |
|  |  |  | Motif-4 | -95 | - | TCTGATTGGCTGAGA |
| P. infestans | PITG\_00720 | 1000 | Motif-0 | -72 | - | GCAAATTTGATTGTGA |
|  |  |  | Motif-1 | -950 | + | TACATGTAC |
|  |  |  | Motif-2 | -680 | + | GACGGCAACTTGCC |
| P. infestans | PITG\_00721 | 958 | Motif-1 | -574 | + | TACATGTAG |
|  |  |  | Motif-2 | -437 | + | ATCTCATCAACTTGTA |
|  |  |  | Motif-6 | -941 | + | CCCCCCCCCCCGCTCG |
| P. infestans | PITG\_00722 | 1000 | Motif-1 | -107 | + | TACATGAAC |
|  |  |  | Motif-9 | -87 | - | CGGTACCGGTA |
| P. infestans | PITG\_00723 | 1000 | Motif-16 | -535 | + | CAGTGGCAGCACCAGC |
|  |  |  | Motif-2 | -843 | - | GTAGTTGAAGTG |
| P. infestans | PITG\_00724 | 449 | Motif-1 | -284 | + | TACATGTAT |
|  |  |  | Motif-2 | -15 | + | TCCGTTGAAGTGGC |
| P. infestans | PITG\_00725 | 101 | Motif-1 | -76 | - | TACATGAAC |
| P. infestans | PITG\_00726 | 1000 | Motif-3 | -61 | - | CTCTGGGGGTTTGTAT |
|  |  |  | Motif-4 | -565 | + | TGTGAGTGGTTAAAA |
| P. infestans | PITG\_00729 | 1000 | Motif-0 | -916 | - | GCGAATCTTAAACTGA |
|  |  |  | Motif-1 | -178 | + | TACATGTAC |
| P. infestans | PITG\_00730 | 1000 | Motif-0 | -481 | - | GCAAATCGCCAAATGC |
|  |  |  | Motif-7 | -188 | + | CAGTATTAATA |
| P. infestans | PITG\_00731 | 665 | Motif-0 | -415 | + | CCATTTCACATTTTGG |
|  |  |  | Motif-1 | -189 | + | TACATGTAC |
|  |  |  | Motif-6 | -311 | - | CGACCCCCCCCCCCAT |
| P. infestans | PITG\_00732 | 1000 | Motif-1 | -547 | + | TACATGTAC |
|  |  |  | Motif-3 | -469 | - | GACTTGGGTCTTGTTT |
| P. infestans | PITG\_00733 | 1000 | Motif-0 | -28 | + | TCACTTCCGCATTTCG |
|  |  |  | Motif-1 | -709 | + | TACATGTAT |
|  |  |  | Motif-3 | -632 | + | TAAAAACTTCGAGTCA |
|  |  |  | Motif-4 | -192 | + | TCTGATTGGGCAAAA |
| P. infestans | PITG\_00734 | 535 | Motif-0 | -59 | - | GCAAATCTGGTAGTGA |
|  |  |  | Motif-17 | -166 | - | AGTGTAGCCATTTCT |
| P. infestans | PITG\_00735 | 1000 | Motif-1 | -338 | + | TACGTGTAC |
|  |  |  | Motif-3 | -577 | + | TCTTACCTTTTGTAT |
| P. infestans | PITG\_00737 | 1000 | Motif-0 | -67 | + | CAGTTTCAATTTCGCT |
|  |  |  | Motif-3 | -253 | + | ACTTGGTTTTTGTAA |
|  |  |  | Motif-4 | -418 | + | TCGGATTGGCCAAAT |
| P. infestans | PITG\_00739 | 1000 | Motif-0 | -562 | - | TCAGTTTTACTTCTAC |
| P. infestans | PITG\_00740 | 1000 | Motif-0 | -610 | - | GAGAATTGCTAAATGA |
|  |  |  | Motif-2 | -188 | + | CATTTTGAAGTTGA |
| P. infestans | PITG\_00741 | 1000 | Motif-0 | -562 | - | TCAGTTTTACTTCTAC |
| P. infestans | PITG\_00743 | 1000 | Motif-2 | -107 | + | TCAGTTCAACATCG |
|  |  |  | Motif-3 | -155 | - | CACTGAGTGTTTGTAA |
|  |  |  | Motif-8 | -883 | + | TAATTTTTATTTTCA |
| P. infestans | PITG\_00745 | 1000 | Motif-1 | -76 | - | TACATGAAC |
|  |  |  | Motif-4 | -657 | - | TTTGATTGGACGTTA |
| P. infestans | PITG\_00746 | 305 | Motif-0 | -44 | + | TCATTATCCGTTCTTC |
| P. infestans | PITG\_00747 | 330 | Motif-1 | -20 | + | TACATGTAG |
|  |  |  | Motif-2 | -137 | - | AGCTTCAACCT |
|  |  |  | Motif-3 | -260 | - | CATACACAGGCCAAGT |
| P. infestans | PITG\_00748 | 330 | Motif-1 | -318 | + | TACATGTAG |
|  |  |  | Motif-2 | -70 | - | GGCGTTGAAGTG |
|  |  |  | Motif-3 | -84 | + | TACACAGGCCAAGTCA |
| P. infestans | PITG\_00752 | 1000 | Motif-8 | -520 | + | TTTTACTTAACAAAA |
| P. infestans | PITG\_00753 | 633 | Motif-0 | -435 | + | TCACTTTTGATTTCTG |
|  |  |  | Motif-2 | -602 | + | ACTTCAAGATG |
| P. infestans | PITG\_00754 | 633 | Motif-0 | -214 | - | TCACTTTTGATTTCTG |
|  |  |  | Motif-2 | -42 | - | ACTTCAAGATG |
| P. infestans | PITG\_00757 | 1000 | Motif-0 | -39 | - | GGAGATTGACGAATGA |
|  |  |  | Motif-2 | -86 | - | ACTTCAAGATG |
|  |  |  | Motif-4 | -341 | + | TACCAGCCAATAGAA |
| P. infestans | PITG\_00760 | 1000 | Motif-2 | -476 | - | GACTTCAACTT |
|  |  |  | Motif-4 | -456 | - | TCCGATTGGTCAGAT |
| P. infestans | PITG\_00761 | 1000 | Motif-4 | -252 | - | GCTGCTTGGCCAAAA |
|  |  |  | Motif-8 | -750 | + | TATTATTATATTATA |
| P. infestans | PITG\_00762 | 684 | Motif-2 | -587 | - | CGACTTCAACAGGC |
| P. infestans | PITG\_00763 | 684 | Motif-2 | -111 | + | CGACTTCAACAGGC |
| P. infestans | PITG\_00764 | 163 | Motif-0 | -153 | + | TCAAATTTGCAAATGA |
| P. infestans | PITG\_00765 | 163 | Motif-0 | -26 | - | TCAAATTTGCAAATGA |
| P. infestans | PITG\_00766 | 278 | Motif-8 | -146 | - | TTTTATTTTTTAAAT |
| P. infestans | PITG\_00770 | 603 | Motif-1 | -465 | + | TACATGTAG |
| P. infestans | PITG\_00771 | 872 | Motif-0 | -23 | - | TTAAAATCGAAAATGA |
|  |  |  | Motif-1 | -96 | + | TACATGTAT |
|  |  |  | Motif-2 | -760 | + | GCTGCAACTTG |
|  |  |  | Motif-4 | -660 | - | TCTGATTGGACCATT |
|  |  |  | Motif-7 | -558 | - | TATTATTAATA |
| P. infestans | PITG\_00772 | 872 | Motif-0 | -865 | + | TTAAAATCGAAAATGA |
|  |  |  | Motif-1 | -784 | + | TACATGTAT |
|  |  |  | Motif-2 | -123 | - | GCTGCAACTTG |
|  |  |  | Motif-4 | -227 | + | TCTGATTGGACCATT |
|  |  |  | Motif-7 | -322 | - | AATTATTAATA |
| P. infestans | PITG\_00773 | 1000 | Motif-0 | -277 | + | CATTCTCGCATTTTCC |
|  |  |  | Motif-2 | -409 | - | CTTGTTGAAGTG |
| P. infestans | PITG\_00774 | 1000 | Motif-0 | -54 | + | CATTCGACAATTTCCC |
|  |  |  | Motif-1 | -417 | + | TACATGTAG |
|  |  |  | Motif-2 | -883 | + | CATATTGAAGTT |
|  |  |  | Motif-6 | -980 | + | CGTCCCCCCCCCAACC |
|  |  |  | Motif-7 | -814 | + | CAATATTAATA |
| P. infestans | PITG\_00777 | 1000 | Motif-1 | -310 | - | TATATATGTATTT |
|  |  |  | Motif-3 | -271 | + | ACTTAGTTTTTTTAT |
|  |  |  | Motif-8 | -263 | + | TTTTTATAATTAAAA |
| P. infestans | PITG\_00778 | 1000 | Motif-0 | -374 | - | ATCACTTCTTCTTTT |
| P. infestans | PITG\_00782 | 1000 | Motif-0 | -394 | - | GCTAAACGGCAAATGA |
| P. infestans | PITG\_00783 | 1000 | Motif-1 | -517 | + | TACATGTAT |
|  |  |  | Motif-16 | -175 | - | CAACAGTGGCACCAAC |
|  |  |  | Motif-4 | -68 | + | TTTCACTGGCTGAAT |
| P. infestans | PITG\_00786 | 1000 | Motif-4 | -737 | + | TACGATTGGTCAGAA |
| P. infestans | PITG\_00787 | 1000 | Motif-1 | -54 | + | ACTGCATGTAG |
|  |  |  | Motif-4 | -479 | - | TACGATTGGTCAAAA |
| P. infestans | PITG\_00789 | 1000 | Motif-0 | -71 | + | CATTCCCCAATTTCCC |
| P. infestans | PITG\_00791 | 1000 | Motif-1 | -157 | - | TACATGAGCAATTTAT |
|  |  |  | Motif-2 | -909 | - | GCTGTTGAAGTTAC |
|  |  |  | Motif-4 | -59 | - | TGCGATTGGCGAATA |
|  |  |  | Motif-8 | -743 | + | TTTTTTTAAATTGTT |
| P. infestans | PITG\_00792 | 1000 | Motif-0 | -68 | - | TTAAATTGGAAAGTGA |
|  |  |  | Motif-1 | -537 | + | TACATGTAT |
|  |  |  | Motif-4 | -911 | - | TATCAGCTAATCCCA |
|  |  |  | Motif-8 | -397 | - | TATGTTTTATTTTTA |
| P. infestans | PITG\_00793 | 1000 | Motif-0 | -35 | - | GCTAATTCTGAAATGA |
|  |  |  | Motif-1 | -990 | - | TGTACATCTAC |
|  |  |  | Motif-3 | -537 | + | ATTTAGACATTGTAT |
|  |  |  | Motif-4 | -421 | - | TGTGATTGGCTCATT |
| P. infestans | PITG\_00794 | 1000 | Motif-4 | -79 | + | TTCGATTGGCTCGAA |
| P. infestans | PITG\_00795 | 1000 | Motif-0 | -336 | - | GTAAAGTCGAGAATGA |
|  |  |  | Motif-3 | -443 | + | TCTTGATATTTGTAT |
|  |  |  | Motif-7 | -871 | - | TATTATTAATA |
| P. infestans | PITG\_00796 | 1000 | Motif-1 | -428 | + | TACATGTAC |
| P. infestans | PITG\_00797 | 1000 | Motif-3 | -429 | + | TACACGCACCAAATCA |
| P. infestans | PITG\_00799 | 1000 | Motif-0 | -42 | + | CCATTTTCAAAACTAC |
|  |  |  | Motif-1 | -226 | + | TACATGCAC |
|  |  |  | Motif-7 | -535 | + | CAGTATTAATA |
| P. infestans | PITG\_00800 | 1000 | Motif-1 | -476 | + | TACTTGTACA |
|  |  |  | Motif-7 | -630 | - | AATTATTAATA |
| P. infestans | PITG\_00804 | 1000 | Motif-1 | -826 | - | TACGTGTAC |
|  |  |  | Motif-9 | -284 | + | CCGTACCGGTA |
| P. infestans | PITG\_00805 | 454 | Motif-1 | -243 | + | AGTACCTGTAGCAGA |
|  |  |  | Motif-8 | -440 | - | TTTTTTTTATCTACA |
| P. infestans | PITG\_00806 | 1000 | Motif-1 | -590 | + | AACATGTAC |
| P. infestans | PITG\_00807 | 1000 | Motif-0 | -27 | + | TCATTTTCAAAGTAAC |
|  |  |  | Motif-1 | -233 | + | TACAAGTACA |
|  |  |  | Motif-16 | -749 | + | CAACAGCAACAGCAGC |
|  |  |  | Motif-4 | -205 | - | CGTCAGTGGATGAAA |
| P. infestans | PITG\_00808 | 1000 | Motif-1 | -301 | + | TACATGTAT |
| P. infestans | PITG\_00809 | 384 | Motif-3 | -175 | + | ATACAAACTTCAATAT |
| P. infestans | PITG\_00816 | 1000 | Motif-0 | -279 | - | CCATTCGAGATTTTGC |
|  |  |  | Motif-3 | -125 | + | ATTTGATTTCAGTAT |
| P. infestans | PITG\_00819 | 297 | Motif-0 | -266 | + | GAAGATTGGAGAGTGA |
| P. infestans | PITG\_00820 | 297 | Motif-0 | -47 | - | GAAGATTGGAGAGTGA |
| P. infestans | PITG\_00821 | 1000 | Motif-0 | -62 | - | GCGAGTTGAAGAATGA |
|  |  |  | Motif-1 | -192 | - | CTCTTTTACATGTTT |
|  |  |  | Motif-17 | -651 | + | AGTGTAGCCATGCGA |
|  |  |  | Motif-4 | -781 | - | CCTGATTGGGCAATA |
| P. infestans | PITG\_00827 | 1000 | Motif-2 | -42 | + | CCATTTCAAGTTCC |
| P. infestans | PITG\_00831 | 1000 | Motif-0 | -62 | + | CATTTTCAATTTCGCC |
|  |  |  | Motif-1 | -672 | - | AAAACGTGTACCGGTA |
|  |  |  | Motif-2 | -645 | + | AGCGTTGAAGTGGG |
|  |  |  | Motif-7 | -985 | - | TACTATTAATA |
|  |  |  | Motif-9 | -672 | - | GTGTACCGGTA |
| P. infestans | PITG\_00832 | 1000 | Motif-1 | -900 | - | TCCCAGTACACGTTT |
| P. infestans | PITG\_00833 | 1000 | Motif-0 | -69 | + | TCATTCATCGTTTCTC |
|  |  |  | Motif-1 | -362 | + | TACATGTAG |
|  |  |  | Motif-2 | -117 | + | CAGGTTGACGTG |
|  |  |  | Motif-4 | -897 | - | TCTGATTGGCAAAAA |
| P. infestans | PITG\_00834 | 1000 | Motif-0 | -56 | - | GAAAATCGAGAAATGA |
| P. infestans | PITG\_00839 | 1000 | Motif-0 | -55 | - | GAAAATCGAGAAATGA |
|  |  |  | Motif-3 | -480 | + | ACTTATTAATAGTAT |
|  |  |  | Motif-7 | -477 | - | TACTATTAATA |
| P. infestans | PITG\_00840 | 1000 | Motif-1 | -492 | - | ATAACATGTA |
| P. infestans | PITG\_00841 | 1000 | Motif-0 | -66 | + | CATTATCAAATTCGCC |
|  |  |  | Motif-1 | -635 | + | AACATGTAC |
| P. infestans | PITG\_00842 | 690 | Motif-0 | -212 | + | GCACTGCGAATTTTGC |
|  |  |  | Motif-1 | -378 | - | TACATATAC |
| P. infestans | PITG\_00843 | 690 | Motif-0 | -22 | - | GAAGATTGAAGAATGA |
|  |  |  | Motif-1 | -313 | - | TACATGTGCG |
| P. infestans | PITG\_00844 | 121 | Motif-0 | -19 | + | CATTTCCGAATTTCTC |
|  |  |  | Motif-1 | -112 | + | TACATGTAG |
|  |  |  | Motif-4 | -89 | - | GTTGATGGGTTAAAA |
| P. infestans | PITG\_00845 | 1000 | Motif-0 | -707 | + | TAAAATTCAGAACTGA |
|  |  |  | Motif-1 | -630 | + | ATTAAATGTA |
|  |  |  | Motif-2 | -749 | + | CACGTTGAAGCA |
| P. infestans | PITG\_00846 | 1000 | Motif-8 | -796 | + | TTTTTCTTAATAAAT |
| P. infestans | PITG\_00847 | 1000 | Motif-8 | -366 | - | TTTTATTAAGCAAGA |
| P. infestans | PITG\_00848 | 1000 | Motif-0 | -303 | - | TCACTCACCAATCTGT |
|  |  |  | Motif-1 | -963 | - | TACATGTGCT |
|  |  |  | Motif-4 | -259 | - | TCCGATTGGCCGGAA |
| P. infestans | PITG\_00849 | 1000 | Motif-8 | -526 | - | TTTTTTTTAGTTATT |
| P. infestans | PITG\_00854 | 1000 | Motif-0 | -994 | - | TCACTACCCAAACCGC |
|  |  |  | Motif-1 | -224 | + | TACATATAC |
|  |  |  | Motif-3 | -979 | + | ACTTCATTTTTGTAT |
|  |  |  | Motif-9 | -259 | - | CTGTACCGGTA |
| P. infestans | PITG\_00855 | 1000 | Motif-0 | -44 | + | TCACTACCCAAACCGC |
|  |  |  | Motif-1 | -807 | - | TACATATAC |
|  |  |  | Motif-3 | -57 | + | TACAAAAATGAAGTCA |
|  |  |  | Motif-9 | -774 | + | CTGTACCGGTA |
| P. infestans | PITG\_00856 | 347 | Motif-1 | -184 | + | TACATGTAA |
| P. infestans | PITG\_00857 | 887 | Motif-1 | -129 | + | TACATGTAC |
|  |  |  | Motif-2 | -190 | - | AACTTCAACTT |
|  |  |  | Motif-4 | -506 | - | GTTTATTGGTCAAAA |
| P. infestans | PITG\_00858 | 887 | Motif-1 | -766 | + | TACATGTAC |
|  |  |  | Motif-2 | -552 | - | CCTTCAACGTATCAAA |
|  |  |  | Motif-4 | -396 | + | GTTTATTGGTCAAAA |
| P. infestans | PITG\_00859 | 255 | Motif-4 | -20 | - | CATTATTGGCTAGTA |
| P. infestans | PITG\_00860 | 1000 | Motif-1 | -384 | + | ATGACATGTAT |
|  |  |  | Motif-2 | -644 | + | GCTTCAACATTCGCCA |
| P. infestans | PITG\_00861 | 1000 | Motif-1 | -103 | - | ACTACAGGTA |
|  |  |  | Motif-2 | -970 | - | CGCTTCAACAT |
|  |  |  | Motif-3 | -208 | + | ACTTAAAGTTTGTAA |
| P. infestans | PITG\_00865 | 1000 | Motif-6 | -307 | - | TCCAACCCCCCCCCCC |
| P. infestans | PITG\_00866 | 1000 | Motif-1 | -199 | + | AACATGTAC |
| P. infestans | PITG\_00867 | 1000 | Motif-0 | -879 | + | GAAAAACCTAGAATGA |
|  |  |  | Motif-16 | -37 | + | CCGCCGCAGCGGCGAC |
|  |  |  | Motif-2 | -815 | - | CAACTTCAACAAA |
| P. infestans | PITG\_00868 | 630 | Motif-1 | -72 | + | TACATGTAT |
|  |  |  | Motif-3 | -126 | + | ACTTAGGTTTTGTAT |
| P. infestans | PITG\_00869 | 338 | Motif-0 | -312 | - | TCACTCCCCAATTTG |
| P. infestans | PITG\_00870 | 338 | Motif-0 | -40 | + | CACTCCCCAATTTGAC |
| P. infestans | PITG\_00871 | 1000 | Motif-0 | -32 | + | TCATTCCGCAACACGC |
|  |  |  | Motif-1 | -164 | + | TACATGTAC |
|  |  |  | Motif-3 | -998 | + | TACAAAACTCAAGTAA |
| P. infestans | PITG\_00872 | 1000 | Motif-0 | -54 | - | GCAGACTCAAAAATGA |
|  |  |  | Motif-1 | -169 | + | TACATGTAA |
| P. infestans | PITG\_00874 | 1000 | Motif-8 | -912 | - | TATTTCTATTTAAAA |
| P. infestans | PITG\_00875 | 1000 | Motif-0 | -530 | + | CATTCGTGAATTTGCT |
|  |  |  | Motif-3 | -777 | + | ACTTGACATTTGTAA |
| P. infestans | PITG\_00877 | 509 | Motif-0 | -275 | + | TCACTCTGCAAATATC |
|  |  |  | Motif-4 | -42 | - | GCTGATCGGTCGATA |
| P. infestans | PITG\_00878 | 509 | Motif-0 | -250 | - | TCACTCTGCAAATATC |
|  |  |  | Motif-4 | -482 | + | GCTGATCGGTCGATA |
| P. infestans | PITG\_00879 | 280 | Motif-4 | -138 | - | TTTGATTGGTGGAAA |
| P. infestans | PITG\_00880 | 1000 | Motif-1 | -497 | - | GTTACATGAAG |
|  |  |  | Motif-2 | -190 | - | GGCTTCAACGT |
| P. infestans | PITG\_00881 | 1000 | Motif-1 | -103 | + | TACATGAAC |
| P. infestans | PITG\_00882 | 1000 | Motif-1 | -335 | + | TACGTGTAC |
|  |  |  | Motif-17 | -468 | + | AATGTAGCCATATTT |
| P. infestans | PITG\_00883 | 1000 | Motif-0 | -746 | + | GAAAATTGAAAAGTTA |
|  |  |  | Motif-4 | -114 | + | TTTGATTGGTGGACA |
| P. infestans | PITG\_00884 | 1000 | Motif-0 | -406 | - | GAAAATTGAAAAGTTA |
|  |  |  | Motif-4 | -97 | + | TGTGATTCGTCGAAA |
| P. infestans | PITG\_00885 | 155 | Motif-4 | -43 | - | GCTGACTGGCTAAAA |
| P. infestans | PITG\_00886 | 248 | Motif-1 | -199 | - | ACAACATGTA |
| P. infestans | PITG\_00887 | 103 | Motif-0 | -79 | - | CCATTTCTAAATCTTA |
| P. infestans | PITG\_00888 | 1000 | Motif-1 | -414 | + | TACATGTAC |
|  |  |  | Motif-2 | -239 | + | GTTGTTGAAGTC |
|  |  |  | Motif-3 | -177 | + | ACTTGGCTTCACTAT |
| P. infestans | PITG\_00889 | 1000 | Motif-2 | -416 | - | GAGGTTGAAGCT |
|  |  |  | Motif-3 | -436 | - | ACTTAATCTCAGTAT |
|  |  |  | Motif-4 | -167 | + | TTTCATTGGCTAAAA |
| P. infestans | PITG\_00891 | 1000 | Motif-1 | -261 | + | TACATGTAC |
|  |  |  | Motif-16 | -543 | + | CGGCAGCAGCAGCGAC |
|  |  |  | Motif-4 | -227 | + | TATAATTGGCTGATT |
| P. infestans | PITG\_00892 | 767 | Motif-0 | -64 | + | CATTTCCAAAGTTGCC |
|  |  |  | Motif-1 | -276 | + | TACATGTAT |
| P. infestans | PITG\_00893 | 767 | Motif-0 | -718 | + | GCAACTTTGGAAATGA |
|  |  |  | Motif-1 | -499 | + | TACATGTAG |
| P. infestans | PITG\_00894 | 1000 | Motif-3 | -211 | + | TACAAAATCCAAGTAA |
| P. infestans | PITG\_00896 | 1000 | Motif-2 | -358 | - | CGGCAAGTTGCAGGC |
|  |  |  | Motif-4 | -421 | + | TATGATTGGTCTAAA |
| P. infestans | PITG\_00898 | 619 | Motif-1 | -179 | + | TACATGTAC |
|  |  |  | Motif-2 | -234 | + | ACTGCAACATG |
| P. infestans | PITG\_00899 | 619 | Motif-1 | -448 | + | TACATGTAG |
|  |  |  | Motif-2 | -396 | + | CATGTTGCAGTG |
| P. infestans | PITG\_00906 | 1000 | Motif-0 | -174 | - | TCATTTTCTCTTCGAC |
| P. infestans | PITG\_00907 | 1000 | Motif-0 | -140 | - | GGAATTTGTAAAATGA |
|  |  |  | Motif-1 | -263 | - | TCAAGGTACATGTGT |
| P. infestans | PITG\_00908 | 1000 | Motif-3 | -107 | - | ATCTCGGAGTTTGTAT |
| P. infestans | PITG\_00911 | 440 | Motif-1 | -151 | + | TACATGTAC |
|  |  |  | Motif-9 | -65 | + | AAGTACCGGTA |
| P. infestans | PITG\_00912 | 692 | Motif-1 | -200 | + | TACATGTAC |
|  |  |  | Motif-2 | -86 | + | CAACTTGAAGTT |
| P. infestans | PITG\_00913 | 382 | Motif-2 | -103 | + | CCGCTTCAAGCTCC |
| P. infestans | PITG\_00914 | 377 | Motif-1 | -283 | + | GCAACATGTAATGCA |
|  |  |  | Motif-2 | -286 | + | ACTGCAACATG |
| P. infestans | PITG\_00916 | 675 | Motif-0 | -507 | + | GAAGATTGGAAAATGG |
|  |  |  | Motif-1 | -181 | + | TACATGTAG |
|  |  |  | Motif-2 | -353 | - | CACTTCAACTT |
| P. infestans | PITG\_00917 | 675 | Motif-0 | -184 | - | GAAGATTGGAAAATGG |
|  |  |  | Motif-1 | -502 | + | TACATGTAG |
|  |  |  | Motif-2 | -332 | + | ACTTCAACTTG |
| P. infestans | PITG\_00919 | 835 | Motif-0 | -47 | + | CATTCTCCAACTTGCT |
| P. infestans | PITG\_00921 | 1000 | Motif-0 | -70 | + | TCATTTAAGCTTCTGC |
|  |  |  | Motif-1 | -828 | + | TACATGCAC |
|  |  |  | Motif-17 | -132 | + | GCTGTACCCATCTGT |
| P. infestans | PITG\_00923 | 1000 | Motif-1 | -140 | - | AATACCGGTACCGGTA |
|  |  |  | Motif-9 | -137 | + | CGGTACCGGTA |
| P. infestans | PITG\_00926 | 809 | Motif-0 | -791 | - | TCACAATAGAATTTGC |
|  |  |  | Motif-1 | -490 | + | TACATGTAC |
| P. infestans | PITG\_00927 | 809 | Motif-0 | -34 | + | TCACAATAGAATTTGC |
|  |  |  | Motif-1 | -112 | + | TACATGTTCCCTTGA |
| P. infestans | PITG\_00928 | 1000 | Motif-0 | -146 | - | TTGAATTGTGAAATGA |
|  |  |  | Motif-1 | -530 | + | TACATGTAG |
| P. infestans | PITG\_00929 | 1000 | Motif-2 | -67 | + | CATCTTGAAGTG |
| P. infestans | PITG\_00930 | 462 | Motif-4 | -93 | + | TCCCATTGGTCAAAA |
| P. infestans | PITG\_00931 | 184 | Motif-17 | -170 | + | AATGTAGCCATACTT |
|  |  |  | Motif-4 | -72 | - | TCTGGTTGGTTAAAA |
|  |  |  | Motif-8 | -81 | + | TTTGTCTTATTTTAA |
| P. infestans | PITG\_00932 | 184 | Motif-17 | -29 | - | AATGTAGCCATACTT |
|  |  |  | Motif-4 | -94 | + | TAAGATTGGCTAATA |
|  |  |  | Motif-8 | -118 | - | TTTGTCTTATTTTAA |
| P. infestans | PITG\_00933 | 1000 | Motif-0 | -180 | + | GCAAAATTGATACTGA |
|  |  |  | Motif-4 | -39 | - | TATGATTGGTCAAAA |
| P. infestans | PITG\_00934 | 983 | Motif-1 | -260 | + | TACATGTAG |
| P. infestans | PITG\_00935 | 409 | Motif-0 | -49 | + | CATTTGTGAATTTGCG |
|  |  |  | Motif-6 | -391 | - | ATCACCCCCCCCCCCC |
| P. infestans | PITG\_00936 | 368 | Motif-0 | -91 | + | TCACTCCGCACGTGTC |
|  |  |  | Motif-8 | -18 | + | TATTATTAAACAGAA |
| P. infestans | PITG\_00937 | 368 | Motif-0 | -293 | - | TCACTCCGCACGTGTC |
|  |  |  | Motif-8 | -365 | - | TATTATTAAACAGAA |
| P. infestans | PITG\_00938 | 993 | Motif-0 | -326 | + | TCACTCACAATTGTCC |
|  |  |  | Motif-17 | -901 | + | ACTGTAGCCATCTCA |
|  |  |  | Motif-7 | -748 | - | TACTATTAATA |
|  |  |  | Motif-8 | -189 | - | TATTATTAAATATCA |
| P. infestans | PITG\_00939 | 993 | Motif-0 | -52 | + | TCACTATCGCTTTCCC |
|  |  |  | Motif-17 | -107 | - | ACTGTAGCCATCTCA |
|  |  |  | Motif-7 | -253 | - | AAATATTAATA |
|  |  |  | Motif-8 | -819 | + | TATTATTAAATATCA |
| P. infestans | PITG\_00940 | 641 | Motif-0 | -518 | - | TCAGTGATGATTCTAC |
|  |  |  | Motif-3 | -197 | - | AACTTGATATTTGTAT |
|  |  |  | Motif-4 | -173 | - | GTTGATTGGCTGTTA |
| P. infestans | PITG\_00941 | 641 | Motif-0 | -139 | + | TCAGTGATGATTCTAC |
|  |  |  | Motif-3 | -459 | + | ACTTGATATTTGTAT |
|  |  |  | Motif-4 | -483 | + | GTTGATTGGCTGTTA |
| P. infestans | PITG\_00943 | 1000 | Motif-1 | -36 | + | TACATGTAA |
| P. infestans | PITG\_00944 | 1000 | Motif-0 | -540 | - | TCATTTCTCCTCTCGA |
|  |  |  | Motif-1 | -288 | - | TGGACATGTAC |
|  |  |  | Motif-18 | -319 | - | TTGTGTGGTACACATA |
| P. infestans | PITG\_00945 | 1000 | Motif-1 | -244 | - | TGCATGTAC |
|  |  |  | Motif-7 | -147 | - | TATTATTAATA |
|  |  |  | Motif-9 | -447 | - | ATGTACCGGTA |
| P. infestans | PITG\_00948 | 1000 | Motif-1 | -57 | - | AAAACATGAACCGTA |
| P. infestans | PITG\_00952 | 1000 | Motif-0 | -691 | + | CATTCTCACATTTACC |
|  |  |  | Motif-1 | -111 | + | TACATGTAT |
|  |  |  | Motif-3 | -110 | - | AATACAAAATACATGT |
|  |  |  | Motif-9 | -132 | - | GTGTACCGGTA |
| P. infestans | PITG\_00953 | 127 | Motif-0 | -92 | - | TCATTATTCAATCGTA |
| P. infestans | PITG\_00954 | 127 | Motif-0 | -51 | + | TCATTATTCAATCGTA |
| P. infestans | PITG\_00955 | 1000 | Motif-4 | -63 | - | TCTGATTGGCTGTAA |
|  |  |  | Motif-6 | -514 | - | CGCCCCCCCCCCCAAA |
| P. infestans | PITG\_00957 | 1000 | Motif-1 | -17 | + | TACATGTAC |
|  |  |  | Motif-8 | -59 | + | TTTTTTTTAATTGTA |
| P. infestans | PITG\_00960 | 1000 | Motif-0 | -136 | + | GCACTTCCCTTTTTAC |
|  |  |  | Motif-4 | -44 | - | GATGATTGGCTAAAA |
|  |  |  | Motif-8 | -117 | - | TTATTTTTATTAATA |
| P. infestans | PITG\_00962 | 438 | Motif-0 | -300 | - | GCGAAACTGGGACTGA |
|  |  |  | Motif-2 | -158 | - | TCCTCTTCAAGCTGTA |
| P. infestans | PITG\_00964 | 658 | Motif-3 | -262 | - | GTGTTGGGATTTGTAT |
| P. infestans | PITG\_00965 | 658 | Motif-3 | -286 | + | ACTTGGCCTTTGTAA |
| P. infestans | PITG\_00967 | 1000 | Motif-0 | -66 | - | GCCAAAATGGAAATGA |
| P. infestans | PITG\_00970 | 368 | Motif-0 | -139 | - | GAAAAATGAGAAGCGA |
| P. infestans | PITG\_00971 | 368 | Motif-0 | -162 | - | TTATTTTTCTATTTGA |
| P. infestans | PITG\_00972 | 551 | Motif-0 | -63 | + | CATTCCCGAATTTGCC |
|  |  |  | Motif-1 | -352 | + | TACATGTAT |
|  |  |  | Motif-7 | -247 | - | TACTATTAGTA |
|  |  |  | Motif-9 | -329 | + | CAGTACCGGTA |
| P. infestans | PITG\_00973 | 522 | Motif-0 | -16 | + | TCACTCTGACTTTGCC |
|  |  |  | Motif-1 | -381 | - | TACATGAAC |
|  |  |  | Motif-2 | -268 | - | TAAGTTGCAGTG |
|  |  |  | Motif-7 | -395 | - | TATTATTAATA |
| P. infestans | PITG\_00974 | 522 | Motif-0 | -103 | - | TCAAATTGGGAGATGA |
|  |  |  | Motif-1 | -132 | - | TATTCATGTATTA |
|  |  |  | Motif-2 | -179 | - | GCTTCAACACG |
|  |  |  | Motif-7 | -138 | + | TATTATTAATA |
| P. infestans | PITG\_00977 | 1000 | Motif-2 | -167 | - | CAGCCTCAACATC |
| P. infestans | PITG\_00979 | 304 | Motif-0 | -76 | + | GCATTAAGCAACTCGC |
| P. infestans | PITG\_00980 | 289 | Motif-18 | -63 | - | TAATGTGGTGTACATA |
| P. infestans | PITG\_00981 | 289 | Motif-18 | -242 | + | TAATGTGGTGTACATA |
| P. infestans | PITG\_00982 | 1000 | Motif-0 | -481 | - | TCACTTCCACATCCAA |
| P. infestans | PITG\_00983 | 131 | Motif-0 | -117 | + | GCAATTTAGAGAATGA |
| P. infestans | PITG\_00985 | 347 | Motif-0 | -32 | + | CCATTCCTGAAGTTTC |
| P. infestans | PITG\_00986 | 347 | Motif-0 | -331 | - | CCATTCCTGAAGTTTC |
| P. infestans | PITG\_00987 | 352 | Motif-6 | -26 | + | CGCCCTTCCTCCCCAC |
| P. infestans | PITG\_00988 | 1000 | Motif-3 | -218 | + | TCTTGACATTTGTAA |
|  |  |  | Motif-4 | -177 | - | AACGATTGGTCAAAA |
| P. infestans | PITG\_00989 | 1000 | Motif-4 | -183 | + | GACGATTGGACGAAA |
| P. infestans | PITG\_00990 | 449 | Motif-0 | -430 | - | CCATTCTTCATCTGTC |
|  |  |  | Motif-2 | -322 | - | TAAGTTGAAGCT |
|  |  |  | Motif-4 | -93 | + | TCTGATTGAACAAAA |
| P. infestans | PITG\_00991 | 449 | Motif-0 | -35 | + | CCATTCTTCATCTGTC |
|  |  |  | Motif-2 | -138 | - | AGCTTCAACTT |
|  |  |  | Motif-4 | -210 | - | TACCATTGGATGAAA |
| P. infestans | PITG\_00994 | 1000 | Motif-4 | -102 | - | GCTGATTGGCTGAAA |
|  |  |  | Motif-6 | -674 | - | GCAGCCCCCCCCCACC |
| P. infestans | PITG\_00996 | 713 | Motif-0 | -598 | - | TCATTCTTCGACTTTT |
|  |  |  | Motif-1 | -104 | + | TACGTGTAC |
| P. infestans | PITG\_00997 | 1000 | Motif-2 | -184 | - | ACTTCAACACC |
|  |  |  | Motif-9 | -27 | - | ATGTTCCGGTA |
| P. infestans | PITG\_00999 | 1000 | Motif-0 | -56 | - | GCAAAAGCGAAAGTGA |
|  |  |  | Motif-2 | -676 | - | AGCTTCAACTT |
|  |  |  | Motif-3 | -625 | + | TCTTGGGTTTTGTAA |
|  |  |  | Motif-6 | -20 | + | CCACCCACCACCTCAC |
| P. infestans | PITG\_01000 | 440 | Motif-9 | -332 | + | GTGTACCGGTA |
| P. infestans | PITG\_01001 | 1000 | Motif-2 | -237 | + | CAGATTGAAGTG |
| P. infestans | PITG\_01002 | 1000 | Motif-3 | -20 | - | GACTTGATTATTGTAC |
| P. infestans | PITG\_01003 | 472 | Motif-18 | -358 | - | TTGTGTAGTGTACAAA |
|  |  |  | Motif-2 | -28 | - | CTTGTTGAAGCG |
| P. infestans | PITG\_01004 | 472 | Motif-18 | -130 | + | TTGTGTAGTGTACAAA |
|  |  |  | Motif-2 | -456 | + | CTTGTTGAAGCG |
| P. infestans | PITG\_01005 | 397 | Motif-0 | -34 | - | GAAGGATGCAAAATGA |
|  |  |  | Motif-1 | -76 | - | TACGTGTAC |
|  |  |  | Motif-18 | -85 | + | TCGTGTGATGTACACG |
|  |  |  | Motif-2 | -68 | - | CAAATTGAAGTT |
| P. infestans | PITG\_01007 | 1000 | Motif-0 | -983 | + | CATTTTTTATTTCGCC |
| P. infestans | PITG\_01008 | 1000 | Motif-2 | -144 | + | GAGGTTGAATCG |
|  |  |  | Motif-3 | -231 | - | ACTTAGTGTTTGTTG |
| P. infestans | PITG\_01009 | 942 | Motif-0 | -153 | + | CATTTTAAAATTTGCC |
|  |  |  | Motif-1 | -783 | + | ATTAAATGTA |
|  |  |  | Motif-2 | -221 | + | GCTTCAACATG |
|  |  |  | Motif-6 | -92 | + | CCCCCCCCCCCGTTAT |
|  |  |  | Motif-8 | -419 | - | TTTTTTTAATTTTTA |
| P. infestans | PITG\_01013 | 1000 | Motif-0 | -959 | + | GCAGTCTCGATTTGCC |
| P. infestans | PITG\_01015 | 1000 | Motif-0 | -48 | + | CATTTTTTATTTCGCC |
|  |  |  | Motif-1 | -371 | - | TGCATGTAC |
|  |  |  | Motif-2 | -717 | + | GAGGTTGAAGCC |
| P. infestans | PITG\_01019 | 1000 | Motif-0 | -543 | - | GTAGATTGCGTAGTGA |
|  |  |  | Motif-17 | -76 | + | AATGTACCCATGTCT |
| P. infestans | PITG\_01020 | 1000 | Motif-1 | -579 | + | TACATGAAC |
|  |  |  | Motif-6 | -518 | - | GCCACGCGCCCCTCCT |
| P. infestans | PITG\_01023 | 582 | Motif-0 | -31 | + | TCAGTTGTACTTTTAC |
|  |  |  | Motif-9 | -63 | + | AAGTACCGGTA |
| P. infestans | PITG\_01024 | 753 | Motif-2 | -559 | - | CACTTCAACGT |
|  |  |  | Motif-7 | -391 | - | TATTATTAATA |
| P. infestans | PITG\_01025 | 466 | Motif-0 | -296 | + | CCATTTCACTATTCGC |
|  |  |  | Motif-7 | -407 | - | TATTATTAATA |
| P. infestans | PITG\_01026 | 1000 | Motif-0 | -30 | - | GAAGATTGCGGAATGG |
|  |  |  | Motif-1 | -639 | + | TACATGTAG |
|  |  |  | Motif-2 | -86 | - | AGCTTCAACTT |
|  |  |  | Motif-4 | -301 | + | TATCAACGAATCATC |
| P. infestans | PITG\_01027 | 1000 | Motif-0 | -511 | + | TCAGTATCGAACTCTC |
|  |  |  | Motif-1 | -430 | + | TACATGTAT |
| P. infestans | PITG\_01028 | 1000 | Motif-0 | -445 | + | TCATTGTTATTTTGGC |
|  |  |  | Motif-4 | -536 | - | TCCGATTGGTTAAAA |
|  |  |  | Motif-8 | -546 | + | TTTGTTTTAATTTTA |
| P. infestans | PITG\_01029 | 1000 | Motif-0 | -43 | + | TCATTTCCTTTTTTCC |
|  |  |  | Motif-1 | -349 | + | ACTATACAAGTTGT |
|  |  |  | Motif-8 | -249 | + | TTTATTTATTTTAAA |
| P. infestans | PITG\_01030 | 1000 | Motif-0 | -124 | + | TAATTCTTCAATTTAA |
|  |  |  | Motif-1 | -474 | + | TACATGTAG |
|  |  |  | Motif-6 | -625 | - | GCCCCCCCCTCCCCGC |
|  |  |  | Motif-9 | -141 | - | CTGTACCGGTA |
| P. infestans | PITG\_01031 | 1000 | Motif-1 | -302 | + | TACATGTAT |
|  |  |  | Motif-2 | -333 | - | AGCTCCAACTT |
| P. infestans | PITG\_01033 | 1000 | Motif-1 | -661 | + | TACATGTAT |
|  |  |  | Motif-2 | -907 | - | TATGTTGACGCG |
| P. infestans | PITG\_01038 | 905 | Motif-0 | -126 | - | TCAAAATTGAAAGTGA |
|  |  |  | Motif-1 | -381 | + | TACATGTGTA |
|  |  |  | Motif-2 | -457 | + | GCATCAACGTCTCCAA |
| P. infestans | PITG\_01039 | 505 | Motif-0 | -254 | + | ACGAATTCAAGAATGA |
| P. infestans | PITG\_01040 | 505 | Motif-0 | -266 | + | CATTCTTGAATTCGTT |
| P. infestans | PITG\_01041 | 372 | Motif-4 | -289 | + | CATGATTGGCTGAAA |
| P. infestans | PITG\_01042 | 372 | Motif-4 | -98 | - | CATGATTGGCTGAAA |
| P. infestans | PITG\_01043 | 1000 | Motif-1 | -97 | - | TGTTGATGTATCG |
|  |  |  | Motif-3 | -329 | + | AAACAAAACTCAAGCT |
|  |  |  | Motif-7 | -175 | - | TATTATTAATA |
|  |  |  | Motif-8 | -179 | + | TTTTTATTAATAATA |
| P. infestans | PITG\_01044 | 1000 | Motif-0 | -21 | + | TCACTTCACCATCGGC |
|  |  |  | Motif-1 | -383 | - | AACATGTATTAACAT |
|  |  |  | Motif-2 | -21 | + | TCACTTCACCATC |
|  |  |  | Motif-3 | -816 | - | AAAACAAAACTCAAGC |
|  |  |  | Motif-7 | -966 | + | TATTATTAATA |
|  |  |  | Motif-8 | -484 | - | TATTTTTTTTTAAAA |
| P. infestans | PITG\_01047 | 1000 | Motif-3 | -182 | - | GACTTAGGGTTTGTAT |
| P. infestans | PITG\_01048 | 1000 | Motif-1 | -237 | + | AGTACCGGTACTTTA |
|  |  |  | Motif-9 | -235 | - | AAGTACCGGTA |
| P. infestans | PITG\_01049 | 1000 | Motif-1 | -470 | + | TACATGTAC |
|  |  |  | Motif-2 | -896 | - | CACTTCAACGT |
|  |  |  | Motif-8 | -189 | - | TTTTAATAATTTTAA |
| P. infestans | PITG\_01050 | 1000 | Motif-0 | -86 | + | CATTCTTCAATTCGCC |
|  |  |  | Motif-1 | -303 | + | TACATGTAA |
|  |  |  | Motif-4 | -284 | + | TCCGATTGGCGAGAA |
|  |  |  | Motif-6 | -323 | - | ACAGCCCCCCCCCCCT |
|  |  |  | Motif-9 | -501 | - | ATGTACCGGTA |
| P. infestans | PITG\_01053 | 964 | Motif-0 | -89 | - | GCGAATCGAGGAATGA |
|  |  |  | Motif-2 | -267 | + | AACGGCAACTCGCA |
| P. infestans | PITG\_01062 | 1000 | Motif-0 | -413 | - | GCAAGTTTTGGAATGA |
|  |  |  | Motif-1 | -559 | + | ATTAAATGTAT |
|  |  |  | Motif-6 | -40 | + | GCCCCCCCCCCCAAAT |
| P. infestans | PITG\_01064 | 1000 | Motif-0 | -406 | - | TCAGTACAAAATTTAC |
|  |  |  | Motif-1 | -259 | + | TACTTGTACA |
| P. infestans | PITG\_01065 | 1000 | Motif-0 | -268 | - | TCACTATCCAATACCC |
|  |  |  | Motif-1 | -194 | + | TACATGTAA |
|  |  |  | Motif-2 | -182 | + | TAAATTGAAGTG |
| P. infestans | PITG\_01066 | 1000 | Motif-0 | -876 | + | TCACTATCCAATACCC |
|  |  |  | Motif-1 | -942 | + | TACATGTAC |
|  |  |  | Motif-2 | -162 | + | ACTTCAAGGTG |
| P. infestans | PITG\_01068 | 1000 | Motif-0 | -646 | - | CCATTTTGCATTTCAA |
|  |  |  | Motif-1 | -889 | + | AGTACATGAAG |
|  |  |  | Motif-9 | -904 | - | CTTTACCGGTA |
| P. infestans | PITG\_01069 | 565 | Motif-0 | -104 | + | GCAATATGGAAACTGG |
|  |  |  | Motif-1 | -172 | - | ATTACATGCAT |
|  |  |  | Motif-2 | -40 | - | ACTGCAAGATG |
|  |  |  | Motif-6 | -398 | + | TGCCCCCCCCCCCAAT |
| P. infestans | PITG\_01070 | 1000 | Motif-1 | -228 | - | GTTAATTACCTGTAC |
|  |  |  | Motif-3 | -626 | - | GCTGGGCATTTGTAT |
|  |  |  | Motif-7 | -593 | - | CAGTATTAATA |
|  |  |  | Motif-8 | -520 | + | TTTGTTTAAATAATA |
| P. infestans | PITG\_01071 | 1000 | Motif-0 | -80 | + | CATTTTCGATTTTGCC |
|  |  |  | Motif-1 | -545 | + | TACATGTAC |
|  |  |  | Motif-3 | -534 | + | GCTGGGCATTTGTAT |
|  |  |  | Motif-7 | -554 | + | TACTATTAATA |
|  |  |  | Motif-8 | -585 | + | TATTTTTAAATAATA |
| P. infestans | PITG\_01072 | 542 | Motif-0 | -83 | + | CATTCTTGCATTTGCT |
|  |  |  | Motif-2 | -368 | + | CGAGTTGAAGTG |
|  |  |  | Motif-4 | -348 | + | TACGATTGGTCCAAA |
| P. infestans | PITG\_01073 | 360 | Motif-3 | -203 | + | TCTTGTTCTTTGTAT |
|  |  |  | Motif-4 | -246 | + | TCCCATTGGTCAACT |
| P. infestans | PITG\_01074 | 776 | Motif-0 | -23 | - | GCAGATTCGATAATGC |
|  |  |  | Motif-1 | -596 | + | TGCATGTAC |
|  |  |  | Motif-2 | -270 | - | CCCTTCAACGT |
|  |  |  | Motif-7 | -386 | + | CAGTATTAATA |
| P. infestans | PITG\_01075 | 776 | Motif-0 | -267 | + | TAAGTCTTCGACTTGC |
|  |  |  | Motif-1 | -188 | + | TACATGCAC |
|  |  |  | Motif-2 | -485 | - | GACTGCAACCTGGA |
|  |  |  | Motif-7 | -401 | - | CAGTATTAATA |
| P. infestans | PITG\_01076 | 455 | Motif-2 | -119 | + | ACTTCAACGTC |
| P. infestans | PITG\_01078 | 1000 | Motif-1 | -524 | + | CCTAAATGTAT |
| P. infestans | PITG\_01079 | 1000 | Motif-1 | -681 | - | ACAATACATTTACT |
| P. infestans | PITG\_01080 | 1000 | Motif-0 | -490 | - | GCAAAATTGTAAATGA |
|  |  |  | Motif-2 | -602 | + | GTAGTTGAAGTT |
| P. infestans | PITG\_01082 | 1000 | Motif-18 | -142 | + | TAGTGTGGTACACAAA |
|  |  |  | Motif-4 | -181 | - | TTCTATTGGATAAAA |
| P. infestans | PITG\_01083 | 1000 | Motif-1 | -826 | - | TACATATAC |
|  |  |  | Motif-2 | -439 | + | GGCGAAGATGAAGGTG |
| P. infestans | PITG\_01084 | 92 | Motif-2 | -67 | - | TATGTTGAAGTT |
| P. infestans | PITG\_01085 | 1000 | Motif-16 | -972 | + | CCGCAGACGCAGCAGC |
| P. infestans | PITG\_01086 | 410 | Motif-1 | -144 | + | TACATGTAT |
| P. infestans | PITG\_01087 | 1000 | Motif-4 | -80 | - | TTTGATTGGCTGAAA |
| P. infestans | PITG\_01088 | 1000 | Motif-0 | -37 | - | CCACTTCTCCATCCTC |
| P. infestans | PITG\_01090 | 1000 | Motif-2 | -484 | + | GATATTGAAGTT |
|  |  |  | Motif-3 | -173 | + | AATACAAAACGGAAAT |
|  |  |  | Motif-8 | -934 | - | TTTGTTTAATTTTAT |
|  |  |  | Motif-9 | -185 | + | ATTTACCGGTA |
| P. infestans | PITG\_01092 | 1000 | Motif-1 | -388 | + | TACATGTAT |
|  |  |  | Motif-2 | -88 | - | AAATTTGAAGTGGA |
|  |  |  | Motif-4 | -814 | + | TGTCAGTGGCCAACA |
| P. infestans | PITG\_01094 | 1000 | Motif-2 | -952 | + | GATGTCGAAGTTAA |
| P. infestans | PITG\_01098 | 271 | Motif-2 | -71 | - | GAGTACGCTGAAGCGG |
| P. infestans | PITG\_01099 | 987 | Motif-1 | -894 | - | AACATGTATCGATTA |
|  |  |  | Motif-8 | -279 | + | TTTGTATTATTAAAT |
| P. infestans | PITG\_01100 | 362 | Motif-0 | -301 | - | TCACTTCACAATTTC |
|  |  |  | Motif-1 | -244 | + | TACATGTAT |
|  |  |  | Motif-2 | -60 | - | ACAACTTCAACGCATC |
| P. infestans | PITG\_01101 | 362 | Motif-0 | -75 | + | CACTTCACAATTTCCT |
|  |  |  | Motif-1 | -126 | + | TACATGTAC |
|  |  |  | Motif-2 | -318 | + | ACAACTTCAACGCATC |
| P. infestans | PITG\_01102 | 425 | Motif-1 | -270 | + | TACATGTAT |
| P. infestans | PITG\_01103 | 425 | Motif-1 | -151 | + | TACATGTAT |
| P. infestans | PITG\_01106 | 1000 | Motif-4 | -686 | - | TTTTAGCCAATTCCC |
| P. infestans | PITG\_01107 | 1000 | Motif-4 | -660 | + | TTTTAGCCAATTCCC |
| P. infestans | PITG\_01108 | 1000 | Motif-1 | -914 | + | ACTATATGTAG |
|  |  |  | Motif-4 | -67 | + | TTTAATTGGCCGAAA |
| P. infestans | PITG\_01109 | 444 | Motif-0 | -224 | + | GCATTCACGATTTTCC |
|  |  |  | Motif-2 | -333 | - | CTGGTTGAAGTG |
| P. infestans | PITG\_01110 | 248 | Motif-0 | -50 | - | GCAATTTGAAAAGTGA |
|  |  |  | Motif-2 | -86 | + | CACGTTGACGTG |
| P. infestans | PITG\_01111 | 551 | Motif-1 | -359 | + | TACATGTAG |
|  |  |  | Motif-17 | -272 | - | ACTGTAGCCATCTTG |
| P. infestans | PITG\_01112 | 551 | Motif-1 | -200 | + | TACATGTAC |
|  |  |  | Motif-17 | -294 | + | ACTGTAGCCATCTTG |
| P. infestans | PITG\_01113 | 382 | Motif-0 | -139 | + | TCAGTCTCCATTCAAC |
| P. infestans | PITG\_01114 | 382 | Motif-0 | -259 | - | TCAGTCTCCATTCAAC |
| P. infestans | PITG\_01115 | 1000 | Motif-0 | -359 | + | CATTCGTCAATTCACC |
|  |  |  | Motif-16 | -133 | + | GAACAGTAACAGCAGC |
| P. infestans | PITG\_01116 | 161 | Motif-2 | -95 | - | GCTGCAACTTG |
| P. infestans | PITG\_01117 | 324 | Motif-0 | -53 | - | CCACTGCCGCTTTTTC |
| P. infestans | PITG\_01118 | 385 | Motif-1 | -223 | + | TGCATGTAC |
|  |  |  | Motif-17 | -254 | - | AGTGTAGCCATCTCT |
| P. infestans | PITG\_01119 | 385 | Motif-1 | -171 | - | TGCATGTAC |
|  |  |  | Motif-17 | -146 | + | AGTGTAGCCATCTCT |
| P. infestans | PITG\_01120 | 458 | Motif-0 | -23 | - | GCAACTTGGGGAGTGA |
| P. infestans | PITG\_01121 | 1000 | Motif-3 | -607 | + | ACTTATAGTTTGTAT |
|  |  |  | Motif-4 | -53 | - | CGTGATTGGTAGATA |
|  |  |  | Motif-7 | -172 | - | CATTATTAATA |
| P. infestans | PITG\_01122 | 1000 | Motif-1 | -684 | - | CCTAAATGTAT |
| P. infestans | PITG\_01123 | 1000 | Motif-1 | -51 | + | TACGTGTAGTGGTACC |
|  |  |  | Motif-18 | -515 | - | TTGTGTGGTGTGCACA |
|  |  |  | Motif-7 | -202 | + | TACTACTAATA |
| P. infestans | PITG\_01124 | 319 | Motif-1 | -187 | - | TACATGTGCA |
| P. infestans | PITG\_01125 | 319 | Motif-1 | -142 | + | TACATGTGCA |
| P. infestans | PITG\_01127 | 1000 | Motif-1 | -583 | - | TGTACATCTAT |
| P. infestans | PITG\_01128 | 591 | Motif-0 | -215 | - | GCAAAAACAAAACTGA |
|  |  |  | Motif-2 | -111 | - | CGCTTCAACGT |
| P. infestans | PITG\_01129 | 591 | Motif-0 | -392 | + | GCAAAAACAAAACTGA |
|  |  |  | Motif-2 | -425 | + | AACTGCAAATTGCA |
| P. infestans | PITG\_01130 | 720 | Motif-0 | -157 | - | GAAGATTGAGAAGTGA |
|  |  |  | Motif-2 | -311 | - | CACTTCAACCT |
| P. infestans | PITG\_01131 | 1000 | Motif-0 | -55 | - | GCGAATTGTAAAATGG |
|  |  |  | Motif-1 | -371 | - | GTACTGTACATATAT |
| P. infestans | PITG\_01132 | 1000 | Motif-0 | -55 | - | GCGAATTGTAAAATGG |
|  |  |  | Motif-1 | -371 | - | GTACTGTACATATAT |
| P. infestans | PITG\_01133 | 1000 | Motif-0 | -62 | - | GCAAAATCTGCAATGA |
|  |  |  | Motif-1 | -958 | + | AACATGTAGTTTTGA |
|  |  |  | Motif-2 | -253 | + | CGGCAACTTGCAGCA |
|  |  |  | Motif-6 | -899 | - | GGCCCCTCCCCCCACT |
| P. infestans | PITG\_01134 | 1000 | Motif-16 | -908 | - | GAACAGCAGCAGCAGG |
|  |  |  | Motif-7 | -988 | + | TACTATTAGTA |
| P. infestans | PITG\_01135 | 274 | Motif-0 | -238 | + | ATCACTTTTATTTTT |
|  |  |  | Motif-2 | -61 | + | ACTGCAACTTG |
|  |  |  | Motif-4 | -92 | - | CTTGATTGGTCGAAC |
| P. infestans | PITG\_01136 | 274 | Motif-0 | -51 | - | ATCACTTTTATTTTT |
|  |  |  | Motif-2 | -224 | + | CAAGTTGCAGTG |
|  |  |  | Motif-4 | -197 | + | CTTGATTGGTCGAAC |
| P. infestans | PITG\_01138 | 895 | Motif-2 | -145 | + | ACTTCAACATG |
|  |  |  | Motif-3 | -56 | + | ACTTGGCTTCAGTAT |
| P. infestans | PITG\_01139 | 765 | Motif-1 | -189 | - | TACATGCATA |
|  |  |  | Motif-2 | -498 | - | CAAGCTGAAGTG |
|  |  |  | Motif-4 | -443 | - | CACGATTGGTCAAAA |
| P. infestans | PITG\_01140 | 1000 | Motif-0 | -656 | - | TCCGTCCGGAATCGGC |
| P. infestans | PITG\_01142 | 1000 | Motif-0 | -400 | + | CCAGTCCTTGATTCAC |
|  |  |  | Motif-1 | -850 | + | TACATATAC |
|  |  |  | Motif-18 | -834 | + | TAGTGTTGTGTACATA |
|  |  |  | Motif-2 | -592 | - | CACTGCAAAATGTC |
| P. infestans | PITG\_01143 | 399 | Motif-1 | -80 | + | TACATGTAC |
|  |  |  | Motif-7 | -346 | - | AACTATTAATA |
| P. infestans | PITG\_01144 | 1000 | Motif-0 | -36 | + | CCATTTCTTCATTTAC |
|  |  |  | Motif-1 | -500 | - | TACACGTATATTTTT |
|  |  |  | Motif-4 | -511 | + | TTTGAATGGATAAAA |
| P. infestans | PITG\_01145 | 1000 | Motif-4 | -101 | + | TTTCATTGGCTATAA |
|  |  |  | Motif-9 | -46 | - | GCGTACCGGTA |
| P. infestans | PITG\_01146 | 663 | Motif-0 | -123 | + | TCATTTTTCGAGTCTC |
| P. infestans | PITG\_01151 | 719 | Motif-1 | -122 | + | TGCATGTAGAAATCT |
|  |  |  | Motif-8 | -173 | - | TAATTTTTAATAGAA |
| P. infestans | PITG\_01152 | 1000 | Motif-0 | -525 | + | CCACTTGCCTATCCGC |
|  |  |  | Motif-2 | -634 | - | GGCTTCAACGT |
| P. infestans | PITG\_01154 | 678 | Motif-1 | -287 | + | TGCATGTAGACGTCG |
|  |  |  | Motif-2 | -60 | - | CATATTGAAGCG |
| P. infestans | PITG\_01155 | 678 | Motif-1 | -406 | - | TGCATGTAGACGTCG |
|  |  |  | Motif-2 | -630 | + | CATATTGAAGCG |
| P. infestans | PITG\_01156 | 1000 | Motif-0 | -161 | - | CCACTTGTGATTCTGC |
|  |  |  | Motif-4 | -66 | + | TTCTATTGGCTAAAA |
| P. infestans | PITG\_01157 | 624 | Motif-0 | -604 | + | ATTTTTTTTTTACTTT |
| P. infestans | PITG\_01158 | 624 | Motif-0 | -36 | - | ATTTTTTTTTTACTTT |
| P. infestans | PITG\_01160 | 1000 | Motif-0 | -62 | + | TCATTCGTCAAGTTAT |
| P. infestans | PITG\_01161 | 1000 | Motif-2 | -117 | + | CAAGTTGATGCG |
|  |  |  | Motif-8 | -61 | - | TACTATTTAGTTAAA |
| P. infestans | PITG\_01162 | 1000 | Motif-1 | -598 | + | TACATGTAT |
| P. infestans | PITG\_01163 | 1000 | Motif-2 | -147 | + | GTTGTTGAAGTT |
| P. infestans | PITG\_01164 | 205 | Motif-0 | -42 | + | CACTTTGAAACTCGTC |
| P. infestans | PITG\_01165 | 1000 | Motif-0 | -344 | + | CACTCTGCAAATTGCC |
|  |  |  | Motif-1 | -813 | + | TACATGTAC |
|  |  |  | Motif-2 | -508 | + | ACTTCAAGATG |
|  |  |  | Motif-8 | -994 | + | TTTTTTTAAGTTGTA |
| P. infestans | PITG\_01169 | 1000 | Motif-8 | -91 | - | TTTATTTTAACTAAA |
| P. infestans | PITG\_01171 | 336 | Motif-0 | -214 | - | TCATTCTTCAATTTG |
|  |  |  | Motif-3 | -56 | + | TACAAAATACAAGTGA |
|  |  |  | Motif-9 | -161 | + | CGGTACCGGTA |
| P. infestans | PITG\_01172 | 336 | Motif-0 | -136 | + | CATTCTTCAATTTGCG |
|  |  |  | Motif-3 | -294 | + | ACTTGTATTTTGTAT |
|  |  |  | Motif-9 | -178 | + | CCGTACCGGTA |
| P. infestans | PITG\_01178 | 1000 | Motif-1 | -788 | + | TACATGTGTA |
| P. infestans | PITG\_01180 | 889 | Motif-0 | -850 | - | TCATTCCGATATCTTC |
|  |  |  | Motif-1 | -537 | - | AGTACCGGTACTATA |
|  |  |  | Motif-2 | -705 | - | CAGGTTGATGTG |
|  |  |  | Motif-9 | -294 | - | AAGTACCGGTA |
| P. infestans | PITG\_01182 | 1000 | Motif-0 | -312 | - | TGATTCTGCAATTTGA |
|  |  |  | Motif-1 | -663 | + | TACATGTAG |
|  |  |  | Motif-9 | -519 | - | ATGTACCGGTA |
| P. infestans | PITG\_01183 | 424 | Motif-1 | -388 | + | AATGCATCTACCGGTA |
|  |  |  | Motif-2 | -102 | - | CAGTTTGAAGTG |
|  |  |  | Motif-3 | -149 | - | TCTTAACCTTTGTAT |
| P. infestans | PITG\_01184 | 351 | Motif-2 | -162 | + | CTCTTCAACTT |
| P. infestans | PITG\_01185 | 351 | Motif-2 | -200 | - | CTCTTCAACTT |
| P. infestans | PITG\_01186 | 610 | Motif-1 | -232 | + | ACCACGTGTACAGTA |
|  |  |  | Motif-2 | -427 | - | GAACTTCATCATG |
| P. infestans | PITG\_01187 | 1000 | Motif-2 | -1000 | + | GTCGTTGAAGTG |
| P. infestans | PITG\_01188 | 1000 | Motif-0 | -54 | + | CATTCCAGAATTCGCT |
|  |  |  | Motif-4 | -137 | - | TCTGATTGGCTGACG |
|  |  |  | Motif-9 | -340 | + | CAGTACCGGTA |
| P. infestans | PITG\_01189 | 1000 | Motif-3 | -171 | - | GACTCAGGGTTTGTAA |
| P. infestans | PITG\_01192 | 715 | Motif-0 | -699 | + | GTAAATTAATGAATGA |
|  |  |  | Motif-1 | -353 | + | TACATGTAC |
|  |  |  | Motif-2 | -368 | - | CACTTCAACAT |
| P. infestans | PITG\_01193 | 715 | Motif-0 | -32 | - | GTAAATTAATGAATGA |
|  |  |  | Motif-1 | -370 | + | TACATGTAT |
|  |  |  | Motif-2 | -357 | + | ACTTCAACATC |
| P. infestans | PITG\_01195 | 1000 | Motif-1 | -536 | + | TACAAGTACT |
|  |  |  | Motif-2 | -41 | - | GATGTTGACGTT |
| P. infestans | PITG\_01198 | 1000 | Motif-1 | -257 | - | ACTAAATGTA |
|  |  |  | Motif-9 | -16 | - | ATTTACCGGTA |
| P. infestans | PITG\_01199 | 898 | Motif-0 | -40 | - | GTTAATCCAGAAATGA |
| P. infestans | PITG\_01200 | 880 | Motif-1 | -420 | + | TACATGTAC |
|  |  |  | Motif-9 | -417 | + | ATGTACCGGTA |
| P. infestans | PITG\_01201 | 136 | Motif-4 | -44 | - | TTTCATTGGGTGAAA |
| P. infestans | PITG\_01202 | 1000 | Motif-0 | -30 | - | GCAGGTTTGCGAGTGA |
|  |  |  | Motif-2 | -157 | + | ACATCAACGTAGCTCT |
|  |  |  | Motif-4 | -989 | + | TATGATTGGCCATTA |
| P. infestans | PITG\_01204 | 130 | Motif-4 | -60 | - | TGCGATTGGCTAAAA |
| P. infestans | PITG\_01205 | 130 | Motif-4 | -85 | + | TGCGATTGGCTAAAA |
| P. infestans | PITG\_01206 | 292 | Motif-0 | -109 | + | TCACTTCTGAGTCTGA |
| P. infestans | PITG\_01207 | 292 | Motif-0 | -199 | - | TCACTTCTGAGTCTGA |
| P. infestans | PITG\_01208 | 237 | Motif-0 | -43 | + | CACTTCGGATTTTGCC |
|  |  |  | Motif-2 | -140 | - | GTGATTGAAGTGGA |
|  |  |  | Motif-4 | -93 | - | GCTGATTGGCTGAAT |
| P. infestans | PITG\_01210 | 753 | Motif-0 | -51 | + | TCATTACACAATTGTC |
|  |  |  | Motif-1 | -669 | + | CTCCTCTTCATGTAC |
| P. infestans | PITG\_01211 | 753 | Motif-0 | -718 | - | TCATTACACAATTGTC |
|  |  |  | Motif-1 | -99 | - | CTCCTCTTCATGTAC |
| P. infestans | PITG\_01212 | 1000 | Motif-0 | -178 | + | CACTATTGAATTTATT |
| P. infestans | PITG\_01214 | 1000 | Motif-0 | -973 | - | GCATTATCGCTTTTCC |
|  |  |  | Motif-1 | -323 | + | TACATGTAC |
| P. infestans | PITG\_01215 | 56 | Motif-4 | -34 | - | GATGATTGGTTGGAA |
| P. infestans | PITG\_01216 | 312 | Motif-17 | -288 | - | ACTGTAGCTATCCTG |
| P. infestans | PITG\_01217 | 486 | Motif-4 | -74 | + | TATGACTGGTTAAAA |
| P. infestans | PITG\_01218 | 770 | Motif-1 | -211 | + | TACATGTAA |
|  |  |  | Motif-4 | -16 | - | TCTCCTTGGTTAAAT |
| P. infestans | PITG\_01219 | 1000 | Motif-1 | -301 | + | TGCATGTAC |
|  |  |  | Motif-17 | -196 | - | AATGTAGCCATGTTC |
| P. infestans | PITG\_01222 | 1000 | Motif-2 | -404 | - | CTGGAAGTTGCAGCT |
|  |  |  | Motif-4 | -685 | + | AATCATTGGATAAAA |
| P. infestans | PITG\_01223 | 607 | Motif-0 | -52 | - | GCAGAATACAAAGTGA |
| P. infestans | PITG\_01224 | 607 | Motif-0 | -449 | - | CTAAATTCGAAAGTGA |
| P. infestans | PITG\_01226 | 149 | Motif-4 | -87 | - | GTTGAGTGGCTGAAA |
|  |  |  | Motif-7 | -57 | - | AATTATTAATA |
| P. infestans | PITG\_01227 | 149 | Motif-4 | -77 | + | GTTGAGTGGCTGAAA |
|  |  |  | Motif-7 | -103 | + | AATTATTAATA |
| P. infestans | PITG\_01228 | 379 | Motif-2 | -246 | - | CCGCTTCATCATC |
|  |  |  | Motif-4 | -339 | + | GACGATTGGCCAATT |
|  |  |  | Motif-8 | -135 | + | TTTAATTTAGTAACA |
| P. infestans | PITG\_01229 | 379 | Motif-2 | -146 | + | CCGCTTCATCATC |
|  |  |  | Motif-4 | -55 | - | GACGATTGGCCAATT |
|  |  |  | Motif-8 | -259 | - | TTTAATTTAGTAACA |
| P. infestans | PITG\_01230 | 927 | Motif-0 | -886 | + | GCAGGTTTGCAAATGA |
|  |  |  | Motif-1 | -302 | + | TACATGTAC |
|  |  |  | Motif-2 | -649 | - | CTAGTTGAAGTT |
| P. infestans | PITG\_01231 | 927 | Motif-0 | -57 | - | GCAGGTTTGCAAATGA |
|  |  |  | Motif-1 | -313 | - | TACATGAAC |
|  |  |  | Motif-2 | -290 | + | CTAGTTGAAGTT |
| P. infestans | PITG\_01232 | 585 | Motif-7 | -527 | - | TAATATTAATA |
| P. infestans | PITG\_01233 | 1000 | Motif-0 | -186 | + | CACTTTTCCTTTTGCT |
|  |  |  | Motif-2 | -752 | + | CACGGCAACTCGAC |
| P. infestans | PITG\_01235 | 130 | Motif-4 | -77 | + | TATGATTGGCTGCAA |
| P. infestans | PITG\_01236 | 130 | Motif-4 | -68 | - | TATGATTGGCTGCAA |
| P. infestans | PITG\_01237 | 825 | Motif-0 | -19 | + | CATTTTCCCACTTGTG |
| P. infestans | PITG\_01238 | 825 | Motif-0 | -822 | - | CATTTTCCCACTTGTG |
| P. infestans | PITG\_01239 | 1000 | Motif-8 | -193 | + | TTTTTTTTAATAAAT |
| P. infestans | PITG\_01240 | 355 | Motif-17 | -231 | + | AGTGTAGCCACTTTT |
|  |  |  | Motif-3 | -308 | + | ACTTGAATTTAGTAA |
|  |  |  | Motif-8 | -288 | - | TATTTTTTAAAAAAA |
| P. infestans | PITG\_01241 | 355 | Motif-17 | -139 | - | AGTGTAGCCACTTTT |
|  |  |  | Motif-3 | -61 | + | TACTAAATTCAAGTCA |
|  |  |  | Motif-8 | -82 | + | TATTTTTTAAAAAAA |
| P. infestans | PITG\_01243 | 447 | Motif-1 | -93 | + | TACATGTAT |
| P. infestans | PITG\_01244 | 447 | Motif-1 | -362 | + | TACATGTAG |
| P. infestans | PITG\_01245 | 169 | Motif-4 | -92 | - | CGTGATTGGGTGAAA |
| P. infestans | PITG\_01246 | 127 | Motif-1 | -35 | + | TACATGTAT |
| P. infestans | PITG\_01247 | 127 | Motif-1 | -100 | + | TACATGTAC |
| P. infestans | PITG\_01248 | 639 | Motif-17 | -564 | - | AATGTAGCCATTCCT |
|  |  |  | Motif-18 | -422 | - | TATTGTGGTCCACACG |
|  |  |  | Motif-4 | -309 | - | TTTGATTGGCTAATT |
|  |  |  | Motif-7 | -324 | - | AAATATTAATA |
| P. infestans | PITG\_01249 | 639 | Motif-17 | -90 | + | AATGTAGCCATTCCT |
|  |  |  | Motif-18 | -233 | + | TATTGTGGTCCACACG |
|  |  |  | Motif-4 | -173 | + | CGCGATTGGCTGAAA |
|  |  |  | Motif-7 | -323 | - | CAATATTAATA |
| P. infestans | PITG\_01250 | 776 | Motif-2 | -766 | + | CAAGTTGAAGTA |
|  |  |  | Motif-4 | -638 | + | GCTGATTGGTCATTA |
| P. infestans | PITG\_01251 | 1000 | Motif-16 | -881 | + | CAGCGGTCGCAGCAGC |
| P. infestans | PITG\_01252 | 1000 | Motif-1 | -173 | + | TGGACATGTAC |
|  |  |  | Motif-2 | -122 | + | ACTTCAACTTC |
|  |  |  | Motif-3 | -157 | - | TTACAAATGCCAAAAA |
| P. infestans | PITG\_01253 | 1000 | Motif-0 | -51 | - | GCAAAAAGCAGAATGG |
|  |  |  | Motif-1 | -336 | + | AGTACAGGTATCGCT |
|  |  |  | Motif-2 | -457 | + | ACGTCAACATG |
|  |  |  | Motif-6 | -630 | + | CCCCCCCCCCCGGAAC |
|  |  |  | Motif-7 | -834 | + | TATTATTAGTA |
| P. infestans | PITG\_01260 | 306 | Motif-4 | -138 | - | TCCGATTGGCTGAAG |
| P. infestans | PITG\_01261 | 1000 | Motif-2 | -997 | + | ACTCCAACATG |
| P. infestans | PITG\_01262 | 1000 | Motif-0 | -154 | - | TCACTTCACGACTTG |
|  |  |  | Motif-2 | -378 | - | AACTCCAACAT |
| P. infestans | PITG\_01263 | 1000 | Motif-2 | -112 | + | CCTTCAACATGTGTCT |
| P. infestans | PITG\_01267 | 547 | Motif-0 | -178 | + | CACTTCTCGATTCGCC |
|  |  |  | Motif-1 | -227 | - | GGTACCGGTACAAGCA |
|  |  |  | Motif-3 | -261 | + | ACTTGGTATTTGTAA |
|  |  |  | Motif-8 | -135 | - | TATTAATTATTATAA |
| P. infestans | PITG\_01269 | 1000 | Motif-0 | -152 | + | CACTCCCCAATTTGCT |
|  |  |  | Motif-1 | -805 | + | TACATGTAC |
| P. infestans | PITG\_01270 | 1000 | Motif-0 | -901 | - | TCACTCCCCAATTTG |
|  |  |  | Motif-1 | -242 | + | TACATGTAG |
| P. infestans | PITG\_01271 | 968 | Motif-0 | -422 | + | GAAAAAGCCGAAGTGA |
|  |  |  | Motif-1 | -156 | - | TACAAGTACA |
|  |  |  | Motif-4 | -56 | + | TTTGATTGGATGAAA |
| P. infestans | PITG\_01272 | 968 | Motif-0 | -562 | - | GAAAAAGCCGAAGTGA |
|  |  |  | Motif-1 | -822 | + | TACAAGTACA |
|  |  |  | Motif-4 | -927 | - | TTTGATTGGATGAAA |
| P. infestans | PITG\_01274 | 521 | Motif-0 | -57 | + | CATTCTCCAATTTGCA |
|  |  |  | Motif-1 | -454 | - | TCCTGCTAAATGTAC |
|  |  |  | Motif-2 | -212 | - | AACGTTGAAGTG |
| P. infestans | PITG\_01275 | 781 | Motif-0 | -49 | + | TCATAACGCAATTCGC |
|  |  |  | Motif-1 | -215 | + | TACAAGTACT |
| P. infestans | PITG\_01276 | 781 | Motif-0 | -748 | - | TCATAACGCAATTCGC |
|  |  |  | Motif-1 | -576 | - | TACAAGTACT |
| P. infestans | PITG\_01277 | 1000 | Motif-0 | -364 | + | GCATTATTGCATTTGC |
|  |  |  | Motif-2 | -658 | + | ACTTCAACTTC |
| P. infestans | PITG\_01278 | 1000 | Motif-0 | -973 | - | GCATTATTGCATTTGC |
|  |  |  | Motif-2 | -673 | - | AACTTCAACTT |
| P. infestans | PITG\_01279 | 112 | Motif-2 | -37 | + | GTAGTTGAAGCG |
| P. infestans | PITG\_01280 | 112 | Motif-2 | -87 | - | GTAGTTGAAGCG |
| P. infestans | PITG\_01282 | 1000 | Motif-0 | -142 | + | TCACTCTTAACTCTCC |
|  |  |  | Motif-18 | -953 | - | TAGTGTGGTGAACCCA |
|  |  |  | Motif-2 | -773 | + | CAAGTTGAAGCA |
| P. infestans | PITG\_01283 | 1000 | Motif-4 | -405 | + | TCGGATTGGTCAAAA |
| P. infestans | PITG\_01284 | 1000 | Motif-4 | -645 | - | TCGGATTGGTCAAAA |
| P. infestans | PITG\_01285 | 446 | Motif-1 | -314 | - | ATTACCGGTACTGTA |
|  |  |  | Motif-3 | -108 | + | TACAAACGCCGAGTCA |
|  |  |  | Motif-9 | -312 | + | CAGTACCGGTA |
| P. infestans | PITG\_01286 | 275 | Motif-0 | -140 | - | TCAGTTCACAATCGTC |
|  |  |  | Motif-4 | -78 | - | CATAATTGGTCAAAA |
| P. infestans | PITG\_01287 | 275 | Motif-0 | -151 | + | TCAGTTCACAATCGTC |
|  |  |  | Motif-4 | -181 | + | TTCGATTGGCTCATA |
| P. infestans | PITG\_01289 | 411 | Motif-0 | -211 | - | TGAAATCGGAGAATGA |
| P. infestans | PITG\_01290 | 411 | Motif-0 | -216 | + | TGAAATCGGAGAATGA |
| P. infestans | PITG\_01291 | 415 | Motif-0 | -188 | + | TCATTTCTGCTTTTAA |
|  |  |  | Motif-9 | -69 | - | AAGTACCGGTA |
| P. infestans | PITG\_01292 | 1000 | Motif-1 | -719 | - | ACCATACATGTCGT |
|  |  |  | Motif-4 | -94 | - | CGTGATTGGAAGAAA |
|  |  |  | Motif-8 | -311 | - | TACTATTAATTATTA |
| P. infestans | PITG\_01296 | 83 | Motif-4 | -55 | + | GTTCATTGGATGAAA |
| P. infestans | PITG\_01297 | 83 | Motif-4 | -43 | - | GTTCATTGGATGAAA |
| P. infestans | PITG\_01298 | 649 | Motif-1 | -525 | + | TACATGTAT |
|  |  |  | Motif-17 | -498 | - | AATGTACCCATTTTA |
|  |  |  | Motif-7 | -385 | + | TACTATTAATA |
|  |  |  | Motif-9 | -531 | - | ATGTACCGGTA |
| P. infestans | PITG\_01299 | 649 | Motif-1 | -102 | - | AGTATACATGTGAT |
|  |  |  | Motif-17 | -166 | + | AATGTACCCATTTTA |
|  |  |  | Motif-7 | -275 | - | TACTATTAATA |
|  |  |  | Motif-9 | -129 | + | ATGTACCGGTA |
| P. infestans | PITG\_01300 | 1000 | Motif-0 | -336 | - | GAAGACTGGAAAATGA |
| P. infestans | PITG\_01301 | 1000 | Motif-0 | -523 | + | GCCGATAGAAGAATGA |
|  |  |  | Motif-1 | -508 | - | TGTGTACGTGTAGT |
| P. infestans | PITG\_01302 | 1000 | Motif-0 | -663 | - | GCCGATAGAAGAATGA |
|  |  |  | Motif-1 | -69 | + | TACATGCAC |
| P. infestans | PITG\_01305 | 855 | Motif-0 | -72 | + | CACTTTCCAATTTGCC |
|  |  |  | Motif-1 | -305 | - | AACATGTATTAATAC |
|  |  |  | Motif-2 | -783 | - | GTGGATGTCGAAGTGG |
|  |  |  | Motif-3 | -429 | + | GACTCGGCTTTCGTAT |
|  |  |  | Motif-7 | -307 | + | AAGTATTAATA |
| P. infestans | PITG\_01306 | 1000 | Motif-0 | -79 | + | TCACTTTACCAGTTGC |
|  |  |  | Motif-1 | -632 | + | TACATGTAC |
|  |  |  | Motif-8 | -655 | + | TTTGTTTAAGTTAAA |
| P. infestans | PITG\_01307 | 1000 | Motif-1 | -451 | + | TACATGCAC |
|  |  |  | Motif-8 | -963 | - | TTTGTTTAAGTTAAA |
| P. infestans | PITG\_01310 | 1000 | Motif-1 | -35 | + | TACATGTAT |
| P. infestans | PITG\_01311 | 381 | Motif-2 | -276 | + | ACTTCAACGTG |
| P. infestans | PITG\_01312 | 381 | Motif-2 | -115 | - | AACTTCAACGT |
| P. infestans | PITG\_01313 | 950 | Motif-4 | -86 | + | ACTGATTGGCTGAAA |
| P. infestans | PITG\_01316 | 1000 | Motif-0 | -138 | + | TCATTTACCAAAGTGC |
|  |  |  | Motif-2 | -979 | - | TTAGTTGAAGCG |
|  |  |  | Motif-7 | -632 | - | TAATATTAATA |
| P. infestans | PITG\_01322 | 1000 | Motif-0 | -342 | + | GCCGATTTGAAAGTGC |
|  |  |  | Motif-1 | -418 | + | AATACCGGTACAGTA |
|  |  |  | Motif-9 | -416 | - | CTGTACCGGTA |
| P. infestans | PITG\_01323 | 1000 | Motif-8 | -838 | - | TTTTATTTATTAAAT |
| P. infestans | PITG\_01324 | 1000 | Motif-4 | -107 | + | ACTGATTGGTTGAAA |
| P. infestans | PITG\_01325 | 1000 | Motif-4 | -379 | - | GCTTATTGGTCGAGA |
|  |  |  | Motif-7 | -981 | - | TACTATTAATA |
| P. infestans | PITG\_01326 | 1000 | Motif-6 | -201 | - | CCACCCACCCACCCAC |
|  |  |  | Motif-9 | -440 | + | AAGTACCGGTA |
| P. infestans | PITG\_01328 | 1000 | Motif-0 | -105 | + | CACTTTTCAATCTGCT |
|  |  |  | Motif-1 | -249 | - | ACTACACGTAT |
|  |  |  | Motif-2 | -54 | + | AACTTCGACTT |
|  |  |  | Motif-3 | -362 | - | GGCTTGGACTTCGTAT |
| P. infestans | PITG\_01329 | 278 | Motif-1 | -73 | + | TACATGTAG |
|  |  |  | Motif-8 | -53 | + | TAATTTTAAATAATT |
| P. infestans | PITG\_01330 | 1000 | Motif-0 | -404 | - | TCAGTCCTCACTCGCC |
| P. infestans | PITG\_01332 | 1000 | Motif-1 | -898 | + | TACATGTAT |
| P. infestans | PITG\_01333 | 1000 | Motif-1 | -193 | + | AACATGTAC |
| P. infestans | PITG\_01335 | 357 | Motif-0 | -60 | + | GCATTCCCCAATTTCT |
| P. infestans | PITG\_01336 | 357 | Motif-0 | -313 | - | GCATTCCCCAATTTCT |
| P. infestans | PITG\_01342 | 761 | Motif-0 | -44 | + | CCATTGCGGGATTTGC |
|  |  |  | Motif-1 | -254 | + | TGCATGTATCAATGA |
|  |  |  | Motif-2 | -108 | + | ACAACTTCAAGGAGGC |
|  |  |  | Motif-8 | -153 | + | TTTTTTTTATCTAAT |
| P. infestans | PITG\_01343 | 761 | Motif-0 | -733 | - | CCATTGCGGGATTTGC |
|  |  |  | Motif-1 | -414 | + | TACATGTAT |
|  |  |  | Motif-2 | -669 | - | ACAACTTCAAGGAGGC |
|  |  |  | Motif-8 | -408 | + | TATGTATTAATAAAA |
| P. infestans | PITG\_01345 | 1000 | Motif-18 | -346 | - | CATTGTGGTGTACATG |
|  |  |  | Motif-4 | -409 | + | CTTGATTGGCTGTAA |
| P. infestans | PITG\_01348 | 1000 | Motif-2 | -936 | + | CAAATTGAAGTT |
| P. infestans | PITG\_01349 | 1000 | Motif-0 | -111 | - | GAAATTTCGAGAATGA |
|  |  |  | Motif-4 | -135 | - | TATGATTGGTTGAAA |
| P. infestans | PITG\_01350 | 778 | Motif-0 | -28 | - | GCTAATTGGCGAATGC |
| P. infestans | PITG\_01351 | 778 | Motif-0 | -504 | + | CCATTCTTCAAAGTGC |
| P. infestans | PITG\_01353 | 226 | Motif-0 | -214 | - | GCATTCGTCAATCAGC |
|  |  |  | Motif-18 | -181 | + | TCGTGTGGTACACATA |
| P. infestans | PITG\_01354 | 226 | Motif-0 | -28 | + | GCATTCGTCAATCAGC |
|  |  |  | Motif-18 | -61 | - | TCGTGTGGTACACATA |
| P. infestans | PITG\_01356 | 1000 | Motif-0 | -333 | - | TCACTTATGGATTCTC |
|  |  |  | Motif-2 | -92 | + | ACTTCAACATC |
|  |  |  | Motif-8 | -291 | - | TTTTATTTAATATAT |
| P. infestans | PITG\_01357 | 1000 | Motif-0 | -37 | - | GCAAATTGACGAGTGG |
|  |  |  | Motif-1 | -269 | - | ACTACCGGTACTATA |
|  |  |  | Motif-2 | -94 | + | CCATTTCAACATCC |
| P. infestans | PITG\_01358 | 1000 | Motif-0 | -505 | - | GAACTCTTCAATTCGC |
|  |  |  | Motif-1 | -230 | + | AACATGTAC |
|  |  |  | Motif-18 | -185 | - | TTGTGTGATGCACATG |
|  |  |  | Motif-6 | -40 | + | CGCCCCCCCCCCTCTC |
|  |  |  | Motif-9 | -353 | - | CAGTACCGGTA |
| P. infestans | PITG\_01360 | 803 | Motif-0 | -37 | + | TCATTCGCATTTCTCC |
|  |  |  | Motif-1 | -439 | + | TACATGTAT |
|  |  |  | Motif-2 | -120 | - | CACTTCAACAT |
| P. infestans | PITG\_01361 | 803 | Motif-0 | -624 | - | TCACTTTCCAGCTTT |
|  |  |  | Motif-1 | -357 | - | TACATATAC |
|  |  |  | Motif-2 | -693 | + | ACTTCAACATG |
| P. infestans | PITG\_01362 | 995 | Motif-0 | -122 | + | GCAGAAGTCAGAATGA |
|  |  |  | Motif-18 | -762 | + | TATTGTGGTGAACAAG |
|  |  |  | Motif-2 | -903 | - | TAACTTCATCCTC |
| P. infestans | PITG\_01363 | 1000 | Motif-0 | -63 | - | GGAAGTTCAGGAATGA |
|  |  |  | Motif-1 | -304 | + | TACATGTAG |
|  |  |  | Motif-4 | -499 | - | TCCGATTGGCCGGAA |
|  |  |  | Motif-7 | -388 | + | TAGTATTAATA |
| P. infestans | PITG\_01365 | 1000 | Motif-4 | -887 | - | GTTTATTGGTCAATA |
| P. infestans | PITG\_01369 | 1000 | Motif-0 | -48 | - | GCAAATTGAGGAATGG |
| P. infestans | PITG\_01372 | 1000 | Motif-1 | -959 | - | TACGTGTAC |
| P. infestans | PITG\_01374 | 1000 | Motif-16 | -926 | - | CAGCAGTCGCAGGAAC |
| P. infestans | PITG\_01375 | 1000 | Motif-1 | -150 | - | ATTACCTGTATAATA |
|  |  |  | Motif-16 | -730 | + | CAGCAGTCGCAGGAAC |
| P. infestans | PITG\_01386 | 1000 | Motif-1 | -178 | + | TACATGTAC |
|  |  |  | Motif-18 | -857 | - | TATTGTGGCTTACACC |
|  |  |  | Motif-4 | -339 | - | GTCGATTCGCTAAAA |
|  |  |  | Motif-6 | -27 | + | CGCCCCCCCCTCTCCC |
|  |  |  | Motif-7 | -818 | - | TATTATTAATA |
|  |  |  | Motif-8 | -818 | - | TTATTATTATTAATA |
| P. infestans | PITG\_01388 | 1000 | Motif-1 | -169 | + | TACATGTAT |
| P. infestans | PITG\_01390 | 660 | Motif-0 | -461 | - | TCATATTGGAACTCAC |
|  |  |  | Motif-1 | -155 | + | AACATGTATCATCAT |
| P. infestans | PITG\_01391 | 660 | Motif-0 | -215 | + | TCATATTGGAACTCAC |
|  |  |  | Motif-1 | -271 | + | TGCATGTATAGTAGT |
| P. infestans | PITG\_01395 | 1000 | Motif-0 | -66 | + | CATTCCGCCATTTGCC |
|  |  |  | Motif-1 | -295 | + | TACATGTAG |
| P. infestans | PITG\_01396 | 1000 | Motif-0 | -779 | + | TTACTTGCAAATTTCA |
|  |  |  | Motif-1 | -433 | + | TACATGTAA |
|  |  |  | Motif-2 | -277 | + | ATGGTTGAAGTG |
|  |  |  | Motif-3 | -822 | + | TAAAAAATCCAAATCA |
|  |  |  | Motif-4 | -519 | - | TATGATTGGTAGAAA |
| P. infestans | PITG\_01397 | 1000 | Motif-0 | -21 | - | GCGAATCTAAAAGTGA |
|  |  |  | Motif-1 | -168 | + | TACCTGTATCTCTGT |
| P. infestans | PITG\_01398 | 354 | Motif-0 | -139 | - | TCAAGTTGGGAAATGA |
|  |  |  | Motif-2 | -229 | + | GTAGTTGAAGTT |
| P. infestans | PITG\_01399 | 354 | Motif-0 | -36 | - | GCGAATCGGATAATGA |
|  |  |  | Motif-2 | -137 | - | GTAGTTGAAGTT |
| P. infestans | PITG\_01400 | 1000 | Motif-0 | -478 | + | CCAGTCTTCAATTAAC |
|  |  |  | Motif-1 | -957 | + | TACATGTAT |
|  |  |  | Motif-3 | -92 | + | TACAAAAAGCAAGACA |
|  |  |  | Motif-4 | -193 | - | TCTGATTGGATGATA |
| P. infestans | PITG\_01401 | 1000 | Motif-0 | -123 | + | CCAGACTCGAATTTAC |
|  |  |  | Motif-4 | -140 | - | TGGGATTGGCCGAAA |
| P. infestans | PITG\_01402 | 624 | Motif-1 | -375 | + | TACATGTAC |
|  |  |  | Motif-3 | -162 | - | ACTTGATTTAAGTAA |
| P. infestans | PITG\_01403 | 719 | Motif-1 | -354 | + | TACATGTAA |
|  |  |  | Motif-18 | -686 | - | TGGTGTTCTGTACACA |
|  |  |  | Motif-2 | -321 | + | ACTTCAATATCTACAA |
| P. infestans | PITG\_01404 | 719 | Motif-1 | -373 | + | TACATGTAC |
|  |  |  | Motif-18 | -49 | + | TGGTGTTCTGTACACA |
|  |  |  | Motif-2 | -150 | + | ACTGCAACGCG |
| P. infestans | PITG\_01405 | 65 | Motif-4 | -57 | - | TTTTATTGGTCAAAA |
| P. infestans | PITG\_01406 | 1000 | Motif-1 | -845 | + | GCAACATGTAGCTTA |
|  |  |  | Motif-17 | -107 | - | GCTGTAGCCATGCGA |
|  |  |  | Motif-2 | -848 | + | GCTGCAACATG |
| P. infestans | PITG\_01407 | 1000 | Motif-0 | -56 | - | GCAAAAATGAGAATGA |
|  |  |  | Motif-1 | -399 | + | TACATGTAG |
|  |  |  | Motif-2 | -80 | - | GACTTCAAATTGAC |
|  |  |  | Motif-7 | -408 | - | TAGTATTAATA |
|  |  |  | Motif-9 | -545 | - | CCGTACCGGTA |
| P. infestans | PITG\_01408 | 366 | Motif-0 | -27 | + | TCACTTCTGCTCTCCC |
| P. infestans | PITG\_01409 | 366 | Motif-0 | -329 | + | GCAAAATCAGGAATGA |
| P. infestans | PITG\_01410 | 1000 | Motif-1 | -866 | + | TACACGTACAACAGT |
| P. infestans | PITG\_01411 | 1000 | Motif-1 | -435 | - | AGTAAATGTAC |
| P. infestans | PITG\_01412 | 305 | Motif-2 | -133 | - | CAGCTTCAACAAA |
| P. infestans | PITG\_01415 | 576 | Motif-2 | -258 | + | TAAACTTCAAGTAGCA |
| P. infestans | PITG\_01416 | 860 | Motif-0 | -335 | - | TCATTGTGGAATTTAA |
|  |  |  | Motif-2 | -64 | - | AGATACGCTGAAGTGA |
|  |  |  | Motif-4 | -206 | + | TATTATTGGCTGCAA |
| P. infestans | PITG\_01417 | 576 | Motif-2 | -334 | - | TAAACTTCAAGTAGCA |
| P. infestans | PITG\_01418 | 860 | Motif-0 | -541 | + | TCATTGTGGAATTTAA |
|  |  |  | Motif-2 | -598 | + | CACCTTGAAGTT |
|  |  |  | Motif-4 | -669 | - | TATTATTGGCTGCAA |
| P. infestans | PITG\_01419 | 300 | Motif-0 | -49 | - | GCAGATTGGCGAGTGA |
|  |  |  | Motif-3 | -144 | - | GACGTGGATTTTGTTT |
| P. infestans | PITG\_01421 | 1000 | Motif-1 | -829 | + | AACATGTATTATTAA |
|  |  |  | Motif-9 | -491 | + | GCGTACCGGTA |
| P. infestans | PITG\_01423 | 1000 | Motif-0 | -55 | + | CCATTCCAGCATTCGC |
| P. infestans | PITG\_01428 | 338 | Motif-0 | -327 | + | GCGGAATGCCAAATGA |
| P. infestans | PITG\_01429 | 338 | Motif-0 | -27 | - | GCGGAATGCCAAATGA |
| P. infestans | PITG\_01430 | 202 | Motif-0 | -37 | - | TAAAGTTGGAGAATGA |
|  |  |  | Motif-2 | -140 | + | ACTTCAATATCTTCAA |
| P. infestans | PITG\_01431 | 1000 | Motif-1 | -29 | + | TACATGTAT |
| P. infestans | PITG\_01434 | 362 | Motif-17 | -46 | + | TCTGTACCCATTTTG |
| P. infestans | PITG\_01435 | 362 | Motif-17 | -331 | - | TCTGTACCCATTTTG |
| P. infestans | PITG\_01438 | 634 | Motif-3 | -94 | + | ACTTGTATTTTGTAA |
| P. infestans | PITG\_01439 | 313 | Motif-8 | -219 | + | TATAATTTATTAATA |
| P. infestans | PITG\_01440 | 313 | Motif-8 | -109 | - | TATAATTTATTAATA |
| P. infestans | PITG\_01441 | 1000 | Motif-1 | -862 | + | AGAACCTGTACAGTT |
|  |  |  | Motif-2 | -26 | + | AAGGTTGAAGGTGA |
| P. infestans | PITG\_01445 | 739 | Motif-0 | -701 | - | TCACTTCGCAAGTGCC |
|  |  |  | Motif-1 | -517 | - | TACGTGTAC |
|  |  |  | Motif-6 | -184 | - | TGCCCCCCCCCCCCAA |
| P. infestans | PITG\_01446 | 739 | Motif-0 | -54 | + | TCACTTCGCAAGTGCC |
|  |  |  | Motif-1 | -225 | + | TACATGTTTTAATTT |
|  |  |  | Motif-6 | -569 | + | CCCCCCCCCCCCAAGA |
| P. infestans | PITG\_01447 | 1000 | Motif-1 | -312 | - | CACACATGTACAATG |
|  |  |  | Motif-8 | -120 | - | TACTATTAAATATTT |
| P. infestans | PITG\_01451 | 201 | Motif-4 | -37 | + | TGCGATTGGACAAAA |
| P. infestans | PITG\_01452 | 714 | Motif-0 | -73 | - | GAAAGTGGCAAAATGA |
|  |  |  | Motif-16 | -468 | - | CGGACGCAGCAGCAGC |
|  |  |  | Motif-6 | -334 | - | ACCCCGCCCTCCGCCC |
| P. infestans | PITG\_01453 | 714 | Motif-0 | -657 | + | GAAAGTGGCAAAATGA |
|  |  |  | Motif-16 | -136 | + | CGACAGCAGCAGTAGC |
|  |  |  | Motif-6 | -396 | + | ACCCCGCCCTCCGCCC |
| P. infestans | PITG\_01454 | 1000 | Motif-0 | -156 | + | GCACTTTTGGACTCGC |
|  |  |  | Motif-3 | -614 | + | ACTTGAAGTATGTAT |
|  |  |  | Motif-6 | -172 | + | CCCCCTCCCCCCATCT |
|  |  |  | Motif-9 | -632 | + | ACGTACCGGTA |
| P. infestans | PITG\_01455 | 1000 | Motif-0 | -206 | + | CACTTTTGGATTCGCC |
| P. infestans | PITG\_01456 | 175 | Motif-8 | -98 | + | TTTATTTAAATTTCA |
| P. infestans | PITG\_01457 | 175 | Motif-8 | -92 | - | TTTATTTAAATTTCA |
| P. infestans | PITG\_01458 | 1000 | Motif-1 | -225 | + | TACATGTAC |
|  |  |  | Motif-2 | -100 | + | TGTGAAGCTGAAGTGC |
|  |  |  | Motif-4 | -121 | - | GCTGATTGGTCAAGA |
|  |  |  | Motif-7 | -47 | + | TATTATTAATA |
|  |  |  | Motif-8 | -57 | - | TAATATTAATTTAAT |
|  |  |  | Motif-9 | -409 | - | CAGTACCGGTA |
| P. infestans | PITG\_01461 | 1000 | Motif-0 | -381 | - | TCACTTTCAAATCTT |
| P. infestans | PITG\_01462 | 1000 | Motif-0 | -53 | + | CCAGTCGCCAATTCTC |
|  |  |  | Motif-2 | -89 | + | CAACTTGAAGTG |
|  |  |  | Motif-3 | -130 | - | ACTTGATTTCAGTAA |
| P. infestans | PITG\_01463 | 1000 | Motif-1 | -644 | - | GCCACATGTACCATA |
| P. infestans | PITG\_01464 | 1000 | Motif-0 | -117 | - | GCAGATTTGAGAGTGA |
|  |  |  | Motif-1 | -506 | + | TACATATAC |
|  |  |  | Motif-3 | -203 | + | TCTTGATTTTTGTAT |
| P. infestans | PITG\_01465 | 1000 | Motif-0 | -266 | + | TGAAATCCGAAAGTGA |
|  |  |  | Motif-3 | -110 | + | ACTTGCGTTTTGTAT |
| P. infestans | PITG\_01468 | 1000 | Motif-0 | -75 | - | GGTAAATGAGAAGTGA |
|  |  |  | Motif-1 | -455 | - | AGTGTACGTGTATG |
|  |  |  | Motif-2 | -135 | - | GAAGTTGAAGCG |
|  |  |  | Motif-4 | -163 | + | TGCGATTGGCTGAAA |
| P. infestans | PITG\_01470 | 385 | Motif-18 | -163 | - | TTGTGTGGTACACATA |
|  |  |  | Motif-2 | -193 | - | CTGGTTGAAGTG |
| P. infestans | PITG\_01471 | 385 | Motif-18 | -238 | + | TTGTGTGGTACACATA |
|  |  |  | Motif-2 | -204 | + | CTGGTTGAAGTG |
| P. infestans | PITG\_01476 | 1000 | Motif-8 | -263 | + | TTTTTTTATTTTGAA |
|  |  |  | Motif-9 | -214 | - | CGGTACCGGTA |
| P. infestans | PITG\_01477 | 575 | Motif-4 | -238 | - | TGTGATTGGCTGGAT |
| P. infestans | PITG\_01479 | 245 | Motif-0 | -134 | - | TCATTCCTGAATCCGG |
| P. infestans | PITG\_01480 | 245 | Motif-0 | -49 | + | CCAGTCTGCATCCTGC |
| P. infestans | PITG\_01484 | 1000 | Motif-0 | -57 | + | CCAGTCGCCAATTCTC |
|  |  |  | Motif-2 | -103 | + | CAACTTGAAGTG |
|  |  |  | Motif-3 | -144 | - | ACTTGATTTCAGTAA |
| P. infestans | PITG\_01485 | 1000 | Motif-0 | -117 | - | GCAGATTTGAGAGTGA |
|  |  |  | Motif-1 | -493 | + | TACATATAC |
|  |  |  | Motif-3 | -203 | + | TCTTGATTTTTGTAT |
| P. infestans | PITG\_01486 | 1000 | Motif-0 | -190 | - | ACACTTTCGGATTTCA |
|  |  |  | Motif-3 | -26 | + | ACTTGAGTTTTGTAT |
| P. infestans | PITG\_01487 | 1000 | Motif-0 | -572 | - | TCATTATCCTACTCCC |
|  |  |  | Motif-1 | -545 | + | TACATGTAA |
|  |  |  | Motif-2 | -522 | + | ACTTCAACTTG |
| P. infestans | PITG\_01488 | 1000 | Motif-2 | -29 | + | AAAACTTCAAGATCCA |
| P. infestans | PITG\_01489 | 1000 | Motif-0 | -697 | - | GCAATTTCAAAAGTGC |
|  |  |  | Motif-8 | -313 | + | TACATTTTATTAAAA |
| P. infestans | PITG\_01490 | 1000 | Motif-1 | -785 | + | AATAAATGTACTTTA |
|  |  |  | Motif-7 | -956 | - | CACTATTAATA |
|  |  |  | Motif-8 | -281 | - | TTTTTTTTTGTTTTA |
| P. infestans | PITG\_01491 | 874 | Motif-16 | -827 | - | CAGCACCAGCAGTAAC |
| P. infestans | PITG\_01496 | 1000 | Motif-0 | -736 | - | CCACTTTCACATCTCC |
| P. infestans | PITG\_01499 | 1000 | Motif-4 | -209 | - | CGTGATTGGTTGATA |
|  |  |  | Motif-7 | -61 | - | AAGTATTAATA |
| P. infestans | PITG\_01504 | 1000 | Motif-0 | -869 | + | TCAGTCGTGAATTTCT |
|  |  |  | Motif-1 | -424 | + | TACATGAAC |
|  |  |  | Motif-4 | -533 | + | GGTGATTGGTGAAAA |
|  |  |  | Motif-6 | -121 | + | CCCCCCCCCCAGTCAC |
| P. infestans | PITG\_01507 | 1000 | Motif-0 | -49 | + | TCATTCTCGATTCGGT |
| P. infestans | PITG\_01508 | 1000 | Motif-16 | -48 | + | CAACAGCAAGAGCAAC |
|  |  |  | Motif-6 | -71 | + | TCCTCCCCCCACACCC |
| P. infestans | PITG\_01510 | 1000 | Motif-2 | -310 | + | TCATCTTCAATTTCAA |
|  |  |  | Motif-3 | -260 | + | TACAATACGCAAGTCA |
| P. infestans | PITG\_01511 | 1000 | Motif-0 | -201 | - | TTATTTTTAAATTAG |
|  |  |  | Motif-2 | -308 | - | CCCTTCAACGT |
|  |  |  | Motif-4 | -931 | - | ATTGATTGGCTGAAA |
|  |  |  | Motif-8 | -54 | + | TTTTTTTCAATAAAA |
| P. infestans | PITG\_01515 | 1000 | Motif-3 | -490 | + | AACTTTGCATTTGTAT |
| P. infestans | PITG\_01516 | 1000 | Motif-3 | -161 | + | TACAAATATAAAGTCA |
|  |  |  | Motif-7 | -30 | + | CATTATTAATA |
| P. infestans | PITG\_01517 | 1000 | Motif-0 | -863 | + | ACATTTTTACATTTAC |
| P. infestans | PITG\_01519 | 1000 | Motif-0 | -305 | + | GAAAAATTAGGAATGG |
|  |  |  | Motif-16 | -343 | + | CAGCAGCTGCAGCGAC |
| P. infestans | PITG\_01523 | 1000 | Motif-0 | -65 | - | GAAAATCGCAGAGTGG |
| P. infestans | PITG\_01524 | 1000 | Motif-8 | -401 | - | TTTTTTTTAATTTTT |
|  |  |  | Motif-9 | -120 | - | CAGTACCGGTA |
| P. infestans | PITG\_01525 | 1000 | Motif-1 | -79 | - | CATACATGTCGCATA |
|  |  |  | Motif-3 | -100 | - | ATTTGTTTTTTCTAT |
| P. infestans | PITG\_01527 | 1000 | Motif-1 | -663 | - | CCTAAATGTAT |
|  |  |  | Motif-4 | -493 | + | TGTGATTGGTCAATG |
| P. infestans | PITG\_01529 | 1000 | Motif-8 | -227 | - | TTTTTTTTAACTTAA |
| P. infestans | PITG\_01534 | 1000 | Motif-1 | -888 | + | TACCTGTAGTGATCT |
|  |  |  | Motif-3 | -173 | + | ACTGAGTGTTTGTAA |
|  |  |  | Motif-8 | -443 | + | TTTAATTTATTTTTT |
| P. infestans | PITG\_01535 | 1000 | Motif-1 | -542 | - | TACATGTGCA |
|  |  |  | Motif-3 | -171 | - | ACTTAAGGTTTGTAA |
|  |  |  | Motif-8 | -672 | + | TTTTTTTAAATTGCA |
|  |  |  | Motif-9 | -123 | - | CAGTACCGGTA |
| P. infestans | PITG\_01537 | 1000 | Motif-0 | -147 | - | TTATTTTACAATTTT |
|  |  |  | Motif-1 | -977 | + | ACTGCATGTAAAGCTG |
|  |  |  | Motif-3 | -81 | - | TACATTAACCAAGTCA |
|  |  |  | Motif-4 | -217 | - | GGGGATTGGCCAAAA |
|  |  |  | Motif-8 | -160 | + | TAATATTTAATTAAA |
| P. infestans | PITG\_01538 | 1000 | Motif-4 | -68 | + | TCTGATTGGTTATTT |
|  |  |  | Motif-8 | -812 | + | TATTATTAAATATCA |
| P. infestans | PITG\_01543 | 1000 | Motif-3 | -432 | + | ACTTAGCGATTCTAT |
|  |  |  | Motif-4 | -270 | + | GCTGATTGGCTGTTT |
|  |  |  | Motif-6 | -647 | + | CCCCCCCCCCCTTCAT |
| P. infestans | PITG\_01546 | 308 | Motif-0 | -72 | + | CCATACCTCAATTCGC |
| P. infestans | PITG\_01547 | 308 | Motif-0 | -252 | - | CCATACCTCAATTCGC |
| P. infestans | PITG\_01549 | 1000 | Motif-0 | -47 | + | CAGTTCCTAATTTGCT |
|  |  |  | Motif-1 | -338 | + | TACATGTAC |
|  |  |  | Motif-16 | -302 | - | GAATAGTAGCCCCGGC |
|  |  |  | Motif-3 | -761 | + | ATTTGAAATTTGTAT |
| P. infestans | PITG\_01551 | 1000 | Motif-3 | -493 | + | TACAAACTTTAAGTCA |
|  |  |  | Motif-9 | -153 | + | AAGTACCGGTA |
| P. infestans | PITG\_01553 | 1000 | Motif-1 | -210 | + | TACATGTAC |
|  |  |  | Motif-2 | -347 | - | GAAGTTCAAGTTGC |
|  |  |  | Motif-4 | -378 | + | TTTGATTGGTAGTAA |
| P. infestans | PITG\_01554 | 1000 | Motif-0 | -40 | - | GAGAATTGCTAAATGA |
| P. infestans | PITG\_01555 | 1000 | Motif-1 | -99 | - | TGCATGTATTTAAGT |
| P. infestans | PITG\_01557 | 1000 | Motif-7 | -809 | - | TACTATTAATA |
| P. infestans | PITG\_01558 | 1000 | Motif-1 | -975 | + | TACATGCATA |
| P. infestans | PITG\_01559 | 1000 | Motif-1 | -48 | - | AGTACAGGTAC |
| P. infestans | PITG\_01560 | 1000 | Motif-4 | -91 | - | TGGGATTGGTCATAA |
|  |  |  | Motif-7 | -438 | - | TAGTATTAATA |
| P. infestans | PITG\_01562 | 1000 | Motif-4 | -696 | + | TTCTAGCTAATGAAA |
| P. infestans | PITG\_01563 | 1000 | Motif-17 | -772 | + | AGTGTACGCATCTTG |
|  |  |  | Motif-8 | -333 | + | TATTTTTATTTTACA |
| P. infestans | PITG\_01564 | 1000 | Motif-0 | -69 | + | CACATTCGCATTCGCC |
|  |  |  | Motif-2 | -449 | + | AACGGCAACATGGA |
| P. infestans | PITG\_01569 | 1000 | Motif-0 | -289 | - | TCAGTTTTGCTTCGAC |
|  |  |  | Motif-1 | -338 | + | TACATGAAACTGGTAC |
|  |  |  | Motif-8 | -698 | + | TTTTTGTAAATAAAA |
| P. infestans | PITG\_01576 | 77 | Motif-4 | -41 | - | TGTCATTGGTCAAAA |
| P. infestans | PITG\_01577 | 77 | Motif-4 | -51 | + | TGTCATTGGTCAAAA |
| P. infestans | PITG\_01578 | 1000 | Motif-2 | -470 | + | GACCTTGAAGTG |
|  |  |  | Motif-3 | -29 | + | TATAAAAAGCCAGTCA |
| P. infestans | PITG\_01579 | 246 | Motif-1 | -135 | - | AACATGTAC |
| P. infestans | PITG\_01580 | 246 | Motif-1 | -114 | + | TACATGTAA |
| P. infestans | PITG\_01581 | 326 | Motif-0 | -191 | - | CCATTTTACGATTCGA |
|  |  |  | Motif-18 | -53 | + | TGGAGTGGTGAACACA |
|  |  |  | Motif-6 | -262 | + | TCCTCCTCCCCCTCCC |
|  |  |  | Motif-7 | -209 | - | AAGTATTAATA |
| P. infestans | PITG\_01582 | 326 | Motif-0 | -151 | + | CCATTTTACGATTCGA |
|  |  |  | Motif-18 | -289 | - | TGGAGTGGTGAACACA |
|  |  |  | Motif-6 | -77 | - | TCCTCCTCCTCCCCCT |
|  |  |  | Motif-7 | -125 | - | AAATATTAATA |
| P. infestans | PITG\_01584 | 1000 | Motif-1 | -484 | - | AATACCGGTACCGTA |
|  |  |  | Motif-8 | -25 | + | TTTGTTTAAATAATA |
|  |  |  | Motif-9 | -482 | + | CGGTACCGGTA |
| P. infestans | PITG\_01588 | 1000 | Motif-1 | -676 | - | TACGTGTAC |
|  |  |  | Motif-8 | -74 | - | TTTTTTTTTTTAAAA |
| P. infestans | PITG\_01590 | 236 | Motif-0 | -27 | + | CACTTTTGCATTCGAC |
| P. infestans | PITG\_01592 | 1000 | Motif-2 | -476 | - | GACTTCAACCT |
|  |  |  | Motif-3 | -226 | + | TACAAAATTCAAGTTA |
| P. infestans | PITG\_01593 | 1000 | Motif-6 | -239 | + | CCCCCCCCCTCCCCGT |
|  |  |  | Motif-8 | -175 | + | TTTTATTAATTTGTA |
| P. infestans | PITG\_01594 | 1000 | Motif-0 | -761 | - | CTCACTTCTTCTTTT |
| P. infestans | PITG\_01598 | 1000 | Motif-0 | -88 | + | TCATTGGTCAACTCAC |
|  |  |  | Motif-1 | -472 | + | TACCTGTAGAAGTCC |
|  |  |  | Motif-4 | -90 | + | TATCATTGGTCAACT |
|  |  |  | Motif-9 | -620 | + | CTGTACCGGTA |
| P. infestans | PITG\_01599 | 1000 | Motif-0 | -811 | + | TCAGTTTTGCTTCTAC |
|  |  |  | Motif-8 | -659 | - | TTATTTTAAATTTAA |
| P. infestans | PITG\_01602 | 1000 | Motif-0 | -614 | + | TCATTTTGATATTTCA |
|  |  |  | Motif-1 | -249 | + | TACATGTAG |
| P. infestans | PITG\_01603 | 162 | Motif-4 | -73 | - | TGTGATTGGCTGAAA |
| P. infestans | PITG\_01604 | 162 | Motif-4 | -104 | + | TGTGATTGGCTGAAA |
| P. infestans | PITG\_01605 | 1000 | Motif-0 | -37 | - | GAGAATTCTAAACTGA |
|  |  |  | Motif-1 | -112 | + | TACATGTAG |
|  |  |  | Motif-6 | -176 | + | TGCCCCCCCCCGTACA |
| P. infestans | PITG\_01608 | 1000 | Motif-1 | -542 | + | TACATGTAC |
|  |  |  | Motif-3 | -85 | - | TCTTGAAATCAGTAT |
|  |  |  | Motif-7 | -512 | - | TATTATTAATA |
| P. infestans | PITG\_01610 | 1000 | Motif-8 | -143 | - | TAATTTTAATCAAAA |
| P. infestans | PITG\_01614 | 1000 | Motif-0 | -861 | + | GCCGATTCGAAAATGG |
|  |  |  | Motif-1 | -167 | + | TACATGCAC |
|  |  |  | Motif-4 | -494 | - | GCTGATTGGACAAAA |
|  |  |  | Motif-6 | -790 | - | ACCCCTACCCCCACCC |
| P. infestans | PITG\_01615 | 443 | Motif-2 | -37 | + | TCGCTTCAAGCTGC |
| P. infestans | PITG\_01616 | 443 | Motif-2 | -420 | - | TCGCTTCAAGCTGC |
| P. infestans | PITG\_01617 | 891 | Motif-2 | -792 | + | CGAGTTCAACATCG |
| P. infestans | PITG\_01618 | 1000 | Motif-1 | -599 | - | AATACCGGTACAGTA |
|  |  |  | Motif-9 | -597 | + | CTGTACCGGTA |
| P. infestans | PITG\_01621 | 268 | Motif-0 | -226 | + | GCGGAATGCAGAGTGA |
| P. infestans | PITG\_01622 | 268 | Motif-0 | -58 | - | GCGGAATGCAGAGTGA |
| P. infestans | PITG\_01623 | 736 | Motif-0 | -171 | - | CCACTTCTTGACTTGA |
| P. infestans | PITG\_01624 | 1000 | Motif-1 | -13 | - | CCTAAATGTAT |
| P. infestans | PITG\_01625 | 1000 | Motif-0 | -83 | + | CACTTTGCAATCCGCC |
|  |  |  | Motif-1 | -571 | + | AGAACATGTAGTTAA |
|  |  |  | Motif-6 | -633 | - | AGCCCCCCCCCCTCCT |
| P. infestans | PITG\_01626 | 1000 | Motif-0 | -48 | + | TCAGTCCCCTTTCGTC |
|  |  |  | Motif-1 | -376 | + | TACGTGTAC |
|  |  |  | Motif-2 | -469 | + | CGTGTTGAAGTG |
|  |  |  | Motif-3 | -125 | - | AACTTGTTTTTTGTAT |
|  |  |  | Motif-4 | -950 | + | TCCCATTGGCTGATA |
| P. infestans | PITG\_01627 | 1000 | Motif-0 | -34 | + | CACAATCGAATTTGCC |
|  |  |  | Motif-1 | -252 | + | TACATGTAC |
|  |  |  | Motif-16 | -285 | + | CAGCAGGAACAGCAAC |
|  |  |  | Motif-2 | -95 | + | ACTTCAACTTG |
|  |  |  | Motif-7 | -596 | + | CATTATTAATA |
| P. infestans | PITG\_01628 | 1000 | Motif-7 | -102 | - | AAATATTAATA |
|  |  |  | Motif-8 | -30 | + | TTTTACTTAGTATAT |
| P. infestans | PITG\_01633 | 1000 | Motif-1 | -894 | + | TACATGTAA |
| P. infestans | PITG\_01637 | 1000 | Motif-8 | -773 | + | TATAAATAAATATAT |
| P. infestans | PITG\_01638 | 1000 | Motif-0 | -897 | - | TCACTTCTCATTTCT |
|  |  |  | Motif-2 | -798 | - | GTAGTTCAACTTCA |
| P. infestans | PITG\_01639 | 1000 | Motif-0 | -100 | + | TCATTTCCCAATATAT |
|  |  |  | Motif-1 | -272 | + | TACATGTAT |
| P. infestans | PITG\_01640 | 701 | Motif-1 | -646 | + | TACATGTAC |
|  |  |  | Motif-8 | -416 | + | TTTTTTTAATTTTAA |
| P. infestans | PITG\_01642 | 1000 | Motif-0 | -55 | - | GAAGGTTCGGGAATGA |
| P. infestans | PITG\_01644 | 415 | Motif-17 | -337 | + | GGTGTAGCCATTTCT |
|  |  |  | Motif-4 | -386 | + | GCTCATTGGCTGGAT |
| P. infestans | PITG\_01645 | 415 | Motif-17 | -93 | - | GGTGTAGCCATTTCT |
|  |  |  | Motif-4 | -44 | - | GCTCATTGGCTGGAT |
| P. infestans | PITG\_01647 | 1000 | Motif-0 | -288 | + | GCCAATTTGGAAATGG |
|  |  |  | Motif-1 | -169 | + | TACATGTAC |
| P. infestans | PITG\_01651 | 506 | Motif-2 | -499 | - | ACTTCAAGTTG |
| P. infestans | PITG\_01652 | 258 | Motif-4 | -32 | + | TATGATTGGCAGATT |
| P. infestans | PITG\_01653 | 258 | Motif-4 | -61 | + | GGTGATTGGTCAGAT |
| P. infestans | PITG\_01654 | 416 | Motif-1 | -294 | + | TACATGTAC |
| P. infestans | PITG\_01655 | 1000 | Motif-1 | -322 | + | TACATGTAC |
|  |  |  | Motif-9 | -524 | - | CAGTACCGGTA |
| P. infestans | PITG\_01658 | 1000 | Motif-2 | -673 | - | GCTGTTGAAGTTAG |
| P. infestans | PITG\_01659 | 1000 | Motif-0 | -182 | - | TCCGTCTGTAATTTGC |
|  |  |  | Motif-1 | -399 | + | AACATGTAC |
|  |  |  | Motif-7 | -304 | - | TATTATTAATA |
| P. infestans | PITG\_01662 | 1000 | Motif-0 | -35 | + | CATTCCGTAATTCGCT |
| P. infestans | PITG\_01663 | 387 | Motif-1 | -246 | - | TCTACCGGTACATGAA |
|  |  |  | Motif-4 | -105 | + | TGCCATTGGTCAACT |
|  |  |  | Motif-9 | -243 | + | ATGTACCGGTA |
| P. infestans | PITG\_01664 | 616 | Motif-1 | -261 | + | TACATGTAC |
|  |  |  | Motif-7 | -245 | - | TAATATTAATA |
| P. infestans | PITG\_01665 | 616 | Motif-1 | -363 | + | TACATGTAC |
|  |  |  | Motif-7 | -382 | + | TAATATTAATA |
| P. infestans | PITG\_01671 | 1000 | Motif-1 | -168 | + | AGCATGTATTATAAT |
|  |  |  | Motif-16 | -885 | - | CACCAGTAGCAGCAGC |
| P. infestans | PITG\_01672 | 1000 | Motif-0 | -57 | - | GCACGTTTGGAAATGA |
|  |  |  | Motif-1 | -348 | + | TACATGTAC |
| P. infestans | PITG\_01673 | 875 | Motif-1 | -642 | - | TACATATAC |
|  |  |  | Motif-2 | -199 | + | ACTTCAACTTC |
|  |  |  | Motif-4 | -183 | - | TTTGATTGGCTGACT |
| P. infestans | PITG\_01674 | 1000 | Motif-1 | -27 | + | TACATGTAA |
| P. infestans | PITG\_01676 | 1000 | Motif-1 | -368 | + | TGCATGTATCCATTT |
|  |  |  | Motif-8 | -780 | - | TTATATTTATTAAGA |
| P. infestans | PITG\_01677 | 1000 | Motif-1 | -481 | + | TACATGTAT |
|  |  |  | Motif-8 | -336 | + | TTATATTTATTAAGA |
| P. infestans | PITG\_01678 | 89 | Motif-4 | -59 | - | TCTGATTTGTTGAAA |
| P. infestans | PITG\_01679 | 89 | Motif-4 | -45 | + | TCTGATTTGTTGAAA |
| P. infestans | PITG\_01681 | 690 | Motif-1 | -574 | + | TACATGTAG |
|  |  |  | Motif-2 | -460 | + | CCATCAACGTGAGTCA |
| P. infestans | PITG\_01682 | 223 | Motif-0 | -209 | - | TCACACGCAAACTCGC |
|  |  |  | Motif-2 | -166 | + | ACTTCAACACG |
| P. infestans | PITG\_01683 | 1000 | Motif-0 | -420 | + | GAAAGTTCGCGAGTGA |
|  |  |  | Motif-2 | -439 | + | ATCACTTCAATTCCTC |
| P. infestans | PITG\_01689 | 1000 | Motif-4 | -737 | + | TTTGATTGGTCAATA |
|  |  |  | Motif-7 | -153 | + | TACTATTAATA |
| P. infestans | PITG\_01695 | 1000 | Motif-1 | -407 | + | TACATGTAG |
|  |  |  | Motif-16 | -899 | - | CAGCCGGAGCACAAAC |
|  |  |  | Motif-2 | -99 | - | CTGGTTGAAGTG |
| P. infestans | PITG\_01697 | 1000 | Motif-0 | -320 | + | CATTCGACAATTTGCT |
|  |  |  | Motif-4 | -345 | + | TTTAATTGGCTATAA |
|  |  |  | Motif-8 | -179 | - | TTTTTTTTAGTTTTT |
| P. infestans | PITG\_01698 | 1000 | Motif-0 | -382 | + | TGATTCTGCATTTTAC |
|  |  |  | Motif-18 | -229 | - | TGGTGTGGTGTACACA |
| P. infestans | PITG\_01701 | 251 | Motif-4 | -102 | + | TCTGATTCGTCAAAA |
| P. infestans | PITG\_01702 | 329 | Motif-1 | -298 | + | TACATGTAG |
| P. infestans | PITG\_01703 | 291 | Motif-2 | -137 | - | CACTTCAACTT |
| P. infestans | PITG\_01704 | 291 | Motif-2 | -164 | + | ACTTCAACTTC |
| P. infestans | PITG\_01705 | 510 | Motif-0 | -445 | - | TCAGTTTGTTTTTCTC |
|  |  |  | Motif-2 | -206 | + | ACTTCAACTTG |
|  |  |  | Motif-3 | -190 | + | TACGAATACCAAGTAA |
|  |  |  | Motif-9 | -109 | + | GCGTACCGGTA |
| P. infestans | PITG\_01706 | 510 | Motif-0 | -81 | + | TCAGTTTGTTTTTCTC |
|  |  |  | Motif-2 | -314 | - | CACTTCAACTT |
|  |  |  | Motif-3 | -335 | + | TACTTGGTATTCGTAA |
|  |  |  | Motif-9 | -412 | - | GCGTACCGGTA |
| P. infestans | PITG\_01708 | 1000 | Motif-1 | -868 | - | GGTACGTGTAG |
| P. infestans | PITG\_01709 | 1000 | Motif-0 | -682 | + | CCATTCTCCAGTTTTC |
| P. infestans | PITG\_01710 | 1000 | Motif-3 | -482 | + | ACTTGGTTTCACTAT |
| P. infestans | PITG\_01711 | 277 | Motif-0 | -266 | - | GCACTTTGTATTTTTC |
|  |  |  | Motif-1 | -161 | + | TACATGTAA |
|  |  |  | Motif-2 | -224 | - | CAGCTTGAAGTG |
| P. infestans | PITG\_01712 | 277 | Motif-0 | -27 | + | GCACTTTGTATTTTTC |
|  |  |  | Motif-1 | -83 | + | GATACGTGTAG |
|  |  |  | Motif-2 | -65 | + | CAGCTTGAAGTG |
| P. infestans | PITG\_01715 | 1000 | Motif-1 | -199 | + | TACATGTGCT |
|  |  |  | Motif-8 | -629 | - | TTTGATTAAGTTTTA |
| P. infestans | PITG\_01716 | 1000 | Motif-0 | -74 | - | AGAAATTTAGGAATGA |
|  |  |  | Motif-1 | -885 | + | TACGTGAACCGGTAAT |
|  |  |  | Motif-8 | -557 | + | TTTGATTAAGTTTTA |
| P. infestans | PITG\_01718 | 399 | Motif-1 | -119 | + | TACATGTAG |
|  |  |  | Motif-17 | -329 | + | AATGTAGCTATCTTG |
|  |  |  | Motif-18 | -346 | + | CTGTGTGGTGCACACT |
| P. infestans | PITG\_01719 | 399 | Motif-1 | -76 | - | ACTAAATGTA |
|  |  |  | Motif-17 | -85 | - | AATGTAGCTATCTTG |
|  |  |  | Motif-18 | -69 | - | CTGTGTGGTGCACACT |
| P. infestans | PITG\_01720 | 141 | Motif-0 | -102 | + | CATTATCGAATTTACG |
| P. infestans | PITG\_01721 | 1000 | Motif-0 | -441 | - | CCACTCTCGGTTTTGA |
|  |  |  | Motif-1 | -949 | + | TACATGTAG |
|  |  |  | Motif-18 | -204 | - | TGGTGTGGTGTACATG |
|  |  |  | Motif-2 | -226 | + | ACTTCAACTTG |
| P. infestans | PITG\_01722 | 1000 | Motif-2 | -31 | + | GAAGTTGCAGTG |
| P. infestans | PITG\_01725 | 1000 | Motif-17 | -111 | + | TATGTAGCCATGTGT |
|  |  |  | Motif-2 | -96 | + | ACTTCAACATG |
|  |  |  | Motif-4 | -63 | - | GGTGATTGGAGAAAA |
| P. infestans | PITG\_01726 | 1000 | Motif-0 | -286 | + | GCAAATGCGGGAGTGA |
| P. infestans | PITG\_01731 | 1000 | Motif-1 | -478 | + | TACATGTAT |
|  |  |  | Motif-2 | -192 | + | ACTTCAACATG |
| P. infestans | PITG\_01732 | 1000 | Motif-0 | -813 | - | TCATTTTGGATTTAGA |
|  |  |  | Motif-1 | -706 | - | TTAATACACGTAGT |
|  |  |  | Motif-7 | -698 | - | TAATATTAATA |
| P. infestans | PITG\_01736 | 1000 | Motif-1 | -842 | + | AACATGTAGTTTTGT |
|  |  |  | Motif-2 | -526 | - | CACTTCAACAT |
|  |  |  | Motif-8 | -673 | + | TTTTTTTAAGCTATT |
| P. infestans | PITG\_01737 | 259 | Motif-0 | -213 | - | TTCAATTTTGAATTGA |
|  |  |  | Motif-17 | -247 | + | AATGTAGCCATATTG |
| P. infestans | PITG\_01738 | 644 | Motif-18 | -48 | - | ATGTGTGGTGCACATG |
|  |  |  | Motif-2 | -347 | + | TCGCTTCACCCTC |
| P. infestans | PITG\_01739 | 1000 | Motif-0 | -424 | - | TTATTTGCCATTTTGA |
|  |  |  | Motif-7 | -960 | + | TACTATTAGTA |
| P. infestans | PITG\_01740 | 1000 | Motif-4 | -654 | + | CATGATTGGATGATA |
| P. infestans | PITG\_01741 | 153 | Motif-0 | -18 | - | TCAAATTTGCAAATGA |
| P. infestans | PITG\_01742 | 153 | Motif-0 | -151 | + | TCAAATTTGCAAATGA |
| P. infestans | PITG\_01745 | 1000 | Motif-6 | -375 | - | CCCCCTCCCTCCGTCC |
| P. infestans | PITG\_01746 | 298 | Motif-4 | -259 | + | CTTGATTGGCTAATA |
| P. infestans | PITG\_01747 | 298 | Motif-4 | -54 | - | CTTGATTGGCTAATA |
| P. infestans | PITG\_01748 | 359 | Motif-1 | -310 | + | TACACGTACAATGGT |
|  |  |  | Motif-8 | -91 | + | TTTTTCTTAATATCA |
| P. infestans | PITG\_01751 | 1000 | Motif-1 | -67 | + | TACATGTAC |
|  |  |  | Motif-4 | -972 | - | TCTGGTTGGCCAAAT |
| P. infestans | PITG\_01752 | 1000 | Motif-0 | -61 | - | GAAAATCCGGAAATGA |
|  |  |  | Motif-1 | -604 | + | TGCATGTAGCTTGAT |
|  |  |  | Motif-4 | -152 | - | GCTGATTGGGCAGTA |
| P. infestans | PITG\_01753 | 303 | Motif-2 | -242 | + | ACTTCAACGTC |
|  |  |  | Motif-4 | -180 | + | TGGTATTGGTCAATA |
| P. infestans | PITG\_01755 | 169 | Motif-4 | -74 | + | TTTGATTGGTGGAAA |
| P. infestans | PITG\_01756 | 169 | Motif-4 | -53 | + | TATCAACCAATGTGC |
| P. infestans | PITG\_01759 | 481 | Motif-2 | -339 | - | GAGCTTCAACCAG |
| P. infestans | PITG\_01760 | 481 | Motif-2 | -155 | + | GAGCTTCAACCAG |
| P. infestans | PITG\_01761 | 373 | Motif-0 | -75 | + | CACATCGCAATTTGCC |
|  |  |  | Motif-3 | -353 | + | ACTTGGAGTTTGTAT |
| P. infestans | PITG\_01762 | 373 | Motif-0 | -313 | + | GCAAATTGCGATGTGG |
|  |  |  | Motif-3 | -34 | + | TACAAACTCCAAGTTA |
| P. infestans | PITG\_01768 | 1000 | Motif-0 | -36 | + | CATTCCTGAATTTGTC |
|  |  |  | Motif-1 | -128 | + | TGGACATGTAC |
| P. infestans | PITG\_01769 | 1000 | Motif-0 | -43 | + | TCACTTCAGAATTTTT |
|  |  |  | Motif-1 | -647 | + | ACTACAGGTA |
|  |  |  | Motif-17 | -303 | + | AGTGTAGCCATCCTG |
|  |  |  | Motif-8 | -160 | - | TATTTCTTAGTTTTT |
| P. infestans | PITG\_01770 | 207 | Motif-4 | -123 | + | CATTATTGGATGAAA |
| P. infestans | PITG\_01771 | 207 | Motif-4 | -99 | - | CATTATTGGATGAAA |
| P. infestans | PITG\_01772 | 506 | Motif-0 | -129 | - | CCGAATTCGGAAGTGA |
|  |  |  | Motif-1 | -66 | + | AACATGTATTTTAAA |
|  |  |  | Motif-7 | -317 | - | CAGTATTAATA |
| P. infestans | PITG\_01774 | 447 | Motif-2 | -78 | + | GCAACTTCAAGCAGTA |
| P. infestans | PITG\_01775 | 153 | Motif-2 | -107 | + | TAGCTTCAACTAG |
| P. infestans | PITG\_01778 | 1000 | Motif-16 | -789 | - | CAGCAGCCGCTCCAGC |
| P. infestans | PITG\_01781 | 1000 | Motif-3 | -735 | - | ACTTGAAGTTACTAT |
| P. infestans | PITG\_01782 | 595 | Motif-2 | -141 | - | AAAGTTGAAGCT |
| P. infestans | PITG\_01787 | 448 | Motif-0 | -152 | + | CACTTTTGTATTTGCT |
| P. infestans | PITG\_01789 | 1000 | Motif-8 | -436 | + | TTTTATTTTTTAAAT |
| P. infestans | PITG\_01790 | 1000 | Motif-0 | -48 | + | TCATTTTCCCTACTTC |
|  |  |  | Motif-1 | -688 | + | TACCTGTACCATGAT |
| P. infestans | PITG\_01791 | 250 | Motif-2 | -107 | + | GGCCAAGTCGAAGTAA |
|  |  |  | Motif-4 | -135 | - | GCTGATTGGCTGAAT |
| P. infestans | PITG\_01792 | 409 | Motif-4 | -75 | + | TTTAATTGGATGAAA |
| P. infestans | PITG\_01793 | 409 | Motif-4 | -318 | - | CTTGATTGGGTATAA |
| P. infestans | PITG\_01794 | 174 | Motif-1 | -121 | + | TACATGTAA |
| P. infestans | PITG\_01797 | 122 | Motif-4 | -98 | - | TATGATTGGTGGAAA |
| P. infestans | PITG\_01798 | 291 | Motif-2 | -241 | + | GCCCTTCAACATC |
| P. infestans | PITG\_01799 | 291 | Motif-2 | -63 | - | GCCCTTCAACATC |
| P. infestans | PITG\_01800 | 1000 | Motif-1 | -751 | - | ACTATACGTGTAAG |
|  |  |  | Motif-2 | -617 | - | ACTTCAAGGTG |
|  |  |  | Motif-3 | -339 | - | CATACAAAAGCGAAGT |
|  |  |  | Motif-9 | -737 | + | AGGTACCGGTA |
| P. infestans | PITG\_01801 | 1000 | Motif-0 | -426 | + | CATTCCGACATTCGCC |
|  |  |  | Motif-1 | -702 | + | TACATGTAG |
| P. infestans | PITG\_01802 | 1000 | Motif-0 | -59 | - | GTAAATCCTAGAATGA |
|  |  |  | Motif-2 | -700 | + | GACAAAGCTGAAGTGG |
| P. infestans | PITG\_01804 | 1000 | Motif-0 | -483 | + | CCAGTCTTAAATCTAC |
|  |  |  | Motif-4 | -70 | - | GGCGATTGGCTCAAA |
|  |  |  | Motif-8 | -500 | - | TATTTATAAGTAAGA |
| P. infestans | PITG\_01807 | 1000 | Motif-0 | -152 | + | GCAATTTCGAAAACGA |
|  |  |  | Motif-4 | -354 | + | GATGATTGGTCAATA |
|  |  |  | Motif-9 | -261 | + | ATTTACCGGTA |
| P. infestans | PITG\_01809 | 688 | Motif-0 | -654 | - | CACTCTCTAATTTCTC |
|  |  |  | Motif-1 | -302 | + | TGCATGTATTAAAAC |
|  |  |  | Motif-2 | -122 | - | CACTTCAACGT |
| P. infestans | PITG\_01810 | 688 | Motif-0 | -16 | + | CATTCTCGCTTTCGCC |
|  |  |  | Motif-1 | -394 | + | TACATGCAC |
|  |  |  | Motif-2 | -576 | + | ACTTCAACGTC |
| P. infestans | PITG\_01811 | 687 | Motif-2 | -355 | - | GCTTCAACTCG |
| P. infestans | PITG\_01815 | 312 | Motif-3 | -226 | + | ACTTGAATTTTGTAA |
|  |  |  | Motif-4 | -281 | - | GGGGATTGGCTAATA |
|  |  |  | Motif-8 | -26 | + | TATTTTTTTATAAAT |
| P. infestans | PITG\_01816 | 336 | Motif-0 | -240 | - | GCACTATTAAACTTCC |
|  |  |  | Motif-1 | -141 | + | TACATGTAG |
| P. infestans | PITG\_01817 | 336 | Motif-0 | -112 | + | GCACTATTAAACTTCC |
|  |  |  | Motif-1 | -117 | + | TACATGCAC |
| P. infestans | PITG\_01818 | 671 | Motif-0 | -124 | - | TAAAATTGGATAATGA |
| P. infestans | PITG\_01819 | 1000 | Motif-1 | -888 | + | TACATGTAC |
|  |  |  | Motif-17 | -415 | - | ACTGTACCCATACTG |
|  |  |  | Motif-9 | -880 | + | CTTTACCGGTA |
| P. infestans | PITG\_01821 | 1000 | Motif-0 | -996 | - | TCATTCTCCTACTGAC |
|  |  |  | Motif-1 | -274 | - | TACTTGTACA |
|  |  |  | Motif-17 | -118 | - | GGTGTAGCCATTTTG |
|  |  |  | Motif-2 | -835 | - | TACGTTGAAGCG |
| P. infestans | PITG\_01822 | 502 | Motif-1 | -204 | + | TACATGTGCT |
| P. infestans | PITG\_01823 | 502 | Motif-1 | -308 | + | AGCACATGTACAGTA |
| P. infestans | PITG\_01825 | 1000 | Motif-0 | -343 | - | GCATTCAGCATTTTAC |
|  |  |  | Motif-8 | -169 | - | TTCATTTAAGTTAAA |
| P. infestans | PITG\_01828 | 1000 | Motif-0 | -183 | + | TCCCTCCTGAACTCGC |
| P. infestans | PITG\_01830 | 1000 | Motif-0 | -877 | + | TCACAAATGAATTTGC |
|  |  |  | Motif-1 | -569 | + | TACATGTAA |
|  |  |  | Motif-8 | -611 | - | TTTTATTTTATTTAA |
| P. infestans | PITG\_01832 | 1000 | Motif-3 | -89 | + | GCTTGGAGTTTGTAT |
|  |  |  | Motif-4 | -680 | + | TGTGATTGGTTTAAA |
| P. infestans | PITG\_01833 | 393 | Motif-2 | -348 | + | CCACTTCAAGCGCA |
| P. infestans | PITG\_01836 | 1000 | Motif-0 | -927 | - | CCATTTGAAAATTCGC |
|  |  |  | Motif-1 | -352 | + | AGTAAATGTACTCCA |
|  |  |  | Motif-3 | -730 | - | ACTCGTATTTAGTAT |
|  |  |  | Motif-8 | -737 | + | TTTTTTTATACTAAA |
| P. infestans | PITG\_01837 | 424 | Motif-0 | -43 | + | TCACACTACATTTTGC |
| P. infestans | PITG\_01838 | 424 | Motif-0 | -323 | + | GCAAATGCCGTAATGA |
| P. infestans | PITG\_01839 | 732 | Motif-1 | -496 | + | TACATGTAC |
|  |  |  | Motif-2 | -236 | + | GCTTCAACATG |
|  |  |  | Motif-4 | -162 | + | TCCCATTGGTCAATT |
| P. infestans | PITG\_01840 | 1000 | Motif-0 | -42 | + | TCACTCCGTTTTTCGC |
|  |  |  | Motif-6 | -437 | - | GCAGCCCCCTCCCCAT |
| P. infestans | PITG\_01841 | 1000 | Motif-0 | -676 | - | GAAGGATTAAGAATGA |
| P. infestans | PITG\_01842 | 1000 | Motif-0 | -569 | - | TCACTTTTTTTTTTTC |
|  |  |  | Motif-2 | -442 | + | ACTTCAAGTTG |
|  |  |  | Motif-8 | -200 | + | TATTTCTAAGTATTT |
| P. infestans | PITG\_01844 | 1000 | Motif-0 | -846 | - | GTGAGATCAAAAATGA |
| P. infestans | PITG\_01845 | 1000 | Motif-1 | -353 | + | AGTACATCTAA |
|  |  |  | Motif-4 | -166 | - | TCAGATTGGGCAAAA |
| P. infestans | PITG\_01846 | 214 | Motif-4 | -147 | + | CATGATTGGTTAAAA |
| P. infestans | PITG\_01847 | 214 | Motif-4 | -82 | - | CATGATTGGTTAAAA |
| P. infestans | PITG\_01848 | 1000 | Motif-0 | -644 | - | ATCACTTCTTCTTTT |
|  |  |  | Motif-1 | -491 | + | TACATGTAC |
| P. infestans | PITG\_01849 | 1000 | Motif-1 | -621 | - | CCAAGTTACCTGTAC |
|  |  |  | Motif-2 | -380 | + | GTGCAACTTGCATTG |
|  |  |  | Motif-7 | -643 | - | CACTATTAATA |
| P. infestans | PITG\_01850 | 1000 | Motif-0 | -26 | + | GCAGGTCGGCAAGTGA |
| P. infestans | PITG\_01851 | 313 | Motif-1 | -238 | + | TACATGTAG |
|  |  |  | Motif-15 | -133 | - | GACTTCAACCACGT |
|  |  |  | Motif-17 | -297 | + | AGTGTAGCCATATTT |
|  |  |  | Motif-3 | -100 | + | TCTTACAGTTTGTAT |
| P. infestans | PITG\_01852 | 182 | Motif-4 | -119 | - | TTTAGACCAATGTGA |
| P. infestans | PITG\_01853 | 182 | Motif-4 | -78 | + | TTTAGACCAATGTGA |
| P. infestans | PITG\_01855 | 1000 | Motif-1 | -811 | + | AGTATACGTGTATT |
|  |  |  | Motif-4 | -92 | + | CTTTATTGGCCAAAA |
| P. infestans | PITG\_01857 | 466 | Motif-2 | -154 | + | TCAACATCAAGTTCGA |
| P. infestans | PITG\_01858 | 179 | Motif-0 | -40 | + | TCATTTCACAACACGC |
|  |  |  | Motif-2 | -116 | + | ACTTCAACGTC |
| P. infestans | PITG\_01859 | 179 | Motif-0 | -154 | - | TCATTTCACAACACG |
|  |  |  | Motif-2 | -73 | - | CACTTCAACGT |
| P. infestans | PITG\_01860 | 1000 | Motif-1 | -452 | - | CTAAGGTAAATGTAT |
|  |  |  | Motif-2 | -747 | + | GCTTCAACTTTCTCAA |
| P. infestans | PITG\_01862 | 1000 | Motif-0 | -57 | + | CATTCCACAATTTGCG |
|  |  |  | Motif-2 | -134 | + | CCACTTCAACTGCG |
|  |  |  | Motif-4 | -740 | + | CGTGATTGGCCGGAT |
|  |  |  | Motif-6 | -142 | + | ACCCCTCCCCACTTCA |
| P. infestans | PITG\_01864 | 1000 | Motif-1 | -160 | + | TACAAGTACT |
| P. infestans | PITG\_01865 | 686 | Motif-6 | -576 | + | CGACCCCCCTCCGCAT |
| P. infestans | PITG\_01867 | 154 | Motif-17 | -122 | + | AGTGTAGCCATCTCA |
|  |  |  | Motif-18 | -104 | + | TGATGTGGCTCACATA |
|  |  |  | Motif-2 | -138 | + | GCGCTTCAAGATCG |
| P. infestans | PITG\_01868 | 154 | Motif-17 | -47 | - | AGTGTAGCCATCTCA |
|  |  |  | Motif-18 | -66 | - | TGATGTGGCTCACATA |
|  |  |  | Motif-2 | -30 | - | GCGCTTCAAGATCG |
| P. infestans | PITG\_01869 | 436 | Motif-0 | -50 | + | TCATTCCAGTATTCGA |
|  |  |  | Motif-1 | -322 | + | ATTAAATGTA |
|  |  |  | Motif-17 | -387 | + | AATGTAGCCATATCT |
|  |  |  | Motif-2 | -275 | - | CTGCATCATGCAGTG |
| P. infestans | PITG\_01870 | 436 | Motif-0 | -402 | - | TCATTCCAGTATTCGA |
|  |  |  | Motif-1 | -124 | - | ATTAAATGTA |
|  |  |  | Motif-17 | -64 | - | AATGTAGCCATATCT |
|  |  |  | Motif-2 | -176 | + | CTGCATCATGCAGTG |
| P. infestans | PITG\_01871 | 1000 | Motif-1 | -310 | + | TACATGTAT |
|  |  |  | Motif-2 | -712 | + | TTGCTTCAAGTTGA |
|  |  |  | Motif-3 | -138 | - | CACTTGGAGATTGTAA |
| P. infestans | PITG\_01872 | 675 | Motif-2 | -95 | + | ACTTCAACATC |
| P. infestans | PITG\_01873 | 675 | Motif-2 | -590 | - | CACTTCAACAT |
| P. infestans | PITG\_01875 | 1000 | Motif-0 | -38 | + | GCACTTTCGATTCGAC |
|  |  |  | Motif-1 | -815 | + | CAAACATGTAGCACA |
| P. infestans | PITG\_01877 | 471 | Motif-0 | -113 | - | GCAAATTTGATAATGA |
|  |  |  | Motif-4 | -246 | - | CCTGATTGGGTAATA |
| P. infestans | PITG\_01878 | 471 | Motif-0 | -374 | + | GCAAATTTGATAATGA |
|  |  |  | Motif-4 | -240 | + | CCTGATTGGGTAATA |
| P. infestans | PITG\_01879 | 1000 | Motif-1 | -159 | - | GGTGCATATAC |
| P. infestans | PITG\_01880 | 1000 | Motif-0 | -185 | + | ATCACTTTTTGTTTT |
|  |  |  | Motif-6 | -275 | - | AGCCCCCCCCTCCCCC |
| P. infestans | PITG\_01881 | 1000 | Motif-0 | -64 | - | GCAGGTTAAGGAATGA |
|  |  |  | Motif-1 | -301 | - | AAATACTACATTTAC |
| P. infestans | PITG\_01882 | 911 | Motif-0 | -896 | - | GCACTCGGCATTCCGC |
|  |  |  | Motif-1 | -361 | + | ACAACATGTAGCTTA |
| P. infestans | PITG\_01883 | 911 | Motif-0 | -31 | + | GCACTCGGCATTCCGC |
|  |  |  | Motif-1 | -160 | + | TACATGTAT |
| P. infestans | PITG\_01884 | 1000 | Motif-0 | -30 | - | GCAGATTGTGGAATGC |
|  |  |  | Motif-4 | -904 | - | TTTGATAGGCTAATT |
|  |  |  | Motif-8 | -895 | - | TTTTTTTAATTTGAT |
| P. infestans | PITG\_01890 | 40 | Motif-1 | -10 | + | TACATATAC |
| P. infestans | PITG\_01896 | 262 | Motif-0 | -154 | - | CAGTTTTACATGTGCT |
|  |  |  | Motif-1 | -99 | + | AGTACCTGTATTGTA |
| P. infestans | PITG\_01897 | 1000 | Motif-3 | -514 | - | TACATAAGTCGAGTCA |
| P. infestans | PITG\_01898 | 97 | Motif-3 | -67 | + | TACAAACGTCCACTCA |
| P. infestans | PITG\_01902 | 1000 | Motif-0 | -42 | + | TCACTTTGACATTTTA |
| P. infestans | PITG\_01904 | 1000 | Motif-0 | -49 | + | TCATTTTGCGATTGGG |
|  |  |  | Motif-1 | -198 | - | AAATAGTACCTGTAC |
|  |  |  | Motif-2 | -892 | - | GTAATTGAAGTGGC |
| P. infestans | PITG\_01905 | 1000 | Motif-0 | -47 | + | CCACTTCAAATTCTGC |
|  |  |  | Motif-1 | -85 | + | TGCATGTAC |
|  |  |  | Motif-2 | -46 | - | GAATTTGAAGTG |
|  |  |  | Motif-7 | -903 | - | TACTACTAATA |
| P. infestans | PITG\_01907 | 1000 | Motif-0 | -40 | - | GTAGAATGCAAAGTGG |
|  |  |  | Motif-6 | -877 | - | TCCCCCAGCCCCCCCC |
| P. infestans | PITG\_01913 | 1000 | Motif-8 | -131 | - | TTTTTTTAATTTTTT |
| P. infestans | PITG\_01914 | 1000 | Motif-1 | -393 | - | TGTACATGTCTA |
|  |  |  | Motif-3 | -503 | + | GACTTGAAAGCTGTAT |
|  |  |  | Motif-4 | -282 | + | CATGATTGGCTAAAG |
| P. infestans | PITG\_01919 | 1000 | Motif-0 | -537 | + | GCCAATTCTGAAATGA |
|  |  |  | Motif-1 | -75 | + | TACACGTATAAAAGT |
|  |  |  | Motif-4 | -630 | + | TATGATTGGATATTA |
|  |  |  | Motif-6 | -198 | + | CCCCCCCCCCCCGAGC |
|  |  |  | Motif-7 | -424 | - | TAATATTAATA |
| P. infestans | PITG\_01920 | 1000 | Motif-4 | -59 | - | AGTGATTGGAGGAAA |
| P. infestans | PITG\_01922 | 1000 | Motif-18 | -148 | - | TGGTGTGGTGCACGTA |
| P. infestans | PITG\_01923 | 468 | Motif-3 | -83 | + | TACAAATACCAAGACA |
| P. infestans | PITG\_01934 | 1000 | Motif-0 | -45 | + | CAGTTTGCAATTTGCT |
|  |  |  | Motif-1 | -175 | + | TACATGTAA |
|  |  |  | Motif-17 | -965 | - | AATGTAGTTATTTTT |
| P. infestans | PITG\_01936 | 1000 | Motif-0 | -115 | - | GAGAATTGAAAAATGG |
| P. infestans | PITG\_01937 | 1000 | Motif-2 | -49 | - | ACGGAAGCTGAAGCTG |
|  |  |  | Motif-3 | -217 | + | ACTCGATCTCAGTAT |
|  |  |  | Motif-6 | -301 | - | CCTTCTACCCCCCCCC |
| P. infestans | PITG\_01938 | 1000 | Motif-1 | -427 | + | AACATGTAC |
|  |  |  | Motif-9 | -441 | - | AAGTACCGGTA |
| P. infestans | PITG\_01940 | 1000 | Motif-1 | -898 | - | AGTATACATGTGCT |
|  |  |  | Motif-2 | -101 | + | GCTTCAACGTCATTCA |
| P. infestans | PITG\_01941 | 1000 | Motif-0 | -19 | + | TCACTCTTGTTTCCGC |
|  |  |  | Motif-1 | -267 | + | TACATGTAC |
|  |  |  | Motif-17 | -111 | + | AGTGTATCCATGTTG |
| P. infestans | PITG\_01942 | 1000 | Motif-0 | -973 | - | TCACTGCTGCATCTTC |
|  |  |  | Motif-1 | -812 | - | TACGTGTAC |
|  |  |  | Motif-6 | -855 | + | CGCTCTCCCCCGACAC |
| P. infestans | PITG\_01945 | 1000 | Motif-18 | -413 | - | TGGAGTGGCTTACAAA |
|  |  |  | Motif-2 | -209 | - | TAACTTGAAGTG |
|  |  |  | Motif-4 | -198 | - | TTTGATTAGTTGATT |
| P. infestans | PITG\_01946 | 877 | Motif-8 | -427 | - | TACTTTTTAACTAAA |
| P. infestans | PITG\_01947 | 877 | Motif-8 | -465 | + | TACTTTTTAACTAAA |
| P. infestans | PITG\_01948 | 727 | Motif-1 | -652 | - | ACGACATGTAT |
| P. infestans | PITG\_01949 | 1000 | Motif-1 | -230 | - | AATACATATAG |
|  |  |  | Motif-2 | -670 | - | CGCTTCAACGT |
|  |  |  | Motif-4 | -79 | + | TATTATTGGCCAGAT |
|  |  |  | Motif-8 | -223 | + | TATTATTATGTAATA |
| P. infestans | PITG\_01950 | 1000 | Motif-0 | -177 | + | TCATTGCGCAACTCGA |
|  |  |  | Motif-1 | -744 | - | TGCATGTAC |
|  |  |  | Motif-2 | -691 | - | AACGTTGAAGCG |
|  |  |  | Motif-3 | -204 | + | TACAAACCTCAAGTCT |
|  |  |  | Motif-4 | -271 | - | TATGATTGGTCAATA |
| P. infestans | PITG\_01951 | 838 | Motif-0 | -25 | + | TTATTTTCAAACTTG |
|  |  |  | Motif-1 | -88 | + | TGTAAATGTATTTTA |
|  |  |  | Motif-2 | -574 | + | ACTTCAACTTC |
| P. infestans | PITG\_01952 | 838 | Motif-0 | -203 | + | TCATTCCACATCCAGC |
|  |  |  | Motif-1 | -762 | + | AATACATTTACAGATA |
|  |  |  | Motif-2 | -274 | - | AACTTCAACTT |
| P. infestans | PITG\_01955 | 1000 | Motif-1 | -853 | + | TACATGTAC |
|  |  |  | Motif-2 | -652 | - | AACTTCAACTT |
| P. infestans | PITG\_01957 | 881 | Motif-0 | -239 | - | TCATTTTTCGAGTCGA |
|  |  |  | Motif-1 | -439 | + | TACATGTAG |
|  |  |  | Motif-2 | -95 | - | CACTTCAACTT |
|  |  |  | Motif-4 | -525 | - | TTTGATTGGTTGTCA |
| P. infestans | PITG\_01958 | 881 | Motif-0 | -28 | + | CACTTTTCAATTTGCA |
|  |  |  | Motif-1 | -450 | + | TACATGTAG |
|  |  |  | Motif-2 | -332 | - | CACTTCAACGT |
|  |  |  | Motif-4 | -371 | + | TTTGATTGGTTGTCA |
| P. infestans | PITG\_01959 | 1000 | Motif-2 | -390 | + | AACTTCAGCATGGA |
| P. infestans | PITG\_01960 | 1000 | Motif-3 | -66 | + | TCTTGAAGTTTGTAT |
| P. infestans | PITG\_01961 | 422 | Motif-0 | -157 | + | TCACTACCGTATTCAC |
| P. infestans | PITG\_01962 | 422 | Motif-0 | -281 | - | TCACTACCGTATTCAC |
| P. infestans | PITG\_01963 | 533 | Motif-0 | -109 | + | CATTTTTCATTTTGCG |
|  |  |  | Motif-1 | -426 | + | TACATGTAC |
| P. infestans | PITG\_01964 | 1000 | Motif-0 | -779 | + | TCAATTTGGGAACTGA |
|  |  |  | Motif-1 | -317 | - | GAAACATACATGTTC |
| P. infestans | PITG\_01966 | 1000 | Motif-1 | -473 | + | AACATGTAC |
|  |  |  | Motif-8 | -776 | + | TTCATTTAATTATAA |
| P. infestans | PITG\_01969 | 1000 | Motif-0 | -836 | - | CACTTTAAAACTCACC |
|  |  |  | Motif-1 | -380 | + | TACATGTAA |
|  |  |  | Motif-4 | -666 | + | TGTTATTGGCTAATA |
| P. infestans | PITG\_01970 | 1000 | Motif-9 | -359 | + | GTGTACCGGTA |
| P. infestans | PITG\_01971 | 1000 | Motif-8 | -972 | + | TATGTTTAATCTTTA |
| P. infestans | PITG\_01973 | 1000 | Motif-0 | -46 | + | CACTTTCCAAGTTGTC |
|  |  |  | Motif-8 | -484 | - | TTTGTCTAATTTAAA |
| P. infestans | PITG\_01974 | 354 | Motif-2 | -193 | + | GCTTCAACGTG |
| P. infestans | PITG\_01975 | 354 | Motif-2 | -172 | + | CACGTTGAAGCA |
| P. infestans | PITG\_01977 | 164 | Motif-0 | -158 | - | TCAGTATTGAACCTGT |
|  |  |  | Motif-2 | -55 | + | ACTTCAACGTC |
|  |  |  | Motif-3 | -148 | + | TACTGAGACCAAGTCA |
| P. infestans | PITG\_01978 | 164 | Motif-0 | -22 | + | TCAGTATTGAACCTGT |
|  |  |  | Motif-2 | -43 | + | GGTGTTGAAGTT |
|  |  |  | Motif-3 | -30 | + | ACTTGGTCTCAGTAT |
| P. infestans | PITG\_01982 | 1000 | Motif-0 | -238 | - | TTCACTTCTTTTTTT |
|  |  |  | Motif-3 | -143 | - | AGCTTGGCGTTTGTAT |
|  |  |  | Motif-4 | -168 | - | TGTGATTGGCAAATA |
|  |  |  | Motif-7 | -575 | + | CACTATTAATA |
|  |  |  | Motif-8 | -572 | - | TTTATCTTATTAATA |
| P. infestans | PITG\_01984 | 1000 | Motif-2 | -622 | - | ACGACTTCAACGACTA |
|  |  |  | Motif-4 | -992 | - | TCTGATTGGCTCGAA |
| P. infestans | PITG\_01985 | 1000 | Motif-0 | -37 | + | CACTTCGAAACTTGCC |
|  |  |  | Motif-1 | -252 | + | TACATGTAT |
|  |  |  | Motif-3 | -845 | - | TACTAATATTCAGTGA |
| P. infestans | PITG\_01986 | 1000 | Motif-1 | -834 | + | TACGTGTAC |
|  |  |  | Motif-3 | -255 | + | TACTAATATTCAGTGA |
| P. infestans | PITG\_01988 | 350 | Motif-0 | -38 | + | CACTTCCACTTTCGCC |
| P. infestans | PITG\_01989 | 282 | Motif-2 | -216 | - | AACTTCAACAT |
| P. infestans | PITG\_01990 | 282 | Motif-2 | -76 | + | ACTTCAACATC |
| P. infestans | PITG\_01991 | 251 | Motif-0 | -81 | + | GCATTTCTGATTCCAC |
|  |  |  | Motif-3 | -97 | + | ACTTGGCCTTAGTAA |
| P. infestans | PITG\_01992 | 251 | Motif-0 | -186 | - | GCATTTCTGATTCCAC |
|  |  |  | Motif-3 | -71 | + | ACTTGGAATTTGTAG |
| P. infestans | PITG\_01993 | 1000 | Motif-0 | -561 | - | GCAAAACCAAGAATGA |
| P. infestans | PITG\_01994 | 255 | Motif-1 | -41 | - | TGCATGTAC |
| P. infestans | PITG\_01995 | 385 | Motif-0 | -382 | + | GCAGTATCAAAAGTGA |
| P. infestans | PITG\_01996 | 385 | Motif-0 | -19 | - | GCAGTATCAAAAGTGA |
| P. infestans | PITG\_01997 | 1000 | Motif-0 | -46 | - | TCACTCTTGTTTCTGA |
| P. infestans | PITG\_01999 | 436 | Motif-0 | -395 | + | TTATTCTTGAATCGTC |
| P. infestans | PITG\_02000 | 1000 | Motif-0 | -49 | - | GGCAGTTGAGAAGTGA |
|  |  |  | Motif-1 | -167 | + | TACATGTAT |
|  |  |  | Motif-3 | -197 | - | GTCTTGCAGTCTGTAT |
|  |  |  | Motif-4 | -881 | - | TTTGATTGGCTGAGA |
| P. infestans | PITG\_02001 | 541 | Motif-0 | -47 | + | GCATTCTCCGATCCCC |
| P. infestans | PITG\_02002 | 541 | Motif-0 | -510 | - | GCATTCTCCGATCCCC |
| P. infestans | PITG\_02003 | 1000 | Motif-3 | -445 | - | TACTTATGCTTTGTAT |
| P. infestans | PITG\_02005 | 1000 | Motif-0 | -34 | + | CACTTCGGAATTTTCT |
|  |  |  | Motif-4 | -921 | - | TATGACTGGTCGAAA |
| P. infestans | PITG\_02010 | 279 | Motif-2 | -228 | + | CCACTTCAACTGTA |
| P. infestans | PITG\_02016 | 1000 | Motif-0 | -369 | + | TCATTTTTCAAGTTTA |
|  |  |  | Motif-4 | -49 | - | GATGATTGGTTAATA |
|  |  |  | Motif-8 | -354 | - | TTTTTTTAATTTTTT |
| P. infestans | PITG\_02019 | 477 | Motif-0 | -158 | - | TCATTACTCAATCCGA |
| P. infestans | PITG\_02021 | 328 | Motif-1 | -67 | + | TACATGTAT |
|  |  |  | Motif-4 | -155 | + | TTTGATTGGCTATTA |
| P. infestans | PITG\_02022 | 711 | Motif-2 | -475 | + | CAGCTTCAACGAC |
|  |  |  | Motif-6 | -83 | + | TCCCCCCCCTCCGCCA |
| P. infestans | PITG\_02026 | 329 | Motif-4 | -55 | + | TTTGATTGGTGAAAA |
| P. infestans | PITG\_02027 | 286 | Motif-0 | -85 | - | GAAAATTTGGGACTGA |
|  |  |  | Motif-17 | -239 | + | AATGTAGCCATGTGT |
|  |  |  | Motif-18 | -133 | + | TGGTGTGGCGCACATG |
| P. infestans | PITG\_02028 | 378 | Motif-2 | -80 | - | ACAGCTTCAAGTCCAA |
|  |  |  | Motif-7 | -260 | + | CAATATTAATA |
| P. infestans | PITG\_02030 | 443 | Motif-0 | -39 | - | GCAGATTCGAAAGTGG |
| P. infestans | PITG\_02031 | 1000 | Motif-1 | -643 | + | TACATGTAC |
|  |  |  | Motif-4 | -309 | - | CCTGATTGGATAAAA |
| P. infestans | PITG\_02032 | 1000 | Motif-1 | -124 | + | TACATGTAG |
|  |  |  | Motif-2 | -138 | + | ACAACTTCAAGGAGTA |
| P. infestans | PITG\_02034 | 1000 | Motif-1 | -419 | - | TCCAATTACATGGAG |
|  |  |  | Motif-3 | -341 | + | CTACAAAAGGCAAGAT |
| P. infestans | PITG\_02035 | 1000 | Motif-0 | -149 | - | TCAGTTTCTTTTTCGC |
|  |  |  | Motif-1 | -622 | + | TACATGCAC |
|  |  |  | Motif-18 | -578 | - | TTGTGTGGTGCACATA |
|  |  |  | Motif-2 | -516 | - | TTTGTTGAAGTG |
| P. infestans | PITG\_02036 | 1000 | Motif-0 | -262 | + | TCACTCAGCTATTTTC |
|  |  |  | Motif-2 | -75 | + | ACTTCAACTTC |
|  |  |  | Motif-6 | -664 | + | GCCACCTCCCCCTCAC |
| P. infestans | PITG\_02037 | 1000 | Motif-0 | -125 | - | TCACTTTTCTTCTTCG |
| P. infestans | PITG\_02038 | 1000 | Motif-0 | -48 | + | CCATTCGCGAATCTTC |
|  |  |  | Motif-4 | -349 | - | TCTGATTGGGCAAAA |
| P. infestans | PITG\_02039 | 753 | Motif-1 | -242 | + | TACATGTAT |
| P. infestans | PITG\_02040 | 271 | Motif-2 | -106 | + | CTTGTTGAAGTT |
|  |  |  | Motif-3 | -198 | + | TTCAAATACCAAGTGA |
| P. infestans | PITG\_02041 | 271 | Motif-2 | -177 | - | CTTGTTGAAGTT |
|  |  |  | Motif-3 | -88 | + | CACTTGGTATTTGAAG |
| P. infestans | PITG\_02042 | 557 | Motif-0 | -116 | + | TCACTTTGCAAGTGTC |
|  |  |  | Motif-2 | -437 | + | TCAGTTGAAGTTGC |
|  |  |  | Motif-4 | -193 | + | TATCATTGGCCAAAA |
| P. infestans | PITG\_02043 | 557 | Motif-0 | -148 | + | TCAGTGCGATATTGGC |
|  |  |  | Motif-2 | -134 | - | TCAGTTGAAGTTGC |
|  |  |  | Motif-4 | -379 | - | TATCATTGGCCAAAA |
| P. infestans | PITG\_02046 | 1000 | Motif-1 | -63 | + | TGTGGATGTACCC |
|  |  |  | Motif-2 | -708 | + | GCTTCAACGTG |
| P. infestans | PITG\_02047 | 1000 | Motif-2 | -654 | - | GGCTTCAACGT |
| P. infestans | PITG\_02048 | 453 | Motif-0 | -183 | + | ACATTTTCCAGTTTCC |
|  |  |  | Motif-18 | -70 | - | TTATGTGGCTTACACA |
| P. infestans | PITG\_02049 | 1000 | Motif-0 | -204 | - | GGGGATTGAGAAATGG |
|  |  |  | Motif-1 | -114 | - | AGTGTACGAGTAGC |
|  |  |  | Motif-2 | -328 | - | GACTTTGAAGTG |
| P. infestans | PITG\_02050 | 1000 | Motif-2 | -432 | + | GACCTTCAACATC |
| P. infestans | PITG\_02052 | 173 | Motif-4 | -33 | + | TTTCATTGGTTGAAA |
| P. infestans | PITG\_02055 | 1000 | Motif-6 | -998 | + | CCCCCCCCCCCTTCCT |
| P. infestans | PITG\_02058 | 1000 | Motif-0 | -73 | + | CCATTCAGGAATTCTC |
|  |  |  | Motif-1 | -220 | + | GATACAGGAACTGTA |
|  |  |  | Motif-2 | -586 | - | AACTTCAACAT |
| P. infestans | PITG\_02062 | 1000 | Motif-4 | -62 | + | CACGACTGGCTGAAA |
| P. infestans | PITG\_02063 | 1000 | Motif-0 | -64 | + | TCATTCGTCAAGTTAT |
| P. infestans | PITG\_02067 | 519 | Motif-17 | -334 | + | AGTGAAGCCATCTTT |
|  |  |  | Motif-2 | -77 | - | GTCGTTGAAGTG |
|  |  |  | Motif-7 | -144 | + | TAGTATTAATA |
|  |  |  | Motif-8 | -151 | - | TAATACTAAATTTAA |
| P. infestans | PITG\_02068 | 384 | Motif-0 | -81 | - | GCAAACCGCGAAATGA |
|  |  |  | Motif-4 | -227 | + | TAGGATTGGTTTAAA |
| P. infestans | PITG\_02069 | 384 | Motif-0 | -319 | + | GCAAACCGCGAAATGA |
|  |  |  | Motif-4 | -172 | - | TAGGATTGGTTTAAA |
| P. infestans | PITG\_02070 | 1000 | Motif-1 | -806 | - | TATACATATATTATA |
|  |  |  | Motif-3 | -706 | + | GACCTACCTTTTGTAT |
| P. infestans | PITG\_02071 | 1000 | Motif-16 | -67 | + | CAGCAGCAGCAGTAAC |
|  |  |  | Motif-3 | -626 | + | TACAAACGCTTAGTCA |
|  |  |  | Motif-6 | -396 | + | GCCCCCCCCCCTCACT |
| P. infestans | PITG\_02072 | 1000 | Motif-0 | -21 | - | TCAAATTTGAGTATGA |
|  |  |  | Motif-4 | -40 | + | TACAATTGGTCAATA |
|  |  |  | Motif-6 | -327 | - | ACCTCCCCCCCCCCCC |
|  |  |  | Motif-8 | -497 | + | TTTTTTTTAGTAAAA |
| P. infestans | PITG\_02073 | 1000 | Motif-2 | -38 | + | GCTTCAACCTGGGTCA |
|  |  |  | Motif-3 | -381 | - | ACTGGATGTATGTAT |
|  |  |  | Motif-4 | -452 | + | AACCATTGGCTGAAT |
| P. infestans | PITG\_02074 | 266 | Motif-6 | -235 | + | TCCCCCACCCCCGCGC |
| P. infestans | PITG\_02075 | 986 | Motif-0 | -55 | + | CATTCCCGCATTTCTC |
|  |  |  | Motif-1 | -261 | + | TACATGTAC |
|  |  |  | Motif-2 | -456 | - | CACTTCAACTT |
|  |  |  | Motif-4 | -140 | - | GTGGATTGGATAAAA |
|  |  |  | Motif-8 | -670 | - | TATGTTTTAGTAATT |
| P. infestans | PITG\_02076 | 986 | Motif-0 | -946 | - | TCATTCCCGCATTTCT |
|  |  |  | Motif-1 | -733 | + | TACATGTAC |
|  |  |  | Motif-2 | -540 | + | ACTTCAACTTG |
|  |  |  | Motif-4 | -861 | + | GTGGATTGGATAAAA |
|  |  |  | Motif-8 | -331 | + | TATGTTTTAGTAATT |
| P. infestans | PITG\_02077 | 122 | Motif-2 | -91 | - | CACTTCGACTT |
| P. infestans | PITG\_02078 | 735 | Motif-6 | -506 | + | CCAACTCCCCCGCTCC |
| P. infestans | PITG\_02079 | 199 | Motif-2 | -190 | + | ACTTCAACTTG |
|  |  |  | Motif-4 | -135 | - | CAGGATTGGCAGAAA |
| P. infestans | PITG\_02080 | 804 | Motif-0 | -94 | - | GGCAATTGGGGAATGA |
|  |  |  | Motif-1 | -695 | + | ACAGTCCATGTATT |
| P. infestans | PITG\_02081 | 804 | Motif-0 | -56 | - | CCAGATTGAGAAATGA |
|  |  |  | Motif-1 | -123 | + | AATACATGGACTGTA |
| P. infestans | PITG\_02082 | 876 | Motif-0 | -59 | - | GCAAATGTATGAATGA |
|  |  |  | Motif-1 | -462 | + | TACATGTAT |
|  |  |  | Motif-3 | -644 | + | ACTTGAATTCAGTAT |
|  |  |  | Motif-9 | -846 | - | ACGTACCGGTA |
| P. infestans | PITG\_02083 | 876 | Motif-0 | -833 | + | GCAAATGTATGAATGA |
|  |  |  | Motif-1 | -40 | + | CGTACCGGTACAGTA |
|  |  |  | Motif-3 | -247 | - | ACTTGAATTCAGTAT |
|  |  |  | Motif-9 | -38 | - | CTGTACCGGTA |
| P. infestans | PITG\_02084 | 1000 | Motif-1 | -85 | - | GTACAGTACCTGTAC |
|  |  |  | Motif-18 | -854 | - | TGGTGTAGTGTACGCA |
|  |  |  | Motif-7 | -121 | + | TAGTATTAATA |
|  |  |  | Motif-8 | -118 | + | TATTAATAAATTTAT |
| P. infestans | PITG\_02085 | 393 | Motif-0 | -38 | - | GAGAATTGAAGAATGA |
|  |  |  | Motif-1 | -369 | + | TACATGTAG |
| P. infestans | PITG\_02086 | 519 | Motif-0 | -151 | + | TCATTTTAGAAGTTAC |
|  |  |  | Motif-2 | -285 | + | CTCTTCAACGT |
| P. infestans | PITG\_02087 | 246 | Motif-0 | -42 | + | CATTGTGCAATTTACC |
|  |  |  | Motif-2 | -69 | - | GGCGAAGCTGAAGTGA |
| P. infestans | PITG\_02088 | 246 | Motif-0 | -219 | + | GTAAATTGCACAATGG |
|  |  |  | Motif-2 | -193 | + | GGCGAAGCTGAAGTGA |
| P. infestans | PITG\_02092 | 1000 | Motif-0 | -554 | + | GAGAATGCAACAATGA |
|  |  |  | Motif-2 | -517 | + | GCTTCAACCTAACTTA |
|  |  |  | Motif-4 | -631 | - | TCTGATTAGACGAAA |
| P. infestans | PITG\_02098 | 1000 | Motif-0 | -89 | - | GTCGATTGAGGAATGA |
|  |  |  | Motif-1 | -530 | + | TACATGTTCTAATTG |
|  |  |  | Motif-2 | -476 | - | TAACCTCAACATC |
|  |  |  | Motif-4 | -523 | + | TCTAATTGGCTAAAA |
| P. infestans | PITG\_02100 | 1000 | Motif-1 | -21 | + | TACATGTAG |
| P. infestans | PITG\_02102 | 1000 | Motif-0 | -591 | - | GCATTATTATTTTTGC |
|  |  |  | Motif-1 | -751 | + | AGTAAATGTACAGCG |
|  |  |  | Motif-8 | -433 | + | TTTGATTAAATATAT |
| P. infestans | PITG\_02103 | 443 | Motif-9 | -260 | + | ACGTACCGGTA |
| P. infestans | PITG\_02104 | 1000 | Motif-0 | -69 | + | ACATTCCTCATTCCGC |
|  |  |  | Motif-1 | -537 | + | TACATGTAT |
| P. infestans | PITG\_02105 | 1000 | Motif-0 | -259 | - | CCATTTCTCAACTTC |
|  |  |  | Motif-1 | -160 | + | TACATGTAC |
|  |  |  | Motif-2 | -265 | + | GCGGTTGAAGTTGA |
|  |  |  | Motif-4 | -82 | - | TCTGATTGGAAGAAT |
| P. infestans | PITG\_02106 | 817 | Motif-1 | -429 | + | TACATGTAA |
| P. infestans | PITG\_02110 | 766 | Motif-6 | -120 | + | ACTCCTCGCCCCACCT |
|  |  |  | Motif-8 | -462 | + | TTTTTCTAAACTAAT |
| P. infestans | PITG\_02111 | 175 | Motif-0 | -29 | - | GGAATTGGAAGAATGA |
|  |  |  | Motif-4 | -66 | - | TTTTATAGGCTAAAA |
| P. infestans | PITG\_02112 | 175 | Motif-0 | -162 | + | GGAATTGGAAGAATGA |
|  |  |  | Motif-4 | -101 | - | TATTATTGGGTGAAA |
| P. infestans | PITG\_02113 | 325 | Motif-2 | -296 | - | CTTGTTGAAGTT |
| P. infestans | PITG\_02114 | 236 | Motif-0 | -29 | + | TCACTCCGATTTCCGC |
| P. infestans | PITG\_02115 | 1000 | Motif-0 | -764 | + | TCACTTTCCAACGTCT |
|  |  |  | Motif-2 | -214 | - | CAAGTTCAAGTG |
|  |  |  | Motif-3 | -241 | + | TACAAAACCCAAGTTA |
| P. infestans | PITG\_02116 | 1000 | Motif-16 | -729 | - | CCACAGGAGCTGCAGC |
| P. infestans | PITG\_02118 | 1000 | Motif-0 | -671 | + | GAAAAGTGCAAACTGA |
|  |  |  | Motif-8 | -691 | - | TATTTTTAATTTTAA |
| P. infestans | PITG\_02119 | 1000 | Motif-0 | -145 | + | TCAGTCACCATTCTAC |
|  |  |  | Motif-1 | -236 | + | TACATGTAT |
|  |  |  | Motif-3 | -823 | + | TCTTGCCTTCTGTAT |
| P. infestans | PITG\_02122 | 585 | Motif-0 | -551 | + | GCGAATTTAGCAATGC |
|  |  |  | Motif-1 | -404 | + | TACATGTAG |
|  |  |  | Motif-2 | -151 | - | CACTTCAACAT |
|  |  |  | Motif-4 | -74 | - | TACGATTGGTGGAAA |
| P. infestans | PITG\_02124 | 1000 | Motif-1 | -71 | - | CACACATGTATAGGA |
|  |  |  | Motif-2 | -560 | - | CACTTCAACAT |
|  |  |  | Motif-4 | -151 | + | TGCGATTGGCAGAAA |
| P. infestans | PITG\_02129 | 1000 | Motif-0 | -59 | + | TCACTATCCGATCCTC |
|  |  |  | Motif-1 | -779 | + | TACATGCATT |
| P. infestans | PITG\_02131 | 1000 | Motif-4 | -38 | + | TCTCATTGGTCAAAT |
| P. infestans | PITG\_02135 | 173 | Motif-0 | -149 | - | TCACAAGGCAACTTGC |
|  |  |  | Motif-17 | -58 | - | TGTGTAGCCATATCG |
| P. infestans | PITG\_02136 | 173 | Motif-0 | -40 | + | TCACAAGGCAACTTGC |
|  |  |  | Motif-17 | -130 | + | TGTGTAGCCATATCG |
| P. infestans | PITG\_02139 | 851 | Motif-1 | -692 | - | TACAAGTACA |
|  |  |  | Motif-2 | -215 | + | GCTCCAACTTG |
| P. infestans | PITG\_02140 | 851 | Motif-1 | -165 | - | TACTTGTACT |
|  |  |  | Motif-2 | -647 | - | GCTCCAACTTG |
| P. infestans | PITG\_02141 | 1000 | Motif-0 | -638 | + | TCAGTACAAAATTTAC |
|  |  |  | Motif-8 | -35 | + | TTTTTTTTAGCAAAT |
| P. infestans | PITG\_02142 | 1000 | Motif-0 | -622 | - | TGGAATTTAAAAGTGA |
|  |  |  | Motif-4 | -724 | + | TCTAATTGACTAAAA |
| P. infestans | PITG\_02143 | 1000 | Motif-0 | -88 | + | CACTCTTCAATTTGAC |
|  |  |  | Motif-1 | -736 | + | TACATGTAT |
|  |  |  | Motif-2 | -46 | + | TCGCTTCACCATC |
| P. infestans | PITG\_02144 | 1000 | Motif-1 | -135 | + | TGCATGTAC |
|  |  |  | Motif-6 | -54 | + | ACACCCACCACCCCAT |
| P. infestans | PITG\_02145 | 893 | Motif-0 | -716 | - | TCACTGTCCACTTCCC |
| P. infestans | PITG\_02146 | 1000 | Motif-17 | -218 | - | GGTGTAGCCATGTTA |
|  |  |  | Motif-4 | -188 | + | GTTCATTGGGTAAAA |
| P. infestans | PITG\_02148 | 404 | Motif-0 | -51 | - | CCCAATTCGAGAGTGA |
| P. infestans | PITG\_02152 | 1000 | Motif-1 | -247 | + | TATAAATGTATAGAA |
|  |  |  | Motif-3 | -333 | + | TACAAATTTCAAATGA |
|  |  |  | Motif-8 | -167 | + | TTTTTTTTAGTTTTT |
| P. infestans | PITG\_02155 | 1000 | Motif-0 | -28 | - | TCAGATTAAAAAGTGA |
|  |  |  | Motif-1 | -54 | + | TACATGTAG |
|  |  |  | Motif-3 | -332 | - | TACAAATACTAACTGA |
| P. infestans | PITG\_02156 | 430 | Motif-2 | -180 | - | CACTTCAACTT |
|  |  |  | Motif-8 | -333 | - | TTTTTTTTAATTAAT |
| P. infestans | PITG\_02157 | 1000 | Motif-0 | -781 | + | CATTTTTGATCTTACC |
| P. infestans | PITG\_02160 | 1000 | Motif-0 | -35 | - | AAGAAATGGAGAGTGA |
| P. infestans | PITG\_02161 | 1000 | Motif-0 | -513 | - | TCATTATCAGATCTCC |
|  |  |  | Motif-1 | -373 | + | TACATGTAC |
|  |  |  | Motif-2 | -702 | - | CACTTCAACTT |
|  |  |  | Motif-4 | -98 | - | GTTGATTGGCTGAAA |
| P. infestans | PITG\_02162 | 1000 | Motif-0 | -415 | - | TCATTCATCAAGTATC |
|  |  |  | Motif-2 | -498 | + | GAAGTTGACGTT |
|  |  |  | Motif-4 | -144 | + | GCTGATTGGCGAAAA |
|  |  |  | Motif-7 | -878 | - | CACTATTAATA |
| P. infestans | PITG\_02164 | 327 | Motif-17 | -53 | + | TGTGTAGCCATTTTT |
|  |  |  | Motif-2 | -84 | - | CACTTCAACGT |
| P. infestans | PITG\_02165 | 1000 | Motif-0 | -104 | + | CAGTCTTCATTTTACC |
|  |  |  | Motif-1 | -256 | + | TACATGTAC |
|  |  |  | Motif-4 | -830 | + | CTCGATTGGCCGGAA |
| P. infestans | PITG\_02166 | 1000 | Motif-3 | -949 | - | ATACACATTTTAAGAC |
| P. infestans | PITG\_02167 | 1000 | Motif-0 | -220 | - | TTATTTCTGACTTTGC |
| P. infestans | PITG\_02168 | 1000 | Motif-0 | -79 | + | CATTCTTCAATTTCCG |
|  |  |  | Motif-3 | -723 | + | TTCAGATCCCAAGTCA |
| P. infestans | PITG\_02169 | 1000 | Motif-0 | -79 | + | CATTCTTCAATTTCCG |
|  |  |  | Motif-3 | -722 | + | TTCAGATCCCAAGTCA |
| P. infestans | PITG\_02172 | 1000 | Motif-0 | -234 | - | TCAGTCTTACATCTAC |
|  |  |  | Motif-8 | -216 | + | TATTTATAAGTAACT |
| P. infestans | PITG\_02174 | 1000 | Motif-0 | -47 | + | TCACTTCGGATTTTTT |
|  |  |  | Motif-1 | -180 | + | TACATGTAT |
|  |  |  | Motif-2 | -161 | + | ACTGCAACACG |
|  |  |  | Motif-9 | -698 | - | ATGTACCGGTA |
| P. infestans | PITG\_02175 | 884 | Motif-0 | -48 | + | CATTTCGGAATTTGCT |
|  |  |  | Motif-1 | -160 | + | TACATGTAC |
|  |  |  | Motif-9 | -377 | - | CAGTACCGGTA |
| P. infestans | PITG\_02177 | 1000 | Motif-8 | -76 | + | TTTTAATAATTTAAA |
| P. infestans | PITG\_02178 | 1000 | Motif-1 | -24 | + | TACATGTAA |
| P. infestans | PITG\_02182 | 1000 | Motif-0 | -72 | + | GCAGTCTCTAATCTGC |
|  |  |  | Motif-1 | -125 | - | ATTACCGGTACCGGTA |
|  |  |  | Motif-6 | -466 | + | CCCCCCCCCACCGCCT |
|  |  |  | Motif-9 | -122 | + | CGGTACCGGTA |
| P. infestans | PITG\_02183 | 239 | Motif-0 | -32 | + | TCATTGTCAAATTAGA |
| P. infestans | PITG\_02185 | 1000 | Motif-0 | -587 | + | CACTCTCCATTTTGTT |
|  |  |  | Motif-1 | -371 | - | ATTATATGTAC |
|  |  |  | Motif-4 | -124 | + | GGTGATTGGGTAAAA |
| P. infestans | PITG\_02188 | 1000 | Motif-0 | -126 | - | TCATTCGGCTTTCTGC |
|  |  |  | Motif-6 | -165 | - | AGCGCCCCCCCCCCCC |
| P. infestans | PITG\_02192 | 1000 | Motif-0 | -34 | + | TCATTCCGTCTTTCTC |
|  |  |  | Motif-4 | -79 | - | GTTGATTGGTCAAAA |
| P. infestans | PITG\_02193 | 443 | Motif-4 | -147 | - | GTCTATTGGTTAAAA |
| P. infestans | PITG\_02194 | 1000 | Motif-3 | -20 | - | GACTTTCTTTTTGTAA |
|  |  |  | Motif-4 | -246 | - | TGTCATTGGTCAATA |
| P. infestans | PITG\_02197 | 384 | Motif-18 | -253 | + | CGGTGTGGCGTACATA |
| P. infestans | PITG\_02198 | 384 | Motif-18 | -147 | - | CGGTGTGGCGTACATA |
| P. infestans | PITG\_02199 | 1000 | Motif-1 | -597 | + | TACATGTAT |
| P. infestans | PITG\_02200 | 1000 | Motif-0 | -104 | + | CATTCTTCAATTCGCG |
|  |  |  | Motif-1 | -697 | + | TACATGCAC |
|  |  |  | Motif-7 | -931 | + | TATTATTAATA |
|  |  |  | Motif-8 | -931 | - | TTTTTATTAATAATA |
| P. infestans | PITG\_02202 | 1000 | Motif-9 | -88 | + | GAGTACCGGTA |
| P. infestans | PITG\_02203 | 1000 | Motif-1 | -448 | - | TACATGCATT |
| P. infestans | PITG\_02205 | 115 | Motif-4 | -63 | + | TCTGATTCGTCAACA |
| P. infestans | PITG\_02206 | 115 | Motif-4 | -67 | - | TCTGATTCGTCAACA |
| P. infestans | PITG\_02207 | 1000 | Motif-1 | -515 | + | TACATGTAT |
|  |  |  | Motif-17 | -212 | - | AATGTAGCCATTTGT |
|  |  |  | Motif-2 | -494 | + | GTGGTTGAAGTG |
|  |  |  | Motif-3 | -949 | + | ACTCAAATTTTGTAT |
| P. infestans | PITG\_02208 | 1000 | Motif-1 | -741 | + | TGTACAGGTAT |
|  |  |  | Motif-3 | -567 | - | AACTCAAATTTTGTAT |
|  |  |  | Motif-4 | -365 | - | TGTGATTGGTAGAAA |
| P. infestans | PITG\_02210 | 645 | Motif-0 | -334 | - | TCAAGTTGGTAAATGA |
|  |  |  | Motif-4 | -552 | - | TTTGATTGGTTCATA |
| P. infestans | PITG\_02211 | 645 | Motif-0 | -326 | - | TCATTTACCAACTTG |
|  |  |  | Motif-4 | -108 | + | TTTGATTGGTTCATA |
| P. infestans | PITG\_02213 | 1000 | Motif-1 | -99 | + | ACTGTACATGGACT |
| P. infestans | PITG\_02218 | 1000 | Motif-4 | -266 | + | ATTGATTGGCCAAAA |
| P. infestans | PITG\_02221 | 621 | Motif-0 | -213 | - | GAAAATAGCAAAGTGA |
| P. infestans | PITG\_02222 | 621 | Motif-0 | -38 | + | CATTTCCGAATTCGTC |
| P. infestans | PITG\_02223 | 652 | Motif-1 | -516 | + | TGCATGTAGACACCC |
| P. infestans | PITG\_02224 | 1000 | Motif-0 | -967 | + | GTCAAATGAGCAGTGA |
| P. infestans | PITG\_02226 | 1000 | Motif-0 | -160 | + | TCACAATTCAATTCGT |
|  |  |  | Motif-1 | -692 | + | TACCTGTAGCTGTAC |
|  |  |  | Motif-2 | -399 | - | CACTTCAACCT |
|  |  |  | Motif-4 | -332 | + | TTGGATTGGCTAAAA |
| P. infestans | PITG\_02227 | 1000 | Motif-0 | -567 | - | GAAGATGCCAAAGTGA |
|  |  |  | Motif-2 | -744 | + | ACTTCAACTTG |
|  |  |  | Motif-3 | -361 | - | CACTTATGCTTTGTAT |
|  |  |  | Motif-4 | -396 | - | CTCGATTGGCTCAAT |
| P. infestans | PITG\_02230 | 1000 | Motif-1 | -858 | + | TACATGTAC |
|  |  |  | Motif-2 | -996 | + | GCTTCAACTCG |
|  |  |  | Motif-9 | -855 | + | ATGTACCGGTA |
| P. infestans | PITG\_02231 | 1000 | Motif-1 | -210 | + | TACATGTAT |
|  |  |  | Motif-2 | -75 | - | GCTTCAACTCG |
|  |  |  | Motif-9 | -216 | - | ATGTACCGGTA |
| P. infestans | PITG\_02232 | 158 | Motif-0 | -74 | + | TCTGTTTTCAATTTGA |
|  |  |  | Motif-2 | -41 | - | GATGTTGACGTT |
| P. infestans | PITG\_02233 | 158 | Motif-0 | -100 | - | TCTGTTTTCAATTTGA |
|  |  |  | Motif-2 | -129 | + | GATGTTGACGTT |
| P. infestans | PITG\_02234 | 1000 | Motif-2 | -104 | + | CCACTTCACCGTC |
| P. infestans | PITG\_02237 | 1000 | Motif-1 | -992 | + | TGCATGTATCGGGCT |
|  |  |  | Motif-3 | -256 | - | AATACAAAATCTAACT |
|  |  |  | Motif-4 | -85 | - | AGCGATTGGACAAAA |
|  |  |  | Motif-8 | -976 | - | TATTTTTAAACTTGA |
| P. infestans | PITG\_02238 | 379 | Motif-0 | -80 | + | TCACTCACGTATTTCA |
|  |  |  | Motif-4 | -134 | - | TACGATTGGAGGAAA |
| P. infestans | PITG\_02239 | 379 | Motif-0 | -315 | - | TCACTCACGTATTTCA |
|  |  |  | Motif-4 | -260 | + | TACGATTGGAGGAAA |
| P. infestans | PITG\_02240 | 164 | Motif-1 | -153 | - | TACATGTGTA |
| P. infestans | PITG\_02242 | 351 | Motif-2 | -218 | - | CACTGCAACCCGGA |
|  |  |  | Motif-3 | -295 | - | ACTTGGATAAAGTAT |
| P. infestans | PITG\_02243 | 351 | Motif-2 | -147 | + | CACTGCAACCCGGA |
|  |  |  | Motif-3 | -71 | + | ACTTGGATAAAGTAT |
| P. infestans | PITG\_02244 | 252 | Motif-0 | -157 | - | ACATTCTTCAACTTTC |
| P. infestans | PITG\_02245 | 252 | Motif-0 | -111 | + | ACATTCTTCAACTTTC |
| P. infestans | PITG\_02246 | 193 | Motif-0 | -32 | + | TCATTTACGATTGTGC |
|  |  |  | Motif-2 | -112 | + | ACTTCAAGTTG |
| P. infestans | PITG\_02247 | 446 | Motif-17 | -116 | + | AATGTACCCATCCAT |
|  |  |  | Motif-2 | -158 | - | CGCTTCAACTT |
| P. infestans | PITG\_02248 | 446 | Motif-17 | -345 | - | AATGTACCCATCCAT |
|  |  |  | Motif-2 | -298 | + | GCTTCAACTTG |
| P. infestans | PITG\_02249 | 448 | Motif-1 | -76 | + | TGCTTCTACATGTTC |
| P. infestans | PITG\_02252 | 1000 | Motif-3 | -532 | + | TACTGAACCCAAGTCA |
| P. infestans | PITG\_02253 | 315 | Motif-0 | -33 | - | GGGAATCGACGAGTGA |
|  |  |  | Motif-2 | -82 | - | CCACATCAAGTTGA |
| P. infestans | PITG\_02254 | 315 | Motif-0 | -298 | + | GGGAATCGACGAGTGA |
|  |  |  | Motif-2 | -240 | - | CACTTCAACTT |
| P. infestans | PITG\_02255 | 1000 | Motif-2 | -143 | - | CCCGTTGAAGTG |
|  |  |  | Motif-3 | -165 | + | TACAAAAATCAAGTAA |
|  |  |  | Motif-4 | -214 | + | GCTGATTGGCGGATT |
| P. infestans | PITG\_02257 | 1000 | Motif-0 | -897 | + | TCATTTTCGAGCTCGA |
|  |  |  | Motif-2 | -823 | - | CCATTTCAAGATCG |
| P. infestans | PITG\_02263 | 297 | Motif-1 | -208 | + | TACATGTAG |
|  |  |  | Motif-2 | -24 | - | TGACTTCAACTGCG |
| P. infestans | PITG\_02264 | 1000 | Motif-1 | -238 | + | AACATGTAC |
|  |  |  | Motif-18 | -82 | - | TAGTGTGGGGTACACA |
| P. infestans | PITG\_02267 | 430 | Motif-2 | -92 | - | TAAATTGAAGTG |
|  |  |  | Motif-4 | -76 | - | GTTGATTGGTTCAAA |
| P. infestans | PITG\_02268 | 430 | Motif-2 | -350 | + | TAAATTGAAGTG |
|  |  |  | Motif-4 | -334 | - | TACCATTGGCAGATA |
| P. infestans | PITG\_02270 | 824 | Motif-0 | -616 | - | TCATTACTCAACTTCA |
| P. infestans | PITG\_02271 | 824 | Motif-0 | -224 | + | TCATTACTCAACTTCA |
| P. infestans | PITG\_02272 | 541 | Motif-0 | -422 | + | TCACTCCTCCTCTTCT |
| P. infestans | PITG\_02273 | 541 | Motif-0 | -35 | - | GAGACTTTAGAAATGA |
| P. infestans | PITG\_02274 | 217 | Motif-0 | -86 | + | TCATTTTTGAATCGAA |
|  |  |  | Motif-2 | -135 | + | ACTTCAACTTG |
| P. infestans | PITG\_02277 | 1000 | Motif-1 | -356 | + | TGCATGTATTATCAT |
|  |  |  | Motif-6 | -662 | + | CCCCCCCCCCCCAAAA |
|  |  |  | Motif-9 | -782 | + | CAGTACCGGTA |
| P. infestans | PITG\_02280 | 1000 | Motif-1 | -48 | - | TACATGAAC |
|  |  |  | Motif-4 | -265 | + | TCTGAATGGATGAAT |
| P. infestans | PITG\_02281 | 192 | Motif-0 | -147 | + | GTCGATTCGGAAGTGA |
|  |  |  | Motif-2 | -107 | + | CGCGTTGAAGTG |
|  |  |  | Motif-4 | -78 | + | TTTGATTGGTTTATT |
| P. infestans | PITG\_02282 | 192 | Motif-0 | -61 | - | GTCGATTCGGAAGTGA |
|  |  |  | Motif-2 | -96 | + | ACTTCAACGCG |
|  |  |  | Motif-4 | -129 | - | TTTGATTGGTTTATT |
| P. infestans | PITG\_02283 | 1000 | Motif-4 | -220 | + | TCTGATTGGCTATAA |
| P. infestans | PITG\_02284 | 1000 | Motif-0 | -50 | + | GCATTCTTCAAGTAGC |
| P. infestans | PITG\_02287 | 1000 | Motif-1 | -484 | + | TACCTGTATCATTCT |
|  |  |  | Motif-6 | -117 | + | CTCCCCCCCTCCCCCT |
| P. infestans | PITG\_02288 | 1000 | Motif-1 | -541 | + | TGCATGTAC |
|  |  |  | Motif-4 | -404 | - | CTCGATTGGCTACAA |
| P. infestans | PITG\_02289 | 165 | Motif-0 | -52 | + | TCACTCGCCAAGTTGA |
|  |  |  | Motif-2 | -44 | - | ACGTCAACTTG |
|  |  |  | Motif-4 | -101 | - | CCTTATTGGCTAAAT |
| P. infestans | PITG\_02290 | 165 | Motif-0 | -129 | - | TCACTCGCCAAGTTGA |
|  |  |  | Motif-2 | -132 | + | ACGTCAACTTG |
|  |  |  | Motif-4 | -55 | - | CATGATTGGTCAAAA |
| P. infestans | PITG\_02291 | 329 | Motif-17 | -216 | - | AATGTAGCCATCTCT |
| P. infestans | PITG\_02292 | 268 | Motif-2 | -91 | - | AACTTCAACCT |
|  |  |  | Motif-4 | -155 | + | CATGATTGGCCAGAA |
| P. infestans | PITG\_02293 | 343 | Motif-1 | -323 | - | GGTACCTGTAACTGCA |
| P. infestans | PITG\_02294 | 343 | Motif-1 | -35 | - | TGCAGTTACAGGTAC |
| P. infestans | PITG\_02302 | 1000 | Motif-0 | -30 | - | GCAGGTTCTGGAATGA |
|  |  |  | Motif-4 | -217 | + | TCTCATTGGATAATA |
| P. infestans | PITG\_02303 | 1000 | Motif-0 | -69 | + | CATTTCCAAATTGGCC |
|  |  |  | Motif-2 | -620 | + | ACTTCAACGTAAACTT |
|  |  |  | Motif-4 | -187 | - | GCTGATTGGCTGCTA |
| P. infestans | PITG\_02304 | 227 | Motif-0 | -158 | - | TCATTTACTAATCCAC |
| P. infestans | PITG\_02305 | 325 | Motif-1 | -225 | + | TACATGTAT |
|  |  |  | Motif-2 | -279 | - | GGACAAGCTGAAGCCA |
|  |  |  | Motif-4 | -74 | - | CCTCATTGGTCAAAA |
| P. infestans | PITG\_02306 | 325 | Motif-1 | -108 | + | TACATGTAG |
|  |  |  | Motif-2 | -40 | + | ACTTCAACGTC |
|  |  |  | Motif-4 | -250 | - | TCTGATTGGATACAA |
| P. infestans | PITG\_02307 | 640 | Motif-1 | -303 | + | TACATGTAT |
|  |  |  | Motif-6 | -326 | + | CCCCCCCCCCCCAACG |
| P. infestans | PITG\_02308 | 1000 | Motif-0 | -187 | - | TCGAAATCAGAAATGA |
|  |  |  | Motif-2 | -516 | - | AGCTTCAACAT |
| P. infestans | PITG\_02310 | 1000 | Motif-1 | -778 | + | TACATGTGTTGATTC |
| P. infestans | PITG\_02311 | 1000 | Motif-0 | -181 | + | CATTCTTCAATTTACT |
|  |  |  | Motif-7 | -620 | + | TATTATTAATA |
| P. infestans | PITG\_02314 | 1000 | Motif-0 | -419 | - | ATTTTATTTTTATTTT |
|  |  |  | Motif-1 | -282 | + | TACATGTTTTTGGCC |
|  |  |  | Motif-8 | -398 | + | TTTTATTTATTTATA |
| P. infestans | PITG\_02315 | 1000 | Motif-0 | -185 | + | GAAAATCAAAGAATGA |
| P. infestans | PITG\_02317 | 1000 | Motif-0 | -716 | + | CACTGCCACATGCGCC |
|  |  |  | Motif-1 | -602 | + | ATTATACTTGTAGT |
|  |  |  | Motif-16 | -669 | + | CACTAGTAGCAGCAAC |
|  |  |  | Motif-7 | -628 | - | AAGTATTAATA |
| P. infestans | PITG\_02319 | 1000 | Motif-8 | -534 | + | TTTTTTTTTTTATTT |
| P. infestans | PITG\_02320 | 945 | Motif-0 | -50 | - | GCAAATAGAAGAGTGA |
|  |  |  | Motif-1 | -653 | + | TACATGTAC |
|  |  |  | Motif-3 | -615 | - | CACTTGGATGCTGTAT |
| P. infestans | PITG\_02321 | 945 | Motif-0 | -911 | + | GCAAATAGAAGAGTGA |
|  |  |  | Motif-1 | -300 | + | TACATGTAA |
|  |  |  | Motif-3 | -345 | - | AATACAGCATCCAAGT |
| P. infestans | PITG\_02322 | 276 | Motif-0 | -242 | + | CATTTTGCAATTTGTC |
|  |  |  | Motif-4 | -225 | + | TCTGATTGGTTGAAA |
| P. infestans | PITG\_02323 | 276 | Motif-0 | -48 | - | CCATTTTGCAATTTG |
|  |  |  | Motif-4 | -66 | - | TCTGATTGGTTGAAA |
| P. infestans | PITG\_02325 | 1000 | Motif-2 | -130 | + | TCACCTCAACTTC |
| P. infestans | PITG\_02326 | 554 | Motif-0 | -429 | - | GCGGAATGCAGAGTGA |
|  |  |  | Motif-2 | -450 | - | CGCTTCAACTT |
|  |  |  | Motif-7 | -496 | + | TAATATTAATA |
| P. infestans | PITG\_02327 | 1000 | Motif-3 | -235 | + | ATTGAATTTTAGTAT |
| P. infestans | PITG\_02328 | 600 | Motif-0 | -31 | - | GGAAATTGCGAAATGG |
|  |  |  | Motif-1 | -322 | - | CTATAATACCTGTAC |
| P. infestans | PITG\_02329 | 639 | Motif-0 | -216 | - | TCATTATACATTTGCC |
|  |  |  | Motif-1 | -97 | + | TACATGTAA |
|  |  |  | Motif-3 | -441 | + | TCTTATTATTTGTAT |
| P. infestans | PITG\_02330 | 1000 | Motif-4 | -131 | - | ACTGATTGGTCTAAA |
|  |  |  | Motif-8 | -34 | + | TTTTTATTAATAAAA |
| P. infestans | PITG\_02332 | 1000 | Motif-0 | -44 | - | GGAAGATGAAAAATGC |
|  |  |  | Motif-1 | -385 | + | AATACCGGTACTTTA |
|  |  |  | Motif-3 | -223 | + | TTTTGGTGTTTGTAA |
|  |  |  | Motif-8 | -355 | + | TTTTAATTATTATAA |
|  |  |  | Motif-9 | -383 | - | AAGTACCGGTA |
| P. infestans | PITG\_02334 | 384 | Motif-0 | -62 | + | CACTTCTAAACTTGCC |
| P. infestans | PITG\_02335 | 569 | Motif-4 | -229 | - | TATAATTGGTTAAAT |
| P. infestans | PITG\_02336 | 569 | Motif-4 | -355 | + | TATAATTGGTTAAAT |
| P. infestans | PITG\_02338 | 172 | Motif-2 | -69 | - | GGGCAGGCTGAAGCGA |
|  |  |  | Motif-4 | -32 | - | AATGATTGGTCAAAA |
| P. infestans | PITG\_02339 | 679 | Motif-1 | -196 | - | GTACTGTACATGTGC |
|  |  |  | Motif-18 | -66 | - | TTGTGTGGCTAACATA |
| P. infestans | PITG\_02340 | 1000 | Motif-0 | -749 | + | GCAAATTGGATACTGC |
| P. infestans | PITG\_02343 | 424 | Motif-1 | -236 | + | AACATGTAGAGTGAC |
|  |  |  | Motif-2 | -122 | - | GCCTCTTCAAGGTCAC |
|  |  |  | Motif-4 | -335 | + | CCTGATTGGCCAAAT |
| P. infestans | PITG\_02344 | 424 | Motif-1 | -196 | - | ACAACATGTA |
|  |  |  | Motif-2 | -318 | + | GCCTCTTCAAGGTCAC |
|  |  |  | Motif-4 | -104 | - | CCTGATTGGCCAAAT |
| P. infestans | PITG\_02345 | 387 | Motif-4 | -66 | - | TGCGATTGGCTAAAA |
| P. infestans | PITG\_02346 | 387 | Motif-4 | -336 | + | TGCGATTGGCTAAAA |
| P. infestans | PITG\_02347 | 1000 | Motif-0 | -494 | - | TCATTCTCCAAAACTC |
| P. infestans | PITG\_02348 | 1000 | Motif-0 | -281 | - | TCACTCTTCAATTTC |
| P. infestans | PITG\_02349 | 807 | Motif-1 | -431 | + | TACACGTAGTTGTAT |
|  |  |  | Motif-2 | -151 | - | CACTTCGACTT |
| P. infestans | PITG\_02350 | 1000 | Motif-1 | -949 | + | TACATGTAT |
|  |  |  | Motif-17 | -656 | + | ACTGTACCCATTTGA |
|  |  |  | Motif-8 | -579 | + | TTTTAATTATTTAAT |
| P. infestans | PITG\_02351 | 1000 | Motif-1 | -221 | - | CATACGTGTATCACA |
|  |  |  | Motif-2 | -579 | - | GACGTTGAAGCT |
| P. infestans | PITG\_02352 | 1000 | Motif-1 | -590 | - | CATACGTGTATCACA |
|  |  |  | Motif-16 | -807 | - | CAGTAGCGGCAGCAAC |
| P. infestans | PITG\_02353 | 1000 | Motif-1 | -591 | - | CATACGTGTATCACA |
|  |  |  | Motif-16 | -805 | - | CCACAGTAGCGGCAGC |
| P. infestans | PITG\_02354 | 1000 | Motif-1 | -221 | - | CATACGTGTATCACA |
|  |  |  | Motif-2 | -579 | - | GACGTTGAAGCT |
| P. infestans | PITG\_02355 | 1000 | Motif-1 | -221 | - | CATACGTGTATCACA |
|  |  |  | Motif-2 | -579 | - | GACGTTGAAGCT |
| P. infestans | PITG\_02356 | 1000 | Motif-1 | -591 | - | CATACGTGTATCACA |
|  |  |  | Motif-16 | -808 | - | CAGTAGCGGCAGCAAC |
| P. infestans | PITG\_02357 | 1000 | Motif-1 | -221 | - | CATACGTGTATCACA |
|  |  |  | Motif-16 | -435 | - | CCACAGTAGCGGCAGC |
| P. infestans | PITG\_02358 | 1000 | Motif-1 | -221 | - | CATACGTGTATCACA |
|  |  |  | Motif-16 | -438 | - | CAGTAGCGGCAGCAAC |
| P. infestans | PITG\_02359 | 1000 | Motif-1 | -732 | - | CATACGTGTATCACA |
| P. infestans | PITG\_02360 | 1000 | Motif-1 | -221 | - | CATACGTGTATCACA |
|  |  |  | Motif-2 | -579 | - | GACGTTGAAGCT |
| P. infestans | PITG\_02361 | 1000 | Motif-1 | -591 | - | CATACGTGTATCACA |
|  |  |  | Motif-16 | -808 | - | CAGTAGCGGCAGCAAC |
| P. infestans | PITG\_02362 | 1000 | Motif-1 | -579 | - | CATACGTGTATCACA |
|  |  |  | Motif-16 | -796 | - | CAGTAGCGGCAGCAAC |
| P. infestans | PITG\_02364 | 1000 | Motif-0 | -639 | + | CATTTCCAAATTGGCT |
|  |  |  | Motif-2 | -550 | + | ACTTCAACTCG |
|  |  |  | Motif-4 | -772 | - | TTTGATTGGTTAATC |
| P. infestans | PITG\_02365 | 1000 | Motif-1 | -861 | + | AGTACATCTAC |
|  |  |  | Motif-3 | -768 | + | ACTTGGTATTTGTAA |
|  |  |  | Motif-8 | -601 | + | TATATTTTATCTTAA |
| P. infestans | PITG\_02368 | 1000 | Motif-0 | -43 | - | GAGAGTTGGCAAATGA |
|  |  |  | Motif-1 | -359 | + | TACATGTTTAATAGT |
|  |  |  | Motif-4 | -747 | + | TCTGATTGGATGGAA |
| P. infestans | PITG\_02371 | 929 | Motif-2 | -108 | - | CTCTTCAACTT |
| P. infestans | PITG\_02372 | 685 | Motif-2 | -670 | - | CACTTCAACTT |
|  |  |  | Motif-3 | -565 | - | TTACAAAACACAAATG |
|  |  |  | Motif-7 | -609 | + | CATTATTAATA |
| P. infestans | PITG\_02373 | 1000 | Motif-0 | -43 | + | CACTCTTGCATTTGCC |
|  |  |  | Motif-1 | -306 | - | AACATGTAC |
|  |  |  | Motif-4 | -350 | - | TTGCATTGGCTGGAA |
| P. infestans | PITG\_02377 | 1000 | Motif-0 | -64 | - | GCAGATTTAGGAGTGC |
|  |  |  | Motif-1 | -387 | - | CACACATGTACAGTA |
| P. infestans | PITG\_02379 | 1000 | Motif-0 | -296 | - | GTAAATTGAAGAATAA |
|  |  |  | Motif-1 | -333 | + | TGTACCGGTACAGGCG |
|  |  |  | Motif-9 | -331 | - | CTGTACCGGTA |
| P. infestans | PITG\_02381 | 1000 | Motif-0 | -318 | - | GGAGATGGGATAGTGA |
|  |  |  | Motif-3 | -126 | + | ACTGGGGGTTTGTAT |
| P. infestans | PITG\_02383 | 199 | Motif-0 | -45 | - | GCAAAAAGAAGACTGA |
| P. infestans | PITG\_02384 | 1000 | Motif-16 | -256 | + | CCACAGCAGCCGCAAC |
|  |  |  | Motif-3 | -415 | - | ATACGGAGGTCAAGAT |
|  |  |  | Motif-8 | -737 | + | TTTTCTTAAGTAAAA |
| P. infestans | PITG\_02387 | 1000 | Motif-0 | -64 | + | CATTCCGCAATTCGCG |
|  |  |  | Motif-1 | -133 | - | AGCATGTATTAATAC |
| P. infestans | PITG\_02390 | 1000 | Motif-2 | -780 | - | TTATCTTCAACATGTA |
| P. infestans | PITG\_02391 | 1000 | Motif-0 | -432 | + | TCACTCTTCTTCTTTC |
|  |  |  | Motif-8 | -149 | - | TTTTTATTAATAAAA |
| P. infestans | PITG\_02392 | 1000 | Motif-1 | -269 | - | AATTCATGTACCGGTA |
|  |  |  | Motif-2 | -497 | - | AGCTCCAACTT |
|  |  |  | Motif-9 | -269 | - | ATGTACCGGTA |
| P. infestans | PITG\_02393 | 610 | Motif-1 | -184 | + | TACGTGTAC |
| P. infestans | PITG\_02394 | 399 | Motif-1 | -393 | + | ATTAAATGTA |
|  |  |  | Motif-16 | -43 | - | TAACAGCAGCAGCAAC |
| P. infestans | PITG\_02397 | 1000 | Motif-0 | -186 | + | TCAGTCCAAAAATTCC |
|  |  |  | Motif-4 | -520 | + | TGGCATTGGCAGAAA |
|  |  |  | Motif-7 | -828 | + | AATTATTAATA |
|  |  |  | Motif-8 | -832 | - | TATTAATAATTTATT |
| P. infestans | PITG\_02399 | 161 | Motif-2 | -121 | + | GCCGTTGAAGTGGC |
| P. infestans | PITG\_02400 | 772 | Motif-0 | -504 | + | TCATTAGTGATCTTGC |
| P. infestans | PITG\_02401 | 772 | Motif-0 | -46 | - | CCGAATTGGGCAATGA |
| P. infestans | PITG\_02406 | 1000 | Motif-4 | -734 | - | TCTCATGGGTTAATA |
|  |  |  | Motif-6 | -337 | - | CGTACCCCCCCCCCCC |
| P. infestans | PITG\_02407 | 1000 | Motif-0 | -22 | - | GCAATTAGCAAAATGA |
|  |  |  | Motif-1 | -142 | + | TACATGTAT |
|  |  |  | Motif-6 | -518 | + | CCCACCCCCACCTACC |
|  |  |  | Motif-8 | -992 | - | TGTTTTTTAGTAAAA |
| P. infestans | PITG\_02408 | 1000 | Motif-4 | -90 | + | TCCGATTCGCTGAAA |
| P. infestans | PITG\_02409 | 463 | Motif-0 | -77 | + | TCATTCTGCTTTCTTC |
| P. infestans | PITG\_02410 | 463 | Motif-0 | -402 | - | TCATTCTGCTTTCTTC |
| P. infestans | PITG\_02413 | 1000 | Motif-0 | -26 | + | GCACTTTCAGATCTCC |
|  |  |  | Motif-1 | -354 | + | TACATGTAC |
| P. infestans | PITG\_02414 | 1000 | Motif-0 | -348 | - | TCACTTTTCATCCCG |
|  |  |  | Motif-1 | -131 | + | TACATGTAC |
|  |  |  | Motif-16 | -370 | + | GAGCAGGAGGAGCAGC |
| P. infestans | PITG\_02415 | 704 | Motif-0 | -587 | - | TCAGTAGCAAACCTGC |
|  |  |  | Motif-4 | -98 | + | TTCAATTGGTCGAAA |
| P. infestans | PITG\_02416 | 988 | Motif-0 | -175 | - | CCATTTTCGCTTTCAC |
|  |  |  | Motif-16 | -431 | + | CAGCCGGCGCCCCAGC |
| P. infestans | PITG\_02417 | 988 | Motif-0 | -828 | + | CATTTTCGCTTTCACC |
|  |  |  | Motif-16 | -573 | - | CAGCCGGCGCCCCAGC |
| P. infestans | PITG\_02418 | 1000 | Motif-0 | -20 | + | CATTTCTCAATTGACC |
|  |  |  | Motif-3 | -109 | + | CATACAAAACGCCACT |
| P. infestans | PITG\_02423 | 279 | Motif-0 | -60 | - | TCAGGATGAAGAATGA |
|  |  |  | Motif-2 | -72 | + | CTTGTTGAAGCG |
| P. infestans | PITG\_02424 | 279 | Motif-0 | -235 | + | TCAGGATGAAGAATGA |
|  |  |  | Motif-2 | -219 | - | CTTGTTGAAGCG |
| P. infestans | PITG\_02425 | 1000 | Motif-0 | -848 | + | TCACTCTGCAAACCTC |
| P. infestans | PITG\_02426 | 1000 | Motif-0 | -261 | - | TCACTCTGCAAACCTC |
| P. infestans | PITG\_02427 | 558 | Motif-0 | -536 | - | TCACTTTTAAATCGCA |
|  |  |  | Motif-1 | -349 | + | TACTTGTACA |
|  |  |  | Motif-2 | -428 | - | AGCTTCAACAT |
| P. infestans | PITG\_02428 | 558 | Motif-0 | -38 | + | TCACTTTTAAATCGCA |
|  |  |  | Motif-1 | -218 | - | TGATGGTACTTGTAC |
|  |  |  | Motif-2 | -140 | + | GCTTCAACATCAACAC |
| P. infestans | PITG\_02429 | 1000 | Motif-2 | -737 | + | ACTTCAACTTG |
| P. infestans | PITG\_02430 | 1000 | Motif-2 | -349 | - | CACTTCAACTT |
| P. infestans | PITG\_02436 | 1000 | Motif-2 | -613 | + | GCTTCAACATG |
|  |  |  | Motif-4 | -326 | + | TATTATTGGTCATAA |
| P. infestans | PITG\_02441 | 1000 | Motif-3 | -738 | + | GACTTGCTGTTAGCAT |
| P. infestans | PITG\_02446 | 143 | Motif-4 | -64 | - | TCTGATTGGCTGATA |
| P. infestans | PITG\_02447 | 143 | Motif-4 | -94 | + | TCTGATTGGCTGATA |
| P. infestans | PITG\_02448 | 338 | Motif-1 | -84 | + | AATACCGGTACTACA |
|  |  |  | Motif-17 | -146 | - | TGTGTAGCCATTTGT |
| P. infestans | PITG\_02449 | 664 | Motif-2 | -131 | + | CAGCTTGAAGTG |
| P. infestans | PITG\_02450 | 664 | Motif-2 | -545 | - | CAGCTTGAAGTG |
| P. infestans | PITG\_02451 | 306 | Motif-0 | -268 | - | GAAATGTGGAAAATGA |
| P. infestans | PITG\_02454 | 1000 | Motif-0 | -65 | + | CACTTTTGCTTTTGCC |
|  |  |  | Motif-1 | -801 | + | GGCACATGTACATTG |
|  |  |  | Motif-2 | -645 | + | CATGTTGCAGTC |
|  |  |  | Motif-4 | -794 | - | TTTCGACCAATGTAC |
| P. infestans | PITG\_02455 | 1000 | Motif-0 | -479 | - | GCACTTCCTAATTTG |
|  |  |  | Motif-2 | -568 | + | ACTGCAACATG |
| P. infestans | PITG\_02456 | 212 | Motif-2 | -117 | - | GACTTCAACGT |
| P. infestans | PITG\_02457 | 1000 | Motif-2 | -372 | + | ACTTCAACTTG |
| P. infestans | PITG\_02460 | 458 | Motif-2 | -299 | - | CCCGTTGAAGTTGA |
|  |  |  | Motif-3 | -427 | + | CACTCGGTGCTTGTAT |
| P. infestans | PITG\_02461 | 64 | Motif-4 | -36 | - | TTTCATTGGACAAAA |
| P. infestans | PITG\_02462 | 64 | Motif-4 | -43 | + | TTTCATTGGACAAAA |
| P. infestans | PITG\_02463 | 617 | Motif-3 | -69 | + | ATTTGATATTTGTAA |
| P. infestans | PITG\_02466 | 313 | Motif-2 | -225 | + | CCCCCTCAACATG |
|  |  |  | Motif-4 | -15 | - | TTTGATTGGCAAAAA |
| P. infestans | PITG\_02467 | 313 | Motif-2 | -101 | - | CCCCCTCAACATG |
|  |  |  | Motif-4 | -313 | + | TTTGATTGGCAAAAA |
| P. infestans | PITG\_02468 | 1000 | Motif-0 | -49 | - | GAAAAATGAAGAATGG |
|  |  |  | Motif-1 | -65 | + | TGGATCTACACGTTC |
|  |  |  | Motif-2 | -153 | - | GCTGCAACATG |
| P. infestans | PITG\_02469 | 352 | Motif-2 | -289 | - | AAAGTTGAAGCG |
| P. infestans | PITG\_02470 | 352 | Motif-2 | -74 | - | CGCTTCAACTT |
| P. infestans | PITG\_02471 | 116 | Motif-0 | -23 | - | GAAAGTTAAAAAGTGA |
|  |  |  | Motif-2 | -105 | + | ACAGCTTCAACTGCAA |
| P. infestans | PITG\_02472 | 1000 | Motif-0 | -36 | - | GCGGAATGAAAAATGA |
| P. infestans | PITG\_02473 | 1000 | Motif-1 | -661 | + | TACATGTAT |
|  |  |  | Motif-2 | -520 | - | GATCTTGAAGTG |
|  |  |  | Motif-3 | -468 | + | GTCCTGGTGTTTGTAT |
| P. infestans | PITG\_02474 | 704 | Motif-0 | -70 | - | GAAAATTGAAATGTGA |
|  |  |  | Motif-1 | -175 | - | AACTTCTACATGTCC |
|  |  |  | Motif-3 | -303 | - | CACTTGTGGTTTGTAT |
| P. infestans | PITG\_02475 | 704 | Motif-0 | -649 | - | TCACATTTCAATTTT |
|  |  |  | Motif-1 | -544 | + | AACTTCTACATGTCC |
|  |  |  | Motif-3 | -416 | + | ACTTGTGGTTTGTAT |
| P. infestans | PITG\_02476 | 132 | Motif-1 | -56 | + | TACATGTAC |
|  |  |  | Motif-3 | -49 | + | ACCTGATGTTTGTAT |
| P. infestans | PITG\_02477 | 132 | Motif-1 | -84 | + | TACATGTAC |
|  |  |  | Motif-3 | -98 | - | ACCTGATGTTTGTAT |
| P. infestans | PITG\_02478 | 186 | Motif-4 | -82 | - | TTTGATAGGTTGAAA |
| P. infestans | PITG\_02479 | 186 | Motif-4 | -92 | - | CGCGATTGGTTTAAA |
| P. infestans | PITG\_02480 | 1000 | Motif-4 | -116 | + | TATGATTGGCCAGTT |
| P. infestans | PITG\_02482 | 1000 | Motif-1 | -758 | - | ACTGTACCAGTAGT |
| P. infestans | PITG\_02483 | 1000 | Motif-8 | -766 | + | TATTATTAAATATCA |
| P. infestans | PITG\_02487 | 1000 | Motif-0 | -196 | + | TCATTGTAGAACTCGC |
|  |  |  | Motif-2 | -427 | + | GCTTCAACATG |
|  |  |  | Motif-4 | -364 | + | TTTGATTGGCTTAAG |
|  |  |  | Motif-9 | -394 | - | CTGTACCGGTA |
| P. infestans | PITG\_02488 | 1000 | Motif-0 | -897 | - | TCATTGCTGGCTCGGC |
|  |  |  | Motif-1 | -278 | + | TACATGTAA |
|  |  |  | Motif-6 | -393 | - | GCAGCCCCCCCCCCCC |
|  |  |  | Motif-9 | -241 | - | GCGTACCGGTA |
| P. infestans | PITG\_02489 | 1000 | Motif-4 | -51 | + | TCCCATTGGCTAAAA |
|  |  |  | Motif-8 | -135 | - | TTTGTCTAATTTTAT |
| P. infestans | PITG\_02490 | 1000 | Motif-1 | -487 | + | TACATGTAG |
| P. infestans | PITG\_02491 | 1000 | Motif-0 | -211 | - | CCATTTCTCGTTTGGA |
|  |  |  | Motif-8 | -177 | + | TAAAATTAAATATAT |
| P. infestans | PITG\_02492 | 170 | Motif-2 | -43 | - | GGCTTCAACTT |
|  |  |  | Motif-4 | -63 | + | TATCATTGGTGGAAA |
| P. infestans | PITG\_02493 | 170 | Motif-2 | -138 | + | GGCTTCAACTT |
|  |  |  | Motif-4 | -76 | - | TATGATTGGTCAAAA |
| P. infestans | PITG\_02494 | 142 | Motif-18 | -61 | + | CGGTGTGGCGCACATA |
|  |  |  | Motif-2 | -101 | + | AAGTACGTTGAAGTAG |
| P. infestans | PITG\_02495 | 142 | Motif-18 | -97 | - | CGGTGTGGCGCACATA |
|  |  |  | Motif-2 | -55 | + | ACTTCAACGTACTTAA |
| P. infestans | PITG\_02496 | 508 | Motif-2 | -504 | + | ACTTCAACTTG |
| P. infestans | PITG\_02497 | 1000 | Motif-0 | -147 | + | GCACAAGTGAATTTGC |
|  |  |  | Motif-3 | -833 | + | CACTTGACTTCTGTAA |
|  |  |  | Motif-4 | -217 | + | GCCCATTGGATGAAA |
| P. infestans | PITG\_02498 | 475 | Motif-0 | -413 | - | TCATTTACATATTTCA |
|  |  |  | Motif-1 | -321 | + | TACGTGTAC |
|  |  |  | Motif-4 | -194 | - | GGTGATTGGTGGAAA |
| P. infestans | PITG\_02499 | 870 | Motif-1 | -662 | - | ACTACATGAAG |
| P. infestans | PITG\_02500 | 558 | Motif-0 | -506 | + | GAAAATTGTAGACTGG |
|  |  |  | Motif-2 | -38 | - | AACTTCAACAT |
|  |  |  | Motif-4 | -157 | - | CGCGATTGGAGAAAT |
| P. infestans | PITG\_02501 | 558 | Motif-0 | -68 | - | GAAAATTGTAGACTGG |
|  |  |  | Motif-2 | -530 | + | ACTTCAACATG |
|  |  |  | Motif-4 | -416 | + | CGCGATTGGAGAAAT |
| P. infestans | PITG\_02502 | 712 | Motif-2 | -499 | + | CAGCTTCAACTAC |
| P. infestans | PITG\_02503 | 605 | Motif-0 | -234 | - | GGAAGTTGGTGAGTGA |
|  |  |  | Motif-4 | -319 | - | TTTTGGCGAATTGGA |
|  |  |  | Motif-9 | -604 | - | GTGTACCGGTA |
| P. infestans | PITG\_02504 | 720 | Motif-0 | -635 | + | GGAAATCAGAGAATGA |
| P. infestans | PITG\_02505 | 720 | Motif-0 | -101 | - | GGAAATCAGAGAATGA |
| P. infestans | PITG\_02506 | 742 | Motif-2 | -633 | - | CACTTCAACTT |
| P. infestans | PITG\_02510 | 406 | Motif-0 | -68 | - | TCACTTTTCAAAATG |
|  |  |  | Motif-1 | -172 | + | TACATGTAC |
|  |  |  | Motif-2 | -315 | - | CACCTTCAACATG |
|  |  |  | Motif-9 | -169 | + | ATGTACCGGTA |
| P. infestans | PITG\_02519 | 514 | Motif-0 | -87 | + | CACTTTTCCTTTCGCC |
| P. infestans | PITG\_02520 | 1000 | Motif-0 | -813 | + | TTCACCTTCTGTTTT |
|  |  |  | Motif-8 | -108 | + | TTTAATTTTATAAAA |
| P. infestans | PITG\_02521 | 1000 | Motif-0 | -585 | + | GCACTCCAGAATTAGC |
|  |  |  | Motif-4 | -745 | - | TGCGATTGGTTGAAA |
|  |  |  | Motif-6 | -998 | + | CCCCCCCCCTCGTCAG |
| P. infestans | PITG\_02524 | 974 | Motif-1 | -178 | + | ATTACAGGTAT |
|  |  |  | Motif-4 | -46 | - | TTTGATGGGATAAAA |
|  |  |  | Motif-7 | -115 | - | TATTATTAATA |
| P. infestans | PITG\_02525 | 342 | Motif-0 | -99 | + | CATTCCTCAATTTGCT |
| P. infestans | PITG\_02526 | 404 | Motif-3 | -48 | + | TACAAAGAGTAAGTCA |
| P. infestans | PITG\_02527 | 283 | Motif-0 | -27 | - | GAAGATTCCGAAATGG |
|  |  |  | Motif-4 | -74 | - | ACTGATTGGTTGAAA |
| P. infestans | PITG\_02528 | 283 | Motif-0 | -272 | + | GAAGATTCCGAAATGG |
|  |  |  | Motif-4 | -57 | + | GATGATTGGCTGATT |
| P. infestans | PITG\_02529 | 420 | Motif-3 | -409 | + | TCTTGATCACTGTAT |
| P. infestans | PITG\_02532 | 414 | Motif-0 | -236 | + | TCAAAGTGAAAAGTGA |
|  |  |  | Motif-3 | -282 | + | TCTTGGCTTTTGTAA |
| P. infestans | PITG\_02533 | 406 | Motif-17 | -211 | - | AGTGTAGCCATGTAG |
|  |  |  | Motif-3 | -82 | + | GACTTTCCTTTTGTAT |
| P. infestans | PITG\_02535 | 290 | Motif-1 | -258 | + | TACATGTAT |
| P. infestans | PITG\_02536 | 369 | Motif-3 | -155 | + | ACTTGATCAAAGTAT |
| P. infestans | PITG\_02537 | 369 | Motif-3 | -229 | - | ACTTGATCAAAGTAT |
| P. infestans | PITG\_02538 | 578 | Motif-7 | -316 | - | TATTATTAGTA |
| P. infestans | PITG\_02543 | 1000 | Motif-2 | -699 | - | GAAGTTGACGTG |
| P. infestans | PITG\_02544 | 1000 | Motif-0 | -450 | + | TCATTTTCGAAACCGA |
|  |  |  | Motif-1 | -414 | - | AGTATACGTAT |
| P. infestans | PITG\_02545 | 1000 | Motif-0 | -43 | - | TGAAAATGGAAAGTGA |
|  |  |  | Motif-1 | -360 | - | AACATGTAC |
|  |  |  | Motif-3 | -873 | + | ACTTGACTTTTGTAT |
| P. infestans | PITG\_02546 | 258 | Motif-0 | -246 | - | TCATTTCTTCTTCGTC |
| P. infestans | PITG\_02547 | 137 | Motif-3 | -83 | - | GTTTGGCATTTGTAT |
| P. infestans | PITG\_02548 | 137 | Motif-3 | -69 | + | GTTTGGCATTTGTAT |
| P. infestans | PITG\_02552 | 1000 | Motif-0 | -984 | + | GCAGAATGCAGAATGC |
| P. infestans | PITG\_02553 | 1000 | Motif-0 | -395 | + | TCAGTTCTCCACTTGA |
|  |  |  | Motif-3 | -79 | - | TCTTGTTTTTTGTAA |
| P. infestans | PITG\_02555 | 1000 | Motif-16 | -188 | + | CAGTCGCAGCAGCCAC |
| P. infestans | PITG\_02556 | 654 | Motif-15 | -133 | + | AACTTCAACCACTT |
|  |  |  | Motif-2 | -133 | - | GTGGTTGAAGTT |
|  |  |  | Motif-3 | -68 | + | TACTGATTCCAAGTCA |
| P. infestans | PITG\_02557 | 406 | Motif-0 | -17 | + | CCATTCATCAAATTGC |
| P. infestans | PITG\_02558 | 406 | Motif-0 | -405 | - | CCATTCATCAAATTGC |
| P. infestans | PITG\_02559 | 919 | Motif-0 | -46 | + | CATTCTTAATTTTGCT |
|  |  |  | Motif-17 | -196 | + | AATGTAGCCATGTTT |
|  |  |  | Motif-2 | -743 | + | CATCTTGAAGTG |
| P. infestans | PITG\_02560 | 204 | Motif-4 | -116 | + | TACGATTGGATGAAA |
| P. infestans | PITG\_02561 | 204 | Motif-4 | -57 | - | TTAGATTGGCCGAAA |
| P. infestans | PITG\_02562 | 1000 | Motif-0 | -240 | + | TCTGTCCTCGATTCGC |
| P. infestans | PITG\_02565 | 1000 | Motif-0 | -474 | - | TCATTTCTGAAGCTCC |
|  |  |  | Motif-1 | -430 | + | TACATGTAG |
|  |  |  | Motif-3 | -98 | - | ACTTGGGTTCAGTAT |
| P. infestans | PITG\_02566 | 301 | Motif-18 | -216 | - | TGGTGTGGTTTACACA |
|  |  |  | Motif-3 | -158 | + | ACTTGGTCTTTGTAA |
| P. infestans | PITG\_02567 | 301 | Motif-18 | -101 | + | TGGTGTGGTTTACACA |
|  |  |  | Motif-3 | -158 | - | CACTTGGTCTTTGTAA |
| P. infestans | PITG\_02576 | 160 | Motif-2 | -109 | - | CAAGTTTAAGTG |
| P. infestans | PITG\_02577 | 160 | Motif-2 | -63 | + | CAAGTTTAAGTG |
| P. infestans | PITG\_02578 | 261 | Motif-18 | -172 | + | TGATGTGGTGCACATT |
| P. infestans | PITG\_02579 | 261 | Motif-18 | -105 | - | TGATGTGGTGCACATT |
| P. infestans | PITG\_02580 | 549 | Motif-0 | -422 | - | GCAAATCTAAAAATGA |
|  |  |  | Motif-1 | -311 | + | TACACGTATTAATAG |
|  |  |  | Motif-4 | -72 | - | TCTTATTTGATAAAA |
|  |  |  | Motif-7 | -305 | - | TACTATTAATA |
|  |  |  | Motif-8 | -201 | + | TTTTTATAATTTAAA |
| P. infestans | PITG\_02581 | 942 | Motif-0 | -920 | - | GCATTCCGCATTTGTC |
|  |  |  | Motif-1 | -249 | - | TGCATGTAC |
|  |  |  | Motif-2 | -151 | - | AAGGTTGACGTG |
|  |  |  | Motif-8 | -438 | + | TAATATTAAATTTAA |
| P. infestans | PITG\_02582 | 942 | Motif-0 | -38 | + | GCATTCCGCATTTGTC |
|  |  |  | Motif-1 | -234 | + | TACATGTAT |
|  |  |  | Motif-2 | -777 | - | CACTTCAACGT |
|  |  |  | Motif-8 | -519 | - | TAATATTAAATTTAA |
| P. infestans | PITG\_02584 | 1000 | Motif-0 | -651 | - | TCATTACTATATTTAC |
| P. infestans | PITG\_02585 | 928 | Motif-0 | -48 | + | CCATTGTGCACTTCGC |
|  |  |  | Motif-1 | -146 | - | TACAAGTACT |
|  |  |  | Motif-7 | -344 | - | CACTATTAATA |
|  |  |  | Motif-8 | -765 | + | TTTTTTTAAGCTTAA |
| P. infestans | PITG\_02586 | 1000 | Motif-1 | -882 | - | ATTACATGCAT |
|  |  |  | Motif-2 | -786 | - | GCACTTCAAGGCGG |
|  |  |  | Motif-7 | -427 | + | CACTATTAATA |
| P. infestans | PITG\_02587 | 556 | Motif-8 | -390 | + | TTTAATTTAGTAAGA |
| P. infestans | PITG\_02588 | 556 | Motif-8 | -181 | - | TTTAATTTAGTAAGA |
| P. infestans | PITG\_02589 | 1000 | Motif-2 | -584 | - | GAAATTGAAGCTGC |
| P. infestans | PITG\_02590 | 590 | Motif-17 | -464 | + | GCTGTAGCCATATGT |
| P. infestans | PITG\_02591 | 590 | Motif-17 | -141 | - | GCTGTAGCCATATGT |
| P. infestans | PITG\_02592 | 323 | Motif-8 | -168 | + | TTTTTTTAAATAATT |
| P. infestans | PITG\_02594 | 394 | Motif-0 | -49 | + | CACATTCGAATTCGCT |
| P. infestans | PITG\_02595 | 111 | Motif-1 | -41 | - | TACTTGTACA |
|  |  |  | Motif-4 | -70 | - | TCCGATTGTTTAAAA |
| P. infestans | PITG\_02596 | 111 | Motif-1 | -80 | + | TACTTGTACA |
|  |  |  | Motif-4 | -56 | + | TCCGATTGTTTAAAA |
| P. infestans | PITG\_02597 | 290 | Motif-0 | -193 | + | CCATTCCGCGATTGCC |
| P. infestans | PITG\_02598 | 330 | Motif-1 | -154 | + | TACATGTAT |
| P. infestans | PITG\_02599 | 1000 | Motif-0 | -56 | - | GCAGATTGAGGACTGG |
|  |  |  | Motif-1 | -241 | + | TGCATGTAC |
| P. infestans | PITG\_02605 | 1000 | Motif-1 | -22 | - | ACTACATATAG |
|  |  |  | Motif-2 | -424 | - | GAACTTGAAGTT |
|  |  |  | Motif-6 | -530 | - | ACACCCCCCCCCCCGA |
|  |  |  | Motif-8 | -63 | + | TAAATTTAAATAAAT |
| P. infestans | PITG\_02607 | 1000 | Motif-0 | -54 | - | GCAAATTGCAATGTGA |
|  |  |  | Motif-1 | -840 | - | ACTATATGTAA |
|  |  |  | Motif-2 | -17 | + | ATCTTCAACGT |
|  |  |  | Motif-8 | -316 | - | TATAATTTTATTAAA |
| P. infestans | PITG\_02608 | 1000 | Motif-0 | -735 | + | GCGTATCGAAAAGTGA |
|  |  |  | Motif-4 | -66 | + | TGTAATTGGCTAATA |
|  |  |  | Motif-7 | -377 | + | CACTATTAATA |
|  |  |  | Motif-8 | -204 | + | TATTATTATATATTT |
| P. infestans | PITG\_02613 | 1000 | Motif-0 | -64 | - | GGCACTTGAGAAGTGA |
|  |  |  | Motif-2 | -570 | + | TCACTTCACCATA |
| P. infestans | PITG\_02614 | 538 | Motif-0 | -466 | + | CATTTTCCAATTTCTC |
|  |  |  | Motif-1 | -438 | - | AACATGTATTTGTTT |
| P. infestans | PITG\_02615 | 257 | Motif-0 | -51 | + | CACTCTTCAATTGACC |
|  |  |  | Motif-2 | -198 | + | ACTTCAACATG |
| P. infestans | PITG\_02616 | 257 | Motif-0 | -27 | + | ACACTTTGCATTCTGC |
|  |  |  | Motif-2 | -69 | - | CACTTCAACAT |
| P. infestans | PITG\_02617 | 865 | Motif-0 | -602 | + | TCATTTTCGAATAGGA |
|  |  |  | Motif-17 | -547 | - | AGTGTAGCCATGTTC |
|  |  |  | Motif-8 | -78 | + | TTTTTATAATTATAT |
|  |  |  | Motif-9 | -316 | + | CAGTACCGGTA |
| P. infestans | PITG\_02618 | 865 | Motif-0 | -279 | - | TCATTTTCGAATAGGA |
|  |  |  | Motif-17 | -333 | + | AGTGTAGCCATGTTC |
|  |  |  | Motif-8 | -802 | - | TTTTTATAATTATAT |
|  |  |  | Motif-9 | -560 | - | CAGTACCGGTA |
| P. infestans | PITG\_02619 | 360 | Motif-1 | -328 | - | GTTACGTACCTGTTC |
| P. infestans | PITG\_02620 | 389 | Motif-0 | -312 | - | TCACACGCAAACTTGC |
|  |  |  | Motif-4 | -63 | - | TTTGATTCGCCAAAA |
| P. infestans | PITG\_02621 | 736 | Motif-1 | -335 | + | TACATGTAG |
| P. infestans | PITG\_02622 | 736 | Motif-1 | -240 | + | TACATGTAG |
| P. infestans | PITG\_02623 | 337 | Motif-0 | -231 | - | CAAAATTGAAAAATGG |
| P. infestans | PITG\_02624 | 885 | Motif-17 | -818 | + | AGTGTAGCCATGTCA |
|  |  |  | Motif-4 | -748 | + | TCCGATTGGCTATAA |
| P. infestans | PITG\_02625 | 479 | Motif-2 | -370 | - | AACTTCAACAT |
| P. infestans | PITG\_02626 | 1000 | Motif-0 | -782 | + | CACTTGCAATTTCGCG |
|  |  |  | Motif-1 | -583 | + | AACATGTAGTTTTGA |
|  |  |  | Motif-2 | -589 | - | CATGTTCAAGTG |
|  |  |  | Motif-3 | -545 | - | GCTTGATGTTTGTAA |
|  |  |  | Motif-9 | -794 | - | AGGTACCGGTA |
| P. infestans | PITG\_02627 | 211 | Motif-18 | -87 | - | TGGTGTTGGGTACACA |
|  |  |  | Motif-3 | -153 | + | CATACCAAATCCAAAT |
| P. infestans | PITG\_02628 | 427 | Motif-1 | -426 | - | TACATATAC |
|  |  |  | Motif-18 | -162 | - | TCGTGTGGCGTACAAG |
| P. infestans | PITG\_02629 | 427 | Motif-1 | -10 | + | TACATATAC |
|  |  |  | Motif-18 | -281 | + | TCGTGTGGCGTACAAG |
| P. infestans | PITG\_02632 | 1000 | Motif-1 | -721 | + | TACATGTAT |
| P. infestans | PITG\_02634 | 1000 | Motif-6 | -645 | + | CCACCCTCCTCCACAC |
| P. infestans | PITG\_02638 | 1000 | Motif-0 | -201 | - | GAAAAATCTGAAATGA |
|  |  |  | Motif-1 | -330 | - | ATAATACGAGTATT |
|  |  |  | Motif-2 | -61 | + | CCACCTCAACCTG |
| P. infestans | PITG\_02639 | 991 | Motif-0 | -732 | + | CATTTTCAAATTCCCT |
|  |  |  | Motif-8 | -324 | - | TTTTATTTTGTTTTA |
| P. infestans | PITG\_02640 | 1000 | Motif-0 | -608 | + | GGGGATTGCATAGTGA |
| P. infestans | PITG\_02641 | 1000 | Motif-8 | -195 | - | TTTTATTAATTTGTT |
| P. infestans | PITG\_02642 | 254 | Motif-0 | -34 | - | GACACTTGGGGAATGA |
|  |  |  | Motif-2 | -96 | + | CAGTTTGAAGTG |
|  |  |  | Motif-4 | -113 | - | TCCTATTGGTTGAAA |
| P. infestans | PITG\_02643 | 744 | Motif-0 | -363 | - | TCATTATGAAATCGGC |
|  |  |  | Motif-2 | -56 | - | CTCTTCAACGT |
|  |  |  | Motif-4 | -731 | + | GCTGATTGGTTGGAA |
| P. infestans | PITG\_02644 | 744 | Motif-0 | -397 | + | TCATTATGAAATCGGC |
|  |  |  | Motif-2 | -699 | - | CACGTTGAAGAG |
|  |  |  | Motif-4 | -28 | - | GCTGATTGGTTGGAA |
| P. infestans | PITG\_02646 | 277 | Motif-2 | -210 | + | TGAGTTGAAGTTGC |
| P. infestans | PITG\_02647 | 277 | Motif-2 | -81 | - | TGAGTTGAAGTTGC |
| P. infestans | PITG\_02649 | 539 | Motif-4 | -260 | - | TCCAATTGGCCAATT |
| P. infestans | PITG\_02650 | 1000 | Motif-0 | -363 | - | TCACTTTAGATTTGCA |
|  |  |  | Motif-1 | -321 | + | TACATGTAC |
| P. infestans | PITG\_02651 | 1000 | Motif-0 | -41 | + | CATTCTCTAATTCACC |
|  |  |  | Motif-1 | -496 | - | ACTACATCTAC |
|  |  |  | Motif-2 | -789 | - | ACAACTTCAAGTTAAA |
|  |  |  | Motif-3 | -232 | - | CACTTGCAAATTGTAT |
|  |  |  | Motif-4 | -204 | - | TCTGATTGGTCAAAC |
|  |  |  | Motif-7 | -832 | - | AATTATTAATA |
|  |  |  | Motif-9 | -733 | + | ATGTACCGGTA |
| P. infestans | PITG\_02652 | 588 | Motif-18 | -71 | + | TGTGGTGGTGTACATA |
|  |  |  | Motif-9 | -342 | - | CAGTACCGGTA |
| P. infestans | PITG\_02653 | 1000 | Motif-0 | -80 | + | CATTATCAAATTTCCT |
|  |  |  | Motif-1 | -705 | + | TACATGTAG |
|  |  |  | Motif-2 | -169 | - | GACGTTGGAGTG |
| P. infestans | PITG\_02654 | 207 | Motif-2 | -65 | + | ATCGTTGAAGTTGG |
|  |  |  | Motif-8 | -131 | + | TTTTCTTAATTAAAA |
| P. infestans | PITG\_02657 | 410 | Motif-0 | -49 | - | GAAAATTTGGGAATGA |
|  |  |  | Motif-2 | -64 | - | CACTTCAACGT |
| P. infestans | PITG\_02658 | 410 | Motif-0 | -376 | - | TCATTCCCAAATTTT |
|  |  |  | Motif-2 | -356 | + | ACTTCAACGTG |
| P. infestans | PITG\_02659 | 155 | Motif-18 | -22 | + | TGGTGTGGGTTACACT |
|  |  |  | Motif-4 | -99 | - | TTTGATTGGCCAATA |
| P. infestans | PITG\_02660 | 155 | Motif-18 | -149 | - | TGGTGTGGGTTACACT |
|  |  |  | Motif-4 | -71 | + | TTTGATTGGCCAATA |
| P. infestans | PITG\_02662 | 1000 | Motif-1 | -649 | + | TACTAGTACTTGTTT |
|  |  |  | Motif-4 | -187 | + | CTGGATTGGTTGAAA |
| P. infestans | PITG\_02663 | 350 | Motif-4 | -205 | - | TATGATTGGCCGAAA |
| P. infestans | PITG\_02664 | 227 | Motif-0 | -49 | + | TCATTTTGATTTCCTC |
|  |  |  | Motif-4 | -40 | - | GATGATTGGAGGAAA |
| P. infestans | PITG\_02665 | 1000 | Motif-0 | -204 | + | CCATTCCACAAATTGC |
|  |  |  | Motif-1 | -315 | + | TGCATGTAC |
|  |  |  | Motif-2 | -293 | + | ACTGCAACATG |
| P. infestans | PITG\_02666 | 1000 | Motif-4 | -431 | + | AATCAGCCAATTGCA |
| P. infestans | PITG\_02667 | 1000 | Motif-2 | -879 | + | CACTTTGAAGTG |
|  |  |  | Motif-4 | -650 | + | TCTCATTGGCTGATT |
| P. infestans | PITG\_02669 | 1000 | Motif-1 | -346 | + | TACGTGTAC |
|  |  |  | Motif-4 | -928 | + | TCTGATTGGATCGAA |
| P. infestans | PITG\_02674 | 1000 | Motif-3 | -354 | - | TACTTGACTTTTGTTT |
|  |  |  | Motif-8 | -361 | + | TTTTACTAAACAAAA |
| P. infestans | PITG\_02675 | 1000 | Motif-3 | -714 | + | ACTTGACTTTTGTTT |
|  |  |  | Motif-8 | -707 | - | TTTTACTAAACAAAA |
| P. infestans | PITG\_02677 | 930 | Motif-2 | -917 | - | CACTTCAACTT |
|  |  |  | Motif-4 | -885 | - | ATTCATTGGTCGAAA |
|  |  |  | Motif-6 | -815 | + | AGCTCCCCCTCCGCCT |
| P. infestans | PITG\_02679 | 108 | Motif-2 | -95 | + | ACTTCAACTTG |
|  |  |  | Motif-4 | -50 | + | TCTTATTGGCCAAAA |
| P. infestans | PITG\_02680 | 1000 | Motif-0 | -781 | + | GGAGATGGGATAGTGA |
|  |  |  | Motif-17 | -102 | + | GATGTAGCCATCCTT |
| P. infestans | PITG\_02681 | 1000 | Motif-4 | -567 | - | TGCGATTGGTAAAAA |
| P. infestans | PITG\_02683 | 1000 | Motif-0 | -37 | - | GCATTGTGACTTTCGC |
|  |  |  | Motif-1 | -491 | + | TACATGTAG |
|  |  |  | Motif-4 | -112 | + | TTCGATAGGACAAAA |
| P. infestans | PITG\_02684 | 1000 | Motif-1 | -998 | - | CTCTGGTTCCTGTAC |
|  |  |  | Motif-18 | -60 | + | AATTGTGGTGCACACA |
| P. infestans | PITG\_02685 | 714 | Motif-0 | -36 | + | CCACTTTTCAACCGTC |
|  |  |  | Motif-2 | -88 | - | CAACTTCAACAAA |
|  |  |  | Motif-7 | -422 | + | TACTATTAATA |
|  |  |  | Motif-9 | -106 | - | CCGTACCGGTA |
| P. infestans | PITG\_02686 | 875 | Motif-0 | -160 | + | CACTTCTCATTTCCTC |
|  |  |  | Motif-1 | -239 | + | CCCCCGTACGTGTAT |
| P. infestans | PITG\_02687 | 875 | Motif-0 | -731 | - | CACTTCTCATTTCCTC |
|  |  |  | Motif-1 | -137 | + | ACTGTACGTGTATT |
| P. infestans | PITG\_02691 | 1000 | Motif-0 | -94 | + | CAGTTCAAAATTTGCG |
| P. infestans | PITG\_02692 | 1000 | Motif-0 | -61 | - | GCGAAAAGCAGAGTGA |
|  |  |  | Motif-1 | -379 | + | TACATGAAC |
|  |  |  | Motif-2 | -559 | - | ATTGTTGAAGTTAA |
|  |  |  | Motif-9 | -814 | - | CCGTACCGGTA |
| P. infestans | PITG\_02693 | 413 | Motif-2 | -30 | + | GCTTCAACTTCGTCGA |
|  |  |  | Motif-23 | -404 | - | TTCTCAAAGAGGCAAA |
| P. infestans | PITG\_02694 | 413 | Motif-2 | -385 | + | GCAGTTGAAGTGAC |
|  |  |  | Motif-23 | -25 | + | TTCTCAAAGAGGCAAA |
| P. infestans | PITG\_02695 | 215 | Motif-1 | -152 | + | ATAACATGTAAAAGTG |
| P. infestans | PITG\_02696 | 215 | Motif-1 | -73 | - | ATAACATGTA |
| P. infestans | PITG\_02697 | 309 | Motif-4 | -80 | + | TCTCATTGGTCGAAA |
| P. infestans | PITG\_02698 | 1000 | Motif-0 | -245 | + | ATTTAATTGTTGCTTT |
|  |  |  | Motif-2 | -464 | - | GGTCAAGTAGAAGTGA |
| P. infestans | PITG\_02700 | 1000 | Motif-0 | -73 | + | TCATTTTGGTTTTCAC |
|  |  |  | Motif-8 | -827 | - | TTTTTTTTTTTATTT |
| P. infestans | PITG\_02702 | 1000 | Motif-0 | -177 | + | CCAGTTTCACTTTGGC |
|  |  |  | Motif-1 | -296 | + | TACATGTAA |
| P. infestans | PITG\_02704 | 1000 | Motif-0 | -73 | + | TCATTTTGGTTTTCAC |
|  |  |  | Motif-1 | -409 | + | AACATGTAC |
|  |  |  | Motif-2 | -603 | + | TCACTTCAACAGCC |
| P. infestans | PITG\_02706 | 1000 | Motif-0 | -465 | + | CACTTTGACTTTTGCT |
|  |  |  | Motif-1 | -408 | + | TACATGTAT |
| P. infestans | PITG\_02707 | 402 | Motif-1 | -357 | + | TACATGTAC |
| P. infestans | PITG\_02708 | 1000 | Motif-1 | -623 | + | TACATGTAC |
|  |  |  | Motif-18 | -633 | + | TAGTGTTGTGTACATG |
| P. infestans | PITG\_02710 | 596 | Motif-0 | -51 | + | CATTTTGGATTTCGCT |
| P. infestans | PITG\_02711 | 596 | Motif-0 | -560 | + | GCGAAATCCAAAATGG |
| P. infestans | PITG\_02712 | 1000 | Motif-1 | -592 | + | TACACGTATCGCTAT |
|  |  |  | Motif-2 | -14 | + | ACTTCAACACG |
|  |  |  | Motif-7 | -498 | - | AACTATTAATA |
| P. infestans | PITG\_02714 | 390 | Motif-8 | -310 | - | TTTTTTTTAATATAA |
| P. infestans | PITG\_02715 | 390 | Motif-8 | -95 | + | TTTTTTTTAATATAA |
| P. infestans | PITG\_02716 | 333 | Motif-1 | -244 | + | AGTGCAGGTACCGTTA |
|  |  |  | Motif-4 | -128 | + | TTTGATTGGATGAAA |
| P. infestans | PITG\_02717 | 333 | Motif-1 | -105 | - | AGTGCAGGTACCGTTA |
|  |  |  | Motif-4 | -191 | - | AGTGATTGGCTGAAA |
| P. infestans | PITG\_02719 | 1000 | Motif-1 | -592 | + | TACATGTAT |
|  |  |  | Motif-17 | -256 | + | TGTGTAGCCATGTCA |
|  |  |  | Motif-2 | -50 | - | GGCTTCAACTT |
|  |  |  | Motif-7 | -850 | + | TATTATTAATA |
| P. infestans | PITG\_02720 | 1000 | Motif-0 | -43 | + | CACTTGCGCATTTCCG |
|  |  |  | Motif-1 | -554 | + | TACGTGTAC |
|  |  |  | Motif-7 | -607 | - | TATTATTAATA |
| P. infestans | PITG\_02721 | 397 | Motif-0 | -173 | - | CCACTTCGAATTTGCC |
|  |  |  | Motif-1 | -262 | + | ACTACGTGTAG |
| P. infestans | PITG\_02722 | 329 | Motif-7 | -148 | - | TATTATTAATA |
| P. infestans | PITG\_02723 | 1000 | Motif-0 | -432 | - | GCATTCCCCGTCTTGC |
|  |  |  | Motif-17 | -464 | + | AGTGTAGCCATGCGA |
|  |  |  | Motif-4 | -290 | + | CTCTATTGGTCAAAA |
|  |  |  | Motif-7 | -923 | - | TATTATTAATA |
|  |  |  | Motif-8 | -945 | + | TTTATATTAATAATA |
| P. infestans | PITG\_02724 | 1000 | Motif-3 | -985 | - | ACTTAATTTTGGTAT |
| P. infestans | PITG\_02725 | 1000 | Motif-1 | -109 | - | TACAAGTACA |
| P. infestans | PITG\_02728 | 1000 | Motif-1 | -742 | + | TACATGTTTTTGCTT |
|  |  |  | Motif-4 | -668 | - | TGCGATTAGTCAAAA |
|  |  |  | Motif-6 | -19 | - | CCCCCCTCCACCTCGC |
|  |  |  | Motif-7 | -142 | - | CATTATTAATA |
|  |  |  | Motif-8 | -299 | - | TTATTTTAATTTTGA |
| P. infestans | PITG\_02729 | 657 | Motif-4 | -131 | - | GCTGATTGGCTGATT |
| P. infestans | PITG\_02732 | 432 | Motif-0 | -165 | - | TCACTGTGCATTCTGA |
|  |  |  | Motif-3 | -283 | - | GACTTGTGTTGTGTAT |
|  |  |  | Motif-4 | -383 | - | TGCCATTGGACAATT |
| P. infestans | PITG\_02733 | 432 | Motif-0 | -283 | + | TCACTGTGCATTCTGA |
|  |  |  | Motif-3 | -164 | - | CATACACAACACAAGT |
|  |  |  | Motif-4 | -64 | + | TGCCATTGGACAATT |
| P. infestans | PITG\_02736 | 361 | Motif-0 | -88 | + | TCGAAATGAAAACTGA |
|  |  |  | Motif-1 | -158 | + | TACATGTAC |
|  |  |  | Motif-4 | -66 | + | GTCTATTGGTTAAAA |
| P. infestans | PITG\_02737 | 356 | Motif-1 | -271 | + | TACATGTAT |
|  |  |  | Motif-4 | -203 | - | TTTCATTGGCCAATA |
| P. infestans | PITG\_02738 | 530 | Motif-0 | -248 | - | TCAAATTCCAGACTGA |
| P. infestans | PITG\_02739 | 530 | Motif-0 | -298 | + | TCAAATTCCAGACTGA |
| P. infestans | PITG\_02740 | 249 | Motif-3 | -123 | + | TACAAAATCCTAGTCA |
|  |  |  | Motif-4 | -153 | - | TTTGATTGGTCAAAA |
| P. infestans | PITG\_02741 | 1000 | Motif-0 | -22 | - | TCGAATTGTGAAATGA |
|  |  |  | Motif-3 | -46 | + | ACTTGCACTCAGTAT |
|  |  |  | Motif-4 | -336 | - | TGTAGGCCAATTAAA |
| P. infestans | PITG\_02744 | 1000 | Motif-4 | -671 | + | TCTGATTGGACCAAA |
| P. infestans | PITG\_02745 | 1000 | Motif-1 | -176 | - | AATACCTGTAACAGAA |
| P. infestans | PITG\_02748 | 1000 | Motif-0 | -54 | - | GCTGATTGAGAAATGG |
|  |  |  | Motif-1 | -106 | + | TACATGTAC |
|  |  |  | Motif-3 | -762 | - | AACTAACTGCCAGTCA |
|  |  |  | Motif-4 | -822 | + | TTCGATTGGTTGATA |
| P. infestans | PITG\_02749 | 1000 | Motif-0 | -265 | - | CACTCCCACATTTGTG |
|  |  |  | Motif-1 | -249 | + | TACATGTAT |
|  |  |  | Motif-9 | -215 | - | AAGTACCGGTA |
| P. infestans | PITG\_02750 | 1000 | Motif-0 | -372 | - | GCAGCTGGGAAAATGA |
|  |  |  | Motif-1 | -163 | + | TACATGTAG |
|  |  |  | Motif-3 | -184 | - | ACTTGTGCTCAGTAT |
| P. infestans | PITG\_02751 | 271 | Motif-0 | -92 | - | GGAATTCGAAGAGTGA |
|  |  |  | Motif-2 | -163 | - | CTACTTCAACCTCA |
| P. infestans | PITG\_02752 | 271 | Motif-0 | -195 | + | GGAATTCGAAGAGTGA |
|  |  |  | Motif-2 | -120 | + | ACTTCAACCTCAACTA |
| P. infestans | PITG\_02759 | 470 | Motif-2 | -390 | + | ACTTCAACATC |
| P. infestans | PITG\_02760 | 470 | Motif-2 | -90 | - | AACTTCAACAT |
| P. infestans | PITG\_02762 | 1000 | Motif-0 | -672 | - | CCACTTTGCAATGTAC |
|  |  |  | Motif-1 | -382 | + | TACATGTGAGTTTTCC |
|  |  |  | Motif-3 | -39 | - | AACTGGCGCTTTGTAT |
| P. infestans | PITG\_02764 | 1000 | Motif-8 | -52 | + | TAATATTTATTTATT |
| P. infestans | PITG\_02765 | 567 | Motif-4 | -291 | + | TACTATTGGATGAAA |
| P. infestans | PITG\_02766 | 567 | Motif-4 | -233 | + | CTCAATTGGTTGAAA |
| P. infestans | PITG\_02767 | 288 | Motif-4 | -120 | + | TCTCATTGGCTGATC |
| P. infestans | PITG\_02768 | 298 | Motif-1 | -176 | + | ACAACATGCACAGTA |
|  |  |  | Motif-2 | -265 | + | ACTGAAGCTGAAGCGA |
| P. infestans | PITG\_02769 | 298 | Motif-1 | -137 | - | ACAACATGCACAGTA |
|  |  |  | Motif-2 | -49 | - | ACTGAAGCTGAAGCGA |
| P. infestans | PITG\_02770 | 431 | Motif-1 | -134 | + | TGTAAATGTACTTTA |
| P. infestans | PITG\_02772 | 1000 | Motif-0 | -45 | + | CCATTCCTGATTCGGC |
|  |  |  | Motif-3 | -458 | + | TTCGAGAACAAAGTCA |
|  |  |  | Motif-6 | -544 | + | CCCCCCCCCTCCTCAG |
|  |  |  | Motif-7 | -226 | + | AAGTATTAATA |
| P. infestans | PITG\_02774 | 1000 | Motif-0 | -288 | - | GTAAATCCTGAAGTGA |
|  |  |  | Motif-1 | -433 | - | AGCATGTACTAATTC |
|  |  |  | Motif-3 | -817 | + | ACTTGATTTTTGTAA |
| P. infestans | PITG\_02776 | 473 | Motif-0 | -319 | + | TCAATACACAATTTGC |
|  |  |  | Motif-4 | -239 | + | TTTGATTGGCTGATT |
|  |  |  | Motif-8 | -267 | + | CATTTTTAAATAAAA |
| P. infestans | PITG\_02777 | 1000 | Motif-9 | -516 | - | CAGTACCGGTA |
| P. infestans | PITG\_02778 | 1000 | Motif-8 | -146 | - | TACTATTTAATATTA |
| P. infestans | PITG\_02779 | 1000 | Motif-0 | -55 | - | GAAAAGTGAAGAATGG |
|  |  |  | Motif-1 | -446 | + | TACATGTAC |
|  |  |  | Motif-7 | -285 | - | CACTATTAATA |
| P. infestans | PITG\_02780 | 993 | Motif-1 | -63 | + | TACATGTAC |
|  |  |  | Motif-2 | -144 | - | GCACTTCAAGTGCA |
|  |  |  | Motif-4 | -93 | + | TGTGATTGGTGGACA |
| P. infestans | PITG\_02784 | 263 | Motif-1 | -105 | + | ACAACATGTATCTTG |
| P. infestans | PITG\_02785 | 1000 | Motif-2 | -213 | - | GAAGTTCAAGTTGC |
|  |  |  | Motif-4 | -244 | + | TTTGATTGGTAGTAA |
| P. infestans | PITG\_02786 | 1000 | Motif-0 | -63 | - | GTAAGATTCGAAATGA |
|  |  |  | Motif-1 | -564 | + | TACATGTAG |
|  |  |  | Motif-3 | -453 | - | ACTTGCTTTTACTAT |
|  |  |  | Motif-7 | -573 | - | TACTATTAATA |
| P. infestans | PITG\_02787 | 695 | Motif-1 | -277 | - | GATGCATGTAG |
|  |  |  | Motif-3 | -108 | - | CACTTGGAAATTGTAA |
| P. infestans | PITG\_02788 | 222 | Motif-0 | -106 | + | TCATTTCACAACTGGT |
| P. infestans | PITG\_02791 | 342 | Motif-2 | -298 | + | CAATTTGAAGTG |
|  |  |  | Motif-4 | -329 | - | TGCGATTGGCCAAAA |
| P. infestans | PITG\_02792 | 1000 | Motif-7 | -13 | + | CAGTATTAATA |
|  |  |  | Motif-9 | -653 | - | CAGTACCGGTA |
| P. infestans | PITG\_02793 | 667 | Motif-2 | -423 | + | GCTTCAACCTGCACCA |
|  |  |  | Motif-3 | -619 | - | AATACGAAGCTCGAGT |
| P. infestans | PITG\_02794 | 667 | Motif-2 | -255 | + | CAGGTTGAAGCC |
|  |  |  | Motif-3 | -63 | - | GACTCGAGCTTCGTAT |
| P. infestans | PITG\_02795 | 1000 | Motif-0 | -211 | - | TCATTGTTATTTTGGC |
| P. infestans | PITG\_02796 | 1000 | Motif-17 | -977 | + | AGCGTAGCCATGTTG |
|  |  |  | Motif-2 | -972 | + | AGCCATGTTGAAACTG |
| P. infestans | PITG\_02797 | 1000 | Motif-17 | -266 | - | AGCGTAGCCATGTTG |
|  |  |  | Motif-18 | -76 | - | TGGTGTGGTGCACATG |
|  |  |  | Motif-2 | -272 | - | AGCCATGTTGAAACTG |
|  |  |  | Motif-7 | -130 | + | TACTATTAATA |
|  |  |  | Motif-8 | -130 | - | TATTTATTAATAGTA |
| P. infestans | PITG\_02801 | 219 | Motif-4 | -83 | + | TTTCATTGGCTGAAA |
| P. infestans | PITG\_02802 | 219 | Motif-4 | -151 | - | TTTCATTGGCTGAAA |
| P. infestans | PITG\_02803 | 225 | Motif-0 | -153 | - | TCATATTGCAATTTG |
|  |  |  | Motif-1 | -63 | + | TGTACATGTCAA |
| P. infestans | PITG\_02807 | 1000 | Motif-0 | -117 | + | CCACTTTCGTTTTGGC |
|  |  |  | Motif-1 | -186 | + | TACATGTAA |
|  |  |  | Motif-2 | -427 | + | ACTTCAACTCG |
| P. infestans | PITG\_02808 | 1000 | Motif-1 | -231 | + | TACATGTAC |
| P. infestans | PITG\_02810 | 1000 | Motif-2 | -138 | - | TATGTTGACGCG |
|  |  |  | Motif-8 | -720 | + | TTTTTTTTATCAGAA |
| P. infestans | PITG\_02812 | 1000 | Motif-1 | -683 | + | TGCATGTTTTAATTT |
|  |  |  | Motif-17 | -846 | + | AGTGTACCCATCCAA |
|  |  |  | Motif-4 | -765 | + | TTTGATTGGCAAATT |
|  |  |  | Motif-7 | -692 | - | CATTATTAATA |
| P. infestans | PITG\_02813 | 1000 | Motif-1 | -229 | + | TACGTGTAC |
|  |  |  | Motif-3 | -892 | - | TTCAAATTTCAAGTGA |
| P. infestans | PITG\_02814 | 1000 | Motif-0 | -127 | + | TCACTCCGTAACCTGC |
|  |  |  | Motif-1 | -602 | + | ACCGTACAAGTATG |
|  |  |  | Motif-3 | -185 | + | TACAAACTCTGAGACA |
|  |  |  | Motif-4 | -148 | - | CGTGACTGGCTGAAT |
| P. infestans | PITG\_02816 | 1000 | Motif-4 | -517 | + | TCCGATTGGATAAAT |
| P. infestans | PITG\_02818 | 1000 | Motif-1 | -537 | + | TACATGTAG |
|  |  |  | Motif-3 | -275 | + | ACTTGTACTTTGTAT |
|  |  |  | Motif-4 | -625 | + | TTTGATTGGCTAAAA |
|  |  |  | Motif-9 | -496 | - | CAGTACCGGTA |
| P. infestans | PITG\_02820 | 726 | Motif-0 | -25 | + | CACTCCGGAATTCGCC |
|  |  |  | Motif-17 | -665 | + | AGTGTAGCCATGCTG |
|  |  |  | Motif-3 | -113 | + | CACTTTACATTTGTAT |
| P. infestans | PITG\_02821 | 726 | Motif-0 | -716 | + | GCGAATTCCGGAGTGA |
|  |  |  | Motif-17 | -76 | - | AGTGTAGCCATGCTG |
|  |  |  | Motif-3 | -629 | - | CACTTTACATTTGTAT |
| P. infestans | PITG\_02822 | 1000 | Motif-16 | -709 | - | GAGACGCAGCCGCAAC |
| P. infestans | PITG\_02823 | 1000 | Motif-16 | -451 | + | GAGACGCAGCCGCAAC |
| P. infestans | PITG\_02824 | 1000 | Motif-0 | -865 | + | GCAGATTTGGTAGTGA |
| P. infestans | PITG\_02826 | 900 | Motif-1 | -305 | + | ACCATCTACTTGTAG |
|  |  |  | Motif-2 | -35 | + | CACTTCAAAACGAC |
| P. infestans | PITG\_02827 | 1000 | Motif-3 | -27 | - | ACTTAAACTTTGTTT |
|  |  |  | Motif-4 | -596 | + | TTTAATTGGCTATAA |
| P. infestans | PITG\_02830 | 1000 | Motif-0 | -61 | - | GCGGATTTCAAACTGA |
| P. infestans | PITG\_02832 | 1000 | Motif-0 | -585 | - | TCATTCGGGAATCACC |
|  |  |  | Motif-1 | -278 | + | TACATGTAC |
| P. infestans | PITG\_02837 | 256 | Motif-3 | -92 | - | GACACGATATTTGTAT |
| P. infestans | PITG\_02838 | 256 | Motif-3 | -141 | + | TCTTGCTTTTTGTAT |
| P. infestans | PITG\_02839 | 1000 | Motif-0 | -318 | + | TCATTCTTCAATCGAT |
|  |  |  | Motif-1 | -215 | - | CTAGCATACATGTTT |
|  |  |  | Motif-4 | -83 | + | TAGAAACCAATCAAA |
|  |  |  | Motif-8 | -508 | + | TTTTATTTAGCAAAT |
| P. infestans | PITG\_02841 | 318 | Motif-0 | -91 | - | GCCAAAACGAAAGTGA |
|  |  |  | Motif-4 | -105 | + | TCTGATTGGTCAGTT |
| P. infestans | PITG\_02842 | 318 | Motif-0 | -180 | - | GACAGTGGAAGAATGA |
|  |  |  | Motif-4 | -228 | - | TCTGATTGGTCAGTT |
| P. infestans | PITG\_02843 | 1000 | Motif-0 | -43 | + | CACTCTACAATTCGCT |
| P. infestans | PITG\_02845 | 1000 | Motif-0 | -36 | + | CACTTTGGATTTCGCC |
| P. infestans | PITG\_02846 | 475 | Motif-1 | -127 | + | ACAACATGTAGCAGA |
|  |  |  | Motif-2 | -167 | + | GAGATTAAAGTGGC |
| P. infestans | PITG\_02847 | 475 | Motif-1 | -336 | - | AACATGTAC |
|  |  |  | Motif-2 | -322 | - | GAGATTAAAGTGGC |
| P. infestans | PITG\_02848 | 397 | Motif-8 | -317 | - | TTCTTTTTATTAAAA |
| P. infestans | PITG\_02850 | 530 | Motif-4 | -349 | + | TGTGATTGGTGAATT |
|  |  |  | Motif-6 | -515 | - | ACTCCCCCCCCCACAT |
| P. infestans | PITG\_02853 | 238 | Motif-17 | -142 | + | ACTGTAGCCATTTAA |
| P. infestans | PITG\_02854 | 238 | Motif-17 | -111 | - | ACTGTAGCCATTTAA |
| P. infestans | PITG\_02855 | 518 | Motif-2 | -38 | - | CACTTCAACTT |
|  |  |  | Motif-4 | -338 | - | TTGGATTGGCCGAAA |
| P. infestans | PITG\_02856 | 518 | Motif-2 | -490 | + | ACTTCAACTTG |
|  |  |  | Motif-4 | -110 | - | TCCGATTAGTCAAAA |
| P. infestans | PITG\_02857 | 535 | Motif-1 | -408 | - | TACTTGTACA |
| P. infestans | PITG\_02858 | 433 | Motif-1 | -194 | + | TACATGTAA |
|  |  |  | Motif-2 | -48 | - | GGGGAGGCTGAAGTGC |
| P. infestans | PITG\_02859 | 1000 | Motif-0 | -57 | + | TCACTCTTCATCGCCC |
|  |  |  | Motif-2 | -41 | + | CCTTCAACTTAAGCGA |
| P. infestans | PITG\_02860 | 1000 | Motif-0 | -49 | - | AAGAATTGAAGAATGA |
|  |  |  | Motif-1 | -406 | - | TACGTGTAC |
|  |  |  | Motif-7 | -786 | + | CACTATTAATA |
|  |  |  | Motif-9 | -539 | - | ATGTTCCGGTA |
| P. infestans | PITG\_02861 | 1000 | Motif-4 | -223 | + | TCTGACTGGCTAAAA |
| P. infestans | PITG\_02863 | 1000 | Motif-4 | -686 | + | GTCGATTGGTTGAAA |
|  |  |  | Motif-8 | -265 | + | TATTTATTAGTTTTA |
| P. infestans | PITG\_02864 | 1000 | Motif-4 | -52 | + | ACTGATTGGCTGATA |
| P. infestans | PITG\_02865 | 1000 | Motif-2 | -528 | - | CAAATTGAAGTG |
|  |  |  | Motif-8 | -685 | - | TATTATTTAACTATT |
| P. infestans | PITG\_02866 | 285 | Motif-4 | -257 | + | TCCGATTGGCTAATT |
| P. infestans | PITG\_02867 | 285 | Motif-4 | -43 | - | TCCGATTGGCTAATT |
| P. infestans | PITG\_02869 | 1000 | Motif-0 | -64 | - | TAAAAATGTGAAATGA |
|  |  |  | Motif-8 | -519 | + | TTTATTTAATCTAAT |
| P. infestans | PITG\_02885 | 1000 | Motif-1 | -116 | + | TACATGTAA |
| P. infestans | PITG\_02887 | 1000 | Motif-0 | -414 | + | TCAGTCCTATTTTTTC |
|  |  |  | Motif-18 | -82 | - | TGGTGTGGCGCACAAA |
|  |  |  | Motif-2 | -40 | + | CAAGTTGCAGCG |
| P. infestans | PITG\_02889 | 110 | Motif-17 | -97 | + | AATGTAGCCATTTTA |
| P. infestans | PITG\_02893 | 732 | Motif-1 | -490 | + | GCATTTTACCTGTAC |
| P. infestans | PITG\_02894 | 732 | Motif-1 | -257 | - | GCATTTTACCTGTAC |
| P. infestans | PITG\_02895 | 1000 | Motif-1 | -257 | - | GCATTTTACCTGTAC |
| P. infestans | PITG\_02897 | 1000 | Motif-0 | -624 | - | TCATTTTGGACCTTCC |
| P. infestans | PITG\_02899 | 1000 | Motif-1 | -137 | + | TACACGTACAGATGG |
| P. infestans | PITG\_02900 | 1000 | Motif-0 | -687 | + | GTAGATTTAAGACTGA |
|  |  |  | Motif-7 | -299 | - | AATTATTAATA |
|  |  |  | Motif-8 | -669 | + | TATTTATAAGTAAGA |
| P. infestans | PITG\_02901 | 1000 | Motif-0 | -589 | + | GTAGATTAAAAAATGG |
|  |  |  | Motif-18 | -151 | - | TAGTGTGGTACACACT |
|  |  |  | Motif-2 | -77 | + | AACTTCGACGT |
|  |  |  | Motif-3 | -656 | + | TCTTAATTTTTGTAT |
| P. infestans | PITG\_02905 | 658 | Motif-1 | -570 | + | TACATGTTTGAATAC |
|  |  |  | Motif-2 | -439 | - | TAAATTGAAGTG |
| P. infestans | PITG\_02906 | 658 | Motif-1 | -98 | + | CAAACATGTATAACA |
|  |  |  | Motif-2 | -231 | + | TAAATTGAAGTG |
| P. infestans | PITG\_02909 | 1000 | Motif-0 | -66 | - | TTAAAATTGGAAATGA |
|  |  |  | Motif-1 | -833 | + | TACATGCATA |
|  |  |  | Motif-7 | -514 | - | AAATATTAATA |
|  |  |  | Motif-8 | -521 | - | TATTAATATATAAAA |
| P. infestans | PITG\_02910 | 1000 | Motif-1 | -107 | + | TACATGTGCA |
|  |  |  | Motif-3 | -296 | - | AACTTGATTTTTGTAT |
|  |  |  | Motif-9 | -113 | - | ATGTACCGGTA |
| P. infestans | PITG\_02912 | 364 | Motif-1 | -145 | + | TACATGTAG |
|  |  |  | Motif-2 | -127 | + | ACTTCAACACG |
|  |  |  | Motif-3 | -47 | + | TACTCAATTCAAGTGA |
| P. infestans | PITG\_02916 | 672 | Motif-0 | -22 | - | GCGAATAGCAAAGTGG |
|  |  |  | Motif-2 | -408 | + | ACTTCAACCTAGCCAT |
| P. infestans | PITG\_02917 | 672 | Motif-0 | -666 | + | GCGAATAGCAAAGTGG |
|  |  |  | Motif-2 | -274 | - | CACTTCAACCT |
| P. infestans | PITG\_02920 | 865 | Motif-1 | -548 | - | TACATGTGCG |
| P. infestans | PITG\_02921 | 865 | Motif-1 | -327 | + | TACATGTGCG |
| P. infestans | PITG\_02923 | 1000 | Motif-1 | -509 | + | TACATGTAC |
|  |  |  | Motif-3 | -340 | + | TACAAACACCAAGCCA |
| P. infestans | PITG\_02924 | 1000 | Motif-3 | -143 | + | TACAAAAATCAAGTCA |
|  |  |  | Motif-8 | -58 | - | TTAGTTTTATTTAAA |
| P. infestans | PITG\_02925 | 1000 | Motif-0 | -55 | - | GGAAATCGAGGAATGA |
| P. infestans | PITG\_02927 | 924 | Motif-1 | -816 | - | AACATGTAC |
|  |  |  | Motif-9 | -821 | - | ATGTACCGGTA |
| P. infestans | PITG\_02928 | 1000 | Motif-0 | -65 | + | CACTTCCTCTTTTGCC |
|  |  |  | Motif-1 | -617 | - | GGTACATCTAT |
|  |  |  | Motif-7 | -961 | + | TATTATTAATA |
|  |  |  | Motif-8 | -321 | + | TTTATTTTATCTTAA |
| P. infestans | PITG\_02930 | 1000 | Motif-0 | -52 | + | CACTTTCACATTTGCC |
|  |  |  | Motif-1 | -179 | + | TACATGTAT |
|  |  |  | Motif-6 | -568 | - | CGGCCCCCCCCCCCCC |
|  |  |  | Motif-7 | -868 | - | TATTATTAATA |
|  |  |  | Motif-8 | -898 | + | TTTATCTTAATAAAA |
| P. infestans | PITG\_02935 | 1000 | Motif-0 | -61 | + | CACTTTCACATTTGCC |
| P. infestans | PITG\_02938 | 1000 | Motif-8 | -319 | - | TTTTATTTAACAATT |
| P. infestans | PITG\_02939 | 486 | Motif-8 | -292 | + | TTTATTTTATTAAAA |
| P. infestans | PITG\_02940 | 486 | Motif-8 | -209 | - | TTTATTTTATTAAAA |
| P. infestans | PITG\_02943 | 148 | Motif-18 | -37 | + | TGATGTGGTGCACACG |
|  |  |  | Motif-8 | -54 | - | TTCTTTTTATTTAAT |
| P. infestans | PITG\_02944 | 148 | Motif-18 | -127 | - | TGATGTGGTGCACACG |
|  |  |  | Motif-8 | -105 | - | TTTTATTAAATAAAA |
| P. infestans | PITG\_02945 | 1000 | Motif-0 | -153 | - | GCATTTTCCAACTCAA |
|  |  |  | Motif-1 | -265 | - | AATACCGGTACAATA |
|  |  |  | Motif-8 | -93 | - | TTTTTTTAATTTTAT |
| P. infestans | PITG\_02947 | 1000 | Motif-0 | -528 | + | TCATTTTCGAATCCTT |
|  |  |  | Motif-1 | -630 | + | TACATGTAC |
|  |  |  | Motif-4 | -553 | + | CTTGATTGGCAGATA |
| P. infestans | PITG\_02948 | 163 | Motif-1 | -125 | + | TACATGTAT |
|  |  |  | Motif-2 | -147 | - | CACTTCAACTT |
| P. infestans | PITG\_02949 | 301 | Motif-17 | -228 | - | TATGTAGCCATGTTG |
|  |  |  | Motif-4 | -245 | + | TCCGATTGGTAGAAA |
| P. infestans | PITG\_02950 | 1000 | Motif-0 | -53 | + | CATTTTCGCTTTTGCT |
|  |  |  | Motif-1 | -637 | + | AGTACATGAAG |
|  |  |  | Motif-2 | -546 | + | GCTTCAACATCTACCA |
|  |  |  | Motif-3 | -156 | - | GTTTTGGCCTTTGTAT |
| P. infestans | PITG\_02951 | 1000 | Motif-1 | -312 | - | TGCATGTAGCGGTAT |
|  |  |  | Motif-2 | -815 | - | AGCTTCAACAT |
|  |  |  | Motif-8 | -118 | - | TTATATTTAATAAAT |
| P. infestans | PITG\_02952 | 763 | Motif-0 | -537 | - | GAAAATTGAAGAATAA |
|  |  |  | Motif-17 | -71 | + | AATGTAGCCATGCAT |
|  |  |  | Motif-2 | -687 | + | CAAGTTGACGTG |
|  |  |  | Motif-6 | -603 | - | TGCCCCCCCCCCCCAC |
| P. infestans | PITG\_02953 | 1000 | Motif-1 | -431 | + | TACATGTAT |
|  |  |  | Motif-8 | -748 | - | TAATTTTTATTTTCA |
| P. infestans | PITG\_02954 | 1000 | Motif-0 | -905 | - | TTATTGGTCACTTTGC |
|  |  |  | Motif-1 | -27 | + | TACGTGTAATATTCAG |
|  |  |  | Motif-2 | -424 | + | GTGGTTGAAGCG |
|  |  |  | Motif-3 | -857 | + | TACAAACTATAAGTCA |
|  |  |  | Motif-4 | -324 | + | TTGCAACCAATGGTC |
| P. infestans | PITG\_02956 | 1000 | Motif-0 | -315 | - | TCACTTCCGCTTCGTC |
| P. infestans | PITG\_02957 | 550 | Motif-4 | -401 | + | TATGATTTGCCAAAA |
| P. infestans | PITG\_02958 | 550 | Motif-4 | -164 | - | TATGATTTGCCAAAA |
| P. infestans | PITG\_02959 | 482 | Motif-1 | -468 | + | TACATATAC |
|  |  |  | Motif-16 | -156 | + | CAGAAGCAGCACCAGC |
| P. infestans | PITG\_02960 | 549 | Motif-0 | -528 | + | GAAAGACGGAGAATGA |
|  |  |  | Motif-1 | -146 | + | TACATATAC |
|  |  |  | Motif-2 | -231 | + | ACCGCCTCAACTTCTC |
|  |  |  | Motif-7 | -388 | - | AACTATTAATA |
| P. infestans | PITG\_02961 | 549 | Motif-0 | -37 | - | GAAAGACGGAGAATGA |
|  |  |  | Motif-1 | -412 | - | TACATATAC |
|  |  |  | Motif-2 | -334 | - | ACCGCCTCAACTTCTC |
|  |  |  | Motif-7 | -172 | + | AACTATTAATA |
| P. infestans | PITG\_02963 | 1000 | Motif-4 | -812 | + | TTTCATTGGTTAATA |
| P. infestans | PITG\_02964 | 1000 | Motif-0 | -413 | + | GCATTTTCGGTTCGGC |
|  |  |  | Motif-1 | -364 | + | TACATGTAT |
| P. infestans | PITG\_02968 | 861 | Motif-1 | -98 | + | GGTACCGGTACAGTA |
|  |  |  | Motif-7 | -285 | - | TACTATTAATA |
|  |  |  | Motif-9 | -96 | - | CTGTACCGGTA |
| P. infestans | PITG\_02970 | 1000 | Motif-1 | -267 | + | TACATGTAA |
|  |  |  | Motif-3 | -313 | + | AATACAAATTCTTAGT |
|  |  |  | Motif-8 | -67 | + | TTTTTTTTTATAAAT |
| P. infestans | PITG\_02971 | 1000 | Motif-0 | -250 | - | TCAGTTTGCGTTTTAA |
|  |  |  | Motif-3 | -582 | + | ACTTATAGTTTGTAT |
| P. infestans | PITG\_02972 | 1000 | Motif-0 | -133 | - | TCACTTTGCAATGCGA |
|  |  |  | Motif-7 | -267 | + | AACTATTAATA |
| P. infestans | PITG\_02973 | 461 | Motif-1 | -253 | + | TACATGCATT |
|  |  |  | Motif-2 | -179 | + | CCGCTTCACCGTC |
|  |  |  | Motif-3 | -65 | - | GGCTTGAAGTTTGTAG |
|  |  |  | Motif-4 | -46 | - | GGTGATTGGTCGGAA |
| P. infestans | PITG\_02974 | 150 | Motif-0 | -145 | - | TTAGTCTTCAATTTAA |
|  |  |  | Motif-8 | -150 | + | TTTTTTTAAATTGAA |
| P. infestans | PITG\_02975 | 150 | Motif-0 | -21 | + | TTAGTCTTCAATTTAA |
|  |  |  | Motif-8 | -15 | - | TTTTTTTAAATTGAA |
| P. infestans | PITG\_02976 | 283 | Motif-0 | -20 | + | TAATTTCTAAATTAGC |
| P. infestans | PITG\_02977 | 283 | Motif-0 | -214 | - | CGATTTCTCAAATTGC |
| P. infestans | PITG\_02979 | 196 | Motif-7 | -154 | - | CATTATTAATA |
| P. infestans | PITG\_02980 | 196 | Motif-7 | -53 | + | CATTATTAATA |
| P. infestans | PITG\_02981 | 1000 | Motif-0 | -749 | + | GTAGATTTAAGACTGA |
|  |  |  | Motif-1 | -186 | + | TACATGTAA |
|  |  |  | Motif-2 | -972 | + | GTCGTTGAAGCG |
|  |  |  | Motif-4 | -418 | + | TATTATTGGGTGAAA |
| P. infestans | PITG\_02984 | 1000 | Motif-0 | -386 | + | TCATTTATCCACTTAC |
|  |  |  | Motif-1 | -120 | - | AAAAAATGTATAGTA |
|  |  |  | Motif-8 | -171 | - | TTTGTTTTAATAAAA |
| P. infestans | PITG\_02985 | 313 | Motif-2 | -107 | + | TCAGTTCAACATCG |
|  |  |  | Motif-3 | -155 | - | CACTGAGTGTTTGTAA |
| P. infestans | PITG\_02986 | 313 | Motif-2 | -220 | - | TCAGTTCAACATCG |
|  |  |  | Motif-3 | -173 | + | ACTGAGTGTTTGTAA |
| P. infestans | PITG\_02989 | 1000 | Motif-8 | -90 | - | TTTTTTTTATTATAT |
| P. infestans | PITG\_02992 | 1000 | Motif-0 | -419 | - | GGAAAATCACGACTGA |
|  |  |  | Motif-1 | -83 | + | TACATGTAT |
|  |  |  | Motif-4 | -142 | - | TGTGAGTGGCTCAAA |
|  |  |  | Motif-7 | -77 | - | TAGTATTAATA |
| P. infestans | PITG\_02993 | 1000 | Motif-17 | -676 | + | AGTGTAGCCATGCGA |
|  |  |  | Motif-4 | -806 | - | CCTGATTGGGCAATA |
| P. infestans | PITG\_02995 | 1000 | Motif-2 | -994 | + | CATATTGAAGCG |
| P. infestans | PITG\_02998 | 1000 | Motif-0 | -40 | - | GCAAGACGAGAAATGA |
|  |  |  | Motif-1 | -349 | + | TACATGTAC |
|  |  |  | Motif-2 | -331 | - | GCTGCAACTTG |
| P. infestans | PITG\_03002 | 272 | Motif-3 | -103 | - | TACTTGTTTTTTGTAA |
| P. infestans | PITG\_03003 | 1000 | Motif-4 | -35 | + | CGGGATTGGTTGAAA |
| P. infestans | PITG\_03004 | 1000 | Motif-0 | -193 | + | TCATTAGCCTTTTTGC |
|  |  |  | Motif-6 | -205 | + | GCCCCCCCCCCCTCAT |
| P. infestans | PITG\_03005 | 1000 | Motif-1 | -775 | + | TACATGTAC |
|  |  |  | Motif-2 | -379 | - | GTCGTTGAAGTG |
| P. infestans | PITG\_03007 | 1000 | Motif-2 | -106 | - | GGCGTTGAAGTT |
| P. infestans | PITG\_03008 | 1000 | Motif-0 | -693 | - | ACAGTTCCGAAATTCC |
|  |  |  | Motif-1 | -864 | + | TACATGTAC |
|  |  |  | Motif-16 | -664 | - | GCGCGGCAGCACCAGC |
|  |  |  | Motif-2 | -114 | - | ACTGCAACTCG |
|  |  |  | Motif-3 | -161 | - | ACTTGGGAATTGTAA |
| P. infestans | PITG\_03009 | 1000 | Motif-2 | -122 | - | TCGTCTTCAACTTGAG |
|  |  |  | Motif-7 | -82 | - | TATTATTAATA |
| P. infestans | PITG\_03011 | 962 | Motif-2 | -42 | - | ATAGCTTCAAGGTGGA |
|  |  |  | Motif-9 | -279 | - | GAGTACCGGTA |
| P. infestans | PITG\_03012 | 1000 | Motif-1 | -183 | + | TACGTGAACATTTTGC |
| P. infestans | PITG\_03014 | 1000 | Motif-1 | -120 | + | TACATGTAC |
|  |  |  | Motif-9 | -114 | - | GTGTACCGGTA |
| P. infestans | PITG\_03015 | 125 | Motif-0 | -114 | - | CCAGTCTTGTTTTGGC |
|  |  |  | Motif-4 | -69 | - | TTCTATTGGTCAAAA |
| P. infestans | PITG\_03016 | 125 | Motif-0 | -27 | + | CCAGTCTTGTTTTGGC |
|  |  |  | Motif-4 | -71 | + | TTCTATTGGTCAAAA |
| P. infestans | PITG\_03018 | 374 | Motif-0 | -135 | + | TCAGTCATCTACTTCC |
| P. infestans | PITG\_03019 | 1000 | Motif-1 | -456 | + | CTTAAATGTACTTTA |
|  |  |  | Motif-2 | -393 | - | GTTGTTGAAGTC |
|  |  |  | Motif-3 | -489 | + | ACTTAGTATTTGTAA |
|  |  |  | Motif-4 | -370 | + | TGTGATTGGCTAAAA |
| P. infestans | PITG\_03020 | 1000 | Motif-0 | -661 | - | TCACTTTTCTTTCTTA |
|  |  |  | Motif-2 | -434 | - | GAAGTTGAAGCT |
|  |  |  | Motif-3 | -104 | + | TACAAAATCCAAGTGA |
|  |  |  | Motif-4 | -200 | + | TATAATTGGTCAAAA |
| P. infestans | PITG\_03024 | 1000 | Motif-0 | -85 | - | TCCTTGTTCAACTTGC |
|  |  |  | Motif-4 | -874 | - | GCTAATTGGTCAAAA |
| P. infestans | PITG\_03026 | 1000 | Motif-1 | -27 | - | TACGTGTAGGAGTTGC |
|  |  |  | Motif-6 | -499 | - | TGCACGCCCTCCTCCC |
| P. infestans | PITG\_03027 | 1000 | Motif-1 | -261 | - | GCCACATGTACATTA |
| P. infestans | PITG\_03028 | 285 | Motif-1 | -131 | - | GATACATAAAC |
| P. infestans | PITG\_03029 | 568 | Motif-0 | -62 | + | TCATTCCAAGTTTTAC |
| P. infestans | PITG\_03030 | 1000 | Motif-16 | -63 | + | GAGCAGGAGCAGGAGC |
| P. infestans | PITG\_03032 | 1000 | Motif-1 | -822 | + | TACTTGTACT |
|  |  |  | Motif-2 | -256 | + | ACAGCTTCAAGTTGGC |
|  |  |  | Motif-8 | -38 | + | TATTTCTAAGTATGA |
| P. infestans | PITG\_03033 | 1000 | Motif-1 | -450 | + | GGTACCGGTACTGTA |
|  |  |  | Motif-9 | -448 | - | CAGTACCGGTA |
| P. infestans | PITG\_03034 | 1000 | Motif-1 | -875 | - | TACATGTGTA |
| P. infestans | PITG\_03036 | 1000 | Motif-0 | -929 | + | TCATTCGACCTTTTGC |
| P. infestans | PITG\_03037 | 967 | Motif-1 | -383 | + | TACATGTAG |
| P. infestans | PITG\_03038 | 967 | Motif-1 | -592 | + | TACATGTAC |
| P. infestans | PITG\_03041 | 1000 | Motif-0 | -368 | - | TCCGAATGAAAAGTGA |
|  |  |  | Motif-1 | -477 | + | GCCACCTACATGTTT |
|  |  |  | Motif-2 | -705 | - | CACTTCAACGT |
| P. infestans | PITG\_03042 | 1000 | Motif-0 | -62 | - | TCCAAATGAAAAGTGA |
|  |  |  | Motif-1 | -431 | + | TACATGTAA |
|  |  |  | Motif-2 | -659 | - | ACTTCAAGTTG |
| P. infestans | PITG\_03043 | 472 | Motif-0 | -371 | + | TCAGTATTTCTTTTTC |
| P. infestans | PITG\_03044 | 1000 | Motif-1 | -746 | + | TACATGTAT |
|  |  |  | Motif-2 | -966 | - | TGACACGCTGAAGTGC |
| P. infestans | PITG\_03045 | 1000 | Motif-0 | -25 | - | GACAATTGCGCAGTGA |
|  |  |  | Motif-1 | -347 | + | TACATGTAC |
|  |  |  | Motif-2 | -135 | + | TGACACGCTGAAGTGC |
| P. infestans | PITG\_03050 | 569 | Motif-0 | -32 | + | TCAGTGTAGATTTTGC |
| P. infestans | PITG\_03051 | 569 | Motif-0 | -88 | + | TCATTCGCAGATCGAC |
| P. infestans | PITG\_03052 | 969 | Motif-0 | -451 | - | GATAATTCAGAAGTGG |
| P. infestans | PITG\_03053 | 887 | Motif-2 | -19 | - | ACTGAGGTTGAAGGCG |
| P. infestans | PITG\_03054 | 1000 | Motif-0 | -454 | + | CCATTCGCCAACACGC |
| P. infestans | PITG\_03055 | 1000 | Motif-0 | -747 | - | GAGAATCCGAAACTGA |
| P. infestans | PITG\_03056 | 1000 | Motif-18 | -393 | + | AGGTGTGGTGCACGCG |
|  |  |  | Motif-4 | -130 | - | TTTTATTTGCTAAAA |
|  |  |  | Motif-8 | -165 | - | TATTTTTTTATTTAA |
| P. infestans | PITG\_03057 | 1000 | Motif-0 | -200 | - | CCATTTCAGAAATTGC |
|  |  |  | Motif-8 | -733 | - | TAATTTTATATTTAA |
| P. infestans | PITG\_03058 | 833 | Motif-0 | -44 | + | TCATTTTCTTTTTGTC |
|  |  |  | Motif-1 | -506 | + | CTTCGGTTCGTGTAT |
| P. infestans | PITG\_03059 | 833 | Motif-0 | -62 | - | GCGAATAGAAAACTGG |
|  |  |  | Motif-1 | -342 | - | CTTCGGTTCGTGTAT |
| P. infestans | PITG\_03060 | 1000 | Motif-1 | -202 | - | GTATGGTACATTTAC |
|  |  |  | Motif-18 | -54 | + | TCATGTGGTGCACGTA |
|  |  |  | Motif-3 | -214 | - | TTACAGAAGCCAAAAC |
| P. infestans | PITG\_03061 | 1000 | Motif-2 | -195 | + | CTGCTTCAACTTCG |
| P. infestans | PITG\_03063 | 486 | Motif-1 | -464 | + | TACATGTTGCCTTGT |
| P. infestans | PITG\_03066 | 603 | Motif-1 | -535 | + | TACTTGTACT |
| P. infestans | PITG\_03067 | 242 | Motif-1 | -94 | + | TACATGTAC |
|  |  |  | Motif-2 | -137 | - | AGCTTCAACTT |
|  |  |  | Motif-7 | -188 | - | TAGTATTAATA |
| P. infestans | PITG\_03068 | 742 | Motif-3 | -492 | - | TCTTATTTTTTGTAT |
|  |  |  | Motif-6 | -152 | + | GCCCCCCCCCCCAAAA |
| P. infestans | PITG\_03069 | 742 | Motif-3 | -265 | + | TCTTATTTTTTGTAT |
|  |  |  | Motif-6 | -46 | + | CCCTCCCCCTCCTCCT |
| P. infestans | PITG\_03070 | 1000 | Motif-17 | -441 | - | AGTGTAGCCATTCGG |
| P. infestans | PITG\_03074 | 186 | Motif-3 | -76 | + | ACTTGGTATTTTTAT |
|  |  |  | Motif-8 | -63 | - | TTAATTTTATTTAAT |
| P. infestans | PITG\_03078 | 1000 | Motif-1 | -482 | + | TACATGTAT |
|  |  |  | Motif-2 | -144 | + | ACTTCAACTTG |
|  |  |  | Motif-3 | -95 | + | TACAAAATCCTAGTCA |
| P. infestans | PITG\_03079 | 1000 | Motif-9 | -492 | + | AAGTACCGGTA |
| P. infestans | PITG\_03080 | 1000 | Motif-1 | -803 | + | TACATGTAA |
|  |  |  | Motif-8 | -733 | - | TTTTTCTAAATTTGA |
| P. infestans | PITG\_03082 | 1000 | Motif-0 | -688 | + | TCATTGATTCATTTGC |
|  |  |  | Motif-1 | -675 | + | TGCATGTAGCAGGCC |
|  |  |  | Motif-4 | -901 | - | TTTGTTTGGTCAAAA |
| P. infestans | PITG\_03083 | 597 | Motif-4 | -78 | - | CAGGATTGGCGGAAA |
| P. infestans | PITG\_03085 | 235 | Motif-16 | -174 | + | GGGTAGCAGCAGCAAC |
|  |  |  | Motif-7 | -159 | + | CAGTATTAATA |
| P. infestans | PITG\_03092 | 1000 | Motif-0 | -969 | - | CCACTTCGCAATTTT |
|  |  |  | Motif-1 | -411 | - | ACGACATGTAC |
|  |  |  | Motif-2 | -482 | + | ACTTCAACTTG |
| P. infestans | PITG\_03094 | 261 | Motif-0 | -42 | + | CATTTTAGCTTTTGTC |
| P. infestans | PITG\_03095 | 450 | Motif-1 | -362 | + | TACATGTAT |
| P. infestans | PITG\_03096 | 450 | Motif-1 | -96 | + | TACATGTAG |
| P. infestans | PITG\_03097 | 1000 | Motif-1 | -133 | + | TACATGTAA |
|  |  |  | Motif-4 | -15 | + | TTTTATTGGCTGTAA |
| P. infestans | PITG\_03098 | 1000 | Motif-4 | -813 | + | TACGATTGGCTGCTA |
|  |  |  | Motif-6 | -313 | + | CCCCCCCCCCCCACGG |
| P. infestans | PITG\_03099 | 1000 | Motif-4 | -571 | - | TACGATTGGCTGCTA |
| P. infestans | PITG\_03102 | 1000 | Motif-2 | -277 | + | GAAATTGAAGCTGA |
|  |  |  | Motif-8 | -391 | - | TATTATTAATTTTTA |
| P. infestans | PITG\_03103 | 433 | Motif-1 | -89 | + | ACTACCGGTACAGCTC |
|  |  |  | Motif-9 | -87 | - | CTGTACCGGTA |
| P. infestans | PITG\_03104 | 433 | Motif-1 | -360 | - | ACTACCGGTACAGCTC |
|  |  |  | Motif-9 | -357 | + | CTGTACCGGTA |
| P. infestans | PITG\_03105 | 271 | Motif-2 | -93 | + | ACTTCAACGTC |
| P. infestans | PITG\_03107 | 1000 | Motif-1 | -347 | + | TACATGTAC |
| P. infestans | PITG\_03108 | 336 | Motif-0 | -75 | + | CACTGTGCAATTTGCC |
| P. infestans | PITG\_03109 | 409 | Motif-2 | -324 | + | ACTTCAACTTG |
|  |  |  | Motif-4 | -314 | + | GCGGATTGGCTGAAA |
| P. infestans | PITG\_03110 | 409 | Motif-2 | -95 | - | CACTTCAACTT |
|  |  |  | Motif-4 | -73 | + | GCTGATTGGTCAAAA |
| P. infestans | PITG\_03111 | 496 | Motif-0 | -28 | + | TTATTCTCCGATCTCC |
| P. infestans | PITG\_03112 | 496 | Motif-0 | -71 | - | GCAACTTGTGGAATGA |
| P. infestans | PITG\_03113 | 186 | Motif-2 | -135 | + | ACTTCAACTTG |
| P. infestans | PITG\_03114 | 559 | Motif-0 | -479 | - | TCACTCTCAATTTTG |
|  |  |  | Motif-1 | -57 | + | TACATGTAC |
|  |  |  | Motif-4 | -116 | + | TGTGATTGGCTGTTA |
| P. infestans | PITG\_03115 | 710 | Motif-3 | -415 | + | TCTTGTTGTTTGTAA |
|  |  |  | Motif-9 | -573 | - | ACGTACCGGTA |
| P. infestans | PITG\_03116 | 710 | Motif-3 | -310 | - | GTCTTGTTGTTTGTAA |
|  |  |  | Motif-9 | -145 | - | CAGTACCGGTA |
| P. infestans | PITG\_03119 | 492 | Motif-18 | -85 | - | TGGTGTGGTGCACAAA |
| P. infestans | PITG\_03120 | 492 | Motif-18 | -423 | + | TGGTGTGGTGCACAAA |
| P. infestans | PITG\_03123 | 1000 | Motif-0 | -637 | - | TCACTTTGCAATTCG |
|  |  |  | Motif-8 | -539 | + | TATATATAAATAAAT |
| P. infestans | PITG\_03125 | 1000 | Motif-4 | -153 | - | GTTTATTGGCTGAAA |
|  |  |  | Motif-6 | -56 | + | CCCCCCCCCCACACAC |
| P. infestans | PITG\_03126 | 1000 | Motif-0 | -410 | - | TCCGTCTCCAACCCTC |
|  |  |  | Motif-6 | -786 | - | TCATCCCCCCCCCTCC |
| P. infestans | PITG\_03127 | 1000 | Motif-0 | -966 | + | GAAAATATCGGAATGA |
|  |  |  | Motif-8 | -851 | + | TTTTATTTTTTAAAT |
| P. infestans | PITG\_03128 | 727 | Motif-0 | -333 | + | GTGAATTGAATAATGG |
|  |  |  | Motif-8 | -152 | - | TTTGATTTAATTTAT |
| P. infestans | PITG\_03129 | 685 | Motif-0 | -163 | - | TCATTGCGGACTCTGC |
| P. infestans | PITG\_03131 | 1000 | Motif-1 | -529 | + | AACATGTAGTTTTCC |
|  |  |  | Motif-2 | -58 | - | ACGAATTCAAGTTGTA |
| P. infestans | PITG\_03135 | 804 | Motif-0 | -46 | - | GAAAATTGGTGAATGC |
|  |  |  | Motif-1 | -522 | + | TACATATAC |
|  |  |  | Motif-2 | -633 | + | ACTACTTCAAGTACTA |
| P. infestans | PITG\_03136 | 1000 | Motif-0 | -93 | + | CACTTTCCAACTTCCT |
|  |  |  | Motif-3 | -333 | + | TACAGAAGTCAAGTCA |
| P. infestans | PITG\_03140 | 1000 | Motif-0 | -216 | - | GAAAATTGAAAACTGA |
|  |  |  | Motif-1 | -876 | + | TACATGTAG |
|  |  |  | Motif-9 | -275 | - | CAGTACCGGTA |
| P. infestans | PITG\_03141 | 363 | Motif-8 | -198 | - | TTTTACTTAACAAAA |
| P. infestans | PITG\_03142 | 363 | Motif-8 | -180 | + | TTTTACTTAACAAAA |
| P. infestans | PITG\_03143 | 208 | Motif-4 | -15 | + | GTGCATTGGCAGAAA |
| P. infestans | PITG\_03144 | 208 | Motif-4 | -208 | - | GTGCATTGGCAGAAA |
| P. infestans | PITG\_03145 | 82 | Motif-1 | -26 | + | TACATGTAG |
| P. infestans | PITG\_03146 | 82 | Motif-1 | -64 | + | TACATGTAC |
| P. infestans | PITG\_03148 | 1000 | Motif-0 | -130 | - | TCATTTGGGTTTTGAC |
|  |  |  | Motif-2 | -151 | + | GCTTCAACCTGTCTCT |
|  |  |  | Motif-4 | -138 | + | TCTGATTGGTCAAAA |
| P. infestans | PITG\_03149 | 1000 | Motif-0 | -96 | + | CACTATCTAATTTGCC |
|  |  |  | Motif-3 | -163 | - | GACTTGTCTTCAGTAT |
|  |  |  | Motif-4 | -523 | - | GTTGATTGGTCACAA |
| P. infestans | PITG\_03150 | 314 | Motif-0 | -171 | - | GCAGTAAGAAATTTGC |
|  |  |  | Motif-2 | -231 | - | CACTTCAACAT |
|  |  |  | Motif-4 | -200 | + | CATGATTGGCAGAAA |
| P. infestans | PITG\_03151 | 772 | Motif-2 | -598 | + | ACTTCAACGTG |
| P. infestans | PITG\_03152 | 926 | Motif-0 | -859 | + | GGAAATTCTCGAGTGA |
| P. infestans | PITG\_03153 | 926 | Motif-0 | -83 | - | GGAAATTCTCGAGTGA |
| P. infestans | PITG\_03154 | 1000 | Motif-3 | -301 | - | ACTTAACGTTAGTTT |
| P. infestans | PITG\_03155 | 863 | Motif-16 | -218 | + | CAGCAGCAGGAGCTAC |
| P. infestans | PITG\_03158 | 363 | Motif-1 | -362 | + | AGTACCTGTAGATGA |
| P. infestans | PITG\_03159 | 333 | Motif-4 | -63 | - | TGTTATTGGTCAAAT |
| P. infestans | PITG\_03160 | 333 | Motif-4 | -285 | + | TGTTATTGGTCAAAT |
| P. infestans | PITG\_03161 | 1000 | Motif-2 | -992 | + | CCAGATCAACTTGA |
| P. infestans | PITG\_03163 | 370 | Motif-18 | -105 | + | TGGTGTGGCGCACACA |
|  |  |  | Motif-4 | -280 | + | ACTTATTGGTCAAAA |
| P. infestans | PITG\_03164 | 370 | Motif-18 | -281 | - | TGGTGTGGCGCACACA |
|  |  |  | Motif-4 | -105 | - | ACTTATTGGTCAAAA |
| P. infestans | PITG\_03165 | 267 | Motif-2 | -158 | + | ACTTCAACTTC |
| P. infestans | PITG\_03166 | 267 | Motif-2 | -119 | - | CACTTCAACTT |
| P. infestans | PITG\_03167 | 82 | Motif-1 | -39 | + | TACATGTAT |
| P. infestans | PITG\_03168 | 82 | Motif-1 | -51 | + | TACATGTAT |
| P. infestans | PITG\_03169 | 1000 | Motif-0 | -75 | + | CACTCGCGCATTTCCT |
|  |  |  | Motif-1 | -221 | + | TACATGTAG |
|  |  |  | Motif-2 | -851 | + | CCATCAACGTGGGTAT |
|  |  |  | Motif-7 | -201 | - | AACTATTAATA |
|  |  |  | Motif-8 | -769 | - | TATTTTTTTTTAATA |
|  |  |  | Motif-9 | -781 | + | CTGTACCGGTA |
| P. infestans | PITG\_03170 | 835 | Motif-9 | -507 | + | AAGTACCGGTA |
| P. infestans | PITG\_03171 | 1000 | Motif-2 | -86 | + | GCGCTTCAACTGCA |
|  |  |  | Motif-6 | -653 | - | TGTGCCCCCCCCCCCT |
| P. infestans | PITG\_03174 | 1000 | Motif-1 | -97 | + | TACGTGTAC |
|  |  |  | Motif-8 | -131 | + | TTTGTTTAAATAAAA |
| P. infestans | PITG\_03175 | 1000 | Motif-0 | -45 | + | CATTCTTCATTTTGTC |
|  |  |  | Motif-1 | -107 | - | AGTAAATGTAC |
| P. infestans | PITG\_03176 | 1000 | Motif-18 | -52 | - | TGATGTGGCGTACATG |
|  |  |  | Motif-2 | -44 | + | CCACATCAACTTTC |
| P. infestans | PITG\_03177 | 234 | Motif-3 | -124 | + | ATTTGGCATTTGTAA |
|  |  |  | Motif-4 | -100 | - | TTCCATTGGCTAAAA |
| P. infestans | PITG\_03178 | 234 | Motif-3 | -125 | - | GATTTGGCATTTGTAA |
|  |  |  | Motif-4 | -149 | + | TTCCATTGGCTAAAA |
| P. infestans | PITG\_03179 | 425 | Motif-0 | -69 | + | CATTTTGGAACTCACT |
|  |  |  | Motif-1 | -96 | + | ACTACATCTAT |
| P. infestans | PITG\_03180 | 425 | Motif-0 | -371 | + | GTGAGTTCCAAAATGA |
|  |  |  | Motif-1 | -163 | + | TACATGTAG |
| P. infestans | PITG\_03182 | 1000 | Motif-0 | -307 | + | CCACTAATCAATTTTC |
| P. infestans | PITG\_03184 | 1000 | Motif-1 | -262 | - | ATAACATGTA |
| P. infestans | PITG\_03185 | 1000 | Motif-0 | -203 | + | GAAAATGTCGGAATGA |
|  |  |  | Motif-3 | -262 | + | ACTTAAATTCAGTAT |
|  |  |  | Motif-4 | -163 | + | TATAATTGGCTAAAT |
| P. infestans | PITG\_03186 | 398 | Motif-0 | -61 | - | GTAAATCGGAGAATGA |
|  |  |  | Motif-17 | -308 | + | AATTTAGCCATCTTA |
| P. infestans | PITG\_03188 | 476 | Motif-16 | -81 | - | CAACAGTAGCAACGGC |
|  |  |  | Motif-2 | -136 | - | GGCTTCAACGT |
| P. infestans | PITG\_03190 | 316 | Motif-0 | -314 | - | ACAAGTTTAAGAATGA |
| P. infestans | PITG\_03192 | 1000 | Motif-0 | -54 | + | TCATTTGACAATCGGC |
|  |  |  | Motif-1 | -306 | + | TACATGTAG |
| P. infestans | PITG\_03196 | 1000 | Motif-0 | -798 | - | GCATTTCGCTATCTGC |
|  |  |  | Motif-7 | -567 | + | TACTATTAATA |
|  |  |  | Motif-8 | -480 | - | TATTTATTATTATTA |
| P. infestans | PITG\_03198 | 711 | Motif-0 | -185 | + | ACACTCGACAATTTGC |
|  |  |  | Motif-16 | -560 | - | CCACAGCAACAGCAGC |
| P. infestans | PITG\_03199 | 711 | Motif-0 | -230 | - | GCCAATCGAGAACTGG |
|  |  |  | Motif-16 | -167 | + | CCACAGCAACAGCAGC |
| P. infestans | PITG\_03205 | 406 | Motif-0 | -276 | - | GCAAATCAAAGAGTGA |
| P. infestans | PITG\_03206 | 406 | Motif-0 | -146 | + | GCAAATCAAAGAGTGA |
| P. infestans | PITG\_03207 | 1000 | Motif-16 | -75 | + | CAACCGCCGCAGCAGA |
|  |  |  | Motif-3 | -258 | + | TTGAAAAACCAAGTCA |
| P. infestans | PITG\_03208 | 1000 | Motif-1 | -154 | - | TACATATAC |
| P. infestans | PITG\_03209 | 1000 | Motif-0 | -36 | + | CACTTTGGAATTCGCC |
| P. infestans | PITG\_03213 | 1000 | Motif-0 | -96 | - | GCATTATGCAACTTGC |
|  |  |  | Motif-1 | -616 | + | TACATGTTTTTCAAT |
|  |  |  | Motif-2 | -120 | - | AGCTTCAACTT |
|  |  |  | Motif-8 | -431 | - | TTTTATTTTTTTTTA |
| P. infestans | PITG\_03214 | 1000 | Motif-0 | -698 | - | ATCAATTCGGAAATGA |
|  |  |  | Motif-1 | -531 | + | TACATGTAC |
|  |  |  | Motif-9 | -801 | - | AGGTACCGGTA |
| P. infestans | PITG\_03215 | 497 | Motif-0 | -67 | + | CACTTTGGAATTTGGT |
|  |  |  | Motif-1 | -287 | - | GTCGTCTACCTGTAC |
| P. infestans | PITG\_03216 | 414 | Motif-0 | -16 | + | ATTTAATTATTACTTT |
|  |  |  | Motif-8 | -19 | + | TTAATTTAATTATTA |
| P. infestans | PITG\_03219 | 155 | Motif-2 | -119 | - | GACTTCAACTT |
| P. infestans | PITG\_03220 | 370 | Motif-4 | -216 | - | GCTGATTGGCTTAAA |
| P. infestans | PITG\_03221 | 370 | Motif-4 | -169 | + | GCTGATTGGCTTAAA |
| P. infestans | PITG\_03224 | 1000 | Motif-1 | -187 | - | TACATGTGCA |
|  |  |  | Motif-2 | -629 | - | ACTGCAAGTTG |
|  |  |  | Motif-4 | -971 | - | TGTGATTGGCTATCA |
| P. infestans | PITG\_03225 | 1000 | Motif-1 | -889 | + | TGCATGTAC |
|  |  |  | Motif-2 | -472 | + | CAAGTTGCAGTG |
|  |  |  | Motif-4 | -138 | + | TGTGATTGGCTATCA |
| P. infestans | PITG\_03226 | 332 | Motif-0 | -62 | + | CAGTCTTCAATTTGCT |
|  |  |  | Motif-7 | -203 | + | AAGTATTAATA |
|  |  |  | Motif-9 | -86 | - | CCGTACCGGTA |
| P. infestans | PITG\_03227 | 1000 | Motif-4 | -75 | - | GCTGATTGGCCAGAT |
| P. infestans | PITG\_03229 | 1000 | Motif-0 | -617 | - | TCATTGCGATATTCTC |
|  |  |  | Motif-1 | -404 | - | TGCATGTTTTAATAT |
|  |  |  | Motif-4 | -586 | - | GCTGATTGGCTGGTA |
| P. infestans | PITG\_03230 | 1000 | Motif-0 | -460 | + | TCATTGCGATATTCTC |
|  |  |  | Motif-1 | -537 | + | TACATGCATT |
|  |  |  | Motif-4 | -490 | + | GCTGATTGGCTGGTA |
| P. infestans | PITG\_03231 | 421 | Motif-0 | -30 | + | CATTTCCGATTTCACC |
|  |  |  | Motif-2 | -45 | + | CCAGTTGAAGTG |
|  |  |  | Motif-4 | -79 | + | CCTGATTGGCTGAAA |
| P. infestans | PITG\_03232 | 803 | Motif-0 | -68 | - | GCAGATCGGAGAATGA |
|  |  |  | Motif-2 | -90 | + | ACTGCAACATC |
| P. infestans | PITG\_03235 | 663 | Motif-1 | -511 | + | AACATGTAC |
|  |  |  | Motif-18 | -594 | - | TAGTGTGGCACACAAA |
|  |  |  | Motif-2 | -630 | - | AACTTCAACGT |
|  |  |  | Motif-3 | -537 | + | ACTTAGTGTTTGTAA |
| P. infestans | PITG\_03236 | 1000 | Motif-4 | -942 | - | TTTGATCGGTTATTT |
| P. infestans | PITG\_03237 | 1000 | Motif-0 | -224 | - | TCATTTTTAAAGTGTC |
|  |  |  | Motif-1 | -254 | + | TACATGTAC |
|  |  |  | Motif-4 | -534 | + | TTTGATCGGTTATTT |
| P. infestans | PITG\_03241 | 184 | Motif-18 | -52 | + | TGGTGTGGTACACATA |
|  |  |  | Motif-2 | -62 | + | GAAGTTGCAGTG |
| P. infestans | PITG\_03242 | 1000 | Motif-0 | -173 | - | GGAGATGGGATAGTGA |
| P. infestans | PITG\_03243 | 791 | Motif-2 | -439 | + | ACTTCAAGGTG |
|  |  |  | Motif-3 | -72 | - | ATCTTGACGTTTGTAT |
|  |  |  | Motif-4 | -184 | + | CTTGATTGGCTAGAA |
| P. infestans | PITG\_03244 | 966 | Motif-0 | -60 | - | GGCGAATCGGGAATGA |
|  |  |  | Motif-1 | -179 | + | CGTCCATGTACAGGAG |
|  |  |  | Motif-8 | -236 | + | TTAAATTAAATAAAT |
|  |  |  | Motif-9 | -383 | + | ATGTACCGGTA |
| P. infestans | PITG\_03245 | 966 | Motif-0 | -49 | - | GGAAATCTCGAAATGA |
|  |  |  | Motif-1 | -341 | + | AACATGTATTATACT |
|  |  |  | Motif-8 | -682 | - | TTTTTATATTTTAAA |
|  |  |  | Motif-9 | -594 | - | ATGTACCGGTA |
| P. infestans | PITG\_03246 | 345 | Motif-3 | -41 | + | TACAAACGCCAAGTTA |
| P. infestans | PITG\_03250 | 1000 | Motif-2 | -297 | - | ATAACTTCAAGTACAA |
|  |  |  | Motif-8 | -364 | - | TTTTTTTTAATTTTT |
| P. infestans | PITG\_03252 | 1000 | Motif-1 | -250 | - | TAGTTCTACACGTTT |
|  |  |  | Motif-16 | -840 | + | CAACAGCAACAGCAGC |
|  |  |  | Motif-18 | -75 | - | TGGTGTGGTGCACATA |
|  |  |  | Motif-3 | -812 | - | CACTTAGATATTGTAT |
|  |  |  | Motif-6 | -38 | - | TGATCCCCCCCCCCAT |
|  |  |  | Motif-8 | -299 | + | TTTTTTTAAATAGTT |
| P. infestans | PITG\_03253 | 1000 | Motif-0 | -146 | - | TTACTTTCCAAATTG |
|  |  |  | Motif-3 | -349 | + | TCTTGGGTTTTGTAG |
|  |  |  | Motif-7 | -574 | + | CAGTATTAATA |
|  |  |  | Motif-8 | -30 | + | TATATTTAAATAACT |
| P. infestans | PITG\_03255 | 1000 | Motif-0 | -680 | + | ACATTTCCAAATTTTA |
|  |  |  | Motif-4 | -157 | + | TCCCATTGGCTAAAA |
|  |  |  | Motif-8 | -740 | + | TTATTTTTAATAATA |
| P. infestans | PITG\_03256 | 1000 | Motif-0 | -65 | - | GGAAGTTTTAGAATGA |
|  |  |  | Motif-1 | -305 | + | TGCATGTAGTAAAAA |
| P. infestans | PITG\_03258 | 1000 | Motif-0 | -63 | - | GTAAATCGGAGAATGA |
|  |  |  | Motif-1 | -423 | - | TACAAGTACA |
| P. infestans | PITG\_03260 | 307 | Motif-1 | -140 | + | TACATGTAT |
|  |  |  | Motif-9 | -164 | - | CTGTACCGGTA |
| P. infestans | PITG\_03261 | 1000 | Motif-1 | -914 | + | TGCTACTACAAGTAG |
| P. infestans | PITG\_03262 | 251 | Motif-0 | -123 | + | GCATTCTCCAACTCTA |
|  |  |  | Motif-4 | -57 | - | ACCGATTGGCTGAAA |
| P. infestans | PITG\_03263 | 251 | Motif-0 | -144 | - | GCATTCTCCAACTCTA |
|  |  |  | Motif-4 | -209 | + | ACCGATTGGCTGAAA |
| P. infestans | PITG\_03264 | 208 | Motif-0 | -203 | + | CGAAAATGCAGAGTGA |
| P. infestans | PITG\_03265 | 206 | Motif-2 | -198 | - | AAGGTTGAAGTT |
|  |  |  | Motif-4 | -132 | - | TCTGATTTGCTGAAT |
| P. infestans | PITG\_03266 | 247 | Motif-16 | -99 | - | GAGCAGCCGCTGCAGC |
| P. infestans | PITG\_03267 | 1000 | Motif-0 | -34 | + | TCACTTCCGTTTTTTT |
|  |  |  | Motif-1 | -216 | + | TACATGTAC |
| P. infestans | PITG\_03268 | 936 | Motif-0 | -71 | - | TCACTCGCAGTTTCCC |
| P. infestans | PITG\_03269 | 941 | Motif-0 | -381 | - | GTTAGTTTCAAAATGA |
|  |  |  | Motif-2 | -20 | - | ATGGACGCTGAAGTGG |
|  |  |  | Motif-4 | -248 | + | CCTCATTGGCAGATA |
| P. infestans | PITG\_03270 | 941 | Motif-0 | -576 | + | GTTAGTTTCAAAATGA |
|  |  |  | Motif-2 | -937 | + | ATGGACGCTGAAGTGG |
|  |  |  | Motif-4 | -708 | - | CCTCATTGGCAGATA |
| P. infestans | PITG\_03271 | 1000 | Motif-2 | -652 | + | GGGCTTCAACTCCA |
| P. infestans | PITG\_03272 | 988 | Motif-0 | -762 | - | ACATTTTTCATTTTCA |
|  |  |  | Motif-1 | -947 | + | TACATGTAA |
|  |  |  | Motif-8 | -790 | + | TTTTTTTTTATTTAT |
| P. infestans | PITG\_03273 | 639 | Motif-2 | -16 | - | GCTGAAGCTGAAGTGT |
| P. infestans | PITG\_03274 | 160 | Motif-4 | -59 | - | GATGATTGGCTGAAC |
| P. infestans | PITG\_03275 | 160 | Motif-4 | -116 | + | GATGATTGGCTGAAC |
| P. infestans | PITG\_03276 | 542 | Motif-0 | -516 | + | GAGGATTGGAGAGTGA |
|  |  |  | Motif-3 | -156 | + | GACTTGCTTTTTGTGA |
| P. infestans | PITG\_03277 | 542 | Motif-0 | -42 | - | GAGGATTGGAGAGTGA |
|  |  |  | Motif-3 | -402 | - | GACTTGCTTTTTGTGA |
| P. infestans | PITG\_03278 | 1000 | Motif-0 | -132 | - | TCAAATGCGAAACTGA |
|  |  |  | Motif-2 | -561 | - | CACTTCAACTT |
| P. infestans | PITG\_03279 | 1000 | Motif-0 | -426 | + | CACTTCGTAATTTACG |
| P. infestans | PITG\_03280 | 1000 | Motif-2 | -96 | + | CAGTTTGAAGCTGA |
| P. infestans | PITG\_03281 | 1000 | Motif-1 | -605 | - | TACAAGTACA |
|  |  |  | Motif-18 | -19 | + | TAGTGTGGCTTACGAA |
| P. infestans | PITG\_03282 | 1000 | Motif-1 | -128 | + | TGTACAGGTAT |
| P. infestans | PITG\_03283 | 708 | Motif-8 | -481 | - | TAATTTTTAATATAA |
| P. infestans | PITG\_03284 | 708 | Motif-8 | -242 | + | TAATTTTTAATATAA |
| P. infestans | PITG\_03287 | 1000 | Motif-1 | -593 | + | TACATGAAC |
|  |  |  | Motif-17 | -108 | - | GGTGTAGCCATGTTG |
|  |  |  | Motif-4 | -73 | - | TTTGATTGGCTGATG |
| P. infestans | PITG\_03288 | 1000 | Motif-1 | -477 | + | AGTACATGAAG |
|  |  |  | Motif-17 | -977 | + | GGTGTAGCCATGTTG |
| P. infestans | PITG\_03290 | 455 | Motif-0 | -33 | + | CACTTTCGAATCTGCC |
|  |  |  | Motif-2 | -65 | + | CCACTTCAACTGTG |
|  |  |  | Motif-3 | -163 | - | TACGAAAGTCGAGTGA |
| P. infestans | PITG\_03291 | 455 | Motif-0 | -437 | + | GCAGATTCGAAAGTGA |
|  |  |  | Motif-2 | -360 | - | CACTTCAACTT |
|  |  |  | Motif-3 | -308 | + | TACGAAAGTCGAGTGA |
| P. infestans | PITG\_03292 | 871 | Motif-0 | -119 | - | GAGATTTGGAAAATGG |
|  |  |  | Motif-18 | -168 | - | TGGTGCGGCGTACAAG |
| P. infestans | PITG\_03293 | 185 | Motif-0 | -135 | + | TCACTACTGTTTTGAC |
| P. infestans | PITG\_03294 | 441 | Motif-2 | -192 | + | GCTTCAACACG |
| P. infestans | PITG\_03295 | 441 | Motif-2 | -260 | - | GCTTCAACACG |
| P. infestans | PITG\_03296 | 552 | Motif-0 | -148 | + | GCAAGTGGAAAAATGG |
|  |  |  | Motif-2 | -380 | - | TATGTTGAAGTG |
|  |  |  | Motif-6 | -226 | - | AGTTCCCCCCCCCCCT |
| P. infestans | PITG\_03297 | 552 | Motif-0 | -419 | + | CATTTTTCCACTTGCT |
|  |  |  | Motif-2 | -183 | - | CACTTCAACAT |
|  |  |  | Motif-6 | -342 | + | AGTTCCCCCCCCCCCT |
| P. infestans | PITG\_03298 | 1000 | Motif-4 | -70 | + | TTTGATTGGCTGAAA |
| P. infestans | PITG\_03300 | 259 | Motif-3 | -148 | + | ACTTGAGCTTTGTAT |
|  |  |  | Motif-8 | -44 | - | TTTTTCTAAATTTAA |
| P. infestans | PITG\_03301 | 259 | Motif-3 | -126 | - | TACTTGAGCTTTGTAT |
|  |  |  | Motif-8 | -230 | + | TTTTTCTAAATTTAA |
| P. infestans | PITG\_03303 | 195 | Motif-1 | -128 | - | CCAAATTACCTGTAC |
|  |  |  | Motif-16 | -17 | + | GAACAGCAGCACTGAC |
| P. infestans | PITG\_03304 | 195 | Motif-1 | -76 | + | TACCTGTACCATTTT |
|  |  |  | Motif-16 | -194 | - | GAACAGCAGCACTGAC |
| P. infestans | PITG\_03305 | 204 | Motif-2 | -67 | + | CCACTTCAAGTTTC |
| P. infestans | PITG\_03306 | 1000 | Motif-0 | -49 | + | CATTTCCGATTTCGCT |
|  |  |  | Motif-16 | -887 | + | CAGAAGCAGCGCCAGC |
|  |  |  | Motif-2 | -582 | + | ACTGCAACACG |
|  |  |  | Motif-3 | -491 | - | AACCTGAGTTTTGTAT |
| P. infestans | PITG\_03307 | 1000 | Motif-16 | -408 | - | CAGAAGCAGCGCCAGC |
|  |  |  | Motif-2 | -708 | - | ACTGCAACACG |
|  |  |  | Motif-3 | -803 | - | AATACAAAACTCAGGT |
| P. infestans | PITG\_03308 | 285 | Motif-2 | -232 | + | GCGCTTCAAGATCG |
| P. infestans | PITG\_03309 | 285 | Motif-2 | -67 | - | GCGCTTCAAGATCG |
| P. infestans | PITG\_03310 | 276 | Motif-0 | -216 | + | TCATTTTTAAAGTGAC |
| P. infestans | PITG\_03312 | 321 | Motif-1 | -234 | + | TACATGTAG |
| P. infestans | PITG\_03313 | 176 | Motif-2 | -146 | - | AATATTGAAGTGGA |
|  |  |  | Motif-7 | -136 | + | TATTATTAATA |
| P. infestans | PITG\_03318 | 1000 | Motif-1 | -112 | + | TGCATGTATCGTCAA |
| P. infestans | PITG\_03319 | 1000 | Motif-0 | -257 | - | TCACTCTCATTTCGGA |
| P. infestans | PITG\_03320 | 1000 | Motif-2 | -699 | - | ACTACTTCAATTTCAA |
| P. infestans | PITG\_03322 | 1000 | Motif-0 | -105 | - | TCATTCTTACAATTTC |
|  |  |  | Motif-18 | -50 | - | TTGTGTGGCGCACACT |
|  |  |  | Motif-9 | -281 | + | ACGTACCGGTA |
| P. infestans | PITG\_03331 | 722 | Motif-4 | -664 | + | GGTGATTGGCCAAAA |
|  |  |  | Motif-6 | -72 | - | CGACCCACCCACCCAC |
| P. infestans | PITG\_03332 | 722 | Motif-4 | -73 | - | GGTGATTGGCCAAAA |
|  |  |  | Motif-6 | -666 | + | CGACCCACCCACCCAC |
| P. infestans | PITG\_03333 | 1000 | Motif-0 | -376 | + | GCACTTCCCAACTTT |
|  |  |  | Motif-1 | -226 | + | TACATGTAA |
|  |  |  | Motif-4 | -496 | + | TATAAGCCAATCTGC |
|  |  |  | Motif-8 | -326 | + | TATTTCTAATTTAAA |
| P. infestans | PITG\_03334 | 1000 | Motif-1 | -922 | + | TACATGCAC |
| P. infestans | PITG\_03335 | 1000 | Motif-0 | -62 | + | CATTCCGCATTTTGCT |
|  |  |  | Motif-18 | -948 | - | TGGTGTAGTGCACACA |
|  |  |  | Motif-2 | -928 | - | GCATCAACCTTGACAA |
|  |  |  | Motif-9 | -970 | - | CCGTACCGGTA |
| P. infestans | PITG\_03336 | 1000 | Motif-18 | -213 | + | TGGTGTAGTGCACACA |
|  |  |  | Motif-2 | -130 | - | AAAATTGAAGTGGA |
|  |  |  | Motif-9 | -186 | + | CCGTACCGGTA |
| P. infestans | PITG\_03337 | 278 | Motif-2 | -127 | - | CACTTCAACTT |
| P. infestans | PITG\_03338 | 206 | Motif-2 | -34 | + | ACTTCAACTTC |
|  |  |  | Motif-4 | -83 | - | GACGATTGGTCAATA |
| P. infestans | PITG\_03339 | 206 | Motif-2 | -182 | - | CACTTCAACTT |
|  |  |  | Motif-4 | -106 | - | TACTAGCGAATCAAA |
| P. infestans | PITG\_03340 | 1000 | Motif-0 | -87 | + | CATTCCGGAATTTGCC |
|  |  |  | Motif-1 | -310 | + | TGCATGTAGTCCTCA |
|  |  |  | Motif-4 | -716 | + | TCTCATTGGTCGCAT |
|  |  |  | Motif-6 | -35 | + | CGCACTCCCCACCCCT |
| P. infestans | PITG\_03344 | 351 | Motif-0 | -218 | - | TCATTGTGATATCTGC |
| P. infestans | PITG\_03345 | 351 | Motif-0 | -149 | + | TCATTGTGATATCTGC |
| P. infestans | PITG\_03346 | 1000 | Motif-1 | -444 | + | TACATGTAC |
|  |  |  | Motif-3 | -247 | + | TACAAAAGTCAAGTCT |
|  |  |  | Motif-4 | -172 | + | GATGATTGGCTAGAA |
| P. infestans | PITG\_03347 | 561 | Motif-0 | -70 | + | CATTTCGGAATTGGCG |
|  |  |  | Motif-4 | -231 | + | TTTGATTGGTCTAAA |
| P. infestans | PITG\_03350 | 69 | Motif-1 | -47 | + | TACATGTAC |
|  |  |  | Motif-17 | -68 | + | AGTGTAGCCATCTGA |
| P. infestans | PITG\_03351 | 421 | Motif-4 | -277 | - | TACGATTGGTTTAAA |
| P. infestans | PITG\_03352 | 421 | Motif-4 | -159 | + | TACGATTGGTTTAAA |
| P. infestans | PITG\_03353 | 355 | Motif-4 | -123 | + | CCTGATTGGCTAAAA |
| P. infestans | PITG\_03354 | 355 | Motif-4 | -247 | - | CCTGATTGGCTAAAA |
| P. infestans | PITG\_03355 | 632 | Motif-0 | -221 | + | CACTTTTGGATTTGCT |
|  |  |  | Motif-1 | -573 | - | TACATGTGGACATAA |
| P. infestans | PITG\_03356 | 1000 | Motif-1 | -115 | + | TACATGTAC |
|  |  |  | Motif-4 | -72 | - | TTTGATTGGCTGATC |
| P. infestans | PITG\_03357 | 454 | Motif-1 | -205 | - | TACATGTGCA |
| P. infestans | PITG\_03358 | 417 | Motif-0 | -40 | + | CACTTTTGCTTTCACC |
| P. infestans | PITG\_03359 | 332 | Motif-0 | -126 | - | GGAGATTCGAAAGTAA |
|  |  |  | Motif-3 | -58 | - | AATACAAAAAGCAATT |
| P. infestans | PITG\_03360 | 332 | Motif-0 | -112 | + | CATTTTACAATTTGTT |
|  |  |  | Motif-3 | -289 | - | AAATTGCTTTTTGTAT |
| P. infestans | PITG\_03361 | 686 | Motif-0 | -291 | + | GCCAGTTCAAAAGTGG |
|  |  |  | Motif-1 | -129 | + | TACATGTAC |
|  |  |  | Motif-4 | -583 | + | TGTGATGGGATAATA |
|  |  |  | Motif-8 | -350 | - | TATTGTTTATTAAAA |
| P. infestans | PITG\_03362 | 1000 | Motif-0 | -127 | + | CACTTGTGAACTTACT |
|  |  |  | Motif-1 | -628 | + | TACATGTAT |
| P. infestans | PITG\_03363 | 173 | Motif-18 | -111 | - | TTGTGTGCTGCACAAA |
| P. infestans | PITG\_03364 | 173 | Motif-18 | -78 | + | TTGTGTGCTGCACAAA |
| P. infestans | PITG\_03365 | 457 | Motif-0 | -374 | + | GCCAATCGGAAAATGC |
|  |  |  | Motif-4 | -379 | - | TCCGATTGGCAGAAA |
|  |  |  | Motif-8 | -211 | - | TACTACTAATTTAAA |
| P. infestans | PITG\_03368 | 279 | Motif-1 | -93 | - | AATACATGGACATGTA |
| P. infestans | PITG\_03370 | 921 | Motif-8 | -131 | - | TTTTTTTAAATAGTA |
| P. infestans | PITG\_03372 | 1000 | Motif-3 | -360 | + | CACACAAAACCCAGGT |
| P. infestans | PITG\_03373 | 254 | Motif-1 | -241 | + | AACATGTATTAAATC |
| P. infestans | PITG\_03374 | 270 | Motif-0 | -61 | - | GCAAGTCCGAGAATGA |
|  |  |  | Motif-9 | -18 | - | CTGTACCGGTA |
| P. infestans | PITG\_03375 | 270 | Motif-0 | -225 | + | GCAAGTCCGAGAATGA |
|  |  |  | Motif-9 | -263 | + | CTGTACCGGTA |
| P. infestans | PITG\_03376 | 1000 | Motif-4 | -235 | - | CATTATTGGACAAAA |
| P. infestans | PITG\_03379 | 1000 | Motif-0 | -59 | + | TCACTGTTCCTTTGTC |
| P. infestans | PITG\_03380 | 377 | Motif-1 | -103 | + | TACGTGTAC |
|  |  |  | Motif-9 | -226 | - | CAGTACCGGTA |
| P. infestans | PITG\_03381 | 1000 | Motif-0 | -221 | + | CACTTTGCCATTTGCG |
|  |  |  | Motif-1 | -760 | - | GCTACAGGTAAAATA |
| P. infestans | PITG\_03382 | 1000 | Motif-1 | -180 | + | TACATGTAG |
|  |  |  | Motif-8 | -506 | + | TTTTAATAATTTATT |
| P. infestans | PITG\_03383 | 1000 | Motif-0 | -86 | + | CATTCTCCATTTTACT |
|  |  |  | Motif-1 | -862 | + | TACATGTAC |
|  |  |  | Motif-6 | -800 | - | AGCCCCCCCCCCCCAA |
|  |  |  | Motif-8 | -366 | - | TACTTTTAAGTAATT |
| P. infestans | PITG\_03384 | 285 | Motif-18 | -250 | - | TTGTGTGGTGTACACC |
| P. infestans | PITG\_03385 | 285 | Motif-18 | -51 | + | TTGTGTGGTGTACACC |
| P. infestans | PITG\_03386 | 336 | Motif-4 | -137 | - | TCTGATTGGTGGATA |
|  |  |  | Motif-9 | -313 | + | CTTTACCGGTA |
| P. infestans | PITG\_03387 | 336 | Motif-4 | -214 | + | TCTGATTGGTGGATA |
|  |  |  | Motif-9 | -34 | - | CTTTACCGGTA |
| P. infestans | PITG\_03389 | 1000 | Motif-2 | -118 | - | TAAGTTGAAGTT |
|  |  |  | Motif-9 | -86 | + | AAGTACCGGTA |
| P. infestans | PITG\_03390 | 637 | Motif-2 | -90 | + | CAGCTTCACCCTC |
| P. infestans | PITG\_03391 | 637 | Motif-2 | -66 | + | TCGCTTCAAGCTGC |
| P. infestans | PITG\_03392 | 1000 | Motif-7 | -935 | - | TATTATTAATA |
| P. infestans | PITG\_03393 | 1000 | Motif-2 | -256 | + | GTGGTTGACGTG |
|  |  |  | Motif-7 | -677 | + | TATTATTAATA |
| P. infestans | PITG\_03394 | 300 | Motif-0 | -112 | + | TCATTTCTGAAGCTTA |
| P. infestans | PITG\_03395 | 1000 | Motif-0 | -54 | - | GCAGATTGCGAAGTAA |
|  |  |  | Motif-1 | -648 | + | TACATGTAG |
|  |  |  | Motif-17 | -716 | - | ACTGTACCCATGCCA |
| P. infestans | PITG\_03396 | 1000 | Motif-1 | -476 | + | TACATGTAG |
|  |  |  | Motif-17 | -415 | + | ACTGTACCCATGCCA |
| P. infestans | PITG\_03397 | 144 | Motif-1 | -95 | - | TACAAGTACT |
|  |  |  | Motif-4 | -59 | - | TTTGATTCGCTAAAA |
| P. infestans | PITG\_03398 | 144 | Motif-1 | -59 | + | TACAAGTACT |
|  |  |  | Motif-4 | -100 | + | TTTGATTCGCTAAAA |
| P. infestans | PITG\_03400 | 262 | Motif-2 | -188 | + | ACTTCAACTTG |
| P. infestans | PITG\_03401 | 1000 | Motif-1 | -75 | + | GGTAAATGTACTGAT |
|  |  |  | Motif-18 | -974 | - | TGATGTGGCTTACATA |
| P. infestans | PITG\_03402 | 893 | Motif-1 | -703 | - | TACATGTGTA |
| P. infestans | PITG\_03404 | 245 | Motif-4 | -77 | + | TTGGATTGGTTGAAA |
| P. infestans | PITG\_03405 | 245 | Motif-4 | -183 | - | TTGGATTGGTTGAAA |
| P. infestans | PITG\_03407 | 1000 | Motif-2 | -50 | - | CATTTTGAAGTG |
| P. infestans | PITG\_03408 | 1000 | Motif-2 | -904 | - | CCTTCAACCTTAGCCA |
|  |  |  | Motif-4 | -175 | - | GTCAATTGGACAAAA |
| P. infestans | PITG\_03409 | 617 | Motif-3 | -315 | + | ACTGGACAATTGTAT |
| P. infestans | PITG\_03410 | 315 | Motif-16 | -150 | + | GCATAGCAGCCCTAGC |
| P. infestans | PITG\_03411 | 315 | Motif-16 | -181 | - | GCATAGCAGCCCTAGC |
| P. infestans | PITG\_03412 | 1000 | Motif-1 | -757 | - | TCTGTACGTGTAGA |
|  |  |  | Motif-2 | -657 | + | TGCCACGCTGAAGCGT |
|  |  |  | Motif-7 | -407 | + | AATTATTAATA |
| P. infestans | PITG\_03414 | 598 | Motif-0 | -53 | - | GCAGATGTGGGAGTGA |
|  |  |  | Motif-1 | -318 | + | TACATGTAC |
|  |  |  | Motif-3 | -112 | + | TCTTAAAGTTTGTAT |
| P. infestans | PITG\_03415 | 465 | Motif-0 | -238 | + | GTAATTTCAAGAATGG |
| P. infestans | PITG\_03416 | 1000 | Motif-0 | -938 | - | TCATTCTCCAATTCG |
|  |  |  | Motif-1 | -227 | + | TGCATGTAC |
| P. infestans | PITG\_03417 | 1000 | Motif-0 | -96 | + | CATTCTCCAATTCGCT |
|  |  |  | Motif-1 | -298 | - | TACATGAAC |
| P. infestans | PITG\_03418 | 910 | Motif-1 | -676 | + | TACATGTAC |
|  |  |  | Motif-4 | -313 | - | TATGATTGGTTAAAA |
| P. infestans | PITG\_03419 | 910 | Motif-1 | -242 | + | TACATGTAT |
|  |  |  | Motif-4 | -612 | + | TATGATTGGTTAAAA |
| P. infestans | PITG\_03422 | 1000 | Motif-0 | -153 | + | TCACTCTCAAAGTTAA |
| P. infestans | PITG\_03425 | 1000 | Motif-1 | -184 | - | TACCTGTATACTTTT |
| P. infestans | PITG\_03427 | 232 | Motif-0 | -54 | - | TCAATTTCGAAAATGA |
| P. infestans | PITG\_03428 | 1000 | Motif-0 | -392 | - | TCATTCTCCAAAACTC |
|  |  |  | Motif-18 | -51 | - | TGATGTGGTGTACACA |
| P. infestans | PITG\_03429 | 1000 | Motif-0 | -324 | - | TTATTTTTAAACTTT |
|  |  |  | Motif-8 | -325 | - | TATTTTTAAACTTTT |
| P. infestans | PITG\_03431 | 1000 | Motif-7 | -606 | - | CATTATTAATA |
| P. infestans | PITG\_03432 | 1000 | Motif-1 | -440 | + | TACATGTAT |
|  |  |  | Motif-4 | -190 | + | TATGATTGGTCAAAA |
| P. infestans | PITG\_03434 | 1000 | Motif-4 | -181 | + | TAGAAACCAATCACA |
| P. infestans | PITG\_03435 | 1000 | Motif-0 | -344 | - | TTACTCTTCAAATTG |
|  |  |  | Motif-17 | -352 | + | AATGTAGCCAATTTG |
| P. infestans | PITG\_03436 | 1000 | Motif-2 | -517 | + | CAAGTTGCAGTC |
| P. infestans | PITG\_03437 | 1000 | Motif-1 | -587 | + | TACATGTAC |
|  |  |  | Motif-2 | -192 | + | CACATTGAAGCGGA |
|  |  |  | Motif-3 | -426 | + | ACTTGAATTTAGTAA |
|  |  |  | Motif-8 | -162 | - | TATTATTAAACAAGA |
| P. infestans | PITG\_03438 | 1000 | Motif-2 | -239 | - | ATTTAAGCTGAAGTGG |
| P. infestans | PITG\_03440 | 1000 | Motif-0 | -66 | + | CATTCCGCAATTTGCC |
|  |  |  | Motif-1 | -631 | + | TACATGTAA |
|  |  |  | Motif-3 | -226 | - | TACAAAAAGCAGGTGA |
|  |  |  | Motif-4 | -167 | + | GCCTATTGGCTAAAA |
| P. infestans | PITG\_03441 | 1000 | Motif-0 | -66 | + | CATTCCGCAATTTGCC |
|  |  |  | Motif-1 | -939 | + | TACATGTGCA |
|  |  |  | Motif-3 | -236 | - | TACAAAAAGCAGGTGA |
|  |  |  | Motif-4 | -167 | + | GCCTATTGGCTAAAA |
| P. infestans | PITG\_03443 | 1000 | Motif-1 | -269 | + | AATACGTGTATTTCT |
|  |  |  | Motif-2 | -240 | - | ATTTAAGCTGAAGTGG |
|  |  |  | Motif-7 | -276 | + | AAATATTAATA |
| P. infestans | PITG\_03444 | 348 | Motif-0 | -291 | + | TCACTTACCATTGTGC |
|  |  |  | Motif-1 | -242 | + | TACATGTAG |
| P. infestans | PITG\_03447 | 1000 | Motif-0 | -97 | + | GCATTAGTCAATTTAC |
|  |  |  | Motif-1 | -19 | + | TACAGGTGCAGGTACC |
| P. infestans | PITG\_03448 | 1000 | Motif-0 | -64 | + | GCAGTTTGCGATTCGC |
|  |  |  | Motif-1 | -979 | + | TACATGTAA |
| P. infestans | PITG\_03449 | 704 | Motif-1 | -614 | + | TACATGTAA |
| P. infestans | PITG\_03450 | 347 | Motif-1 | -263 | + | TACATGTAC |
| P. infestans | PITG\_03451 | 1000 | Motif-1 | -430 | + | TACATGTAC |
|  |  |  | Motif-3 | -199 | - | AACTTGGATTTCGTAT |
| P. infestans | PITG\_03454 | 593 | Motif-0 | -46 | - | GCAAGTCGGAAAATGG |
| P. infestans | PITG\_03455 | 593 | Motif-0 | -556 | + | GGAAAATGGCAAGTGC |
| P. infestans | PITG\_03456 | 1000 | Motif-0 | -297 | + | CACTTCCTCTTTTACC |
|  |  |  | Motif-1 | -33 | - | ACGACATGTAC |
| P. infestans | PITG\_03457 | 1000 | Motif-18 | -599 | + | TCGTGTAGTGCACGCG |
| P. infestans | PITG\_03458 | 300 | Motif-0 | -36 | + | CACTTCGGAACTTACC |
|  |  |  | Motif-6 | -78 | + | CCAACTCCCCCCTTCA |
| P. infestans | PITG\_03459 | 300 | Motif-0 | -279 | - | GCACTTCGGAACTTAC |
|  |  |  | Motif-6 | -238 | - | CCAACTCCCCCCTTCA |
| P. infestans | PITG\_03460 | 196 | Motif-0 | -42 | + | TCATACGTGGACTTGC |
| P. infestans | PITG\_03462 | 867 | Motif-0 | -36 | - | GCAATTGCGAGAGTGA |
|  |  |  | Motif-1 | -205 | + | TACATGTAC |
|  |  |  | Motif-2 | -101 | + | ACTTCAACTTG |
|  |  |  | Motif-6 | -866 | - | TCCCCTCCACCCCCAC |
| P. infestans | PITG\_03463 | 867 | Motif-0 | -847 | + | GCAATTGCGAGAGTGA |
|  |  |  | Motif-1 | -300 | + | TACATGTAC |
|  |  |  | Motif-2 | -776 | - | CACTTCAACTT |
|  |  |  | Motif-6 | -17 | + | TCCCCTCCACCCCCAC |
| P. infestans | PITG\_03464 | 468 | Motif-0 | -154 | + | CATTCTCCAATTTGCA |
|  |  |  | Motif-1 | -169 | + | TACATGTAT |
| P. infestans | PITG\_03465 | 468 | Motif-0 | -328 | - | TCATTCTCCAATTTG |
|  |  |  | Motif-1 | -307 | + | TACATGTAT |
| P. infestans | PITG\_03466 | 627 | Motif-0 | -462 | + | CACTTTGCCATTCACC |
|  |  |  | Motif-4 | -531 | - | TTTGATTCGCTGATA |
| P. infestans | PITG\_03467 | 627 | Motif-0 | -180 | + | GTGAATGGCAAAGTGA |
|  |  |  | Motif-4 | -111 | + | TTTGATTCGCTGATA |
| P. infestans | PITG\_03468 | 297 | Motif-2 | -244 | - | CACTTCAACAT |
|  |  |  | Motif-3 | -174 | + | TACAAAACGTCAATCA |
| P. infestans | PITG\_03469 | 1000 | Motif-2 | -996 | - | GCTGCAACTTG |
|  |  |  | Motif-8 | -533 | - | TAAGTTTTATTATAT |
| P. infestans | PITG\_03470 | 1000 | Motif-4 | -684 | - | TTTGATTGGCTGAAA |
| P. infestans | PITG\_03471 | 1000 | Motif-0 | -310 | - | AAAAATTGTAAAGTGA |
|  |  |  | Motif-1 | -481 | + | TACATGTAT |
|  |  |  | Motif-3 | -262 | + | TACAAAATCTCAGTCA |
|  |  |  | Motif-4 | -959 | + | TTTGATTGGATAAAC |
| P. infestans | PITG\_03472 | 1000 | Motif-1 | -795 | + | TACATGTAC |
|  |  |  | Motif-3 | -963 | - | CACTTGATCGTCGTAT |
| P. infestans | PITG\_03473 | 950 | Motif-0 | -825 | - | CCACTTTCCTTTTCTA |
|  |  |  | Motif-2 | -471 | - | GTGCAAGATGAAGCTA |
|  |  |  | Motif-3 | -321 | + | ACTTGGCTTCAGTAT |
| P. infestans | PITG\_03474 | 950 | Motif-0 | -141 | + | CCACTTTCCTTTTCTA |
|  |  |  | Motif-2 | -495 | + | GTGCAAGATGAAGCTA |
|  |  |  | Motif-3 | -643 | + | TACTGAAGCCAAGTGA |
| P. infestans | PITG\_03475 | 341 | Motif-0 | -328 | - | GCATTGTCCAATCTTC |
|  |  |  | Motif-2 | -333 | - | ATCTTCAACTT |
|  |  |  | Motif-4 | -254 | - | TACCATTGGCTAATA |
| P. infestans | PITG\_03476 | 341 | Motif-0 | -29 | + | GCATTGTCCAATCTTC |
|  |  |  | Motif-2 | -18 | + | TCTTCAACTTCGATAA |
|  |  |  | Motif-4 | -102 | + | TACCATTGGCTAATA |
| P. infestans | PITG\_03477 | 451 | Motif-0 | -206 | + | TCACATGTCAATCTGC |
|  |  |  | Motif-1 | -296 | - | GCTACATGAAG |
|  |  |  | Motif-2 | -304 | + | AACTTCGACTT |
|  |  |  | Motif-4 | -106 | + | TCTCATTGGCTGAAA |
| P. infestans | PITG\_03478 | 454 | Motif-1 | -311 | + | TACATGCAC |
|  |  |  | Motif-3 | -143 | + | ACTTGATGTTTGTAG |
| P. infestans | PITG\_03479 | 1000 | Motif-1 | -94 | + | ACTACAGGTAC |
| P. infestans | PITG\_03480 | 127 | Motif-0 | -126 | + | GTTAATTCGGAAGTGA |
| P. infestans | PITG\_03481 | 127 | Motif-0 | -17 | - | GTTAATTCGGAAGTGA |
| P. infestans | PITG\_03482 | 321 | Motif-0 | -62 | - | TCACTCGCCATCTTCA |
| P. infestans | PITG\_03483 | 246 | Motif-2 | -242 | - | GGTCTAGCTGAAGTTG |
| P. infestans | PITG\_03486 | 413 | Motif-4 | -132 | + | TCTGACTGGCTGATA |
| P. infestans | PITG\_03489 | 817 | Motif-3 | -191 | + | ACTTGACTTTTGTAA |
| P. infestans | PITG\_03490 | 171 | Motif-17 | -159 | - | AATGTACCCATCTGT |
|  |  |  | Motif-4 | -118 | + | TCTTATTGGTTGATA |
| P. infestans | PITG\_03491 | 270 | Motif-0 | -221 | + | GTGAATTGAAAACTGA |
| P. infestans | PITG\_03492 | 270 | Motif-0 | -65 | - | GTGAATTGAAAACTGA |
| P. infestans | PITG\_03493 | 431 | Motif-1 | -261 | + | ATTACGTGTATCGTG |
|  |  |  | Motif-2 | -219 | - | GTGGTTGAAGTG |
|  |  |  | Motif-3 | -120 | - | CTCTTGCGCTTTGTAG |
|  |  |  | Motif-4 | -174 | + | TTGGATTGGACGATT |
| P. infestans | PITG\_03496 | 212 | Motif-4 | -204 | - | GCCAATTGGACGAAA |
| P. infestans | PITG\_03497 | 1000 | Motif-16 | -654 | - | CAGCAGCAGCAGTAAG |
|  |  |  | Motif-18 | -379 | - | TAGTGTGTTGCACACA |
| P. infestans | PITG\_03501 | 1000 | Motif-0 | -44 | - | GAGAAATGAAAAGTGA |
|  |  |  | Motif-1 | -223 | + | TACATGTAC |
|  |  |  | Motif-4 | -868 | + | AATGATTGGAAAAAA |
|  |  |  | Motif-8 | -521 | - | TAATATTTTATTAAA |
| P. infestans | PITG\_03502 | 512 | Motif-0 | -52 | - | GCGACACGAAAAATGA |
|  |  |  | Motif-4 | -207 | + | ACTGATTGGACAAAA |
| P. infestans | PITG\_03503 | 512 | Motif-0 | -476 | + | GCGACACGAAAAATGA |
|  |  |  | Motif-4 | -320 | - | ACTGATTGGACAAAA |
| P. infestans | PITG\_03507 | 1000 | Motif-4 | -284 | + | TCTGATTGGCTTTTA |
| P. infestans | PITG\_03508 | 1000 | Motif-0 | -98 | + | TCATTTTCACGCTTGC |
|  |  |  | Motif-7 | -980 | + | CACTATTAATA |
| P. infestans | PITG\_03511 | 1000 | Motif-1 | -209 | + | TACATGTAC |
| P. infestans | PITG\_03512 | 1000 | Motif-0 | -102 | + | GTGAGTTCAGGACTGA |
|  |  |  | Motif-1 | -648 | + | AGTACCGGTACTGTA |
|  |  |  | Motif-9 | -646 | - | CAGTACCGGTA |
| P. infestans | PITG\_03513 | 960 | Motif-0 | -243 | + | TCACTCGCCACTTTGA |
|  |  |  | Motif-1 | -469 | + | TACATGTAC |
|  |  |  | Motif-2 | -874 | + | GTTGTTGAAGTT |
| P. infestans | PITG\_03514 | 199 | Motif-4 | -67 | + | TTTCATTGGTCAAAG |
| P. infestans | PITG\_03515 | 199 | Motif-4 | -147 | - | TTTCATTGGTCAAAG |
| P. infestans | PITG\_03516 | 834 | Motif-0 | -140 | + | TCATTTCTCAAACGAC |
| P. infestans | PITG\_03517 | 1000 | Motif-0 | -196 | - | TAATTTCCCCATCTGC |
|  |  |  | Motif-1 | -236 | + | TACATGTAT |
| P. infestans | PITG\_03518 | 1000 | Motif-1 | -50 | - | TACATGTTTATATTC |
| P. infestans | PITG\_03519 | 260 | Motif-0 | -137 | + | GTCAATTCAAAATTGA |
|  |  |  | Motif-2 | -238 | - | ATCACTTCAAGGTATA |
| P. infestans | PITG\_03520 | 520 | Motif-2 | -429 | + | GCTCCAACTTG |
| P. infestans | PITG\_03521 | 520 | Motif-2 | -101 | - | AGCTCCAACTT |
| P. infestans | PITG\_03529 | 466 | Motif-0 | -430 | + | GCAGATTTGGAAGTGC |
|  |  |  | Motif-2 | -242 | - | AACTTCAACCT |
| P. infestans | PITG\_03530 | 466 | Motif-0 | -52 | - | GCAGATTTGGAAGTGC |
|  |  |  | Motif-2 | -235 | - | TAGGTTGAAGTT |
| P. infestans | PITG\_03531 | 547 | Motif-0 | -414 | - | TCACTTTGACTTCTTC |
| P. infestans | PITG\_03535 | 1000 | Motif-0 | -94 | + | CCATTCCCACTTTTAC |
|  |  |  | Motif-2 | -274 | + | GGCTTCAACTT |
|  |  |  | Motif-9 | -204 | + | ACGTACCGGTA |
| P. infestans | PITG\_03538 | 1000 | Motif-1 | -221 | + | GCTACAAGTACCGGTA |
|  |  |  | Motif-9 | -96 | + | CTGTACCGGTA |
| P. infestans | PITG\_03540 | 1000 | Motif-0 | -73 | + | CAGTTTGGAATCTGCC |
|  |  |  | Motif-1 | -372 | + | TACATGTAT |
|  |  |  | Motif-2 | -347 | + | ACATCAACGTGACAAA |
|  |  |  | Motif-7 | -411 | + | AAGTATTAATA |
| P. infestans | PITG\_03541 | 634 | Motif-1 | -316 | + | TACATGTAC |
|  |  |  | Motif-4 | -591 | - | TATTATTGGACGATA |
|  |  |  | Motif-7 | -411 | - | TACTATTAATA |
|  |  |  | Motif-8 | -614 | - | TACTTATTATTTAAA |
|  |  |  | Motif-9 | -402 | - | CAGTACCGGTA |
| P. infestans | PITG\_03542 | 1000 | Motif-0 | -67 | - | GCCGATTCCAAACTGA |
|  |  |  | Motif-1 | -337 | + | TACATGTAT |
|  |  |  | Motif-6 | -880 | + | CCCCCCCCCCCGACAA |
| P. infestans | PITG\_03543 | 532 | Motif-0 | -63 | - | GCTGATTGAAAAATGA |
|  |  |  | Motif-1 | -354 | + | TACGTGTAC |
|  |  |  | Motif-2 | -183 | - | AACTTCAACTT |
| P. infestans | PITG\_03544 | 590 | Motif-0 | -53 | - | TCATTTCGACATCTGA |
|  |  |  | Motif-1 | -384 | + | TACATGTAG |
|  |  |  | Motif-2 | -460 | - | CATATTGAAGTT |
|  |  |  | Motif-4 | -75 | - | GATGATTGGATAAAA |
| P. infestans | PITG\_03545 | 495 | Motif-4 | -375 | + | CTCTATTGGCTGAAA |
| P. infestans | PITG\_03546 | 792 | Motif-4 | -156 | - | CTTGATTGGTAGAAA |
| P. infestans | PITG\_03548 | 1000 | Motif-0 | -64 | - | TCAGAAATCGATTTGC |
|  |  |  | Motif-2 | -610 | - | CAGCTTCAACGAG |
| P. infestans | PITG\_03549 | 371 | Motif-0 | -326 | + | TCAAATCTCGGAATGA |
|  |  |  | Motif-4 | -198 | + | GCTGATTGGCGGAAT |
| P. infestans | PITG\_03550 | 371 | Motif-0 | -61 | - | TCAAATCTCGGAATGA |
|  |  |  | Motif-4 | -188 | - | GCTGATTGGCGGAAT |
| P. infestans | PITG\_03551 | 363 | Motif-0 | -50 | - | GAGAATTTGAGAATGA |
|  |  |  | Motif-9 | -249 | - | CGGTACCGGTA |
| P. infestans | PITG\_03552 | 363 | Motif-0 | -37 | + | CCACTCCTCTTTTCTC |
|  |  |  | Motif-9 | -125 | + | CGGTACCGGTA |
| P. infestans | PITG\_03553 | 948 | Motif-0 | -904 | + | GCCGAATTGGAAGTGA |
|  |  |  | Motif-1 | -37 | + | TCTCTACAAGTATT |
|  |  |  | Motif-8 | -118 | - | TTTTTTTAATTTGCA |
| P. infestans | PITG\_03554 | 948 | Motif-0 | -60 | - | GCCGAATTGGAAGTGA |
|  |  |  | Motif-1 | -925 | - | TCTCTACAAGTATT |
|  |  |  | Motif-8 | -845 | + | TTTTTTTAATTTGCA |
| P. infestans | PITG\_03555 | 1000 | Motif-0 | -58 | + | TCATTCCCGTAACCGC |
| P. infestans | PITG\_03559 | 1000 | Motif-0 | -839 | - | GCAAAAAGAGAAGTGA |
|  |  |  | Motif-1 | -306 | + | TACATGCATT |
|  |  |  | Motif-2 | -266 | + | ACTTCAACTTG |
|  |  |  | Motif-3 | -928 | - | GACTTGCTACATGTAT |
| P. infestans | PITG\_03560 | 284 | Motif-4 | -219 | - | CTTGATTGGACGAAT |
| P. infestans | PITG\_03561 | 284 | Motif-4 | -80 | + | CTTGATTGGACGAAT |
| P. infestans | PITG\_03564 | 100 | Motif-0 | -57 | + | CCATTTCCGATTCCCC |
| P. infestans | PITG\_03566 | 1000 | Motif-4 | -796 | - | TTCGACTGGAAAAAA |
|  |  |  | Motif-6 | -70 | - | CGACCCACCCACCCAC |
| P. infestans | PITG\_03568 | 1000 | Motif-4 | -192 | + | TTGCAGCCAATTATA |
|  |  |  | Motif-8 | -654 | - | TATTTATAAGTAAGA |
| P. infestans | PITG\_03569 | 945 | Motif-16 | -175 | - | CACCAGCCGCAGTAGC |
|  |  |  | Motif-8 | -897 | - | TATTTTTTTTTAGAA |
| P. infestans | PITG\_03570 | 945 | Motif-16 | -786 | + | CACCAGCCGCAGTAGC |
|  |  |  | Motif-8 | -63 | + | TATTTTTTTTTAGAA |
| P. infestans | PITG\_03571 | 238 | Motif-1 | -210 | + | TACATGTAG |
|  |  |  | Motif-2 | -238 | - | TCAGCTTCAAGATGAC |
|  |  |  | Motif-4 | -90 | - | TCTCATTGGCTGATG |
| P. infestans | PITG\_03573 | 430 | Motif-6 | -283 | - | GCCCCCTCCCACTCCC |
| P. infestans | PITG\_03574 | 286 | Motif-17 | -213 | + | AATGTACCCATTTCG |
| P. infestans | PITG\_03577 | 1000 | Motif-3 | -273 | - | ACTTATGCTTTGTAA |
| P. infestans | PITG\_03578 | 1000 | Motif-0 | -625 | + | TCATTGTTATTTTGGC |
|  |  |  | Motif-4 | -754 | - | TCCGATTGGTTAAAA |
| P. infestans | PITG\_03580 | 1000 | Motif-0 | -926 | + | CCATTTAACATTCTGC |
|  |  |  | Motif-3 | -438 | + | GCTTATGATTTGTAT |
|  |  |  | Motif-6 | -816 | + | TGTCCCCCCCCCCTCT |
| P. infestans | PITG\_03582 | 219 | Motif-4 | -40 | + | GATGATTGGTCATTT |
| P. infestans | PITG\_03583 | 442 | Motif-0 | -60 | + | CACTTTCACATTTGCC |
|  |  |  | Motif-16 | -431 | - | CAGCCGACGCAGCAGC |
| P. infestans | PITG\_03584 | 442 | Motif-0 | -397 | + | GCAAATGTGAAAGTGA |
|  |  |  | Motif-16 | -24 | + | CCGACGCAGCAGCAGC |
| P. infestans | PITG\_03586 | 202 | Motif-3 | -92 | - | CACTAGAGATTTGTAT |
| P. infestans | PITG\_03587 | 202 | Motif-3 | -126 | + | CACTAGAGATTTGTAT |
| P. infestans | PITG\_03588 | 118 | Motif-17 | -71 | - | GATGTAGCCATGTCT |
|  |  |  | Motif-2 | -103 | + | TGGGAAGCTGAAGTTG |
| P. infestans | PITG\_03589 | 417 | Motif-0 | -53 | - | TCACTTCACAATTCG |
|  |  |  | Motif-1 | -64 | + | TACATGTAC |
| P. infestans | PITG\_03590 | 458 | Motif-0 | -18 | + | TCACTCTGTATTTTCA |
|  |  |  | Motif-2 | -219 | + | CAACTTGAAGCG |
|  |  |  | Motif-4 | -171 | + | GTTCATTGGACAAAT |
| P. infestans | PITG\_03591 | 458 | Motif-0 | -456 | - | TCACTCTGTATTTTCA |
|  |  |  | Motif-2 | -251 | - | CAACTTGAAGCG |
|  |  |  | Motif-4 | -302 | - | GTTCATTGGACAAAT |
| P. infestans | PITG\_03593 | 674 | Motif-0 | -64 | - | GCAGGTTCAGGAATGA |
|  |  |  | Motif-2 | -118 | + | GCTTCAACATG |
| P. infestans | PITG\_03594 | 1000 | Motif-0 | -479 | + | TCACTTGCGAATCGGA |
|  |  |  | Motif-1 | -507 | + | TACGTGTAC |
|  |  |  | Motif-2 | -438 | - | CACTTCAACAT |
|  |  |  | Motif-4 | -170 | + | TATTATTGGTCAAAA |
| P. infestans | PITG\_03596 | 1000 | Motif-0 | -551 | + | GCAAGTTCAAAAATGG |
|  |  |  | Motif-3 | -381 | - | ATCTTGGATTTTGTAG |
| P. infestans | PITG\_03597 | 527 | Motif-0 | -278 | - | GGCAGTTTAAGAGTGA |
|  |  |  | Motif-1 | -78 | + | TACATGTAT |
|  |  |  | Motif-2 | -31 | + | GCGCATCAACATCA |
|  |  |  | Motif-7 | -292 | - | CAATATTAATA |
| P. infestans | PITG\_03598 | 527 | Motif-0 | -265 | + | GGCAGTTTAAGAGTGA |
|  |  |  | Motif-1 | -113 | + | TACATGTAA |
|  |  |  | Motif-2 | -186 | - | CACTTTGAAGTG |
|  |  |  | Motif-7 | -246 | + | CAATATTAATA |
| P. infestans | PITG\_03599 | 1000 | Motif-0 | -74 | - | GCGGCTTGAAGAATGA |
|  |  |  | Motif-1 | -424 | + | AGTCCATGTACAAGTG |
|  |  |  | Motif-2 | -776 | - | CTTGTTGAAGTG |
|  |  |  | Motif-7 | -497 | + | TAGTATTAATA |
| P. infestans | PITG\_03600 | 307 | Motif-0 | -294 | - | CCACTGCTGCATTGAC |
|  |  |  | Motif-2 | -120 | + | GCTTCAACCTGTAAAT |
| P. infestans | PITG\_03601 | 307 | Motif-0 | -29 | + | CCACTGCTGCATTGAC |
|  |  |  | Motif-2 | -197 | - | AGCTTCAACCT |
| P. infestans | PITG\_03602 | 429 | Motif-0 | -301 | + | CATTATTCAATTCCCT |
| P. infestans | PITG\_03603 | 350 | Motif-1 | -212 | + | TACATGTAG |
| P. infestans | PITG\_03604 | 165 | Motif-0 | -111 | + | TAATTTTTCGATTTGA |
| P. infestans | PITG\_03605 | 165 | Motif-0 | -70 | - | TAATTTTTCGATTTGA |
| P. infestans | PITG\_03606 | 185 | Motif-0 | -26 | + | TCACTCTTCAGCTTT |
|  |  |  | Motif-3 | -23 | - | ATACAAAGCTGAAGAG |
| P. infestans | PITG\_03607 | 185 | Motif-0 | -174 | - | TCACTCTTCAGCTTT |
|  |  |  | Motif-3 | -178 | + | ATACAAAGCTGAAGAG |
| P. infestans | PITG\_03608 | 173 | Motif-0 | -114 | + | GTAAAATGACGAATGA |
| P. infestans | PITG\_03609 | 173 | Motif-0 | -75 | - | GTAAAATGACGAATGA |
| P. infestans | PITG\_03610 | 1000 | Motif-8 | -181 | + | TTTTTCTAAGCAAAA |
| P. infestans | PITG\_03611 | 575 | Motif-0 | -43 | + | CATTCCTCAATTCACC |
| P. infestans | PITG\_03612 | 689 | Motif-0 | -194 | - | GGAAGTTTAAAAATGA |
|  |  |  | Motif-4 | -573 | - | TGTGATTGGACGAAA |
|  |  |  | Motif-7 | -292 | + | TAGTATTAATA |
| P. infestans | PITG\_03613 | 689 | Motif-0 | -510 | - | TCATTTTTAAACTTC |
|  |  |  | Motif-4 | -131 | + | TGTGATTGGACGAAA |
|  |  |  | Motif-7 | -408 | - | TAGTATTAATA |
| P. infestans | PITG\_03616 | 671 | Motif-0 | -42 | - | GAATATTTCAAAGTGA |
| P. infestans | PITG\_03619 | 1000 | Motif-4 | -56 | - | GCTCATTGGTCATTA |
| P. infestans | PITG\_03620 | 1000 | Motif-0 | -479 | + | TGAGTTGCCAACTTGC |
|  |  |  | Motif-1 | -174 | + | TGCATGTATCATAAA |
| P. infestans | PITG\_03623 | 249 | Motif-2 | -190 | + | GGCATTGAAGTTGC |
| P. infestans | PITG\_03626 | 154 | Motif-4 | -92 | + | ATTCATTGGTTGAAA |
| P. infestans | PITG\_03627 | 676 | Motif-0 | -146 | - | GCAAAATTGGAAATGA |
|  |  |  | Motif-17 | -254 | + | AATGTAGCCATCTAA |
|  |  |  | Motif-4 | -287 | + | TCTGATTGGTGGAAA |
| P. infestans | PITG\_03628 | 676 | Motif-0 | -545 | - | TCATTTCCAATTTTG |
|  |  |  | Motif-17 | -437 | - | AATGTAGCCATCTAA |
|  |  |  | Motif-4 | -404 | - | TCTGATTGGTGGAAA |
| P. infestans | PITG\_03629 | 934 | Motif-1 | -155 | - | CGTACATGTCAT |
| P. infestans | PITG\_03631 | 576 | Motif-0 | -25 | - | CTCACTTTCTGTTTT |
|  |  |  | Motif-1 | -284 | - | GGTACATCTAT |
|  |  |  | Motif-16 | -309 | - | TCGCCGTAGCAGCAGC |
|  |  |  | Motif-2 | -112 | - | ATCTTCAACTT |
| P. infestans | PITG\_03634 | 572 | Motif-0 | -172 | + | TCACTTTTCAAGTAGA |
|  |  |  | Motif-2 | -244 | + | CACTGCAACATAGC |
| P. infestans | PITG\_03635 | 572 | Motif-0 | -58 | + | TCATATCTCGATTTGA |
|  |  |  | Motif-2 | -342 | - | CACTGCAACATAGC |
| P. infestans | PITG\_03638 | 1000 | Motif-0 | -832 | + | TCACTCTTCAACTGAT |
| P. infestans | PITG\_03639 | 1000 | Motif-0 | -73 | - | GCAAAACAGGAAGTGA |
|  |  |  | Motif-2 | -159 | - | GCTTCAACGCG |
| P. infestans | PITG\_03640 | 1000 | Motif-0 | -60 | - | GAAAAACCACGAGTGA |
|  |  |  | Motif-6 | -730 | - | TTCTCCCCCCCCCCCC |
| P. infestans | PITG\_03641 | 150 | Motif-2 | -138 | + | GGAATTGAAGTGAC |
|  |  |  | Motif-4 | -68 | + | TTTGATTGGCTGAAA |
| P. infestans | PITG\_03642 | 150 | Motif-2 | -26 | - | GGAATTGAAGTGAC |
|  |  |  | Motif-4 | -97 | - | TTTGATTGGCTGAAA |
| P. infestans | PITG\_03645 | 220 | Motif-3 | -67 | + | ACTTACGGTTTGTAG |
| P. infestans | PITG\_03646 | 220 | Motif-3 | -167 | + | TACAAACCGTAAGTCA |
| P. infestans | PITG\_03649 | 1000 | Motif-0 | -278 | - | TCATATTTTAACTTAC |
|  |  |  | Motif-1 | -119 | - | TACATGTTTATTAAT |
|  |  |  | Motif-4 | -56 | - | CGTTATTGGATAAAA |
| P. infestans | PITG\_03650 | 1000 | Motif-0 | -276 | - | TCATATTTTAACTTAC |
|  |  |  | Motif-1 | -119 | - | TACATGTTTATTAAT |
|  |  |  | Motif-4 | -56 | - | CGTTATTGGATAAAA |
| P. infestans | PITG\_03651 | 1000 | Motif-4 | -96 | - | TGTGATTGGCTGGTT |
| P. infestans | PITG\_03652 | 1000 | Motif-1 | -238 | + | TACATGTAT |
|  |  |  | Motif-16 | -493 | - | CAGCAGCGGCAGCAAC |
| P. infestans | PITG\_03653 | 616 | Motif-4 | -252 | + | GATGATTGGTCAATA |
|  |  |  | Motif-8 | -517 | - | TTATTTTAAATAAAA |
| P. infestans | PITG\_03655 | 184 | Motif-2 | -111 | + | GCAGTTGAAGTGGA |
| P. infestans | PITG\_03656 | 1000 | Motif-0 | -501 | + | CCAGTCCTCATTTGCC |
|  |  |  | Motif-1 | -180 | - | TGCATGAACAAGTCCT |
|  |  |  | Motif-2 | -94 | + | ACTTCAACATTGGTAA |
|  |  |  | Motif-6 | -580 | + | TCACCTCCCCCGCCAT |
| P. infestans | PITG\_03657 | 511 | Motif-0 | -151 | - | GCACTCGTCAACTTGA |
|  |  |  | Motif-2 | -157 | + | ACAGCCTCAAGTTGAC |
| P. infestans | PITG\_03658 | 323 | Motif-0 | -180 | - | ACGAGTTGAAGAGTGA |
| P. infestans | PITG\_03659 | 323 | Motif-0 | -152 | + | GAAGAGTGAAAAGTGA |
| P. infestans | PITG\_03660 | 360 | Motif-0 | -268 | + | GCACACTTCAACTTAC |
|  |  |  | Motif-2 | -265 | - | TAAGTTGAAGTG |
|  |  |  | Motif-4 | -217 | + | TTTGATTCGATGAAA |
| P. infestans | PITG\_03661 | 690 | Motif-0 | -676 | - | ACAGTCTCAAATCTGC |
|  |  |  | Motif-1 | -137 | + | TACATGTAT |
|  |  |  | Motif-4 | -129 | - | GTGGATTGGCTGAAA |
| P. infestans | PITG\_03662 | 690 | Motif-0 | -30 | + | ACAGTCTCAAATCTGC |
|  |  |  | Motif-1 | -533 | + | TACATGTAT |
|  |  |  | Motif-4 | -224 | + | TTTTTTTGGCTAAAA |
| P. infestans | PITG\_03663 | 1000 | Motif-0 | -26 | + | GCACTTTCAGATCTCC |
|  |  |  | Motif-4 | -614 | - | TGTGATTGGTCGAGT |
|  |  |  | Motif-7 | -254 | - | AACTATTAATA |
|  |  |  | Motif-8 | -303 | - | TTTTTTTAAACAATT |
| P. infestans | PITG\_03665 | 1000 | Motif-2 | -839 | - | GCTGACGTCGAAGTGG |
| P. infestans | PITG\_03669 | 1000 | Motif-1 | -89 | + | TACATGTAA |
|  |  |  | Motif-2 | -791 | + | CACTGCAAAGTGCA |
|  |  |  | Motif-4 | -710 | + | GCTGATTGGATGATT |
| P. infestans | PITG\_03670 | 1000 | Motif-0 | -181 | + | TCACTCTGAGACTCTC |
|  |  |  | Motif-1 | -381 | + | TGCATGTATTGCGGC |
| P. infestans | PITG\_03673 | 424 | Motif-2 | -354 | - | AACTTCAACTT |
|  |  |  | Motif-4 | -341 | - | TCTGATTCGACGAAA |
| P. infestans | PITG\_03674 | 424 | Motif-2 | -81 | - | AAAGTTGAAGTT |
|  |  |  | Motif-4 | -98 | + | TCTGATTCGACGAAA |
| P. infestans | PITG\_03676 | 1000 | Motif-0 | -149 | + | CACTTTTCAATTTGCA |
|  |  |  | Motif-1 | -162 | - | AACATGTAC |
| P. infestans | PITG\_03677 | 1000 | Motif-3 | -29 | + | GAATTACCTTTTGTAT |
| P. infestans | PITG\_03678 | 1000 | Motif-1 | -158 | + | TACATGTAT |
| P. infestans | PITG\_03679 | 1000 | Motif-0 | -56 | + | ACACTTTCCATTTTGA |
| P. infestans | PITG\_03680 | 949 | Motif-1 | -178 | + | TACATGTAA |
|  |  |  | Motif-2 | -538 | - | CGCTTCAACAT |
| P. infestans | PITG\_03683 | 710 | Motif-0 | -72 | + | GCAAGATAAAGACTGA |
| P. infestans | PITG\_03685 | 1000 | Motif-4 | -101 | + | CATAATTGGCTGAAA |
| P. infestans | PITG\_03692 | 398 | Motif-0 | -19 | + | CACGCTTGAATTTGCC |
| P. infestans | PITG\_03693 | 806 | Motif-0 | -793 | - | CCATTCTCGAAATTGA |
|  |  |  | Motif-4 | -717 | + | ACGGATTGGCCGAAA |
| P. infestans | PITG\_03694 | 806 | Motif-0 | -29 | + | CCATTCTCGAAATTGA |
|  |  |  | Motif-4 | -79 | - | TGCGATTGGTCAAAA |
| P. infestans | PITG\_03695 | 925 | Motif-0 | -79 | + | CATTCTTCATTTCGCC |
|  |  |  | Motif-1 | -470 | + | TACATGTAT |
|  |  |  | Motif-4 | -198 | + | TACCATTGGTCAAAA |
| P. infestans | PITG\_03696 | 452 | Motif-2 | -364 | - | TTGGTTGAAGTG |
| P. infestans | PITG\_03697 | 452 | Motif-2 | -100 | + | TTGGTTGAAGTG |
| P. infestans | PITG\_03698 | 1000 | Motif-0 | -62 | + | CATTTCCGAATTGGCC |
|  |  |  | Motif-4 | -246 | - | TTGGATTGGTCAGTA |
| P. infestans | PITG\_03700 | 1000 | Motif-0 | -62 | + | CATTTCCAAATTGGCC |
|  |  |  | Motif-3 | -901 | - | AACGAAGATCAAGTCA |
|  |  |  | Motif-4 | -407 | - | CCTGATTGGTCGATT |
| P. infestans | PITG\_03702 | 135 | Motif-1 | -102 | + | GTAGCCTACGTGTAT |
| P. infestans | PITG\_03703 | 135 | Motif-1 | -48 | - | GTAGCCTACGTGTAT |
| P. infestans | PITG\_03704 | 1000 | Motif-6 | -552 | - | ACCCCCCCCACCACCG |
| P. infestans | PITG\_03707 | 1000 | Motif-17 | -224 | - | AGTGTAGCCATCTGT |
|  |  |  | Motif-3 | -206 | - | CATACAAAACCTAGGT |
|  |  |  | Motif-4 | -808 | - | TTTAGACTAATCACA |
|  |  |  | Motif-8 | -100 | + | TTATATTTATTTTTT |
| P. infestans | PITG\_03709 | 1000 | Motif-2 | -209 | - | CACTTCAACTT |
|  |  |  | Motif-7 | -261 | - | TATTATTAATA |
| P. infestans | PITG\_03710 | 356 | Motif-1 | -331 | + | TACATGTAC |
|  |  |  | Motif-2 | -169 | - | AAGGTTGAAGTG |
| P. infestans | PITG\_03711 | 234 | Motif-1 | -134 | - | TAAATCTACTTGTAT |
| P. infestans | PITG\_03712 | 1000 | Motif-0 | -93 | + | CATTTTGGAATTCACT |
|  |  |  | Motif-1 | -731 | + | TACATGTAT |
| P. infestans | PITG\_03713 | 313 | Motif-0 | -103 | - | GCCAATTGACAAGTGA |
| P. infestans | PITG\_03714 | 158 | Motif-0 | -121 | + | GCAAAATCGAATGTGA |
| P. infestans | PITG\_03715 | 158 | Motif-0 | -53 | - | GCAAAATCGAATGTGA |
| P. infestans | PITG\_03716 | 581 | Motif-4 | -381 | + | GAGGATTGGTCAATT |
| P. infestans | PITG\_03718 | 573 | Motif-1 | -87 | + | TACATGTAG |
|  |  |  | Motif-7 | -419 | - | CACTATTAATA |
| P. infestans | PITG\_03719 | 1000 | Motif-0 | -55 | + | CCAGAATCAAATTTGC |
|  |  |  | Motif-1 | -596 | + | TACATGTAG |
|  |  |  | Motif-2 | -386 | - | CTGCAAGTTGCTGTT |
|  |  |  | Motif-3 | -111 | - | CATTTGGTTTTTGTAT |
| P. infestans | PITG\_03723 | 1000 | Motif-2 | -399 | - | ATCTTCAACTT |
| P. infestans | PITG\_03724 | 1000 | Motif-2 | -358 | + | ACTTCAACATG |
| P. infestans | PITG\_03725 | 1000 | Motif-1 | -983 | + | TACATGTAG |
|  |  |  | Motif-2 | -451 | - | TATCAGGCTGAAGTTG |
|  |  |  | Motif-8 | -502 | - | TAATTTTTAATAGAA |
| P. infestans | PITG\_03726 | 1000 | Motif-0 | -47 | + | CATTTTCCAATTGGCC |
|  |  |  | Motif-1 | -309 | + | TGCATGTATTAACCC |
|  |  |  | Motif-6 | -600 | - | TGACCCCCCCCCCAAA |
| P. infestans | PITG\_03729 | 1000 | Motif-1 | -141 | - | AGTACATGGACATGCA |
|  |  |  | Motif-4 | -682 | + | TATGATTGGTTAATG |
|  |  |  | Motif-8 | -612 | - | TATTATTAAATATCA |
| P. infestans | PITG\_03730 | 1000 | Motif-0 | -55 | + | CATTTTCGGATTTGCT |
|  |  |  | Motif-2 | -392 | + | TTGGTTGAAGTG |
| P. infestans | PITG\_03732 | 1000 | Motif-1 | -357 | + | TACATGTAT |
|  |  |  | Motif-2 | -266 | - | CACTTCAACTT |
| P. infestans | PITG\_03738 | 1000 | Motif-0 | -87 | + | CCATTCCGCATTTTGG |
|  |  |  | Motif-1 | -369 | + | TACATGTAC |
|  |  |  | Motif-16 | -653 | + | CCGCAGCAGCACTAAA |
|  |  |  | Motif-4 | -215 | + | TTCTATTGGCCAAAA |
|  |  |  | Motif-9 | -710 | - | GAGTACCGGTA |
| P. infestans | PITG\_03739 | 1000 | Motif-0 | -853 | + | CATTTCTAAATGCACC |
| P. infestans | PITG\_03740 | 1000 | Motif-1 | -452 | + | TACATGTAA |
|  |  |  | Motif-2 | -935 | + | CTGCAAGTTGCAGGC |
|  |  |  | Motif-6 | -751 | + | CCCCCGCCCTCCCCCA |
| P. infestans | PITG\_03741 | 1000 | Motif-1 | -408 | + | TACATGTAA |
| P. infestans | PITG\_03742 | 849 | Motif-0 | -18 | + | TCACTTCAGTATTCCA |
|  |  |  | Motif-2 | -524 | + | GCTTCAACATG |
| P. infestans | PITG\_03746 | 1000 | Motif-0 | -57 | + | CATTCGCCAATTTGCG |
|  |  |  | Motif-1 | -457 | - | TACATTTACATGTTC |
|  |  |  | Motif-7 | -417 | - | AACTATTAATA |
| P. infestans | PITG\_03747 | 1000 | Motif-0 | -529 | - | GCAATCTGGAGAATGA |
|  |  |  | Motif-1 | -797 | + | TACATGTAC |
| P. infestans | PITG\_03748 | 1000 | Motif-1 | -405 | + | TACATGTAC |
|  |  |  | Motif-2 | -818 | - | CTGCTTCAACGTCA |
|  |  |  | Motif-4 | -256 | - | TGTTATTGGTAGAAA |
| P. infestans | PITG\_03749 | 175 | Motif-17 | -65 | + | AATGTAGCCATCTGT |
| P. infestans | PITG\_03750 | 1000 | Motif-17 | -656 | + | AATGTACCCATTTCA |
|  |  |  | Motif-8 | -740 | + | TATTTTTTTATATTA |
| P. infestans | PITG\_03751 | 1000 | Motif-8 | -990 | - | TATTATTAATTTTTT |
| P. infestans | PITG\_03754 | 692 | Motif-1 | -292 | + | TACATGTAG |
| P. infestans | PITG\_03755 | 562 | Motif-2 | -519 | - | GAAATTGAAGTG |
|  |  |  | Motif-4 | -500 | + | AATCATTGGACAAAA |
| P. infestans | PITG\_03756 | 562 | Motif-2 | -55 | + | GAAATTGAAGTG |
|  |  |  | Motif-4 | -77 | - | AATCATTGGACAAAA |
| P. infestans | PITG\_03758 | 493 | Motif-0 | -372 | + | TCAGTGCGGAATTCGT |
|  |  |  | Motif-4 | -461 | + | TTTAATTGGACAATA |
| P. infestans | PITG\_03759 | 493 | Motif-0 | -87 | - | GCAAGTTGAAGAAGGA |
|  |  |  | Motif-4 | -47 | - | TTTAATTGGACAATA |
| P. infestans | PITG\_03763 | 989 | Motif-1 | -407 | - | AACATGTATACACAC |
|  |  |  | Motif-16 | -282 | - | CCACAGCAGTAGCAGC |
|  |  |  | Motif-18 | -868 | + | TCGTGTGGCGCACACC |
|  |  |  | Motif-4 | -893 | + | CATGATTGGATAAAT |
| P. infestans | PITG\_03764 | 989 | Motif-1 | -597 | + | AACATGTATACACAC |
|  |  |  | Motif-16 | -720 | + | CAGCAGTAGCAGCAGG |
|  |  |  | Motif-18 | -137 | - | TCGTGTGGCGCACACC |
|  |  |  | Motif-4 | -111 | - | CATGATTGGATAAAT |
| P. infestans | PITG\_03765 | 1000 | Motif-0 | -64 | - | GAAAGGTGAAAAGTGA |
| P. infestans | PITG\_03767 | 200 | Motif-3 | -154 | - | TACAACAACCGATTCA |
|  |  |  | Motif-7 | -134 | - | TATTACTAATA |
|  |  |  | Motif-8 | -138 | + | TTATTATTAGTAATA |
| P. infestans | PITG\_03768 | 200 | Motif-3 | -62 | + | TACAACAACCGATTCA |
|  |  |  | Motif-7 | -74 | - | TATTATTAGTA |
|  |  |  | Motif-8 | -77 | - | TTATTATTAGTAATA |
| P. infestans | PITG\_03770 | 1000 | Motif-4 | -65 | - | TTTGATTGGACTAAA |
| P. infestans | PITG\_03772 | 1000 | Motif-1 | -485 | + | TACATGTAA |
|  |  |  | Motif-16 | -702 | - | CAGCAGCTGGACCAGC |
|  |  |  | Motif-2 | -497 | - | CACGTTGAATTG |
| P. infestans | PITG\_03773 | 1000 | Motif-16 | -813 | + | CAGCAGCTGGACCAGC |
|  |  |  | Motif-4 | -378 | - | CGGCATTGGTCGAAA |
| P. infestans | PITG\_03775 | 1000 | Motif-1 | -357 | + | TACATGTAT |
|  |  |  | Motif-2 | -128 | - | ACTCAAGCTGAAATGG |
|  |  |  | Motif-6 | -37 | + | CGTTCTCCCCCCCCAC |
|  |  |  | Motif-8 | -872 | + | TTTTTCTTAGTTTTA |
| P. infestans | PITG\_03776 | 339 | Motif-0 | -310 | + | GCAGTCTCCGTTTTTC |
|  |  |  | Motif-1 | -193 | + | TGCATGTATCGTCTC |
| P. infestans | PITG\_03777 | 217 | Motif-4 | -64 | - | AACGATTGGTAGAAA |
| P. infestans | PITG\_03778 | 217 | Motif-4 | -98 | - | GTTCATTGGACGATA |
| P. infestans | PITG\_03782 | 314 | Motif-1 | -305 | - | TACAAGTACT |
| P. infestans | PITG\_03783 | 557 | Motif-0 | -496 | - | GCATTTTACAACTTT |
|  |  |  | Motif-16 | -368 | + | GAGCAGCGGCAGTAAC |
|  |  |  | Motif-4 | -76 | + | TGCGATTGGGTGAAA |
| P. infestans | PITG\_03784 | 557 | Motif-0 | -76 | + | GCATTTTACAACTTT |
|  |  |  | Motif-16 | -205 | - | GAGCAGCGGCAGTAAC |
|  |  |  | Motif-4 | -496 | - | TGCGATTGGGTGAAA |
| P. infestans | PITG\_03786 | 1000 | Motif-0 | -179 | + | GAAAAAGAAAAAATGA |
| P. infestans | PITG\_03789 | 118 | Motif-4 | -72 | + | TTTAATTGGTCAAAA |
| P. infestans | PITG\_03790 | 118 | Motif-4 | -61 | - | TTTAATTGGTCAAAA |
| P. infestans | PITG\_03791 | 243 | Motif-2 | -190 | + | GAAATTGAAGTG |
|  |  |  | Motif-4 | -134 | + | TCTGATTGGTCATAA |
| P. infestans | PITG\_03792 | 783 | Motif-1 | -252 | + | TACTTGTACA |
|  |  |  | Motif-2 | -669 | - | GAGGAAGTCGAAGCGA |
|  |  |  | Motif-9 | -47 | - | AAGTACCGGTA |
| P. infestans | PITG\_03797 | 1000 | Motif-16 | -571 | - | CAGCAGCAACAGCGAC |
|  |  |  | Motif-2 | -987 | + | CAGGTTGAAGTA |
| P. infestans | PITG\_03798 | 325 | Motif-16 | -313 | + | GTGCAGTAGCACCAGC |
|  |  |  | Motif-2 | -160 | + | TAAGTTGAAGTA |
| P. infestans | PITG\_03802 | 643 | Motif-0 | -469 | - | CCACTTCGGAATCCCC |
| P. infestans | PITG\_03803 | 643 | Motif-0 | -190 | + | CCACTTCGGAATCCCC |
| P. infestans | PITG\_03804 | 1000 | Motif-1 | -310 | + | TACATGTAT |
| P. infestans | PITG\_03808 | 1000 | Motif-8 | -949 | - | TTTTTATAAATTACA |
| P. infestans | PITG\_03810 | 1000 | Motif-8 | -761 | - | TATTTTTAATTTGAT |
| P. infestans | PITG\_03811 | 1000 | Motif-1 | -320 | + | TGCATGTAGCTGTAG |
|  |  |  | Motif-2 | -53 | + | ACTCCAACACG |
|  |  |  | Motif-3 | -161 | - | CATACACAGCGTAAGT |
|  |  |  | Motif-8 | -693 | + | TAATTTTTAATAAAT |
| P. infestans | PITG\_03812 | 836 | Motif-0 | -401 | + | TGAAGTTGCAGAATGA |
|  |  |  | Motif-1 | -192 | + | TACATGTAT |
| P. infestans | PITG\_03813 | 542 | Motif-1 | -229 | + | TACATGTAT |
|  |  |  | Motif-2 | -130 | - | AATGTTGAAGTG |
|  |  |  | Motif-4 | -510 | - | GATGATTGGCTAATT |
| P. infestans | PITG\_03814 | 542 | Motif-1 | -321 | + | TACATGTAG |
|  |  |  | Motif-2 | -423 | - | CACTTCAACAT |
|  |  |  | Motif-4 | -47 | + | GATGATTGGCTAATT |
| P. infestans | PITG\_03815 | 544 | Motif-0 | -19 | + | CACTTCTCAATTCTCC |
|  |  |  | Motif-1 | -513 | - | TGCATGTAC |
|  |  |  | Motif-2 | -159 | + | CAAATTGAAGTG |
|  |  |  | Motif-8 | -316 | + | TTTATTTATATAATA |
| P. infestans | PITG\_03820 | 127 | Motif-0 | -20 | + | TCACTTTTTGAGTGGC |
| P. infestans | PITG\_03822 | 170 | Motif-1 | -146 | + | ACTACGTGTATTACT |
|  |  |  | Motif-4 | -72 | - | TATGATTGGATGTTA |
| P. infestans | PITG\_03823 | 1000 | Motif-1 | -994 | - | AACATGTATTAATAG |
|  |  |  | Motif-2 | -922 | - | CGCTTCAACTT |
|  |  |  | Motif-4 | -806 | + | TATCATTGGCTGCAA |
|  |  |  | Motif-7 | -996 | + | AACTATTAATA |
| P. infestans | PITG\_03824 | 587 | Motif-1 | -446 | + | TACATGCAC |
|  |  |  | Motif-4 | -166 | + | TTTGATTGGTAGATT |
| P. infestans | PITG\_03825 | 587 | Motif-1 | -150 | - | TACATGCAC |
|  |  |  | Motif-4 | -436 | - | TTTGATTGGTAGATT |
| P. infestans | PITG\_03826 | 754 | Motif-2 | -58 | + | GACGTTGCAGTT |
| P. infestans | PITG\_03827 | 754 | Motif-2 | -708 | - | GACGTTGCAGTT |
| P. infestans | PITG\_03828 | 1000 | Motif-1 | -363 | + | TACGGGTACAAGTACC |
|  |  |  | Motif-2 | -204 | - | AACTTCAACTT |
| P. infestans | PITG\_03830 | 937 | Motif-0 | -435 | + | GGAAATGCCAGAATGA |
|  |  |  | Motif-1 | -328 | - | TACTTGTACA |
|  |  |  | Motif-3 | -306 | + | TACAAATCACAAGTCA |
| P. infestans | PITG\_03831 | 298 | Motif-4 | -212 | + | TCTCATTGGCGAAAA |
| P. infestans | PITG\_03832 | 298 | Motif-4 | -64 | + | GCAGATTGGCTGAAA |
| P. infestans | PITG\_03833 | 1000 | Motif-0 | -53 | + | CACTTTGCAACTTGTT |
|  |  |  | Motif-1 | -473 | - | TACCGGTCCATGTAC |
|  |  |  | Motif-2 | -181 | - | CAGGTTGAAGCG |
|  |  |  | Motif-7 | -861 | - | CATTATTAATA |
| P. infestans | PITG\_03834 | 1000 | Motif-1 | -848 | + | TACATGTAG |
| P. infestans | PITG\_03839 | 1000 | Motif-0 | -86 | + | TCATTCCTACATTTTT |
| P. infestans | PITG\_03840 | 320 | Motif-0 | -153 | - | GGAAGTTGGCGACTGA |
|  |  |  | Motif-4 | -99 | + | CGTGATTGGCCCAAA |
| P. infestans | PITG\_03843 | 308 | Motif-17 | -41 | - | GATGTAGCCATTTGT |
|  |  |  | Motif-2 | -292 | + | GCTGCAACTTG |
| P. infestans | PITG\_03844 | 308 | Motif-17 | -282 | + | GATGTAGCCATTTGT |
|  |  |  | Motif-2 | -27 | - | GCTGCAACTTG |
| P. infestans | PITG\_03846 | 1000 | Motif-17 | -107 | - | AGTGTAGCCATACGT |
|  |  |  | Motif-3 | -381 | + | TACAAATTTCTAGTCA |
| P. infestans | PITG\_03850 | 1000 | Motif-18 | -173 | - | TTGTGTAGCGTACATA |
|  |  |  | Motif-4 | -238 | + | CGTGATTGGTCAATA |
|  |  |  | Motif-6 | -862 | - | GCCCCCCCCCTCCAAC |
| P. infestans | PITG\_03854 | 946 | Motif-17 | -259 | + | TGTGTAGTCATCTTT |
|  |  |  | Motif-2 | -297 | + | ACTTCAACTTG |
| P. infestans | PITG\_03855 | 265 | Motif-17 | -205 | + | AATGTAGCCATTTAA |
|  |  |  | Motif-4 | -159 | + | TCTAATTGGTCGAAA |
|  |  |  | Motif-9 | -222 | - | GAGTACCGGTA |
| P. infestans | PITG\_03862 | 414 | Motif-17 | -148 | - | AATGTAGCCATTTTG |
|  |  |  | Motif-8 | -367 | - | TTCTTTTTAATTTAT |
| P. infestans | PITG\_03863 | 414 | Motif-17 | -281 | + | AATGTAGCCATTTTG |
|  |  |  | Motif-8 | -62 | + | TTCTTTTTAATTTAT |
| P. infestans | PITG\_03864 | 354 | Motif-3 | -316 | + | TACAAATTCCAAGTCA |
|  |  |  | Motif-4 | -235 | - | TGTCAGTGGCAAAAA |
| P. infestans | PITG\_03865 | 354 | Motif-3 | -52 | + | ACTTGGAATTTGTAA |
|  |  |  | Motif-4 | -83 | - | TCTGACTGGTTAAAA |
| P. infestans | PITG\_03867 | 171 | Motif-0 | -145 | + | GGCGAATCGAGAATGA |
| P. infestans | PITG\_03868 | 171 | Motif-0 | -42 | - | GGCGAATCGAGAATGA |
| P. infestans | PITG\_03872 | 722 | Motif-1 | -563 | - | TGCATGTAGCCATTT |
|  |  |  | Motif-8 | -604 | + | TTTTTTTTATCTTTT |
| P. infestans | PITG\_03873 | 302 | Motif-0 | -164 | - | TAATTTATCAATTTAC |
|  |  |  | Motif-4 | -271 | + | GATCATTGGCCAAAA |
| P. infestans | PITG\_03874 | 302 | Motif-0 | -154 | + | TAATTTATCAATTTAC |
|  |  |  | Motif-4 | -46 | - | GATCATTGGCCAAAA |
| P. infestans | PITG\_03875 | 338 | Motif-0 | -58 | + | CACTCCTCAACTTGCC |
| P. infestans | PITG\_03876 | 186 | Motif-1 | -115 | - | GATACATAAAC |
| P. infestans | PITG\_03877 | 186 | Motif-1 | -82 | + | GATACATAAAC |
| P. infestans | PITG\_03878 | 125 | Motif-1 | -89 | - | AACATGTATTGATAG |
| P. infestans | PITG\_03879 | 268 | Motif-4 | -230 | - | TCTCATTGGGTAAGT |
| P. infestans | PITG\_03880 | 268 | Motif-4 | -53 | + | TCTCATTGGGTAAGT |
| P. infestans | PITG\_03881 | 448 | Motif-0 | -407 | + | GCAAATTCGAAAATGA |
|  |  |  | Motif-2 | -421 | - | GCTTCAAAGTCGACCA |
|  |  |  | Motif-4 | -235 | - | TCGTATTGGCTAATA |
| P. infestans | PITG\_03882 | 448 | Motif-0 | -56 | + | CATTTTCGAATTTGCT |
|  |  |  | Motif-2 | -43 | + | GCTTCAAAGTCGACCA |
|  |  |  | Motif-4 | -169 | + | CGTGATTGGCGGAAT |
| P. infestans | PITG\_03883 | 298 | Motif-4 | -116 | + | TCTGATTGGAAAATA |
| P. infestans | PITG\_03884 | 298 | Motif-4 | -197 | - | TCTGATTGGAAAATA |
| P. infestans | PITG\_03886 | 222 | Motif-17 | -134 | + | TGTGTAGCCATCTGA |
|  |  |  | Motif-2 | -143 | + | ACTTCAACTTG |
|  |  |  | Motif-4 | -94 | - | CCTGATTGGCTGTTT |
| P. infestans | PITG\_03887 | 1000 | Motif-1 | -9 | - | TACGTGTAC |
|  |  |  | Motif-4 | -904 | + | TTTTATTGTCTAAAA |
| P. infestans | PITG\_03888 | 1000 | Motif-1 | -476 | + | TACATGTAC |
|  |  |  | Motif-4 | -299 | - | TTTTATTGTCTAAAA |
| P. infestans | PITG\_03889 | 892 | Motif-16 | -110 | - | GCGCCGTAGCACCGGC |
|  |  |  | Motif-7 | -858 | + | AAGTATTAATA |
|  |  |  | Motif-8 | -855 | - | TATTTCTTATTAATA |
| P. infestans | PITG\_03890 | 278 | Motif-0 | -189 | + | TTCAATTTCAAATTCA |
|  |  |  | Motif-2 | -157 | + | ATTGTTGAAGTG |
| P. infestans | PITG\_03891 | 278 | Motif-0 | -105 | - | TTCAATTTCAAATTCA |
|  |  |  | Motif-2 | -133 | - | ATTGTTGAAGTG |
| P. infestans | PITG\_03892 | 279 | Motif-3 | -271 | + | ACTTGATATTTGTGT |
| P. infestans | PITG\_03897 | 1000 | Motif-1 | -752 | + | TACATGTAG |
|  |  |  | Motif-4 | -257 | - | GTTGATTGGCCAAAA |
| P. infestans | PITG\_03898 | 1000 | Motif-1 | -494 | + | TACATGTAG |
|  |  |  | Motif-4 | -234 | + | GTCGATTGGCCAAAA |
| P. infestans | PITG\_03899 | 332 | Motif-1 | -12 | + | TACTTGTACA |
| P. infestans | PITG\_03900 | 309 | Motif-0 | -51 | - | GAAGATTGGTGAGTGA |
|  |  |  | Motif-2 | -203 | - | CAGCTTCAACAAA |
| P. infestans | PITG\_03901 | 292 | Motif-17 | -253 | + | GGTGTAGCCATTTCT |
| P. infestans | PITG\_03902 | 249 | Motif-4 | -147 | + | TCGGATTGGTTGAAT |
| P. infestans | PITG\_03903 | 249 | Motif-4 | -117 | - | TCGGATTGGTTGAAT |
| P. infestans | PITG\_03904 | 257 | Motif-0 | -147 | - | TCAGTTCTCGTTCTAT |
| P. infestans | PITG\_03905 | 268 | Motif-18 | -64 | + | TAGTGTGGCGCACAAA |
|  |  |  | Motif-4 | -132 | + | TGGTATTGGCTAAAA |
| P. infestans | PITG\_03906 | 268 | Motif-18 | -220 | - | TAGTGTGGCGCACAAA |
|  |  |  | Motif-4 | -124 | + | TATCATTGGATAAAA |
| P. infestans | PITG\_03910 | 236 | Motif-1 | -75 | + | ACTACATCTACAGTA |
| P. infestans | PITG\_03911 | 927 | Motif-0 | -318 | - | GCGAAAGGGGAAATAA |
|  |  |  | Motif-1 | -464 | - | AGTACATTTACCAGTA |
| P. infestans | PITG\_03912 | 927 | Motif-0 | -625 | + | GCGAAAGGGGAAATAA |
|  |  |  | Motif-1 | -478 | - | TACTGGTAAATGTAC |
| P. infestans | PITG\_03915 | 399 | Motif-0 | -108 | - | TCATTTCTAAATTGAT |
|  |  |  | Motif-4 | -174 | + | TTTGATTAGCTAAAA |
| P. infestans | PITG\_03916 | 399 | Motif-0 | -307 | + | TCATTTCTAAATTGAT |
|  |  |  | Motif-4 | -207 | - | GTGGATTGGCCAAAA |
| P. infestans | PITG\_03918 | 135 | Motif-15 | -53 | - | AACCTCAACCACGC |
| P. infestans | PITG\_03919 | 135 | Motif-15 | -96 | + | AACCTCAACCACGC |
| P. infestans | PITG\_03922 | 1000 | Motif-0 | -82 | - | GCAAGTTTCGGAATGA |
|  |  |  | Motif-1 | -147 | + | TACATGTAT |
|  |  |  | Motif-2 | -992 | + | CAAGTTGAAGTA |
| P. infestans | PITG\_03924 | 259 | Motif-2 | -160 | + | CAACTTGAAGTG |
| P. infestans | PITG\_03926 | 1000 | Motif-0 | -496 | + | TCACTTTCACATTGCA |
|  |  |  | Motif-2 | -596 | - | CTGCTTCAAGATCG |
| P. infestans | PITG\_03929 | 1000 | Motif-3 | -153 | - | CACTTGATTATTGTTT |
|  |  |  | Motif-9 | -30 | - | ATGTACCGGTA |
| P. infestans | PITG\_03930 | 478 | Motif-17 | -427 | + | TGTGTAGCCATTTCG |
| P. infestans | PITG\_03931 | 478 | Motif-17 | -66 | - | TGTGTAGCCATTTCG |
| P. infestans | PITG\_03932 | 735 | Motif-2 | -135 | + | CAACTTCAACAAA |
|  |  |  | Motif-7 | -21 | + | CAGTATTAATA |
| P. infestans | PITG\_03933 | 1000 | Motif-0 | -82 | - | GAAAAAGTGGAAGTGA |
|  |  |  | Motif-4 | -947 | + | TCTGATTGGCCGACT |
| P. infestans | PITG\_03934 | 1000 | Motif-4 | -194 | - | TGCGATTGGCTGATA |
| P. infestans | PITG\_03935 | 1000 | Motif-6 | -125 | - | ACCCCCCCCCCCCGGC |
| P. infestans | PITG\_03936 | 327 | Motif-4 | -180 | - | CTCGATTGGAAGAAA |
| P. infestans | PITG\_03937 | 327 | Motif-4 | -120 | - | TGCGATTGGTGGAAA |
| P. infestans | PITG\_03938 | 254 | Motif-1 | -136 | + | TATACCTGTATTTCA |
|  |  |  | Motif-4 | -89 | + | ATTTATTGGCTAAAA |
| P. infestans | PITG\_03939 | 735 | Motif-4 | -86 | - | TCTTATTGGACAGAA |
| P. infestans | PITG\_03940 | 735 | Motif-4 | -664 | + | TCTTATTGGACAGAA |
| P. infestans | PITG\_03944 | 1000 | Motif-1 | -26 | + | TACATGTAC |
|  |  |  | Motif-9 | -189 | - | CAGTACCGGTA |
| P. infestans | PITG\_03945 | 376 | Motif-2 | -32 | + | TCTTCAACATGCGCGA |
| P. infestans | PITG\_03946 | 376 | Motif-2 | -343 | - | GACTTCAACTT |
| P. infestans | PITG\_03947 | 302 | Motif-0 | -138 | - | TCACTGCTCAAGTTGA |
|  |  |  | Motif-2 | -142 | - | CAAGTTGAAGAG |
| P. infestans | PITG\_03948 | 302 | Motif-0 | -180 | + | TCACTGCTCAAGTTGA |
|  |  |  | Motif-2 | -155 | - | GCATTTCAACGTCA |
| P. infestans | PITG\_03950 | 415 | Motif-0 | -44 | + | CATTCTTCAATTTGCG |
|  |  |  | Motif-1 | -317 | + | TACATGTAG |
|  |  |  | Motif-8 | -192 | + | TTTTTATAATTAATA |
| P. infestans | PITG\_03951 | 300 | Motif-0 | -300 | + | GCAGATAGAAGAATGG |
| P. infestans | PITG\_03952 | 646 | Motif-1 | -205 | + | TACATGTAG |
|  |  |  | Motif-17 | -270 | - | ACTGTAGTCATTCGG |
| P. infestans | PITG\_03953 | 646 | Motif-1 | -449 | + | TACATGTAC |
|  |  |  | Motif-17 | -391 | + | ACTGTAGTCATTCGG |
| P. infestans | PITG\_03954 | 1000 | Motif-0 | -80 | - | CCAAAACGGGGAATGA |
|  |  |  | Motif-4 | -164 | + | CTGGATTGGCCAAAA |
|  |  |  | Motif-6 | -687 | - | ACCCCCCCCCCCTAGC |
| P. infestans | PITG\_03955 | 398 | Motif-1 | -170 | - | AACATCTACCTGTAC |
|  |  |  | Motif-7 | -245 | + | TATTACTAATA |
|  |  |  | Motif-8 | -239 | - | TTTTTTTAAATATTA |
| P. infestans | PITG\_03956 | 808 | Motif-1 | -421 | + | TACATGTTTTGTAGT |
| P. infestans | PITG\_03957 | 808 | Motif-1 | -396 | - | TGCTGCTACATGTTT |
| P. infestans | PITG\_03958 | 387 | Motif-2 | -253 | - | ACTGCAACTCG |
| P. infestans | PITG\_03959 | 387 | Motif-2 | -145 | + | ACTGCAACTCG |
| P. infestans | PITG\_03960 | 1000 | Motif-0 | -70 | + | CACTTTTCCTTTTCTC |
|  |  |  | Motif-1 | -346 | - | TACATGCATT |
|  |  |  | Motif-2 | -297 | - | ACCAAATCAACTTCTA |
| P. infestans | PITG\_03961 | 1000 | Motif-0 | -49 | - | GAGGATGGAAAACTGA |
|  |  |  | Motif-1 | -386 | + | TACATGCAC |
| P. infestans | PITG\_03965 | 1000 | Motif-0 | -475 | - | GAAAAATCAAGAGTGA |
|  |  |  | Motif-1 | -610 | + | TACATGTTTTGTTCC |
|  |  |  | Motif-2 | -38 | - | CCAGTTGAAGTG |
| P. infestans | PITG\_03967 | 170 | Motif-17 | -111 | + | AGTGTAGCCATGTTT |
|  |  |  | Motif-4 | -61 | - | TTTGATTGGACAAAA |
| P. infestans | PITG\_03969 | 94 | Motif-17 | -89 | + | AATGTAGCCATGTGT |
| P. infestans | PITG\_03972 | 252 | Motif-0 | -23 | + | TTATTCTCCGACTTAC |
| P. infestans | PITG\_03973 | 252 | Motif-0 | -245 | - | TTATTCTCCGACTTAC |
| P. infestans | PITG\_03975 | 1000 | Motif-0 | -332 | - | GCATTTCCAATTCTTC |
| P. infestans | PITG\_03977 | 1000 | Motif-1 | -349 | - | CATACATATAG |
|  |  |  | Motif-2 | -98 | - | ACTGCAACTCG |
|  |  |  | Motif-8 | -823 | - | TTTTTTTATGTATAA |
| P. infestans | PITG\_03978 | 176 | Motif-0 | -28 | - | TAGAAATGAAGAGTGA |
|  |  |  | Motif-8 | -124 | + | TTTAATTTTTTAAAA |
| P. infestans | PITG\_03979 | 146 | Motif-0 | -83 | - | GATAAGTGGAGAATGA |
| P. infestans | PITG\_03980 | 1000 | Motif-4 | -302 | - | TGTCATTGGAGGAAT |
|  |  |  | Motif-7 | -995 | - | CACTATTAATA |
| P. infestans | PITG\_03985 | 1000 | Motif-0 | -956 | - | CCACTCCCGAATGTGC |
|  |  |  | Motif-2 | -544 | + | GACTTTGAAGTTGC |
| P. infestans | PITG\_03986 | 1000 | Motif-12 | -422 | + | GCGTGCCGGTGGTGG |
| P. infestans | PITG\_03987 | 1000 | Motif-0 | -487 | + | GCAACTTGCATAGTGA |
|  |  |  | Motif-4 | -385 | + | GTGGATTGGTCAAAA |
| P. infestans | PITG\_03988 | 663 | Motif-1 | -335 | + | TACATGTAC |
|  |  |  | Motif-2 | -623 | + | ACATCAACGTGTGGCA |
|  |  |  | Motif-3 | -219 | + | ATTTGGCTTTTGTAA |
| P. infestans | PITG\_03989 | 150 | Motif-2 | -137 | + | CACGTTGAAGCA |
|  |  |  | Motif-4 | -57 | + | GCTGATTCGTCGAAA |
| P. infestans | PITG\_03990 | 150 | Motif-2 | -24 | + | GCTTCAACGTG |
|  |  |  | Motif-4 | -78 | + | TCGGATTGGATGAAA |
| P. infestans | PITG\_03991 | 343 | Motif-0 | -321 | - | TCATTTTTCCAGTCGA |
| P. infestans | PITG\_03992 | 1000 | Motif-1 | -147 | - | CGTGGATGTATTG |
| P. infestans | PITG\_03994 | 706 | Motif-0 | -552 | - | GAAAAATTGAAAATGC |
|  |  |  | Motif-2 | -602 | - | TAAATTGAAGTTGA |
|  |  |  | Motif-8 | -515 | - | TTTTTCTTAGCAAAT |
| P. infestans | PITG\_03995 | 706 | Motif-0 | -170 | + | GAAAAATTGAAAATGC |
|  |  |  | Motif-2 | -17 | + | TCACTTCAAGTTTC |
|  |  |  | Motif-8 | -206 | + | TTTTTCTTAGCAAAT |
| P. infestans | PITG\_03996 | 313 | Motif-2 | -104 | - | GACTTCAACAT |
| P. infestans | PITG\_03998 | 344 | Motif-3 | -89 | + | ACTTGGTTTTTGTAA |
|  |  |  | Motif-4 | -120 | - | TCTGATTGGTTGTGT |
| P. infestans | PITG\_03999 | 344 | Motif-3 | -269 | + | TACAAAAACCAAGTAA |
|  |  |  | Motif-4 | -239 | + | TCTGATTGGTTGTGT |
| P. infestans | PITG\_04000 | 166 | Motif-4 | -112 | + | TGTGATTGGCTATTT |
| P. infestans | PITG\_04001 | 166 | Motif-4 | -69 | - | TGTGATTGGCTATTT |
| P. infestans | PITG\_04002 | 307 | Motif-2 | -295 | + | ACTTCAACTTG |
|  |  |  | Motif-4 | -78 | - | TTTCATTGGCTACAT |
| P. infestans | PITG\_04005 | 1000 | Motif-1 | -516 | + | TACATGTAA |
| P. infestans | PITG\_04006 | 1000 | Motif-0 | -62 | - | GGAAATTGGGGAATAA |
|  |  |  | Motif-1 | -537 | + | TACACGTACCGGTAC |
|  |  |  | Motif-2 | -82 | + | AACTTCGACTT |
|  |  |  | Motif-9 | -534 | + | ACGTACCGGTA |
| P. infestans | PITG\_04010 | 1000 | Motif-0 | -66 | + | CCATTTGCTAATCTGC |
|  |  |  | Motif-4 | -233 | + | CTCGATCGGCTAAAA |
|  |  |  | Motif-8 | -916 | + | TAATATTTATTTTAT |
| P. infestans | PITG\_04012 | 1000 | Motif-1 | -404 | - | TATAGATGTATTG |
|  |  |  | Motif-2 | -106 | + | CACATTGAAGTG |
| P. infestans | PITG\_04013 | 334 | Motif-1 | -31 | + | TACATGTAC |
| P. infestans | PITG\_04021 | 435 | Motif-1 | -153 | + | TACATGTAT |
| P. infestans | PITG\_04023 | 1000 | Motif-0 | -442 | - | TCATTTTTAATCTCG |
|  |  |  | Motif-7 | -410 | - | TACTATTAATA |
|  |  |  | Motif-8 | -559 | - | TAAATTTAAATTTTA |
| P. infestans | PITG\_04024 | 1000 | Motif-2 | -344 | - | GCTTCAACACG |
| P. infestans | PITG\_04027 | 373 | Motif-1 | -166 | + | TACATGTAC |
|  |  |  | Motif-3 | -34 | + | TTACAAAATTCAAGCT |
| P. infestans | PITG\_04028 | 373 | Motif-1 | -215 | + | TACATGTAC |
|  |  |  | Motif-3 | -355 | - | TTACAAAATTCAAGCT |
| P. infestans | PITG\_04031 | 145 | Motif-3 | -39 | + | ACTCAAAGTTTGTAT |
| P. infestans | PITG\_04032 | 145 | Motif-3 | -121 | - | CACTCAAAGTTTGTAT |
| P. infestans | PITG\_04033 | 1000 | Motif-0 | -63 | - | GCAGAATGACGACTGA |
|  |  |  | Motif-1 | -859 | - | AATACCGGTACAATA |
|  |  |  | Motif-4 | -243 | + | TTTCATTGGTCAAGT |
|  |  |  | Motif-8 | -535 | + | TATTTTTTAATATAA |
| P. infestans | PITG\_04034 | 1000 | Motif-0 | -964 | + | GCAGAATGACGACTGA |
|  |  |  | Motif-1 | -167 | + | AATACCGGTACAATA |
|  |  |  | Motif-4 | -783 | - | TTTCATTGGTCAAGT |
|  |  |  | Motif-8 | -491 | - | TATTTTTTAATATAA |
| P. infestans | PITG\_04035 | 513 | Motif-0 | -368 | + | TCACTATGGATTTTAA |
|  |  |  | Motif-17 | -173 | - | AATGTAGCCATTTCG |
| P. infestans | PITG\_04037 | 1000 | Motif-0 | -301 | + | GACAAATGACAAATGA |
|  |  |  | Motif-2 | -135 | + | ACTTCAACTTG |
|  |  |  | Motif-3 | -120 | + | TTACAAAATGCAAGAT |
| P. infestans | PITG\_04038 | 124 | Motif-17 | -85 | + | AATGTAGCCATGTCA |
| P. infestans | PITG\_04043 | 1000 | Motif-1 | -243 | + | TACATGTAG |
| P. infestans | PITG\_04044 | 1000 | Motif-0 | -622 | - | GGAAGATTCGAAATGA |
|  |  |  | Motif-18 | -681 | + | TATTGTGGTGCACACT |
|  |  |  | Motif-2 | -312 | + | GCTCCAACTTG |
|  |  |  | Motif-4 | -959 | + | TATCAGCGAATGGGA |
| P. infestans | PITG\_04049 | 1000 | Motif-0 | -67 | - | GCAAATAGTAAAGTGA |
|  |  |  | Motif-1 | -171 | - | TACATATAC |
|  |  |  | Motif-4 | -454 | - | GTGTATTGGTCAATA |
| P. infestans | PITG\_04050 | 1000 | Motif-0 | -758 | - | GCACTATTCAATTAGC |
|  |  |  | Motif-1 | -563 | + | TACATGTAT |
| P. infestans | PITG\_04052 | 1000 | Motif-0 | -55 | - | GTAGATTCTAGAGTGA |
|  |  |  | Motif-1 | -64 | + | TACATGTGCT |
|  |  |  | Motif-8 | -139 | + | TTTTTTTTATTTGTT |
| P. infestans | PITG\_04053 | 657 | Motif-0 | -195 | + | TCATTCCGACTTCTAC |
| P. infestans | PITG\_04054 | 561 | Motif-1 | -242 | + | TACATGTAC |
|  |  |  | Motif-2 | -547 | + | GTGGTTGACGTG |
|  |  |  | Motif-9 | -330 | - | CCGTACCGGTA |
| P. infestans | PITG\_04056 | 1000 | Motif-1 | -124 | + | TGCATGTAC |
|  |  |  | Motif-4 | -319 | - | TCTGATTGGTTGAAG |
| P. infestans | PITG\_04057 | 1000 | Motif-0 | -63 | - | GCTGATTGGGAAATGA |
|  |  |  | Motif-9 | -300 | - | AGGTACCGGTA |
| P. infestans | PITG\_04058 | 947 | Motif-1 | -866 | + | GATACCGGTACCAGTA |
|  |  |  | Motif-4 | -744 | - | GGTGATTGGTTAAAG |
| P. infestans | PITG\_04059 | 947 | Motif-1 | -97 | - | GATACCGGTACCAGTA |
|  |  |  | Motif-4 | -218 | + | GGTGATTGGTTAAAG |
| P. infestans | PITG\_04061 | 176 | Motif-0 | -19 | - | GCGGATCCAAGAGTGA |
| P. infestans | PITG\_04062 | 176 | Motif-0 | -173 | + | GCGGATCCAAGAGTGA |
| P. infestans | PITG\_04063 | 760 | Motif-0 | -55 | - | GTAGATTCTAGAGTGA |
|  |  |  | Motif-1 | -64 | + | TACATGTGCT |
|  |  |  | Motif-8 | -125 | + | TTTTATTTTTTTTAT |
| P. infestans | PITG\_04065 | 1000 | Motif-0 | -63 | + | CATTCCTGCATTTGCG |
|  |  |  | Motif-17 | -817 | + | ACTGTACCCATTTTG |
|  |  |  | Motif-4 | -250 | + | GCCGATTGGTCGAAA |
|  |  |  | Motif-8 | -649 | + | TATATTTTATCTTAA |
| P. infestans | PITG\_04070 | 1000 | Motif-0 | -940 | + | GCAAATGTAGAAGTGG |
| P. infestans | PITG\_04071 | 1000 | Motif-0 | -43 | - | GCGAATTGAATTGTGA |
| P. infestans | PITG\_04075 | 319 | Motif-0 | -48 | + | TCATTTCCTCTTTGAC |
|  |  |  | Motif-3 | -224 | - | ATTTGTTTTTAGTAT |
| P. infestans | PITG\_04077 | 1000 | Motif-0 | -795 | + | TCAGTAGTCATCTCGC |
|  |  |  | Motif-1 | -867 | + | TACATGCAC |
|  |  |  | Motif-16 | -941 | + | AAACAGCAGGACCAAC |
|  |  |  | Motif-4 | -988 | + | GTCGATTGGATGAAA |
| P. infestans | PITG\_04078 | 1000 | Motif-0 | -323 | - | TCAGTAGTCATCTCGC |
|  |  |  | Motif-1 | -243 | + | TGCATGTATAAACGC |
|  |  |  | Motif-16 | -177 | - | AAACAGCAGGACCAAC |
|  |  |  | Motif-4 | -102 | + | TACCATTGGCCAAAA |
| P. infestans | PITG\_04081 | 1000 | Motif-0 | -64 | - | GGAAAACGAGAAATGG |
|  |  |  | Motif-1 | -189 | + | TACATGTAA |
|  |  |  | Motif-9 | -142 | - | CAGTACCGGTA |
| P. infestans | PITG\_04085 | 814 | Motif-0 | -66 | - | GGAAAACGCGAAGTGA |
|  |  |  | Motif-1 | -455 | + | TACGTGTAC |
|  |  |  | Motif-3 | -648 | + | ACTTGATATTAGTAA |
|  |  |  | Motif-4 | -590 | + | TCTGATTGGCCAAAC |
| P. infestans | PITG\_04086 | 814 | Motif-0 | -67 | - | GGAAAACGCGAAGTGA |
|  |  |  | Motif-1 | -368 | - | TACGTGTAC |
|  |  |  | Motif-3 | -181 | - | GACTTGATATTAGTAA |
|  |  |  | Motif-4 | -239 | - | TCTGATTGGCCAAAC |
| P. infestans | PITG\_04090 | 1000 | Motif-0 | -64 | - | GGAAAACGCGAAGTGA |
|  |  |  | Motif-1 | -448 | + | TACGTGTAC |
|  |  |  | Motif-3 | -640 | + | ACTTGATATCAGTAA |
|  |  |  | Motif-4 | -582 | + | TCTGATTGGCCAAAC |
| P. infestans | PITG\_04091 | 1000 | Motif-16 | -565 | - | TAACAGCAGCACCGAC |
| P. infestans | PITG\_04093 | 1000 | Motif-1 | -181 | - | GTACTGTACATGTGC |
|  |  |  | Motif-7 | -751 | - | AAATATTAATA |
| P. infestans | PITG\_04096 | 1000 | Motif-6 | -332 | + | CCCCCCCCCACCTTGT |
| P. infestans | PITG\_04097 | 1000 | Motif-0 | -66 | - | GAAAAACGTGAAGTGA |
|  |  |  | Motif-1 | -283 | + | TACATGTAA |
|  |  |  | Motif-8 | -477 | + | TACTATTAATTTTTT |
| P. infestans | PITG\_04098 | 1000 | Motif-0 | -529 | - | GCAATCTGGAGAATGA |
|  |  |  | Motif-1 | -668 | - | AGGACATGTAC |
|  |  |  | Motif-4 | -758 | + | TGCCATTGGCCATAA |
| P. infestans | PITG\_04099 | 1000 | Motif-0 | -48 | - | AGAAATCGGAAAGTGA |
|  |  |  | Motif-1 | -277 | - | TACATATAC |
|  |  |  | Motif-16 | -529 | + | CAGCAGGAGCAGCAGC |
|  |  |  | Motif-2 | -726 | + | CAAGTTCAAGTT |
|  |  |  | Motif-6 | -832 | - | ACCCCCTCCTCCTCCA |
| P. infestans | PITG\_04104 | 1000 | Motif-0 | -203 | + | CCATTTCCACTTCGGC |
| P. infestans | PITG\_04105 | 1000 | Motif-4 | -277 | + | GCTGATTGGTCAAAC |
| P. infestans | PITG\_04109 | 376 | Motif-1 | -258 | - | GTACTGTACGTGTTT |
| P. infestans | PITG\_04110 | 511 | Motif-1 | -447 | + | ATTACCGGTACAGTA |
|  |  |  | Motif-7 | -177 | + | TATTATTAATA |
|  |  |  | Motif-9 | -445 | - | CTGTACCGGTA |
| P. infestans | PITG\_04111 | 511 | Motif-1 | -79 | - | ATTACCGGTACAGTA |
|  |  |  | Motif-7 | -345 | - | TATTATTAATA |
|  |  |  | Motif-9 | -77 | + | CTGTACCGGTA |
| P. infestans | PITG\_04114 | 1000 | Motif-0 | -61 | + | TCAGTCTCGATATCTC |
|  |  |  | Motif-1 | -457 | - | TACATGTGCCGATAC |
| P. infestans | PITG\_04115 | 1000 | Motif-3 | -371 | - | CACTTGAATTTTTTAT |
|  |  |  | Motif-4 | -987 | + | TGTGATTGGATAAAA |
| P. infestans | PITG\_04116 | 1000 | Motif-4 | -488 | - | TGTGATTGGATAAAA |
| P. infestans | PITG\_04117 | 1000 | Motif-0 | -289 | + | TCATTAATAAACTTTC |
|  |  |  | Motif-2 | -47 | + | ACTTCAAGATG |
| P. infestans | PITG\_04121 | 1000 | Motif-8 | -691 | + | TTTTTTTTAGTTTTT |
| P. infestans | PITG\_04122 | 958 | Motif-1 | -497 | + | TACATGTAT |
|  |  |  | Motif-8 | -165 | - | TAAATTTTAATTAAA |
|  |  |  | Motif-9 | -513 | + | CAGTACCGGTA |
| P. infestans | PITG\_04124 | 744 | Motif-0 | -52 | - | GACAGTTGAGAAGTGA |
|  |  |  | Motif-2 | -177 | + | CAACTTCATCCTC |
| P. infestans | PITG\_04125 | 789 | Motif-0 | -87 | - | GAAAATTGGGGAATGA |
|  |  |  | Motif-1 | -411 | + | TACATGTAT |
| P. infestans | PITG\_04126 | 1000 | Motif-0 | -61 | + | CATTACTCAATTCGCT |
|  |  |  | Motif-1 | -480 | + | TACATGTAG |
| P. infestans | PITG\_04127 | 1000 | Motif-0 | -540 | + | CTCACTTTTTTTTTT |
|  |  |  | Motif-2 | -428 | + | AGTGTTGAAGTGGC |
| P. infestans | PITG\_04129 | 1000 | Motif-0 | -57 | - | GCAAATAGAGGAATGA |
|  |  |  | Motif-1 | -136 | + | TACATGTAC |
|  |  |  | Motif-2 | -198 | + | GCTTCAACATG |
|  |  |  | Motif-4 | -586 | + | GGGGATTGGGCAAAA |
| P. infestans | PITG\_04130 | 1000 | Motif-0 | -56 | + | CATTCTTCTATTTGCC |
|  |  |  | Motif-1 | -126 | + | TACATGTAC |
|  |  |  | Motif-2 | -202 | + | GCTTCAACATG |
|  |  |  | Motif-6 | -687 | + | CGACCCCCCCCGGACC |
| P. infestans | PITG\_04132 | 1000 | Motif-0 | -84 | + | TCCTTCTGCAACTTAC |
|  |  |  | Motif-1 | -124 | - | AATACCGGTACAGAA |
|  |  |  | Motif-9 | -122 | + | CTGTACCGGTA |
| P. infestans | PITG\_04133 | 143 | Motif-0 | -77 | - | GAAAATAGAATAGTGA |
| P. infestans | PITG\_04134 | 1000 | Motif-0 | -44 | + | TCATTCACGCATCTTC |
|  |  |  | Motif-2 | -578 | - | GTACTTCAAGTTCA |
|  |  |  | Motif-3 | -116 | + | ATCAAATTTCAACTCA |
| P. infestans | PITG\_04135 | 1000 | Motif-0 | -48 | - | GAGAATTCGGGAATGA |
|  |  |  | Motif-1 | -188 | + | GATGCATGAAC |
|  |  |  | Motif-8 | -354 | + | TATTTTTAAACAAGT |
| P. infestans | PITG\_04136 | 1000 | Motif-8 | -42 | - | TTTAATTTATTTATT |
| P. infestans | PITG\_04139 | 1000 | Motif-0 | -386 | + | GGGGATTGATAAATGA |
|  |  |  | Motif-2 | -862 | - | CACATTGAAGTG |
|  |  |  | Motif-4 | -791 | - | TTTGATTGGTTATAA |
|  |  |  | Motif-8 | -636 | - | TTTGTTTTTTTTAAA |
| P. infestans | PITG\_04141 | 1000 | Motif-0 | -75 | - | GAAGATTGGAGAATGA |
|  |  |  | Motif-1 | -387 | + | TACATGTAC |
|  |  |  | Motif-8 | -992 | - | TTTTTTTTAATAAAT |
| P. infestans | PITG\_04142 | 1000 | Motif-9 | -493 | + | CGGTACCGGTA |
| P. infestans | PITG\_04143 | 1000 | Motif-2 | -975 | - | GATATTGAAGTT |
| P. infestans | PITG\_04145 | 1000 | Motif-0 | -55 | + | TCACTTGACTTTTTGC |
|  |  |  | Motif-1 | -385 | + | TACATGTAT |
|  |  |  | Motif-6 | -516 | - | TCTGCCCCCCCCTCCC |
|  |  |  | Motif-9 | -968 | + | AGGTACCGGTA |
| P. infestans | PITG\_04146 | 1000 | Motif-2 | -424 | + | GCTGCAACATG |
| P. infestans | PITG\_04148 | 1000 | Motif-0 | -62 | + | TCACATTAGATTTTGC |
| P. infestans | PITG\_04149 | 556 | Motif-0 | -131 | - | GAAAATTGAGGAATGA |
|  |  |  | Motif-18 | -506 | - | TGGTGTGGCCCACACT |
|  |  |  | Motif-2 | -513 | - | CACTTCAACGT |
| P. infestans | PITG\_04150 | 556 | Motif-0 | -440 | - | TCATTCCTCAATTTT |
|  |  |  | Motif-18 | -66 | + | TGGTGTGGCCCACACT |
|  |  |  | Motif-2 | -53 | + | ACTTCAACGTTGACAC |
| P. infestans | PITG\_04152 | 1000 | Motif-0 | -489 | + | GCAAAAGAAAGAGTGA |
| P. infestans | PITG\_04153 | 1000 | Motif-0 | -55 | + | TCACTTGACTTTTCGC |
|  |  |  | Motif-1 | -631 | + | TACATGTAA |
| P. infestans | PITG\_04156 | 1000 | Motif-1 | -338 | + | TACATGTAT |
|  |  |  | Motif-9 | -649 | - | AGGTACCGGTA |
| P. infestans | PITG\_04158 | 1000 | Motif-0 | -78 | - | GAAGGTTGGAGAGTGA |
|  |  |  | Motif-1 | -693 | + | TACATGTAA |
| P. infestans | PITG\_04164 | 1000 | Motif-0 | -60 | + | CAGTTCTAAACTTACC |
|  |  |  | Motif-1 | -807 | + | TACATGTAA |
|  |  |  | Motif-4 | -574 | - | CTTTATTGGTCATAT |
|  |  |  | Motif-7 | -988 | + | TACTATTAATA |
| P. infestans | PITG\_04165 | 1000 | Motif-0 | -60 | + | CAGTTCTAAACTTACC |
|  |  |  | Motif-1 | -807 | + | TACATGTAA |
|  |  |  | Motif-4 | -574 | - | CTTTATTGGTCATAT |
|  |  |  | Motif-7 | -988 | + | TACTATTAATA |
| P. infestans | PITG\_04169 | 1000 | Motif-1 | -478 | + | TACCTGTACAACCAT |
|  |  |  | Motif-2 | -852 | + | CAAGTTGCAGTG |
|  |  |  | Motif-4 | -491 | + | GGTGATTGGATGATA |
| P. infestans | PITG\_04172 | 1000 | Motif-0 | -131 | + | CACTTTAGCATTTGCA |
|  |  |  | Motif-1 | -475 | - | CACACATGTAGAATA |
|  |  |  | Motif-2 | -228 | - | ACTGCAACTTC |
|  |  |  | Motif-6 | -825 | - | GGACCCCCCCCCCTCC |
| P. infestans | PITG\_04173 | 1000 | Motif-0 | -120 | + | CCACATCTCAATTCAC |
|  |  |  | Motif-4 | -713 | + | GTTGATTGGTTGAAA |
| P. infestans | PITG\_04174 | 1000 | Motif-0 | -25 | - | GACAAATCAAAAGTGA |
| P. infestans | PITG\_04175 | 1000 | Motif-0 | -26 | - | GAAAATTGTCGACTGA |
|  |  |  | Motif-1 | -669 | + | TACATGTAA |
|  |  |  | Motif-4 | -783 | + | TTTCAGCTAATGATC |
|  |  |  | Motif-7 | -84 | + | CACTATTAATA |
| P. infestans | PITG\_04178 | 1000 | Motif-0 | -61 | + | TCAGATCCCAACTTAC |
|  |  |  | Motif-1 | -388 | + | TACATGTAG |
| P. infestans | PITG\_04179 | 1000 | Motif-8 | -142 | - | TAATTTTAAATATTT |
| P. infestans | PITG\_04182 | 1000 | Motif-0 | -32 | + | CACTCTTCAATTTACT |
|  |  |  | Motif-1 | -116 | + | TACATGTAC |
| P. infestans | PITG\_04184 | 1000 | Motif-0 | -49 | + | CACTTCCGAACTTCCG |
|  |  |  | Motif-18 | -500 | + | TGGTTTGGTGTACAGA |
|  |  |  | Motif-4 | -529 | + | GCGGATTGGTAGAAA |
| P. infestans | PITG\_04194 | 1000 | Motif-0 | -65 | + | TCACTTCACGTTTTCA |
|  |  |  | Motif-1 | -730 | + | TACATGTAC |
| P. infestans | PITG\_04196 | 1000 | Motif-0 | -41 | + | CCATTCCGGTATTTAC |
|  |  |  | Motif-1 | -416 | - | AGAACATGTATTTCT |
| P. infestans | PITG\_04200 | 1000 | Motif-0 | -123 | - | TCATTCTTAATCTTCT |
|  |  |  | Motif-4 | -921 | + | TATGATTGGATGAAG |
|  |  |  | Motif-8 | -386 | + | TATGTTTTAGTTTTA |
| P. infestans | PITG\_04202 | 1000 | Motif-0 | -37 | - | TCAAGTTGAGAAGTGA |
|  |  |  | Motif-1 | -220 | + | TACATGTAT |
|  |  |  | Motif-9 | -442 | - | ATGTTCCGGTA |
| P. infestans | PITG\_04204 | 1000 | Motif-0 | -82 | + | TCATTTCGAAAGTGAC |
|  |  |  | Motif-2 | -736 | + | GGTCAAGTTGAAACCC |
| P. infestans | PITG\_04207 | 1000 | Motif-0 | -172 | + | CACTTTCGATTTTGTC |
| P. infestans | PITG\_04208 | 1000 | Motif-1 | -196 | + | TACATGCATT |
| P. infestans | PITG\_04209 | 1000 | Motif-3 | -127 | + | TACAAATATCGTGTCA |
| P. infestans | PITG\_04213 | 1000 | Motif-0 | -37 | - | TCAAGTTGAGAAGTGA |
|  |  |  | Motif-1 | -240 | + | TACATGTAT |
|  |  |  | Motif-6 | -555 | - | CCCGCCCCCCGCCCCC |
|  |  |  | Motif-9 | -257 | - | ATGTACCGGTA |
| P. infestans | PITG\_04215 | 1000 | Motif-2 | -355 | + | GCTTCAACCTGCGAAA |
|  |  |  | Motif-4 | -431 | + | ATATAGCCAATTACA |
| P. infestans | PITG\_04222 | 1000 | Motif-3 | -300 | - | CACTTGTAATCTGTAT |
| P. infestans | PITG\_04224 | 1000 | Motif-1 | -857 | - | TACATGAAC |
|  |  |  | Motif-4 | -20 | + | TCCAATTGGATAATA |
| P. infestans | PITG\_04226 | 334 | Motif-0 | -325 | + | GAGAATCTGCAAATGA |
|  |  |  | Motif-4 | -247 | + | CATGATTGGTGGAAA |
| P. infestans | PITG\_04227 | 334 | Motif-0 | -25 | - | GAGAATCTGCAAATGA |
|  |  |  | Motif-4 | -102 | - | CATGATTGGTGGAAA |
| P. infestans | PITG\_04228 | 258 | Motif-0 | -153 | + | CACTCCTCCACTTCCC |
| P. infestans | PITG\_04229 | 258 | Motif-0 | -120 | + | GGAAGTGGAGGAGTGA |
| P. infestans | PITG\_04230 | 260 | Motif-0 | -248 | + | GGAAAAGTCGGAATGA |
|  |  |  | Motif-4 | -111 | - | GGTGATTGGTCAAAA |
| P. infestans | PITG\_04231 | 260 | Motif-0 | -28 | - | GGAAAAGTCGGAATGA |
|  |  |  | Motif-4 | -164 | + | GGTGATTGGTCAAAA |
| P. infestans | PITG\_04232 | 1000 | Motif-0 | -70 | + | CACTCTCCAAGTTGCC |
|  |  |  | Motif-3 | -261 | + | ACTGGGAGTTTGTAT |
| P. infestans | PITG\_04236 | 1000 | Motif-0 | -673 | + | TCATTAATAAACTTTC |
| P. infestans | PITG\_04243 | 1000 | Motif-0 | -248 | + | TCATATATGATTTTGC |
|  |  |  | Motif-4 | -122 | + | TTACAGCCAATCTCA |
| P. infestans | PITG\_04246 | 1000 | Motif-1 | -346 | - | GCTACCTACGTGTAT |
|  |  |  | Motif-6 | -192 | - | TCCCCCCCCCCCGCCT |
| P. infestans | PITG\_04248 | 300 | Motif-0 | -58 | + | TCATTCATCAGTTTTC |
| P. infestans | PITG\_04249 | 300 | Motif-0 | -258 | - | TCATTCATCAGTTTTC |
| P. infestans | PITG\_04250 | 1000 | Motif-0 | -531 | + | GTGACTTGGAAAATGA |
|  |  |  | Motif-2 | -581 | + | TCGCTTCACCGTC |
|  |  |  | Motif-7 | -66 | + | TAGTATTAATA |
| P. infestans | PITG\_04251 | 1000 | Motif-1 | -693 | + | ATTAAATGTATAATA |
|  |  |  | Motif-4 | -902 | + | TCTTATTGGCAGAAA |
|  |  |  | Motif-8 | -861 | + | TTTTATTAAATATTT |
| P. infestans | PITG\_04254 | 1000 | Motif-2 | -85 | - | CGACTTCAAGGCGA |
| P. infestans | PITG\_04255 | 1000 | Motif-0 | -44 | + | CACTTTCGATTTTGTC |
|  |  |  | Motif-3 | -525 | + | ACTTGAATTAAGTAT |
| P. infestans | PITG\_04262 | 1000 | Motif-0 | -916 | + | TCATTATGGAAGTTGC |
|  |  |  | Motif-1 | -892 | + | TACATGTAC |
|  |  |  | Motif-2 | -161 | + | CAGATTGAAGTG |
|  |  |  | Motif-4 | -48 | - | TGCGATTGGCTAAAT |
|  |  |  | Motif-6 | -530 | + | ACCCCCCCCCCCTTGG |
|  |  |  | Motif-7 | -848 | + | TATTATTAATA |
|  |  |  | Motif-9 | -872 | + | GTGTACCGGTA |
| P. infestans | PITG\_04263 | 606 | Motif-0 | -23 | - | GAAGATCGCAAAATGA |
|  |  |  | Motif-1 | -111 | + | TACATGTAA |
|  |  |  | Motif-8 | -107 | - | TATTAATTAATTACA |
| P. infestans | PITG\_04264 | 755 | Motif-9 | -265 | - | ATGTACCGGTA |
| P. infestans | PITG\_04265 | 1000 | Motif-0 | -87 | - | CACTTCCTCATGTCCC |
| P. infestans | PITG\_04269 | 1000 | Motif-1 | -35 | + | TACATGTAA |
|  |  |  | Motif-8 | -323 | + | TTAATTTAATTTATA |
| P. infestans | PITG\_04270 | 1000 | Motif-0 | -30 | + | TCATTCGCAAATATTC |
|  |  |  | Motif-1 | -412 | + | TACATGAAC |
|  |  |  | Motif-7 | -160 | - | CAGTATTAATA |
| P. infestans | PITG\_04271 | 1000 | Motif-1 | -712 | + | TACATGTAT |
|  |  |  | Motif-4 | -586 | + | TGTGATTGACCGAAA |
| P. infestans | PITG\_04275 | 1000 | Motif-6 | -572 | - | AACCCCCCCCCCTCAC |
| P. infestans | PITG\_04276 | 1000 | Motif-0 | -216 | + | TCATTTCAAAATTTCT |
|  |  |  | Motif-1 | -290 | - | AATACCGGTACTGCA |
|  |  |  | Motif-16 | -364 | - | TAACAGCAGCACCAGC |
|  |  |  | Motif-9 | -288 | + | CAGTACCGGTA |
| P. infestans | PITG\_04279 | 1000 | Motif-0 | -48 | + | TCAGTTCGCAAATCAC |
|  |  |  | Motif-1 | -147 | + | TACATGTAT |
|  |  |  | Motif-2 | -336 | - | GAGGTTGAAGTT |
|  |  |  | Motif-6 | -527 | + | CCAACCCCCCCCTAAA |
|  |  |  | Motif-8 | -370 | + | TATTTCTAAACTTAA |
| P. infestans | PITG\_04281 | 1000 | Motif-1 | -416 | + | AACATGTAGATATCT |
| P. infestans | PITG\_04283 | 1000 | Motif-0 | -46 | + | TCAGTCGTCAGTTCGC |
|  |  |  | Motif-1 | -531 | + | TACATGTAT |
|  |  |  | Motif-17 | -170 | - | TCTGTAGCCATGTCA |
|  |  |  | Motif-3 | -105 | + | TCTTGAGTTTACTAT |
|  |  |  | Motif-4 | -74 | - | TTTTGGCGAATTAAA |
| P. infestans | PITG\_04286 | 1000 | Motif-17 | -440 | - | AATGTAGCCATCTCG |
| P. infestans | PITG\_04288 | 1000 | Motif-0 | -598 | - | TCCAATTCGCGAGTGA |
|  |  |  | Motif-3 | -927 | - | AACTTGGAATGTGTAT |
|  |  |  | Motif-4 | -361 | + | GTTTATTGGTCATTA |
|  |  |  | Motif-8 | -320 | + | TAATATTAAGTAACA |
|  |  |  | Motif-9 | -560 | - | CGGTACCGGTA |
| P. infestans | PITG\_04290 | 1000 | Motif-0 | -55 | + | TCACTTGACTTTTTCC |
| P. infestans | PITG\_04291 | 1000 | Motif-0 | -675 | - | GAGGATTGATGAGTGA |
|  |  |  | Motif-2 | -886 | - | CGGCATCAACTTGC |
|  |  |  | Motif-3 | -714 | - | GACTTGGCATTAGTTT |
|  |  |  | Motif-9 | -768 | + | GAGTACCGGTA |
| P. infestans | PITG\_04294 | 1000 | Motif-1 | -550 | + | TACATGCAC |
|  |  |  | Motif-8 | -490 | + | TAATTTTAATTAAAA |
| P. infestans | PITG\_04295 | 276 | Motif-0 | -188 | + | CCACTTCAACATTTTC |
|  |  |  | Motif-2 | -186 | + | ACTTCAACATTTTCAA |
|  |  |  | Motif-4 | -176 | - | GTAGATTGGTTGAAA |
| P. infestans | PITG\_04296 | 1000 | Motif-0 | -53 | + | TCATTATCCTTTCTGC |
|  |  |  | Motif-4 | -502 | + | TATGATTGGCTGATA |
| P. infestans | PITG\_04297 | 1000 | Motif-8 | -461 | + | TATTACTAAATAATT |
| P. infestans | PITG\_04300 | 1000 | Motif-0 | -34 | - | GCAAAACGAGAACTGG |
|  |  |  | Motif-1 | -363 | + | ATTACACGTACCGGCA |
|  |  |  | Motif-6 | -982 | - | ACCCCCCGCCCCTCCA |
| P. infestans | PITG\_04301 | 1000 | Motif-0 | -194 | - | GCAAATCGTAAAATGA |
|  |  |  | Motif-1 | -339 | + | TACATGTGCCAGAAAT |
|  |  |  | Motif-2 | -597 | - | ACCTTCAACGT |
| P. infestans | PITG\_04303 | 1000 | Motif-0 | -54 | - | GCAGATCCAAGAATGA |
|  |  |  | Motif-1 | -80 | - | ACTACGTGTATCACA |
|  |  |  | Motif-2 | -90 | - | ATCACATCAAGTTGTG |
|  |  |  | Motif-8 | -232 | - | TTTTTTTAAGTTGTA |
| P. infestans | PITG\_04310 | 1000 | Motif-4 | -423 | + | TATGACTGGATGAAA |
| P. infestans | PITG\_04314 | 1000 | Motif-1 | -519 | + | TGCATGTAC |
|  |  |  | Motif-3 | -876 | - | ACTTAGTTTTACTAA |
| P. infestans | PITG\_04315 | 1000 | Motif-0 | -180 | + | GCAAAATAAAGAGTGC |
|  |  |  | Motif-1 | -219 | - | TACGTGTAC |
|  |  |  | Motif-4 | -728 | + | TGAGATTGGCTAAAT |
| P. infestans | PITG\_04316 | 1000 | Motif-2 | -723 | + | TCAAGTTCAACGTGCA |
|  |  |  | Motif-3 | -173 | + | ACTGAGTGTTTGTAA |
| P. infestans | PITG\_04317 | 1000 | Motif-0 | -62 | + | TCACTTTAAATTCGCC |
|  |  |  | Motif-2 | -698 | + | GTTGTTGAAGCT |
| P. infestans | PITG\_04320 | 1000 | Motif-0 | -49 | + | CACTTTTGAATTTGCC |
|  |  |  | Motif-1 | -90 | + | TACATGTTCTCATTT |
|  |  |  | Motif-2 | -302 | - | AACTTCAACTT |
| P. infestans | PITG\_04321 | 1000 | Motif-0 | -38 | + | TCATTTTGCCTTCTCA |
|  |  |  | Motif-1 | -185 | - | TGCATGTATCTTCGC |
|  |  |  | Motif-3 | -574 | + | ACTTGAAATTTGTAA |
|  |  |  | Motif-9 | -459 | + | ATGTACCGGTA |
| P. infestans | PITG\_04324 | 841 | Motif-0 | -46 | - | GAAGATCGCAGAGTGA |
|  |  |  | Motif-1 | -780 | - | GCTACCGGTACTGTA |
|  |  |  | Motif-9 | -778 | + | CAGTACCGGTA |
| P. infestans | PITG\_04325 | 841 | Motif-0 | -46 | + | TCACTTTGTGTTTCTC |
|  |  |  | Motif-1 | -76 | + | GCTACCGGTACTGTA |
|  |  |  | Motif-9 | -74 | - | CAGTACCGGTA |
| P. infestans | PITG\_04326 | 1000 | Motif-0 | -49 | - | GAAAATTCCACAATGA |
|  |  |  | Motif-1 | -232 | + | TACTTGTACT |
| P. infestans | PITG\_04328 | 594 | Motif-1 | -497 | + | ACTACAGGTACACTG |
|  |  |  | Motif-2 | -311 | + | CAAATTGAAGTT |
| P. infestans | PITG\_04329 | 1000 | Motif-0 | -49 | - | GAAAATGCCACAATGA |
|  |  |  | Motif-1 | -232 | + | TACTTGTACT |
| P. infestans | PITG\_04333 | 265 | Motif-0 | -20 | + | TCATTCGACAATCTCC |
|  |  |  | Motif-4 | -179 | + | GATAATTGGTTGATA |
| P. infestans | PITG\_04334 | 265 | Motif-0 | -261 | - | TCATTCGACAATCTCC |
|  |  |  | Motif-4 | -101 | - | GATAATTGGTTGATA |
| P. infestans | PITG\_04335 | 256 | Motif-1 | -217 | + | TACACGTACTGCTCT |
|  |  |  | Motif-2 | -112 | - | AAAGTTGAAGCG |
| P. infestans | PITG\_04337 | 1000 | Motif-1 | -204 | + | TGTACATGGACAAATA |
|  |  |  | Motif-2 | -866 | + | ACATCAACATTGGCAA |
|  |  |  | Motif-6 | -996 | - | CGTACCCCCCCCTCCC |
|  |  |  | Motif-8 | -119 | - | TTTTTTTAAATATTT |
| P. infestans | PITG\_04339 | 1000 | Motif-0 | -36 | - | GAAAATGAAGGAGTGA |
|  |  |  | Motif-1 | -483 | + | TACATGAAC |
| P. infestans | PITG\_04340 | 1000 | Motif-0 | -208 | - | GTAAATGTAAAAATGC |
|  |  |  | Motif-1 | -585 | + | TACATGTAC |
| P. infestans | PITG\_04341 | 1000 | Motif-0 | -180 | - | ACATTTTCGCATTTAC |
|  |  |  | Motif-1 | -417 | + | TACATGCAC |
|  |  |  | Motif-18 | -240 | - | TGTGGTGGTGTACGAA |
| P. infestans | PITG\_04342 | 1000 | Motif-1 | -481 | + | TGCATGTAC |
|  |  |  | Motif-2 | -989 | - | CACTTCGACTT |
|  |  |  | Motif-3 | -277 | - | GACTTGTGTTCAGTAT |
| P. infestans | PITG\_04343 | 1000 | Motif-0 | -962 | - | GCTAATCGAAAAGTGC |
| P. infestans | PITG\_04344 | 1000 | Motif-0 | -247 | - | TCACTTCACAAATTT |
|  |  |  | Motif-2 | -176 | + | CACTTTGAAGTG |
| P. infestans | PITG\_04345 | 1000 | Motif-0 | -265 | - | TCACTTTGCAATTTG |
|  |  |  | Motif-2 | -441 | - | GACGTTGAAGCT |
| P. infestans | PITG\_04350 | 1000 | Motif-0 | -288 | + | TCATTTTCATAACTGC |
|  |  |  | Motif-1 | -398 | + | TACATGCAC |
| P. infestans | PITG\_04351 | 1000 | Motif-0 | -53 | + | CACTTCCCAATTTTCC |
| P. infestans | PITG\_04352 | 1000 | Motif-4 | -917 | - | TTTAATTGGGTAATA |
|  |  |  | Motif-6 | -76 | - | TGCCCCCCCCCCTCCT |
| P. infestans | PITG\_04353 | 1000 | Motif-0 | -63 | + | TCACAACTGAATTTAC |
|  |  |  | Motif-1 | -189 | + | TACATGCAC |
| P. infestans | PITG\_04354 | 1000 | Motif-0 | -61 | - | GATAATTGAGAAGTGA |
|  |  |  | Motif-1 | -223 | - | AACATGTAC |
|  |  |  | Motif-2 | -629 | - | AATGTTGACGTG |
|  |  |  | Motif-6 | -34 | + | CCCCCTCCCCCCGTGC |
|  |  |  | Motif-9 | -136 | - | AGGTACCGGTA |
| P. infestans | PITG\_04355 | 1000 | Motif-0 | -55 | + | CATTCCTCAATTTACC |
|  |  |  | Motif-1 | -652 | - | TACATGAAC |
| P. infestans | PITG\_04356 | 1000 | Motif-3 | -824 | - | GTCTTGCCTTTTGTAT |
| P. infestans | PITG\_04358 | 1000 | Motif-0 | -35 | - | CTCAATTCGAAAATGA |
| P. infestans | PITG\_04359 | 427 | Motif-8 | -56 | - | TTTGATTTATTATAA |
| P. infestans | PITG\_04360 | 1000 | Motif-1 | -117 | - | GCTACCGGTACAAGAA |
|  |  |  | Motif-7 | -340 | - | AAATATTAATA |
| P. infestans | PITG\_04365 | 1000 | Motif-1 | -507 | + | GTTTGTTACATGTTT |
|  |  |  | Motif-4 | -827 | + | GTTGATTGGATATGA |
| P. infestans | PITG\_04371 | 1000 | Motif-0 | -536 | - | GGAAATCGGCAAGTGC |
|  |  |  | Motif-1 | -793 | - | TGCGTGTACTTTTAAC |
|  |  |  | Motif-2 | -87 | + | GATGTTGCAGTT |
| P. infestans | PITG\_04372 | 382 | Motif-0 | -228 | + | TCATTATCAAATTCTA |
|  |  |  | Motif-3 | -110 | - | TACTTAATATTTGTAT |
|  |  |  | Motif-4 | -37 | - | TTTGATTGGATAAAA |
| P. infestans | PITG\_04373 | 1000 | Motif-0 | -49 | - | GAAAATTGATGAATGG |
|  |  |  | Motif-1 | -251 | + | AACATGTAC |
| P. infestans | PITG\_04374 | 1000 | Motif-0 | -267 | - | TCACTCCAAAATTCT |
| P. infestans | PITG\_04375 | 1000 | Motif-0 | -832 | - | GTAACTTCCAAAGTGA |
|  |  |  | Motif-4 | -252 | - | TCTGATAGGCTAATA |
| P. infestans | PITG\_04376 | 1000 | Motif-4 | -121 | - | CATGATTCGTTAAAA |
| P. infestans | PITG\_04377 | 1000 | Motif-4 | -964 | + | CATGATTCGTTAAAA |
| P. infestans | PITG\_04378 | 1000 | Motif-0 | -67 | + | CATTCTGCAATTTGCC |
|  |  |  | Motif-1 | -343 | - | TAGCATTACATGTTC |
|  |  |  | Motif-2 | -164 | + | CATGTTGCAGTT |
| P. infestans | PITG\_04379 | 768 | Motif-0 | -91 | - | GCGAGATGCAAAATGG |
|  |  |  | Motif-1 | -159 | + | TACATGTAC |
|  |  |  | Motif-7 | -546 | + | AAGTATTAATA |
| P. infestans | PITG\_04380 | 768 | Motif-0 | -693 | + | GCGAGATGCAAAATGG |
|  |  |  | Motif-1 | -426 | + | TACATGTAT |
|  |  |  | Motif-7 | -233 | - | AAGTATTAATA |
| P. infestans | PITG\_04381 | 754 | Motif-0 | -39 | + | GCATTTGCGAACTTCC |
|  |  |  | Motif-1 | -659 | + | TACATGTAT |
| P. infestans | PITG\_04384 | 1000 | Motif-4 | -92 | + | TTTCATTGGTTGAAA |
|  |  |  | Motif-7 | -322 | - | CACTATTAATA |
|  |  |  | Motif-8 | -747 | - | TTCTTTTAAGCTAAA |
| P. infestans | PITG\_04385 | 1000 | Motif-0 | -60 | + | CATTCTCACTTTTACC |
|  |  |  | Motif-1 | -244 | - | ACAACAGGTACAGTA |
|  |  |  | Motif-2 | -123 | + | ACTTCAACATC |
|  |  |  | Motif-6 | -538 | + | GACCCCCCCCCCTCAT |
| P. infestans | PITG\_04386 | 1000 | Motif-0 | -64 | + | TCATTCCGATTTTCAC |
|  |  |  | Motif-1 | -554 | + | TACATGTAG |
|  |  |  | Motif-2 | -171 | + | CAAGTTGATGTG |
| P. infestans | PITG\_04387 | 1000 | Motif-0 | -53 | + | TCATTCACCAATTTCT |
|  |  |  | Motif-8 | -273 | + | TTTTTTTAAGTTTCT |
|  |  |  | Motif-9 | -998 | + | CTGTACCGGTA |
| P. infestans | PITG\_04388 | 1000 | Motif-0 | -38 | - | GTAAATCTGGAACTGA |
|  |  |  | Motif-1 | -392 | + | TACATGTAC |
|  |  |  | Motif-2 | -442 | + | CATATTGAAGCG |
|  |  |  | Motif-7 | -459 | - | TACTATTAATA |
| P. infestans | PITG\_04390 | 1000 | Motif-8 | -999 | + | TATTTCTTAATAATA |
| P. infestans | PITG\_04393 | 1000 | Motif-8 | -437 | - | TATTTTAAAGTAAAA |
|  |  |  | Motif-9 | -501 | - | ACGTACCGGTA |
| P. infestans | PITG\_04394 | 1000 | Motif-0 | -126 | + | GAAAATTCAAAACTGG |
|  |  |  | Motif-8 | -241 | - | TATTTTTAAGCAACA |
| P. infestans | PITG\_04395 | 1000 | Motif-0 | -190 | + | ACATTTTGGCTTTTGC |
| P. infestans | PITG\_04396 | 1000 | Motif-0 | -52 | + | TCATTTCCACTTTCAA |
|  |  |  | Motif-7 | -379 | + | AACTATTAATA |
| P. infestans | PITG\_04397 | 284 | Motif-1 | -208 | - | AACATGTAGCCATGT |
|  |  |  | Motif-17 | -210 | - | CATGTAGCCATGTCG |
| P. infestans | PITG\_04398 | 1000 | Motif-1 | -480 | - | AATGCAGGTACAGTA |
|  |  |  | Motif-3 | -322 | - | CACTTGATCTTTGTAA |
| P. infestans | PITG\_04399 | 624 | Motif-1 | -210 | + | TACATGTAT |
| P. infestans | PITG\_04401 | 1000 | Motif-0 | -146 | - | TTACTCTACAATTTG |
| P. infestans | PITG\_04402 | 1000 | Motif-0 | -396 | - | GAGGATTCAGGAATGA |
| P. infestans | PITG\_04403 | 1000 | Motif-1 | -112 | + | TACATGTAG |
|  |  |  | Motif-3 | -222 | - | CATACAAAACGCACAT |
|  |  |  | Motif-4 | -383 | + | TTTGATTGGTGGAAT |
|  |  |  | Motif-7 | -488 | - | CACTATTAATA |
|  |  |  | Motif-9 | -118 | - | ATGTACCGGTA |
| P. infestans | PITG\_04404 | 1000 | Motif-0 | -45 | - | GCAATTTCGAAAGTGG |
|  |  |  | Motif-1 | -459 | + | TACATGTAT |
| P. infestans | PITG\_04405 | 1000 | Motif-0 | -526 | - | TCACTGTTGCTTTGTC |
|  |  |  | Motif-1 | -651 | + | TACATGTAT |
|  |  |  | Motif-2 | -72 | - | GAGCGTCTTGCAGTC |
| P. infestans | PITG\_04407 | 1000 | Motif-0 | -36 | + | CACTTTGGATTTCGCC |
| P. infestans | PITG\_04410 | 1000 | Motif-0 | -168 | - | ACATTCTCTAAGTTGC |
|  |  |  | Motif-1 | -501 | - | TACATGAAC |
| P. infestans | PITG\_04414 | 1000 | Motif-2 | -243 | - | CATATTGAAGTG |
| P. infestans | PITG\_04415 | 1000 | Motif-2 | -978 | + | CATATTGAAGTG |
| P. infestans | PITG\_04418 | 550 | Motif-3 | -220 | + | ATACGGATCACAAGAC |
| P. infestans | PITG\_04419 | 550 | Motif-3 | -346 | - | ATACGGATCACAAGAC |
| P. infestans | PITG\_04421 | 1000 | Motif-0 | -68 | + | TCACTTTTCTTTTTCG |
|  |  |  | Motif-1 | -892 | - | TGCACGTACAAGTAT |
|  |  |  | Motif-8 | -505 | - | TTTTAATAATTAAGA |
| P. infestans | PITG\_04424 | 1000 | Motif-1 | -61 | + | TACATGCAC |
| P. infestans | PITG\_04425 | 1000 | Motif-3 | -38 | + | TCTTGATATCAGTAT |
| P. infestans | PITG\_04430 | 1000 | Motif-0 | -415 | - | TTATTTGCCATTTTGA |
|  |  |  | Motif-1 | -948 | + | TACATGTAG |
| P. infestans | PITG\_04432 | 1000 | Motif-0 | -537 | - | TCAGTGCTCGATTGAC |
|  |  |  | Motif-4 | -751 | - | ACCCATTGGTAAAAA |
| P. infestans | PITG\_04434 | 1000 | Motif-0 | -293 | + | GTAAAATGGACAATGG |
| P. infestans | PITG\_04436 | 1000 | Motif-0 | -982 | + | GTAAAATGGACAATGG |
| P. infestans | PITG\_04437 | 1000 | Motif-0 | -843 | + | TCATATTTCAGTTTG |
| P. infestans | PITG\_04439 | 1000 | Motif-1 | -646 | + | TACATGCATA |
|  |  |  | Motif-4 | -979 | - | CCTGATTGGACAAAT |
|  |  |  | Motif-7 | -223 | - | TACTACTAATA |
| P. infestans | PITG\_04440 | 1000 | Motif-0 | -45 | - | GCAAATACTGAAATGA |
| P. infestans | PITG\_04441 | 265 | Motif-0 | -46 | - | GTCAAATGGATAATGA |
|  |  |  | Motif-4 | -176 | - | TGTGATTGGTGGAAA |
|  |  |  | Motif-8 | -123 | - | TATTTTTAAGCTTAA |
| P. infestans | PITG\_04442 | 265 | Motif-0 | -235 | + | GTCAAATGGATAATGA |
|  |  |  | Motif-4 | -104 | + | TGTGATTGGTGGAAA |
|  |  |  | Motif-8 | -157 | + | TATTTTTAAGCTTAA |
| P. infestans | PITG\_04443 | 1000 | Motif-0 | -665 | + | GAGAATTCTAAAGTGA |
|  |  |  | Motif-1 | -282 | - | TACATGCAC |
|  |  |  | Motif-4 | -105 | - | CCGGATTGGTTGATA |
| P. infestans | PITG\_04444 | 1000 | Motif-0 | -956 | - | GAGAATTCTAAAGTGA |
| P. infestans | PITG\_04445 | 260 | Motif-0 | -103 | - | GCAAATTGGACAGCGA |
| P. infestans | PITG\_04446 | 972 | Motif-1 | -272 | + | TACATGTAC |
|  |  |  | Motif-4 | -323 | - | AATCATTGGCTAAAA |
|  |  |  | Motif-7 | -788 | - | TACTACTAATA |
| P. infestans | PITG\_04447 | 1000 | Motif-1 | -308 | + | TACATGTGCA |
|  |  |  | Motif-7 | -985 | - | TACTATTAATA |
| P. infestans | PITG\_04448 | 1000 | Motif-0 | -509 | - | CAGTCGGTCATTTGCC |
|  |  |  | Motif-1 | -117 | + | TACATGTAC |
|  |  |  | Motif-4 | -407 | + | TGTGATTGGTCAATA |
|  |  |  | Motif-9 | -114 | + | ATGTACCGGTA |
| P. infestans | PITG\_04449 | 407 | Motif-0 | -48 | - | GAGAAATGGAAAATGA |
|  |  |  | Motif-1 | -164 | + | TACATGTAC |
|  |  |  | Motif-8 | -222 | + | TATTTATTAATAAGT |
| P. infestans | PITG\_04450 | 407 | Motif-0 | -24 | - | GGGAAACTGGAAATGA |
|  |  |  | Motif-1 | -251 | + | TACATGTAC |
|  |  |  | Motif-8 | -200 | - | TATTTATTAATAAGT |
| P. infestans | PITG\_04452 | 840 | Motif-4 | -39 | - | TCTCATTGTTTGATA |
| P. infestans | PITG\_04453 | 840 | Motif-4 | -816 | + | TCTCATTGTTTGATA |
| P. infestans | PITG\_04454 | 1000 | Motif-1 | -177 | + | TACATGTAT |
| P. infestans | PITG\_04456 | 1000 | Motif-2 | -967 | - | CCATCAACGTGACCAA |
| P. infestans | PITG\_04457 | 1000 | Motif-1 | -853 | - | GATACGTGTAG |
|  |  |  | Motif-3 | -466 | + | ACTTGACTTCTGTAT |
| P. infestans | PITG\_04458 | 317 | Motif-0 | -75 | - | GTAGAACGAGGAATGA |
|  |  |  | Motif-2 | -155 | - | GGCTTCAACGT |
| P. infestans | PITG\_04462 | 1000 | Motif-4 | -43 | - | TCTGATTGGATACCA |
| P. infestans | PITG\_04464 | 1000 | Motif-1 | -460 | - | GCAGCATGTACAACCA |
|  |  |  | Motif-3 | -362 | + | GCTTGATATTACTAT |
|  |  |  | Motif-8 | -307 | - | TTTGTTTTTTTTAAA |
| P. infestans | PITG\_04465 | 1000 | Motif-1 | -873 | - | TTTATATGTAT |
|  |  |  | Motif-6 | -380 | + | CCCCCTCCCACCTTCT |
| P. infestans | PITG\_04466 | 1000 | Motif-1 | -161 | + | TTTATATGTAT |
|  |  |  | Motif-6 | -656 | - | AGCCCCCCTCCCACCT |
| P. infestans | PITG\_04467 | 1000 | Motif-2 | -372 | + | GCTCACGCTGAAGCTG |
| P. infestans | PITG\_04468 | 1000 | Motif-0 | -980 | + | CATTCCACAATTTGCG |
|  |  |  | Motif-1 | -158 | + | TACATGTAT |
| P. infestans | PITG\_04470 | 1000 | Motif-1 | -457 | - | TACATATAC |
| P. infestans | PITG\_04471 | 1000 | Motif-1 | -457 | - | TACATATAC |
| P. infestans | PITG\_04472 | 1000 | Motif-0 | -42 | + | ATCTCATTTTCATTTT |
|  |  |  | Motif-4 | -178 | - | TTTTATTGGCTAATT |
|  |  |  | Motif-8 | -763 | + | TTTTTTTAAACTTTT |
| P. infestans | PITG\_04473 | 1000 | Motif-0 | -492 | - | GCAAAACCAGAAATGA |
|  |  |  | Motif-1 | -745 | - | TAATACTACTTGTAG |
| P. infestans | PITG\_04475 | 1000 | Motif-0 | -584 | + | GCACTCCGAGTTTCTC |
|  |  |  | Motif-1 | -439 | + | AATACATGCAGTGGA |
|  |  |  | Motif-17 | -905 | + | GGTGTAGCCATGCGA |
|  |  |  | Motif-8 | -25 | + | TTTGTTTAAATAATA |
|  |  |  | Motif-9 | -790 | - | AGGTACCGGTA |
| P. infestans | PITG\_04478 | 1000 | Motif-0 | -49 | + | CATTCCCACATTCACC |
|  |  |  | Motif-2 | -446 | - | ACTTCAACGCG |
| P. infestans | PITG\_04479 | 1000 | Motif-1 | -117 | + | TACATGCAC |
|  |  |  | Motif-4 | -65 | + | TTTGATTGGCTAAAC |
| P. infestans | PITG\_04481 | 462 | Motif-2 | -292 | - | AACTTCAACTT |
| P. infestans | PITG\_04482 | 300 | Motif-0 | -43 | + | CACTTCTCTATTTGCC |
|  |  |  | Motif-2 | -136 | - | GTGGTTGAAGCG |
|  |  |  | Motif-4 | -93 | - | TCTGATTGGACATTA |
| P. infestans | PITG\_04485 | 331 | Motif-0 | -48 | - | CCACTTTCTTATTGGC |
| P. infestans | PITG\_04486 | 331 | Motif-0 | -124 | - | TCATTCTAAAATCTGA |
| P. infestans | PITG\_04487 | 755 | Motif-0 | -80 | - | TCATTGGTCAATTTGT |
|  |  |  | Motif-4 | -77 | - | GATCATTGGTCAATT |
|  |  |  | Motif-8 | -89 | + | TAATATTAAACAAAT |
| P. infestans | PITG\_04490 | 356 | Motif-0 | -53 | + | TCACAATTCAATTTAC |
|  |  |  | Motif-1 | -170 | + | TACATGTAA |
|  |  |  | Motif-8 | -282 | + | TATTTTTTAATATAT |
| P. infestans | PITG\_04491 | 1000 | Motif-3 | -39 | - | TACTTAAATTTTGTAT |
|  |  |  | Motif-4 | -61 | + | TCTCATTGGCTGTAA |
| P. infestans | PITG\_04494 | 1000 | Motif-4 | -486 | + | TCTGATTGGTTGCTA |
| P. infestans | PITG\_04497 | 1000 | Motif-18 | -100 | - | TGGTGTGGCGCACACA |
| P. infestans | PITG\_04498 | 1000 | Motif-2 | -565 | - | CCACTTCATCTTC |
| P. infestans | PITG\_04505 | 140 | Motif-1 | -134 | + | TACATGTAC |
| P. infestans | PITG\_04511 | 1000 | Motif-8 | -98 | - | TATTTTTATTTAAAT |
| P. infestans | PITG\_04512 | 796 | Motif-6 | -630 | - | GGCCCTCCCCACTCAC |
| P. infestans | PITG\_04515 | 1000 | Motif-18 | -947 | - | TATTGTGGCCCACACA |
|  |  |  | Motif-8 | -672 | - | TTTTTTTATATTTAT |
| P. infestans | PITG\_04522 | 1000 | Motif-0 | -661 | + | CACTACGGAATTTGCT |
|  |  |  | Motif-3 | -531 | - | ATACACATGTCAAATG |
| P. infestans | PITG\_04523 | 855 | Motif-2 | -322 | - | CCGCATCAACCTCA |
| P. infestans | PITG\_04524 | 855 | Motif-2 | -547 | + | CCGCATCAACCTCA |
| P. infestans | PITG\_04529 | 252 | Motif-0 | -207 | - | GCACTATCGAAGTTGC |
|  |  |  | Motif-4 | -82 | - | CACGATTGGCCAACT |
| P. infestans | PITG\_04530 | 252 | Motif-0 | -61 | + | GCACTATCGAAGTTGC |
|  |  |  | Motif-4 | -185 | + | CACGATTGGCCAACT |
| P. infestans | PITG\_04532 | 1000 | Motif-1 | -685 | - | CATACCGGTACATACA |
|  |  |  | Motif-4 | -47 | + | ATTGATTGGTTTAAA |
|  |  |  | Motif-7 | -162 | - | AAGTATTAATA |
|  |  |  | Motif-9 | -682 | + | ATGTACCGGTA |
| P. infestans | PITG\_04533 | 1000 | Motif-1 | -257 | + | TACATGTAA |
|  |  |  | Motif-16 | -575 | + | CCACAGCAGCAGTAAC |
|  |  |  | Motif-7 | -675 | - | CATTATTAATA |
|  |  |  | Motif-9 | -979 | - | ATGTACCGGTA |
| P. infestans | PITG\_04534 | 1000 | Motif-0 | -501 | - | TCACTTTCAGATTTT |
|  |  |  | Motif-1 | -163 | + | TACATGTAC |
|  |  |  | Motif-2 | -41 | + | CACTCCAACTT |
|  |  |  | Motif-4 | -52 | + | TTTGATTGGCTCACT |
| P. infestans | PITG\_04538 | 1000 | Motif-0 | -408 | + | TCACTCAAAAATTTCA |
|  |  |  | Motif-1 | -277 | + | TACATGCATT |
|  |  |  | Motif-3 | -539 | - | ACTTGATTATACTAT |
| P. infestans | PITG\_04539 | 1000 | Motif-2 | -822 | - | CGCGTCAACTCGCA |
|  |  |  | Motif-3 | -164 | + | TACAAACCCTGAGTCA |
| P. infestans | PITG\_04540 | 1000 | Motif-0 | -44 | + | CCAGTTTGCAATTTCA |
| P. infestans | PITG\_04545 | 572 | Motif-1 | -15 | + | TGTACCTGTATA |
|  |  |  | Motif-17 | -561 | + | AGTGTAGCCATGCCA |
|  |  |  | Motif-4 | -118 | - | GCTGATTGGTCTTAA |
| P. infestans | PITG\_04546 | 502 | Motif-0 | -155 | + | ACATTTTGAATTTTTC |
|  |  |  | Motif-1 | -181 | + | TACATGTAA |
|  |  |  | Motif-4 | -237 | - | TTTGATTGGACAGAA |
| P. infestans | PITG\_04547 | 502 | Motif-0 | -363 | - | ACATTTTGAATTTTTC |
|  |  |  | Motif-1 | -329 | + | TACATGTAG |
|  |  |  | Motif-4 | -280 | + | TTTGATTGGACAGAA |
| P. infestans | PITG\_04549 | 1000 | Motif-4 | -323 | - | TGTGATTGGCTAATC |
| P. infestans | PITG\_04552 | 1000 | Motif-0 | -375 | + | GTAAATTCAGGAAGGA |
|  |  |  | Motif-1 | -71 | + | TACATGTAG |
|  |  |  | Motif-16 | -979 | - | CAACAGCAGCAGTAAC |
|  |  |  | Motif-2 | -886 | - | GTACTTCAACCTCC |
|  |  |  | Motif-8 | -478 | + | TAATTTTAAATATTT |
|  |  |  | Motif-9 | -673 | + | CTGTACCGGTA |
| P. infestans | PITG\_04558 | 1000 | Motif-0 | -299 | - | GCAAATTTGACAGTGA |
|  |  |  | Motif-8 | -370 | + | TTATTTTATTTTAAA |
| P. infestans | PITG\_04562 | 1000 | Motif-0 | -278 | - | TCATTCTTCAATTTT |
| P. infestans | PITG\_04566 | 1000 | Motif-2 | -94 | - | CACTTCAACGT |
| P. infestans | PITG\_04567 | 1000 | Motif-1 | -540 | + | AACATGTAC |
|  |  |  | Motif-3 | -982 | - | GACTTTGCTTTAGTAT |
| P. infestans | PITG\_04568 | 1000 | Motif-1 | -539 | - | ATAACATGTA |
|  |  |  | Motif-3 | -105 | + | GACTTTGCTTTAGTAT |
| P. infestans | PITG\_04569 | 957 | Motif-0 | -186 | - | GCTAAATCGGAAGTGC |
|  |  |  | Motif-2 | -813 | - | TTCACTTCAAGCCCTC |
| P. infestans | PITG\_04577 | 1000 | Motif-0 | -392 | + | CCATTTTCCCATCTCA |
|  |  |  | Motif-1 | -659 | - | AAAACAGGTACAAGA |
| P. infestans | PITG\_04578 | 1000 | Motif-0 | -633 | - | CCATTTTCCCATCTCA |
|  |  |  | Motif-1 | -364 | - | GTCTTGTACCTGTTT |
| P. infestans | PITG\_04581 | 1000 | Motif-0 | -43 | + | GCACTCATCAATTAGC |
|  |  |  | Motif-1 | -182 | + | AGTACCGGTATTGTA |
|  |  |  | Motif-2 | -245 | + | GCTTCAACATG |
|  |  |  | Motif-8 | -95 | - | TTTATTTATATTAAA |
|  |  |  | Motif-9 | -183 | + | CAGTACCGGTA |
| P. infestans | PITG\_04582 | 415 | Motif-0 | -36 | + | CATTCTTGAACTCGCC |
|  |  |  | Motif-2 | -190 | + | GCTTCAACATG |
| P. infestans | PITG\_04583 | 415 | Motif-0 | -394 | + | GCGAGTTCAAGAATGA |
|  |  |  | Motif-2 | -235 | - | CGCTTCAACAT |
| P. infestans | PITG\_04584 | 1000 | Motif-0 | -459 | + | CACTTCCCAATTTGCC |
|  |  |  | Motif-1 | -841 | + | TACATGTAT |
| P. infestans | PITG\_04588 | 1000 | Motif-18 | -426 | - | TGGTGTGGTGCACTCA |
|  |  |  | Motif-2 | -92 | + | GCTTCAACATG |
| P. infestans | PITG\_04589 | 1000 | Motif-0 | -327 | + | GCAAATAGCAAAGTGA |
|  |  |  | Motif-3 | -292 | - | ACTTAGTTTCACTAT |
| P. infestans | PITG\_04590 | 1000 | Motif-0 | -907 | + | CACTTTGCTATTTGCT |
|  |  |  | Motif-3 | -942 | + | ACTTAGTTTCACTAT |
| P. infestans | PITG\_04591 | 168 | Motif-0 | -44 | - | GCAAATCTGGGAATGG |
| P. infestans | PITG\_04592 | 168 | Motif-0 | -140 | + | GCAAATCTGGGAATGG |
| P. infestans | PITG\_04593 | 749 | Motif-1 | -744 | - | TACGTGTAC |
|  |  |  | Motif-2 | -149 | + | ACTTCAAAGTAGGCAA |
|  |  |  | Motif-4 | -371 | + | TGTCAGTGGCCAACA |
| P. infestans | PITG\_04598 | 1000 | Motif-1 | -138 | + | TACACGTACTGTGAT |
| P. infestans | PITG\_04599 | 1000 | Motif-0 | -64 | + | CCATTCCACAACTTGA |
|  |  |  | Motif-1 | -335 | + | TACATGTAG |
|  |  |  | Motif-3 | -711 | + | ACTTGCGTTCAGTAT |
|  |  |  | Motif-4 | -114 | + | TATCATTGGCTGTAA |
| P. infestans | PITG\_04603 | 1000 | Motif-2 | -265 | - | GCTGCAACTTG |
| P. infestans | PITG\_04608 | 370 | Motif-1 | -300 | + | AACATGTAC |
|  |  |  | Motif-3 | -132 | - | AACTTGGGCTTAGTAT |
| P. infestans | PITG\_04609 | 284 | Motif-3 | -188 | + | ACTCGAAGTTTGTAG |
|  |  |  | Motif-6 | -116 | - | TGCCCCACCTCCTCCT |
| P. infestans | PITG\_04610 | 284 | Motif-3 | -110 | + | TACAAACTTCGAGTGA |
|  |  |  | Motif-6 | -178 | + | ACCTCCTCCTCCTCCT |
| P. infestans | PITG\_04611 | 1000 | Motif-0 | -200 | + | GCAGTTCCCATTCCTC |
|  |  |  | Motif-1 | -414 | + | TACATGTAT |
| P. infestans | PITG\_04612 | 1000 | Motif-0 | -498 | + | GCACTTTACATTTAGC |
|  |  |  | Motif-1 | -926 | + | TACATGTAC |
| P. infestans | PITG\_04615 | 1000 | Motif-0 | -396 | - | GTAGATCGCAAACTGA |
|  |  |  | Motif-1 | -778 | + | TACATGTAA |
|  |  |  | Motif-8 | -541 | - | TTTATTTTTATAAAA |
| P. infestans | PITG\_04618 | 1000 | Motif-0 | -57 | + | CACTCCCGAGTTCGCC |
|  |  |  | Motif-1 | -353 | - | GGTACATGAAG |
|  |  |  | Motif-3 | -581 | + | ACTTGATTAAAGTAT |
|  |  |  | Motif-8 | -432 | + | TATTTTTTATTTACA |
| P. infestans | PITG\_04619 | 1000 | Motif-0 | -172 | + | CCATTCTGCATCTCCC |
| P. infestans | PITG\_04624 | 1000 | Motif-0 | -59 | - | GAGAATCGGATAGTGA |
|  |  |  | Motif-1 | -130 | - | TACAAGTACA |
| P. infestans | PITG\_04626 | 1000 | Motif-0 | -322 | - | GTAAAAGTGGAAATGA |
|  |  |  | Motif-8 | -106 | - | TATAATTTAATTATT |
| P. infestans | PITG\_04627 | 528 | Motif-0 | -82 | + | TTAGTCTGCAATCGGC |
|  |  |  | Motif-4 | -455 | - | GTCGATTGGATGAAA |
| P. infestans | PITG\_04628 | 528 | Motif-0 | -462 | - | TTAGTCTGCAATCGGC |
|  |  |  | Motif-4 | -88 | + | GTCGATTGGATGAAA |
| P. infestans | PITG\_04629 | 1000 | Motif-1 | -219 | + | TACATGTAG |
| P. infestans | PITG\_04633 | 1000 | Motif-0 | -21 | + | TCATTTCTCGTTTTTG |
|  |  |  | Motif-1 | -326 | - | TACATGAAC |
|  |  |  | Motif-7 | -741 | - | AATTATTAATA |
|  |  |  | Motif-8 | -121 | - | TTTTATTATTTTTTA |
| P. infestans | PITG\_04634 | 1000 | Motif-0 | -438 | - | TCAGTCTCGGTTCGTC |
|  |  |  | Motif-1 | -203 | - | AAATTGTACATGTCC |
|  |  |  | Motif-8 | -196 | - | TTTTTTTAAATTGTA |
| P. infestans | PITG\_04637 | 1000 | Motif-0 | -84 | + | CCAGTGTCAAATCTAC |
|  |  |  | Motif-4 | -848 | - | TATGATTTGTTGAAA |
| P. infestans | PITG\_04638 | 1000 | Motif-1 | -125 | + | TACATGTAA |
|  |  |  | Motif-4 | -429 | + | TATGATTTGTTGAAA |
|  |  |  | Motif-8 | -86 | + | TAATTTTTTTTTAAA |
| P. infestans | PITG\_04639 | 981 | Motif-1 | -369 | - | TACATGCATA |
|  |  |  | Motif-4 | -747 | - | TTTCATTGGTCACAA |
| P. infestans | PITG\_04640 | 981 | Motif-1 | -615 | - | AGCATGTATTAATAT |
|  |  |  | Motif-4 | -217 | + | GGTGACTGGTCAAAA |
| P. infestans | PITG\_04641 | 520 | Motif-1 | -201 | + | TACATGTAG |
|  |  |  | Motif-7 | -210 | + | CAATATTAATA |
| P. infestans | PITG\_04642 | 520 | Motif-1 | -192 | + | TACATGTAC |
|  |  |  | Motif-7 | -321 | - | CAATATTAATA |
| P. infestans | PITG\_04643 | 1000 | Motif-0 | -636 | + | TCACTCCGACTTTTGA |
|  |  |  | Motif-4 | -212 | + | TCTGATTGGTCAATT |
| P. infestans | PITG\_04644 | 1000 | Motif-1 | -213 | - | ACTGTACGAGTAGT |
|  |  |  | Motif-4 | -321 | + | TTTAAGCGAATGAAA |
| P. infestans | PITG\_04645 | 397 | Motif-17 | -368 | - | AATGTAGCCATGTTT |
| P. infestans | PITG\_04646 | 890 | Motif-1 | -158 | + | TACATGTAC |
|  |  |  | Motif-17 | -254 | - | ACTGTAGTCATCTCT |
| P. infestans | PITG\_04647 | 890 | Motif-1 | -124 | - | AACATGTATTCGAGT |
|  |  |  | Motif-17 | -651 | + | ACTGTAGTCATCTCT |
| P. infestans | PITG\_04648 | 1000 | Motif-0 | -85 | + | TCATTGTCGAACCTTC |
|  |  |  | Motif-6 | -18 | + | CCCTCCCCCTCCTCCG |
| P. infestans | PITG\_04650 | 1000 | Motif-0 | -860 | + | TCACTTTCGATACCTC |
|  |  |  | Motif-6 | -832 | - | ACCCCCTCCCCCAAAT |
| P. infestans | PITG\_04652 | 1000 | Motif-0 | -929 | + | TCACTTCGAATCGTGC |
|  |  |  | Motif-4 | -972 | - | TTCTATTGGCTGAAA |
| P. infestans | PITG\_04657 | 994 | Motif-1 | -784 | + | TACATGTAA |
| P. infestans | PITG\_04658 | 1000 | Motif-1 | -348 | + | TTTACCTGTACAATA |
|  |  |  | Motif-2 | -248 | - | TATTAAGATGAAGTGA |
|  |  |  | Motif-4 | -98 | - | TCGGATTGGTCTAAA |
| P. infestans | PITG\_04659 | 1000 | Motif-1 | -668 | + | TACATGTAC |
|  |  |  | Motif-2 | -574 | - | GATGTTGACGCG |
| P. infestans | PITG\_04661 | 1000 | Motif-0 | -56 | + | CATTTCCTCATTTGCG |
| P. infestans | PITG\_04663 | 1000 | Motif-0 | -105 | + | TCACATACCAATCTGC |
|  |  |  | Motif-4 | -178 | + | GTCCATTGGCTGAAA |
| P. infestans | PITG\_04664 | 414 | Motif-0 | -20 | - | GACAGTTCGAAAATGG |
|  |  |  | Motif-17 | -104 | + | GATGTAGCCATTTCT |
|  |  |  | Motif-4 | -60 | - | TTTGATTGGTGGAAA |
| P. infestans | PITG\_04665 | 331 | Motif-8 | -161 | + | TATGTTTTAGCATAA |
| P. infestans | PITG\_04666 | 331 | Motif-8 | -185 | - | TATGTTTTAGCATAA |
| P. infestans | PITG\_04671 | 1000 | Motif-0 | -907 | + | CATTCCCCAATTCACC |
|  |  |  | Motif-2 | -871 | - | CACCTTCAACCTG |
| P. infestans | PITG\_04673 | 1000 | Motif-0 | -112 | - | GTCAGATTGAAAGTGA |
|  |  |  | Motif-1 | -731 | + | TACATGTAG |
|  |  |  | Motif-18 | -293 | - | TATTGTGGTGTAAAAG |
|  |  |  | Motif-2 | -698 | - | GCGACTTCAATGTGAA |
|  |  |  | Motif-4 | -546 | - | GCCGATTGGATATAA |
| P. infestans | PITG\_04674 | 182 | Motif-3 | -57 | - | CACTTGCGTTTCGTAA |
|  |  |  | Motif-4 | -32 | - | CTTGCTTGGTTGAAA |
| P. infestans | PITG\_04675 | 182 | Motif-3 | -140 | - | CTTACGAAACGCAAGT |
|  |  |  | Motif-4 | -165 | + | CTTGCTTGGTTGAAA |
| P. infestans | PITG\_04676 | 77 | Motif-2 | -41 | + | CATGTTCAAGCG |
| P. infestans | PITG\_04680 | 1000 | Motif-2 | -48 | + | GACGTCGAAGTGGG |
| P. infestans | PITG\_04681 | 1000 | Motif-0 | -197 | - | TCACTCCTCCTTTGCA |
|  |  |  | Motif-4 | -132 | + | GTCAATTGGCTAAAA |
|  |  |  | Motif-8 | -422 | - | TATGATTTAGTTTAA |
| P. infestans | PITG\_04683 | 1000 | Motif-4 | -346 | + | CGTGATTCGCTAATA |
| P. infestans | PITG\_04685 | 482 | Motif-7 | -307 | - | TACTATTAATA |
| P. infestans | PITG\_04686 | 482 | Motif-7 | -183 | - | AAATATTAATA |
| P. infestans | PITG\_04687 | 1000 | Motif-0 | -247 | + | CATTTTTCAACTCGCC |
|  |  |  | Motif-1 | -998 | + | TGCATGTAGTTGTGT |
|  |  |  | Motif-8 | -411 | - | TTTGTATTATTAAAA |
| P. infestans | PITG\_04691 | 453 | Motif-1 | -195 | + | TACATGTAC |
| P. infestans | PITG\_04692 | 875 | Motif-8 | -296 | + | TTTTATTAAATTATA |
| P. infestans | PITG\_04694 | 1000 | Motif-0 | -76 | + | CCACATTTCAATTTGA |
| P. infestans | PITG\_04695 | 1000 | Motif-1 | -384 | + | TACATGAAC |
|  |  |  | Motif-17 | -78 | + | AATGTAGCCATCCTT |
|  |  |  | Motif-6 | -323 | - | GCCACGCGCCCCTCCT |
| P. infestans | PITG\_04697 | 1000 | Motif-1 | -229 | + | TACATGTAT |
| P. infestans | PITG\_04700 | 620 | Motif-4 | -367 | + | GTCAATTGGCTAAAA |
| P. infestans | PITG\_04701 | 620 | Motif-4 | -268 | - | GTCAATTGGCTAAAA |
| P. infestans | PITG\_04702 | 95 | Motif-3 | -41 | + | ACTTGGAGTTTGTAA |
| P. infestans | PITG\_04703 | 95 | Motif-3 | -68 | + | TACAAACTCCAAGTCA |
| P. infestans | PITG\_04704 | 342 | Motif-0 | -50 | - | TCCAATTCGAAAGTGA |
| P. infestans | PITG\_04705 | 342 | Motif-0 | -308 | + | TCCAATTCGAAAGTGA |
| P. infestans | PITG\_04707 | 173 | Motif-0 | -157 | + | GCAGGTTGAACAATGA |
|  |  |  | Motif-4 | -60 | + | TTTGATTGGTTGAAT |
| P. infestans | PITG\_04708 | 173 | Motif-0 | -32 | - | GCAGGTTGAACAATGA |
|  |  |  | Motif-4 | -128 | - | TTTGATTGGTTGAAT |
| P. infestans | PITG\_04712 | 1000 | Motif-1 | -500 | + | TACATGTAC |
| P. infestans | PITG\_04714 | 1000 | Motif-0 | -47 | - | GCAAATAGCGGAATGC |
|  |  |  | Motif-1 | -928 | + | TACATGTAT |
|  |  |  | Motif-3 | -369 | + | GCTTGTTTTTTGTAT |
|  |  |  | Motif-4 | -987 | - | CATCATTGGCTGAAA |
| P. infestans | PITG\_04715 | 1000 | Motif-1 | -118 | + | TACATGTAA |
|  |  |  | Motif-3 | -684 | - | AGCTTGTTTTTTGTAT |
|  |  |  | Motif-4 | -66 | + | CATCATTGGCTGAAA |
| P. infestans | PITG\_04717 | 1000 | Motif-1 | -87 | + | TGCATGTAC |
| P. infestans | PITG\_04720 | 144 | Motif-17 | -75 | - | AATGTAGCCATGTTG |
|  |  |  | Motif-2 | -80 | - | CATGTTGAATTG |
| P. infestans | PITG\_04721 | 144 | Motif-17 | -84 | + | AATGTAGCCATGTTG |
|  |  |  | Motif-2 | -76 | + | CATGTTGAATTG |
| P. infestans | PITG\_04722 | 561 | Motif-2 | -169 | - | AGAGTTGAAGTGGA |
|  |  |  | Motif-4 | -145 | + | TCTGATTGGTCGAAA |
| P. infestans | PITG\_04723 | 187 | Motif-0 | -104 | - | GCTAAAACGGAAGTGA |
| P. infestans | PITG\_04725 | 1000 | Motif-0 | -194 | - | GCATTTTGAGTTTTAC |
|  |  |  | Motif-1 | -255 | - | TACATGCAC |
| P. infestans | PITG\_04729 | 1000 | Motif-0 | -306 | - | TTAAATACGAAAGTGA |
|  |  |  | Motif-1 | -85 | - | GGTACCGGTACCGTA |
|  |  |  | Motif-9 | -83 | + | CGGTACCGGTA |
| P. infestans | PITG\_04731 | 1000 | Motif-0 | -587 | - | GCACTTTGCGTTCTTC |
| P. infestans | PITG\_04733 | 1000 | Motif-0 | -317 | + | GCATTGTCGAATTGAC |
|  |  |  | Motif-1 | -138 | + | TACATGTAC |
| P. infestans | PITG\_04736 | 1000 | Motif-1 | -869 | + | ATTACAGGTACTCTT |
|  |  |  | Motif-4 | -108 | + | TTTAATTGGTCGATT |
| P. infestans | PITG\_04738 | 1000 | Motif-0 | -31 | + | CATTCTCGAAGTTGCC |
|  |  |  | Motif-2 | -26 | - | CACGGCAACTTCGA |
|  |  |  | Motif-6 | -649 | + | GGCCCCCCCCCGCTAC |
|  |  |  | Motif-7 | -173 | + | TACTATTAATA |
| P. infestans | PITG\_04742 | 392 | Motif-4 | -108 | + | TTTAATTGGTCGATT |
| P. infestans | PITG\_04746 | 1000 | Motif-1 | -487 | + | GGTACCGGTACCGTA |
|  |  |  | Motif-7 | -395 | - | AAGTATTAATA |
|  |  |  | Motif-9 | -485 | - | CGGTACCGGTA |
| P. infestans | PITG\_04747 | 1000 | Motif-1 | -67 | - | GGTACCGGTACCGTA |
|  |  |  | Motif-9 | -65 | + | CGGTACCGGTA |
| P. infestans | PITG\_04752 | 404 | Motif-0 | -388 | + | GCAACTTCGAGAATGA |
| P. infestans | PITG\_04753 | 404 | Motif-0 | -31 | + | CATTCTCGAAGTTGCC |
| P. infestans | PITG\_04754 | 599 | Motif-0 | -30 | + | CATTCTGGAAGTTGCC |
| P. infestans | PITG\_04755 | 608 | Motif-0 | -30 | + | CATTCTGGAAGTTGCC |
| P. infestans | PITG\_04756 | 578 | Motif-0 | -30 | + | CATTCTGGAAGTTGCC |
| P. infestans | PITG\_04757 | 1000 | Motif-9 | -347 | - | ATGTACCGGTA |
| P. infestans | PITG\_04758 | 1000 | Motif-17 | -99 | + | AGTGTAGTCATGCGA |
|  |  |  | Motif-4 | -222 | - | CATGATTGGGCAATA |
| P. infestans | PITG\_04759 | 470 | Motif-4 | -99 | + | TTTAATTGGTCGATT |
| P. infestans | PITG\_04760 | 351 | Motif-4 | -108 | + | TTTAATTGGTCGATT |
| P. infestans | PITG\_04761 | 215 | Motif-4 | -108 | + | TTTTATTGGTCGATT |
| P. infestans | PITG\_04762 | 905 | Motif-4 | -499 | - | TTTAATTGGTCGATT |
| P. infestans | PITG\_04763 | 351 | Motif-4 | -108 | + | TTTAATTGGTCGATT |
| P. infestans | PITG\_04764 | 341 | Motif-4 | -108 | + | TTTTATTGGTCGATT |
| P. infestans | PITG\_04765 | 479 | Motif-4 | -108 | + | TTTAATTGGTCGATT |
| P. infestans | PITG\_04766 | 341 | Motif-4 | -108 | + | TTTAATTGGTCGATT |
| P. infestans | PITG\_04767 | 351 | Motif-4 | -108 | + | TTTAATTGGTCGATT |
| P. infestans | PITG\_04768 | 218 | Motif-4 | -108 | + | TTTTATTGGTCGATT |
| P. infestans | PITG\_04769 | 351 | Motif-4 | -108 | + | TTTTATTGGTCGATT |
| P. infestans | PITG\_04770 | 351 | Motif-4 | -108 | + | TTTAATTGGTCGATT |
| P. infestans | PITG\_04773 | 523 | Motif-0 | -60 | - | TCATTTTCAAACCTT |
|  |  |  | Motif-1 | -472 | + | GGTACCGGTACCGTA |
|  |  |  | Motif-9 | -470 | - | CGGTACCGGTA |
| P. infestans | PITG\_04774 | 523 | Motif-0 | -478 | + | TCATTTTCAAACCTTT |
|  |  |  | Motif-1 | -66 | - | GGTACCGGTACCGTA |
|  |  |  | Motif-9 | -64 | + | CGGTACCGGTA |
| P. infestans | PITG\_04778 | 1000 | Motif-4 | -108 | + | TTTAATTGGTCGATT |
| P. infestans | PITG\_04780 | 872 | Motif-0 | -31 | + | CATTCTCGAAGTTGCC |
|  |  |  | Motif-2 | -26 | - | CACGGCAACTTCGA |
|  |  |  | Motif-7 | -173 | + | TACTATTAATA |
| P. infestans | PITG\_04781 | 872 | Motif-0 | -31 | + | CATTCTCGAAGTTGCC |
|  |  |  | Motif-2 | -26 | - | CACGGCAACTTCGA |
|  |  |  | Motif-7 | -173 | + | TACTATTAATA |
| P. infestans | PITG\_04786 | 560 | Motif-0 | -477 | - | TCACTTCGCAATTTG |
|  |  |  | Motif-1 | -37 | + | TACACGTACTCATGA |
| P. infestans | PITG\_04787 | 560 | Motif-0 | -98 | - | TCAAATTGCGAAGTGA |
|  |  |  | Motif-1 | -535 | + | TGAGTACGTGTAAT |
| P. infestans | PITG\_04792 | 1000 | Motif-4 | -109 | + | CTTTATTGGTCGATT |
| P. infestans | PITG\_04794 | 1000 | Motif-3 | -640 | - | TTCTTAGTTTTTGTAT |
|  |  |  | Motif-8 | -636 | - | TATTTCTTAGTTTTT |
| P. infestans | PITG\_04795 | 328 | Motif-4 | -108 | + | TTTAATTGGTCGATT |
| P. infestans | PITG\_04796 | 328 | Motif-4 | -235 | - | TTTAATTGGTCGATT |
| P. infestans | PITG\_04798 | 1000 | Motif-4 | -235 | - | TTTAATTGGTCGATT |
| P. infestans | PITG\_04802 | 1000 | Motif-2 | -854 | - | GACGTTGCAGTT |
|  |  |  | Motif-6 | -414 | + | GGAACCCCCCCCTCCC |
| P. infestans | PITG\_04806 | 631 | Motif-4 | -108 | + | TTTAATTGGTCGATT |
| P. infestans | PITG\_04807 | 896 | Motif-4 | -108 | + | TTTAATTGGTCGATT |
| P. infestans | PITG\_04809 | 255 | Motif-4 | -108 | + | TTTAATTGGTCGATT |
| P. infestans | PITG\_04810 | 255 | Motif-4 | -162 | - | TTTAATTGGTCGATT |
| P. infestans | PITG\_04812 | 184 | Motif-4 | -108 | + | TTTAATTGGTCGATT |
| P. infestans | PITG\_04815 | 1000 | Motif-0 | -628 | + | CATTCTGGAAGTTGCC |
|  |  |  | Motif-7 | -782 | - | TAATATTAATA |
|  |  |  | Motif-9 | -966 | - | ATGTACCGGTA |
| P. infestans | PITG\_04819 | 1000 | Motif-3 | -824 | - | GTCTTGCCTTTTGTAT |
| P. infestans | PITG\_04820 | 1000 | Motif-0 | -637 | - | TTATTTCTAAGTTTG |
|  |  |  | Motif-1 | -622 | + | TACATGTAG |
|  |  |  | Motif-4 | -526 | - | GCTGATTGGCCGAAT |
|  |  |  | Motif-9 | -942 | - | GCGTACCGGTA |
| P. infestans | PITG\_04821 | 293 | Motif-2 | -142 | - | CAGCTTCAACTAG |
| P. infestans | PITG\_04822 | 349 | Motif-7 | -213 | - | AATTATTAATA |
|  |  |  | Motif-8 | -217 | + | TTTTTATTAATAATT |
| P. infestans | PITG\_04823 | 349 | Motif-7 | -147 | + | AATTATTAATA |
|  |  |  | Motif-8 | -147 | - | TTTTTATTAATAATT |
| P. infestans | PITG\_04824 | 1000 | Motif-2 | -243 | + | ACTTCAACGTG |
|  |  |  | Motif-3 | -325 | + | AACACAAAGCGGAAGT |
|  |  |  | Motif-7 | -807 | - | TACTATTAATA |
|  |  |  | Motif-8 | -814 | - | TATTAATATTTAAAA |
| P. infestans | PITG\_04827 | 1000 | Motif-0 | -29 | - | GCAAAACGAAGAATGA |
|  |  |  | Motif-1 | -369 | + | TGCATGTATTATAGT |
| P. infestans | PITG\_04828 | 1000 | Motif-0 | -425 | - | GCAAAACGAAAAGTGA |
|  |  |  | Motif-4 | -749 | + | GGGGATTGGGCAAAA |
| P. infestans | PITG\_04830 | 652 | Motif-0 | -167 | - | GCATTCTCAATATTGC |
|  |  |  | Motif-1 | -233 | + | TACATGTAA |
|  |  |  | Motif-2 | -606 | + | ACGTCAACTTG |
| P. infestans | PITG\_04831 | 652 | Motif-0 | -281 | - | TCATTCATAATTTCAC |
|  |  |  | Motif-1 | -427 | + | TACATGTAC |
|  |  |  | Motif-2 | -57 | - | ACGTCAACTTG |
| P. infestans | PITG\_04832 | 326 | Motif-2 | -113 | + | CAAGTTAAAGCTGC |
| P. infestans | PITG\_04833 | 326 | Motif-2 | -227 | - | CAAGTTAAAGCTGC |
| P. infestans | PITG\_04834 | 1000 | Motif-0 | -801 | + | CACTTTAGAATTGACG |
|  |  |  | Motif-2 | -513 | + | CAAGTGGAAGTG |
| P. infestans | PITG\_04835 | 1000 | Motif-2 | -780 | - | CTGCACGTTGCTGTC |
| P. infestans | PITG\_04837 | 188 | Motif-1 | -28 | + | TACATGTAC |
|  |  |  | Motif-8 | -105 | + | TTTTACTAATTATTT |
| P. infestans | PITG\_04838 | 188 | Motif-1 | -162 | + | TACATGTAC |
|  |  |  | Motif-8 | -98 | - | TTTTACTAATTATTT |
| P. infestans | PITG\_04841 | 555 | Motif-3 | -526 | - | CTTACAAGGCTCAAGT |
| P. infestans | PITG\_04842 | 417 | Motif-0 | -170 | - | CATTTCTAAACGTGCC |
|  |  |  | Motif-4 | -270 | + | GGTCACTGGCTGAAA |
| P. infestans | PITG\_04843 | 417 | Motif-0 | -263 | + | CATTTCTAAACGTGCC |
|  |  |  | Motif-4 | -162 | - | GGTCACTGGCTGAAA |
| P. infestans | PITG\_04844 | 214 | Motif-0 | -140 | + | GGAAATTTGCTAATGA |
|  |  |  | Motif-4 | -98 | - | TTTCATTGGTTAAGA |
| P. infestans | PITG\_04845 | 214 | Motif-0 | -90 | - | GGAAATTTGCTAATGA |
|  |  |  | Motif-4 | -79 | - | TGTGATTGGAGGAAA |
| P. infestans | PITG\_04846 | 520 | Motif-2 | -317 | - | CACTTCAACTT |
| P. infestans | PITG\_04847 | 962 | Motif-4 | -403 | + | GACGATTGGCTAAAA |
| P. infestans | PITG\_04850 | 871 | Motif-1 | -157 | + | TACATGTAA |
|  |  |  | Motif-2 | -772 | + | GCACTTCAACCGCC |
|  |  |  | Motif-3 | -751 | + | ACTTGAGTTCAGTAT |
| P. infestans | PITG\_04851 | 871 | Motif-1 | -604 | - | AACATGTAC |
|  |  |  | Motif-2 | -113 | - | GCACTTCAACCGCC |
|  |  |  | Motif-3 | -135 | - | GACTTGAGTTCAGTAT |
| P. infestans | PITG\_04853 | 307 | Motif-0 | -95 | - | TCGTTTTCCAATTTAT |
| P. infestans | PITG\_04854 | 379 | Motif-0 | -285 | + | TCACTCTGCGACTTGT |
| P. infestans | PITG\_04855 | 379 | Motif-0 | -110 | - | TCACTCTGCGACTTGT |
| P. infestans | PITG\_04856 | 950 | Motif-0 | -672 | + | TCATTTTTATTTCTAC |
|  |  |  | Motif-1 | -122 | + | AATTCATGTACAGCTG |
| P. infestans | PITG\_04857 | 113 | Motif-4 | -71 | + | AACGATTGGCTTAAA |
| P. infestans | PITG\_04858 | 113 | Motif-4 | -57 | - | AACGATTGGCTTAAA |
| P. infestans | PITG\_04859 | 572 | Motif-0 | -463 | + | GCAAAATTGGAAATGC |
|  |  |  | Motif-1 | -313 | - | GCTACCGGTACTGTA |
|  |  |  | Motif-8 | -389 | - | TTTTATTTAGCAAAA |
|  |  |  | Motif-9 | -311 | + | CAGTACCGGTA |
| P. infestans | PITG\_04860 | 572 | Motif-0 | -124 | + | CATTTCCAATTTTGCT |
|  |  |  | Motif-1 | -265 | + | ACTGTACCAGTAGT |
|  |  |  | Motif-8 | -198 | + | TTTTATTTAGCAAAA |
|  |  |  | Motif-9 | -272 | - | CAGTACCGGTA |
| P. infestans | PITG\_04861 | 1000 | Motif-4 | -445 | - | CCTTATTGGGTGATA |
| P. infestans | PITG\_04862 | 1000 | Motif-0 | -30 | - | TCAAATTCCCAACTGA |
|  |  |  | Motif-2 | -193 | + | CAAGTTGAAGTA |
|  |  |  | Motif-3 | -73 | - | CATACTAGATCCAAGT |
| P. infestans | PITG\_04863 | 251 | Motif-0 | -20 | + | TCACTCTGCAAGTGTC |
|  |  |  | Motif-4 | -69 | - | TGCGATTGGTTAAAA |
| P. infestans | PITG\_04864 | 251 | Motif-0 | -247 | - | TCACTCTGCAAGTGTC |
|  |  |  | Motif-4 | -197 | + | TGCGATTGGTTAAAA |
| P. infestans | PITG\_04865 | 274 | Motif-17 | -81 | + | AATGTAGCCATATCT |
| P. infestans | PITG\_04866 | 274 | Motif-17 | -208 | - | AATGTAGCCATATCT |
| P. infestans | PITG\_04867 | 761 | Motif-1 | -481 | + | TACATGTAC |
|  |  |  | Motif-2 | -398 | - | AAGGTTGAAGTG |
| P. infestans | PITG\_04868 | 1000 | Motif-0 | -61 | - | GAAAAATCCTAAATGA |
|  |  |  | Motif-4 | -190 | - | GTCCATTGGACAAAA |
| P. infestans | PITG\_04869 | 354 | Motif-7 | -270 | + | TAATATTAATA |
|  |  |  | Motif-8 | -270 | - | TATGTATTAATATTA |
| P. infestans | PITG\_04870 | 354 | Motif-7 | -95 | - | TAATATTAATA |
|  |  |  | Motif-8 | -99 | + | TATGTATTAATATTA |
| P. infestans | PITG\_04871 | 111 | Motif-8 | -43 | + | TTTTATTAAACTAAA |
| P. infestans | PITG\_04872 | 111 | Motif-8 | -78 | - | TTTTTTTTTATTAAA |
| P. infestans | PITG\_04873 | 339 | Motif-17 | -223 | + | AATGTACCCATGTTT |
| P. infestans | PITG\_04875 | 122 | Motif-0 | -46 | - | GCAAAAAAAAGAGTGA |
|  |  |  | Motif-2 | -120 | - | AACTTCAACTT |
| P. infestans | PITG\_04878 | 84 | Motif-8 | -73 | - | TTTTTTTAAATAATT |
| P. infestans | PITG\_04879 | 84 | Motif-8 | -23 | - | TTTAATTATTTAAAA |
| P. infestans | PITG\_04880 | 516 | Motif-0 | -344 | - | CCATTTCTAATTATGC |
|  |  |  | Motif-17 | -336 | - | TGTGTAGCCATTTCT |
| P. infestans | PITG\_04881 | 516 | Motif-0 | -188 | + | CCATTTCTAATTATGC |
|  |  |  | Motif-17 | -195 | + | TGTGTAGCCATTTCT |
| P. infestans | PITG\_04882 | 156 | Motif-0 | -30 | - | GACAATTGCGAAGTGG |
|  |  |  | Motif-2 | -94 | - | CACTTCAACTT |
|  |  |  | Motif-4 | -73 | - | GCTGATTGGCGGAAA |
| P. infestans | PITG\_04883 | 1000 | Motif-15 | -189 | + | AACTTCAACCACGC |
|  |  |  | Motif-2 | -189 | - | GTGGTTGAAGTT |
| P. infestans | PITG\_04884 | 1000 | Motif-0 | -130 | - | GACAATTGAGAAATGC |
| P. infestans | PITG\_04887 | 1000 | Motif-4 | -181 | - | CGCGATTGGTTAAAA |
|  |  |  | Motif-9 | -236 | - | ATTTACCGGTA |
| P. infestans | PITG\_04888 | 321 | Motif-0 | -283 | + | GGAAATCAAAGAGTGA |
| P. infestans | PITG\_04889 | 321 | Motif-0 | -54 | - | GGAAATCAAAGAGTGA |
| P. infestans | PITG\_04893 | 318 | Motif-0 | -170 | - | TCAGTTTTCAGTTCTC |
|  |  |  | Motif-8 | -59 | - | TATTTTTTTATATTT |
| P. infestans | PITG\_04894 | 318 | Motif-0 | -164 | + | TCAGTTTTCAGTTCTC |
|  |  |  | Motif-8 | -272 | + | TTTTTTTATATTTAT |
| P. infestans | PITG\_04895 | 1000 | Motif-1 | -993 | - | TACAAGTACT |
| P. infestans | PITG\_04896 | 1000 | Motif-0 | -643 | + | TCACTCTCAGATTTAT |
|  |  |  | Motif-6 | -516 | + | GCCCCCCGCCCCGTCC |
| P. infestans | PITG\_04899 | 1000 | Motif-16 | -859 | - | GCGCAGCTGCAGCAAC |
|  |  |  | Motif-6 | -923 | + | CCCCCCCCCCCCCGGT |
| P. infestans | PITG\_04900 | 1000 | Motif-1 | -660 | - | TACATGAAC |
|  |  |  | Motif-3 | -451 | - | GATTTGCGATTTGTTT |
| P. infestans | PITG\_04902 | 1000 | Motif-3 | -529 | + | CACTTGGTGTTCGTAG |
| P. infestans | PITG\_04903 | 1000 | Motif-0 | -692 | + | CTACTTCCAAATTTCA |
|  |  |  | Motif-1 | -262 | + | TACATGTAT |
|  |  |  | Motif-2 | -497 | + | GCTTCAACACG |
|  |  |  | Motif-4 | -140 | - | CATGATTGGACGAAA |
|  |  |  | Motif-7 | -256 | - | CATTATTAATA |
| P. infestans | PITG\_04904 | 1000 | Motif-0 | -642 | - | CTACTTCCAAATTTCA |
|  |  |  | Motif-1 | -806 | + | TACATGTAG |
|  |  |  | Motif-2 | -832 | + | CGTGTTGAAGCG |
| P. infestans | PITG\_04905 | 275 | Motif-1 | -118 | + | TACATGTAG |
| P. infestans | PITG\_04906 | 375 | Motif-4 | -167 | - | TCAGATTGGACAAAA |
| P. infestans | PITG\_04907 | 1000 | Motif-0 | -511 | - | ACATTTCAAAATTCTC |
|  |  |  | Motif-1 | -141 | - | CATACCGGTACAGTA |
|  |  |  | Motif-3 | -656 | + | GACTAGAAATTTGTAT |
|  |  |  | Motif-8 | -196 | + | TATTTTTTTGTTAAA |
|  |  |  | Motif-9 | -139 | + | CTGTACCGGTA |
| P. infestans | PITG\_04908 | 1000 | Motif-1 | -16 | + | TACATGTAC |
|  |  |  | Motif-9 | -233 | - | ATGTACCGGTA |
| P. infestans | PITG\_04909 | 1000 | Motif-0 | -834 | - | ACACTGCAGAATCTGC |
|  |  |  | Motif-2 | -270 | + | GAACTTGAAGTT |
|  |  |  | Motif-4 | -951 | - | AGTGATTGGCTAAAT |
| P. infestans | PITG\_04910 | 1000 | Motif-0 | -247 | + | GCAGACTGCAACTTGC |
|  |  |  | Motif-2 | -242 | + | CTGCAACTTGCAGCT |
|  |  |  | Motif-4 | -143 | + | AGTGATTGGCTAAAT |
| P. infestans | PITG\_04912 | 474 | Motif-2 | -121 | - | CATATTGAAGTG |
| P. infestans | PITG\_04915 | 162 | Motif-1 | -133 | - | CATTCATGTACTG |
|  |  |  | Motif-8 | -44 | + | TATTTTTAAATAGCT |
| P. infestans | PITG\_04917 | 1000 | Motif-1 | -106 | - | GATACGTGTAG |
|  |  |  | Motif-2 | -80 | - | CACTTCAACAT |
| P. infestans | PITG\_04921 | 1000 | Motif-1 | -365 | - | TGCATGTAC |
|  |  |  | Motif-8 | -149 | + | TAATTTTAAATTACT |
| P. infestans | PITG\_04922 | 1000 | Motif-0 | -35 | + | TCACTTCTCAGTCTAC |
|  |  |  | Motif-9 | -153 | - | AGGTACCGGTA |
| P. infestans | PITG\_04923 | 1000 | Motif-1 | -275 | - | AACATGTAC |
|  |  |  | Motif-6 | -667 | + | ACCCCCCCCCCCCAGC |
|  |  |  | Motif-7 | -403 | - | TATTATTAATA |
|  |  |  | Motif-9 | -280 | - | ATGTACCGGTA |
| P. infestans | PITG\_04924 | 459 | Motif-1 | -374 | + | TACATGTAC |
| P. infestans | PITG\_04925 | 1000 | Motif-0 | -46 | + | CACTCCTCATTTTACC |
|  |  |  | Motif-1 | -223 | + | TACATGTAC |
|  |  |  | Motif-2 | -283 | + | ACTGCAACGTG |
|  |  |  | Motif-7 | -809 | - | TACTACTAATA |
|  |  |  | Motif-8 | -613 | + | TATTAATAAATATCA |
| P. infestans | PITG\_04926 | 1000 | Motif-0 | -64 | + | CATTTTGCAACTTGCG |
|  |  |  | Motif-1 | -424 | + | TACATGTAC |
| P. infestans | PITG\_04927 | 1000 | Motif-0 | -40 | + | CACTCTGCATTTTGCT |
| P. infestans | PITG\_04928 | 1000 | Motif-0 | -967 | - | TCACTCTAGAACTTTT |
|  |  |  | Motif-2 | -176 | + | ACTTCAACCTCTCTAA |
|  |  |  | Motif-6 | -96 | + | CCACCTCCCCCGCCAT |
| P. infestans | PITG\_04930 | 1000 | Motif-0 | -559 | + | GAAGAATTAAAAATGA |
|  |  |  | Motif-1 | -300 | - | ACAACATGTA |
| P. infestans | PITG\_04931 | 1000 | Motif-7 | -660 | - | TATTACTAATA |
|  |  |  | Motif-8 | -534 | - | TACTTTTAAATAATA |
|  |  |  | Motif-9 | -688 | - | AAGTACCGGTA |
| P. infestans | PITG\_04934 | 1000 | Motif-1 | -896 | + | TACATGTAC |
|  |  |  | Motif-7 | -949 | - | TATTATTAATA |
| P. infestans | PITG\_04937 | 170 | Motif-17 | -47 | - | AATGTAGCCATTTCA |
| P. infestans | PITG\_04938 | 170 | Motif-17 | -138 | + | AATGTAGCCATTTCA |
| P. infestans | PITG\_04939 | 1000 | Motif-1 | -379 | + | AGTGTACATGGATT |
| P. infestans | PITG\_04940 | 1000 | Motif-1 | -664 | - | AGTGTACATGGATT |
| P. infestans | PITG\_04941 | 1000 | Motif-1 | -128 | + | TTAATACAAGTAAT |
|  |  |  | Motif-8 | -137 | + | TACTATTTATTAATA |
| P. infestans | PITG\_04942 | 1000 | Motif-0 | -561 | + | CACTCTTCCATTCCCT |
|  |  |  | Motif-2 | -423 | + | CAAGTGGAAGTG |
|  |  |  | Motif-3 | -756 | + | ACTTGCAGTTTGTAA |
|  |  |  | Motif-6 | -164 | - | CGAGCCCCCCACTCAC |
|  |  |  | Motif-8 | -854 | - | TAAATTTAAGTTTAA |
| P. infestans | PITG\_04947 | 1000 | Motif-0 | -64 | - | GAAAAACCGGGAATGG |
|  |  |  | Motif-1 | -326 | + | TACATGTGCA |
|  |  |  | Motif-2 | -161 | - | GATCTTGAAGTG |
|  |  |  | Motif-4 | -579 | - | GCTTATTGGGCAGTA |
| P. infestans | PITG\_04948 | 540 | Motif-0 | -55 | - | AAAAAATGGGGAATGA |
|  |  |  | Motif-1 | -269 | + | TACGTGTAC |
| P. infestans | PITG\_04949 | 1000 | Motif-0 | -36 | + | CCACTTCTCGAATTAC |
|  |  |  | Motif-1 | -130 | + | TACATGTAC |
|  |  |  | Motif-7 | -494 | - | TATTATTAATA |
|  |  |  | Motif-9 | -136 | - | ATGTACCGGTA |
| P. infestans | PITG\_04952 | 1000 | Motif-0 | -583 | - | GCAAAAGGAGAAATGA |
|  |  |  | Motif-1 | -690 | + | TACATGTAG |
|  |  |  | Motif-2 | -790 | + | ACTTCAACTTG |
|  |  |  | Motif-4 | -828 | + | TCTGATTGGATGAAA |
| P. infestans | PITG\_04953 | 440 | Motif-0 | -382 | - | TCAGTGATCCTTTTGC |
|  |  |  | Motif-1 | -203 | + | TGCATGTAC |
| P. infestans | PITG\_04954 | 1000 | Motif-1 | -51 | + | AATACCGGTACATTA |
|  |  |  | Motif-9 | -49 | - | ATGTACCGGTA |
| P. infestans | PITG\_04956 | 1000 | Motif-0 | -36 | - | GGGAATTTCTAAATGA |
|  |  |  | Motif-2 | -98 | + | ACTTCAACATC |
|  |  |  | Motif-8 | -146 | + | TTTTATTATATAAAT |
| P. infestans | PITG\_04957 | 506 | Motif-2 | -74 | + | TCGCTTCAAGATCA |
|  |  |  | Motif-4 | -160 | + | CGTGATTGGTAGAAT |
| P. infestans | PITG\_04958 | 506 | Motif-2 | -446 | - | TCGCTTCAAGATCA |
|  |  |  | Motif-4 | -361 | - | CGTGATTGGTAGAAT |
| P. infestans | PITG\_04959 | 1000 | Motif-0 | -236 | + | GAAAATTGAAAAATTG |
|  |  |  | Motif-1 | -103 | - | AGAATACAAGTAGT |
| P. infestans | PITG\_04960 | 1000 | Motif-0 | -325 | - | GGAAAATATGAAATGA |
|  |  |  | Motif-1 | -688 | - | AGAATACGCGTACT |
|  |  |  | Motif-3 | -341 | - | AACTTGCTATTTGTAT |
|  |  |  | Motif-4 | -563 | + | TCTGATTGGTCAAAA |
| P. infestans | PITG\_04961 | 1000 | Motif-0 | -648 | + | GCAAATTCGAAAGTGA |
|  |  |  | Motif-2 | -581 | - | ACAACTTCAACAGCAA |
| P. infestans | PITG\_04963 | 1000 | Motif-0 | -428 | + | GCAAAAAGAGGAATGA |
|  |  |  | Motif-2 | -466 | - | AACTCCAACTT |
| P. infestans | PITG\_04965 | 866 | Motif-0 | -453 | - | TCACTATCGTTTCTTC |
| P. infestans | PITG\_04967 | 765 | Motif-1 | -90 | + | TACATGTGTA |
| P. infestans | PITG\_04969 | 1000 | Motif-0 | -58 | - | GCAGGATGGAGAATGA |
| P. infestans | PITG\_04970 | 1000 | Motif-2 | -601 | + | AGGCAAGTCGAAGCCA |
|  |  |  | Motif-7 | -612 | + | CAATATTAATA |
| P. infestans | PITG\_04971 | 1000 | Motif-0 | -757 | + | CCACTTGCCTATCTGA |
|  |  |  | Motif-16 | -328 | - | CAGCAGTAGCAGCAAC |
| P. infestans | PITG\_04972 | 1000 | Motif-0 | -736 | - | CCACTTGCCTATCTGA |
|  |  |  | Motif-2 | -264 | + | ACTTCAAGATG |
| P. infestans | PITG\_04974 | 205 | Motif-1 | -169 | - | CCTACCTGTACATTG |
| P. infestans | PITG\_04975 | 1000 | Motif-2 | -244 | + | ACAAGTTCAACTTGTC |
| P. infestans | PITG\_04976 | 1000 | Motif-2 | -125 | - | GTTGTTGAAGTG |
|  |  |  | Motif-8 | -241 | + | TTTTAATAATTAACA |
| P. infestans | PITG\_04977 | 1000 | Motif-0 | -123 | + | CATTCCCCAATTTCCT |
|  |  |  | Motif-1 | -559 | + | TACATGTAG |
|  |  |  | Motif-2 | -706 | - | CACTGCAAACTGCA |
|  |  |  | Motif-3 | -247 | - | TCTTGGTCTCAGTAT |
|  |  |  | Motif-9 | -572 | + | GCGTACCGGTA |
| P. infestans | PITG\_04978 | 402 | Motif-0 | -55 | - | GCGAAAACGGAAATGA |
| P. infestans | PITG\_04981 | 1000 | Motif-0 | -35 | - | GGTAAAGGAGAAATGA |
|  |  |  | Motif-1 | -255 | + | TGCATGTAC |
|  |  |  | Motif-2 | -180 | + | GCTTCAACCTCCGAAA |
| P. infestans | PITG\_04982 | 1000 | Motif-0 | -35 | - | GGTAAAGGAGAAATGA |
|  |  |  | Motif-1 | -295 | + | TACATGTTCAGTTTT |
|  |  |  | Motif-2 | -231 | - | GAGGTTGAAGTT |
|  |  |  | Motif-9 | -357 | - | CAGTACCGGTA |
| P. infestans | PITG\_04983 | 1000 | Motif-1 | -898 | - | TACATGAAC |
|  |  |  | Motif-2 | -96 | - | ATCTTCAACTT |
|  |  |  | Motif-7 | -711 | - | TATTATTAATA |
| P. infestans | PITG\_04988 | 1000 | Motif-8 | -372 | + | TTAGTTTAAATATAA |
| P. infestans | PITG\_04989 | 1000 | Motif-0 | -51 | + | CATTTTTCAATTCCTC |
|  |  |  | Motif-2 | -158 | - | GCGCTTCAACTGCA |
|  |  |  | Motif-8 | -638 | - | TTTTTTTTAATTTAA |
| P. infestans | PITG\_04990 | 445 | Motif-1 | -238 | + | TCAAGCTACATCTAC |
|  |  |  | Motif-3 | -353 | + | TAGAAAAACCCAATGA |
| P. infestans | PITG\_04991 | 578 | Motif-0 | -33 | - | TTAAAATGGGGAATGG |
| P. infestans | PITG\_04992 | 578 | Motif-0 | -561 | + | TTAAAATGGGGAATGG |
| P. infestans | PITG\_04994 | 1000 | Motif-0 | -30 | + | TCAGTTTTCGTTTTCT |
|  |  |  | Motif-1 | -312 | + | TACATATAC |
|  |  |  | Motif-7 | -112 | + | TACTATTAATA |
| P. infestans | PITG\_04998 | 1000 | Motif-0 | -45 | + | CACTTTCGATCTCACC |
|  |  |  | Motif-3 | -146 | + | ACTCAAGGTTTGTAT |
| P. infestans | PITG\_04999 | 1000 | Motif-0 | -86 | - | GCAAAACTGGAAATGG |
|  |  |  | Motif-1 | -472 | + | TGCATGTAGCTCTTA |
|  |  |  | Motif-8 | -601 | - | TTTTAATAAACTAAA |
| P. infestans | PITG\_05000 | 374 | Motif-1 | -314 | + | TACGTGTAC |
|  |  |  | Motif-6 | -26 | + | ACCCCCCCCCCTCTCT |
| P. infestans | PITG\_05001 | 374 | Motif-1 | -69 | - | TACGTGTAC |
|  |  |  | Motif-6 | -362 | - | AGACCCCCCCCCCTCT |
| P. infestans | PITG\_05002 | 299 | Motif-1 | -64 | + | TACATGTAC |
|  |  |  | Motif-2 | -23 | + | CAAGTTCAAGTG |
| P. infestans | PITG\_05003 | 1000 | Motif-1 | -199 | + | TACATGTAC |
|  |  |  | Motif-2 | -399 | + | CCATTTCAAGTTCC |
|  |  |  | Motif-4 | -797 | - | ATTGATTGGTCTAAA |
| P. infestans | PITG\_05007 | 306 | Motif-1 | -151 | - | AATACAAGTACCGTA |
|  |  |  | Motif-3 | -84 | + | ACTTAGTTTTTGTAG |
| P. infestans | PITG\_05010 | 1000 | Motif-3 | -950 | + | ACTTGCGAATTGTAT |
| P. infestans | PITG\_05011 | 1000 | Motif-3 | -195 | - | AACTTGCGAATTGTAT |
| P. infestans | PITG\_05012 | 1000 | Motif-1 | -153 | + | TACATGTAT |
| P. infestans | PITG\_05017 | 1000 | Motif-0 | -429 | - | CATTTGTTATTTTGCC |
|  |  |  | Motif-1 | -492 | - | AGTACCGGTACAGTCA |
|  |  |  | Motif-4 | -607 | - | TCTAATTGACTAAAA |
|  |  |  | Motif-9 | -486 | - | AAGTACCGGTA |
| P. infestans | PITG\_05028 | 1000 | Motif-0 | -861 | + | ACACTCACCAATTTTT |
|  |  |  | Motif-1 | -587 | - | GGTACATATAG |
|  |  |  | Motif-4 | -659 | - | TTTCATTGGGCAAAT |
| P. infestans | PITG\_05032 | 1000 | Motif-1 | -787 | + | TGCATGTAGAAGGTC |
| P. infestans | PITG\_05043 | 1000 | Motif-4 | -252 | - | CATGATTGGTCAAAA |
| P. infestans | PITG\_05045 | 1000 | Motif-4 | -499 | + | TTTCATTGGCCACTA |
| P. infestans | PITG\_05049 | 1000 | Motif-16 | -679 | - | CAACAGCAGTAGCGAC |
|  |  |  | Motif-2 | -656 | + | ACCTTCAACTT |
|  |  |  | Motif-4 | -740 | - | TGTGATCGGCCAAAT |
| P. infestans | PITG\_05056 | 1000 | Motif-1 | -93 | + | TACCTGTATTATTAG |
|  |  |  | Motif-2 | -767 | - | TCTACTTCAAGTAGCA |
| P. infestans | PITG\_05058 | 579 | Motif-0 | -27 | - | GCGGAATCGAAAATGA |
| P. infestans | PITG\_05065 | 1000 | Motif-1 | -73 | + | TGTACATGAATTGTT |
|  |  |  | Motif-15 | -126 | + | AACTTCAACCACTC |
|  |  |  | Motif-2 | -126 | - | GTGGTTGAAGTT |
| P. infestans | PITG\_05066 | 1000 | Motif-4 | -444 | + | TTTGATTGGACAAAA |
| P. infestans | PITG\_05067 | 1000 | Motif-0 | -48 | - | GCCAATTGAAGAGTGG |
|  |  |  | Motif-1 | -330 | + | TACATGTAC |
|  |  |  | Motif-8 | -926 | + | TTTTTTTAAATAGCA |
| P. infestans | PITG\_05075 | 1000 | Motif-0 | -942 | - | TCACTTATAAAATTG |
|  |  |  | Motif-2 | -465 | + | GCAATTGAAGTGGC |
|  |  |  | Motif-4 | -385 | - | AGTGATTGGCTGAAA |
| P. infestans | PITG\_05079 | 1000 | Motif-0 | -781 | + | GCACTTCCCAGCTCG |
|  |  |  | Motif-17 | -276 | + | TCTGTAGCCATGTGG |
|  |  |  | Motif-4 | -255 | - | CCTGATTGGCTCAAA |
| P. infestans | PITG\_05080 | 485 | Motif-0 | -20 | + | CACTTGGCAACTTACT |
| P. infestans | PITG\_05081 | 1000 | Motif-0 | -153 | + | ACATTCGCCAACTTCC |
|  |  |  | Motif-6 | -744 | - | TCAGCCCCCCCCCCCT |
|  |  |  | Motif-8 | -647 | - | TATTTTTTAATTTTT |
| P. infestans | PITG\_05082 | 1000 | Motif-1 | -446 | + | TACAAGTACT |
|  |  |  | Motif-9 | -460 | - | CGGTACCGGTA |
| P. infestans | PITG\_05083 | 977 | Motif-0 | -41 | - | GATAAATGAACAGTGA |
| P. infestans | PITG\_05084 | 686 | Motif-4 | -56 | - | TTTGCTTGGACAAAA |
| P. infestans | PITG\_05091 | 662 | Motif-0 | -40 | + | TCAGTCTTAAATTCAA |
|  |  |  | Motif-1 | -344 | - | TACGTGTAC |
| P. infestans | PITG\_05092 | 1000 | Motif-0 | -39 | - | GCTGATTGCAAAGTGG |
| P. infestans | PITG\_05095 | 1000 | Motif-0 | -41 | + | CATTTTCACTTTTGCT |
|  |  |  | Motif-6 | -178 | - | TGAGCCCCCCCCCTCC |
| P. infestans | PITG\_05096 | 1000 | Motif-0 | -41 | + | CATTTTCACTTTTGCT |
|  |  |  | Motif-6 | -180 | - | AGCCCCCCCCCCTCCT |
| P. infestans | PITG\_05097 | 1000 | Motif-0 | -35 | + | CATTCTCAAATTTGCT |
|  |  |  | Motif-1 | -691 | + | TACATGTAA |
|  |  |  | Motif-15 | -569 | + | AACTTCAACCAGTC |
|  |  |  | Motif-2 | -569 | - | CTGGTTGAAGTT |
| P. infestans | PITG\_05099 | 1000 | Motif-1 | -606 | + | TACATGTAA |
| P. infestans | PITG\_05101 | 1000 | Motif-0 | -351 | - | GTAAAAAGAAGAATGA |
|  |  |  | Motif-4 | -580 | - | TGTCATTGGCACAAA |
| P. infestans | PITG\_05102 | 1000 | Motif-1 | -651 | + | TACAAGTACT |
|  |  |  | Motif-2 | -390 | - | CAAGTTGAATTT |
|  |  |  | Motif-4 | -891 | - | GATCATTGGTCAAAA |
| P. infestans | PITG\_05103 | 475 | Motif-0 | -66 | - | GCCAAAGAGAAAATGA |
|  |  |  | Motif-1 | -217 | - | GTAAACTTCATGTAT |
| P. infestans | PITG\_05104 | 564 | Motif-0 | -235 | - | GCGAGAGGGAGAATGA |
|  |  |  | Motif-1 | -555 | - | TACATGAAC |
| P. infestans | PITG\_05106 | 1000 | Motif-3 | -888 | - | CACTTGAATATAGTAT |
| P. infestans | PITG\_05110 | 1000 | Motif-3 | -176 | + | CATACAGAACCAAAGT |
| P. infestans | PITG\_05111 | 1000 | Motif-1 | -236 | - | TATATATGTACTG |
|  |  |  | Motif-17 | -469 | + | TGTGTAGCCATATTG |
|  |  |  | Motif-2 | -439 | + | GCTTCAACGTG |
|  |  |  | Motif-3 | -120 | - | TTACAAAAGATAAGAT |
|  |  |  | Motif-8 | -716 | - | TTTTTTTAAATTTTA |
| P. infestans | PITG\_05112 | 1000 | Motif-1 | -510 | + | TACATGTAG |
|  |  |  | Motif-17 | -652 | - | TGTGTAGCCATATTG |
|  |  |  | Motif-2 | -677 | - | AGCTTCAACGT |
|  |  |  | Motif-8 | -405 | + | TTTTTTTAAATTTTA |
| P. infestans | PITG\_05113 | 273 | Motif-2 | -21 | + | ACCCAAGTCGAAGTAA |
|  |  |  | Motif-7 | -104 | + | TATTACTAATA |
| P. infestans | PITG\_05114 | 273 | Motif-2 | -268 | - | ACCCAAGTCGAAGTAA |
|  |  |  | Motif-7 | -180 | - | TATTACTAATA |
| P. infestans | PITG\_05115 | 630 | Motif-1 | -225 | + | TACATGTAT |
|  |  |  | Motif-2 | -287 | - | GATGTTGATGTG |
|  |  |  | Motif-8 | -347 | - | TATTTATTAACTAAA |
| P. infestans | PITG\_05118 | 1000 | Motif-0 | -78 | - | GAAAGTTGCGAACTGA |
|  |  |  | Motif-1 | -276 | - | TACATATAC |
| P. infestans | PITG\_05122 | 1000 | Motif-8 | -694 | - | TTTGTTTTATTAAAT |
| P. infestans | PITG\_05125 | 1000 | Motif-0 | -224 | + | TCATTTGCGAATCGGA |
|  |  |  | Motif-1 | -675 | - | TACATATAC |
|  |  |  | Motif-3 | -374 | + | TACTCAAATCAAGTCA |
| P. infestans | PITG\_05126 | 1000 | Motif-0 | -18 | + | TCATTACCCAACTCTA |
|  |  |  | Motif-3 | -559 | + | TACAAATTTCAAATGA |
| P. infestans | PITG\_05127 | 792 | Motif-0 | -56 | - | GAAAGTCGGAGACTGA |
|  |  |  | Motif-1 | -207 | + | GTCATTTTCATGTAC |
|  |  |  | Motif-18 | -199 | - | TTATGTGGCGTACATG |
|  |  |  | Motif-2 | -746 | - | GTGCATGCTGAAGTA |
|  |  |  | Motif-3 | -101 | + | ACTTGGTCTCAGTAT |
|  |  |  | Motif-4 | -218 | + | TCCGATTGGCTGTCA |
| P. infestans | PITG\_05128 | 1000 | Motif-2 | -368 | - | CTCTTCAACTT |
| P. infestans | PITG\_05129 | 1000 | Motif-0 | -252 | + | TCATTAATAAACTTAC |
|  |  |  | Motif-2 | -52 | + | GCTTCAACATG |
| P. infestans | PITG\_05131 | 1000 | Motif-0 | -528 | + | GCAAATACCGAAGTGA |
| P. infestans | PITG\_05133 | 1000 | Motif-1 | -984 | + | TACATGTAG |
|  |  |  | Motif-3 | -783 | - | ACTCGTAGTTAGTAT |
| P. infestans | PITG\_05134 | 1000 | Motif-2 | -164 | + | GCTTCAACGTG |
| P. infestans | PITG\_05138 | 1000 | Motif-2 | -16 | + | TGAGTTGAAGTG |
| P. infestans | PITG\_05140 | 1000 | Motif-0 | -51 | + | CCATTTCTTCATCTGC |
| P. infestans | PITG\_05141 | 1000 | Motif-2 | -191 | - | CCAGTTGAAGTG |
| P. infestans | PITG\_05143 | 1000 | Motif-8 | -419 | + | TTATTTTTAGTAAAT |
| P. infestans | PITG\_05146 | 1000 | Motif-1 | -244 | + | TACATGTAC |
|  |  |  | Motif-4 | -317 | + | TTGCGACCAATCTAA |
| P. infestans | PITG\_05147 | 1000 | Motif-0 | -329 | - | TCATTCAAGAACTTTC |
|  |  |  | Motif-3 | -176 | + | ACTTGGTTAAAGTAT |
|  |  |  | Motif-8 | -566 | - | TTTGTCTAATTTATA |
| P. infestans | PITG\_05148 | 1000 | Motif-1 | -95 | + | TACATGTAC |
|  |  |  | Motif-4 | -37 | + | GCTGATTGGTCAAAA |
|  |  |  | Motif-8 | -499 | - | TTTTTTTAATTTGCA |
| P. infestans | PITG\_05149 | 1000 | Motif-0 | -138 | - | GCATTTCCGCTTCTTC |
|  |  |  | Motif-2 | -103 | + | GCTCCAACATCAGCAA |
| P. infestans | PITG\_05150 | 985 | Motif-1 | -726 | + | TGCATGTATTTTTGC |
| P. infestans | PITG\_05151 | 1000 | Motif-0 | -90 | + | CACTATTCAACTTGTC |
|  |  |  | Motif-3 | -153 | + | TACAAACTCCAAGTCG |
|  |  |  | Motif-4 | -411 | - | TAATAGCCAATTGGA |
| P. infestans | PITG\_05154 | 1000 | Motif-0 | -332 | - | TCACTCTTCATTTCG |
|  |  |  | Motif-1 | -127 | - | AAAATCTACAAGTAC |
|  |  |  | Motif-2 | -390 | - | TGATTTCAACGTGC |
| P. infestans | PITG\_05155 | 1000 | Motif-1 | -103 | - | AACATGTAC |
| P. infestans | PITG\_05156 | 1000 | Motif-0 | -92 | - | GCAGATTGCAGAATGA |
|  |  |  | Motif-8 | -263 | + | TTTTATTAAATATGA |
| P. infestans | PITG\_05161 | 1000 | Motif-4 | -693 | - | TCTGATTGGCAAAAA |
| P. infestans | PITG\_05169 | 1000 | Motif-3 | -357 | - | AAGAAAAGCCCAGTCA |
| P. infestans | PITG\_05170 | 1000 | Motif-4 | -296 | + | TCTCATTCGTCAAAA |
| P. infestans | PITG\_05171 | 1000 | Motif-4 | -333 | - | TTTGATTGGTAGAAA |
| P. infestans | PITG\_05172 | 1000 | Motif-0 | -222 | - | CACTGTCCAATGTGTC |
|  |  |  | Motif-1 | -528 | + | AACATGTATTTATTA |
| P. infestans | PITG\_05173 | 1000 | Motif-0 | -675 | + | ATTTTATTTTCAGATT |
|  |  |  | Motif-1 | -165 | + | TACATGTAT |
|  |  |  | Motif-3 | -814 | - | CACTTTGTTTTAGTAT |
| P. infestans | PITG\_05174 | 307 | Motif-2 | -236 | + | CAAGTTGACGTG |
|  |  |  | Motif-4 | -71 | - | TCGGATTGGATGATA |
| P. infestans | PITG\_05175 | 307 | Motif-2 | -82 | + | ACGTCAACTTG |
|  |  |  | Motif-4 | -218 | + | TTTAATTGGTAGAAA |
| P. infestans | PITG\_05176 | 783 | Motif-1 | -652 | + | AGTACATCTAC |
| P. infestans | PITG\_05177 | 1000 | Motif-1 | -125 | + | TACATGTAT |
|  |  |  | Motif-8 | -551 | - | TACTTTTTAACTAAA |
| P. infestans | PITG\_05180 | 1000 | Motif-17 | -27 | + | AATATACCCATTTGG |
| P. infestans | PITG\_05185 | 1000 | Motif-6 | -29 | + | GGACCCCCCCCCTCAA |
| P. infestans | PITG\_05186 | 589 | Motif-2 | -411 | - | ACTCCAACGCG |
|  |  |  | Motif-4 | -94 | + | ATTCATTGGACAAAA |
| P. infestans | PITG\_05187 | 589 | Motif-2 | -189 | + | ACTCCAACGCG |
|  |  |  | Motif-4 | -510 | - | ATTCATTGGACAAAA |
| P. infestans | PITG\_05188 | 533 | Motif-0 | -104 | - | GGATATTGGAGACTGA |
|  |  |  | Motif-18 | -141 | - | TGGTGTAGCTTACACT |
| P. infestans | PITG\_05190 | 1000 | Motif-4 | -291 | - | GTGGATTGGATGAAA |
|  |  |  | Motif-7 | -372 | + | TATTATTAATA |
| P. infestans | PITG\_05191 | 1000 | Motif-2 | -106 | + | CGTGTTGAAGTG |
|  |  |  | Motif-8 | -71 | + | TTTGATTAAATAATT |
| P. infestans | PITG\_05193 | 196 | Motif-1 | -54 | + | TACTTGTACA |
|  |  |  | Motif-3 | -177 | + | TACAAAAAGCAAGTTA |
|  |  |  | Motif-4 | -85 | + | TTGGATTGGATGAAT |
| P. infestans | PITG\_05194 | 196 | Motif-1 | -150 | + | TACAAGTACA |
|  |  |  | Motif-3 | -33 | + | ACTTGCTTTTTGTAG |
|  |  |  | Motif-4 | -126 | - | TTGGATTGGATGAAT |
| P. infestans | PITG\_05196 | 1000 | Motif-0 | -30 | - | GCGAGATTAGAAGTGA |
|  |  |  | Motif-8 | -345 | + | TTTTAATAAGTAAAT |
| P. infestans | PITG\_05197 | 1000 | Motif-4 | -369 | + | TGTCATTGGTCGGAA |
|  |  |  | Motif-6 | -273 | + | GCCGCCCCCCCCCAGC |
| P. infestans | PITG\_05198 | 1000 | Motif-0 | -42 | - | GAGAATTGAAGAATGT |
|  |  |  | Motif-1 | -406 | + | TACATGTAA |
|  |  |  | Motif-2 | -186 | + | CCAGTTGAAGCGGA |
|  |  |  | Motif-8 | -443 | + | TTTTTTTTAATAATA |
| P. infestans | PITG\_05200 | 866 | Motif-1 | -543 | + | TACATGTAC |
|  |  |  | Motif-2 | -212 | + | TTCGTTGAAGTG |
| P. infestans | PITG\_05201 | 1000 | Motif-0 | -973 | + | TCATTTACCGTATTCC |
| P. infestans | PITG\_05202 | 1000 | Motif-0 | -330 | - | GGAGATTCAGAAGTGA |
| P. infestans | PITG\_05206 | 1000 | Motif-0 | -167 | - | ACACTTCCCAAATTGA |
|  |  |  | Motif-3 | -122 | - | ACTTGATTTCACTAT |
| P. infestans | PITG\_05208 | 1000 | Motif-1 | -915 | + | TACATGTAA |
|  |  |  | Motif-2 | -456 | - | ACTTCAAGTTG |
| P. infestans | PITG\_05210 | 1000 | Motif-1 | -913 | - | TTTACCTGTATTATA |
|  |  |  | Motif-3 | -163 | + | TACAAACGTCAAGTCA |
| P. infestans | PITG\_05211 | 806 | Motif-0 | -74 | + | CATTTTCAAACTTACC |
|  |  |  | Motif-1 | -207 | - | GGTACATCTAT |
| P. infestans | PITG\_05212 | 1000 | Motif-0 | -282 | - | GCATTCCTCTATTTCC |
|  |  |  | Motif-4 | -178 | - | CCTGATTGGGCGATA |
| P. infestans | PITG\_05216 | 1000 | Motif-1 | -511 | + | TACATGCAC |
|  |  |  | Motif-3 | -108 | - | GACTTGGTTTAAGTAT |
| P. infestans | PITG\_05217 | 1000 | Motif-0 | -34 | + | CATTGTTCAACTTGCC |
| P. infestans | PITG\_05221 | 1000 | Motif-4 | -538 | + | TCTGATTGGTCAAAA |
| P. infestans | PITG\_05222 | 252 | Motif-1 | -134 | + | TACAAGTACA |
| P. infestans | PITG\_05223 | 496 | Motif-1 | -435 | + | TACATGTAA |
| P. infestans | PITG\_05224 | 331 | Motif-1 | -189 | + | TACCTGTACTGTAGT |
| P. infestans | PITG\_05225 | 1000 | Motif-0 | -39 | - | GCAGATTGCAAAATGC |
|  |  |  | Motif-4 | -194 | + | TCAGATTGGCCAAAA |
|  |  |  | Motif-6 | -877 | + | CCCCCCCCCCCCCGAT |
| P. infestans | PITG\_05226 | 1000 | Motif-4 | -198 | - | GTTTATTGGGTAATT |
| P. infestans | PITG\_05227 | 1000 | Motif-4 | -207 | + | TATGATTGGCAGAAA |
| P. infestans | PITG\_05228 | 1000 | Motif-7 | -171 | + | TAATATTAATA |
| P. infestans | PITG\_05231 | 1000 | Motif-2 | -820 | + | ACTCCAACACG |
| P. infestans | PITG\_05235 | 970 | Motif-0 | -601 | - | TCAGTTCACATTCTAC |
|  |  |  | Motif-1 | -245 | + | TACTTGTACT |
| P. infestans | PITG\_05236 | 1000 | Motif-0 | -740 | + | TCATTCTCCCAGTTGT |
|  |  |  | Motif-1 | -605 | + | TGCATGTATGCATGT |
|  |  |  | Motif-4 | -990 | + | TCCGATTAGCTAAAA |
| P. infestans | PITG\_05238 | 1000 | Motif-6 | -62 | + | AGAGCCCCCCCCCTAC |
| P. infestans | PITG\_05239 | 1000 | Motif-1 | -127 | + | TACGTGTAC |
|  |  |  | Motif-4 | -658 | + | TTTAATTGGGTAATA |
| P. infestans | PITG\_05240 | 1000 | Motif-1 | -981 | - | TACGTGTAC |
|  |  |  | Motif-4 | -456 | - | TTTAATTGGGTAATA |
| P. infestans | PITG\_05243 | 621 | Motif-0 | -599 | - | TCATTCCTTAACTTG |
|  |  |  | Motif-1 | -334 | + | TACATGTAT |
| P. infestans | PITG\_05244 | 621 | Motif-0 | -36 | + | CATTCCTTAACTTGCC |
|  |  |  | Motif-1 | -295 | + | TACATGTAT |
| P. infestans | PITG\_05245 | 1000 | Motif-0 | -960 | + | CACTCCGCAATGCACC |
|  |  |  | Motif-1 | -21 | + | TACATGTAC |
|  |  |  | Motif-3 | -614 | + | ACTTAGCTTTTGTAT |
|  |  |  | Motif-9 | -752 | - | CGGTACCGGTA |
| P. infestans | PITG\_05246 | 1000 | Motif-4 | -722 | - | TCTGATTTGTTAAAT |
|  |  |  | Motif-7 | -893 | + | TAATATTAATA |
| P. infestans | PITG\_05247 | 617 | Motif-0 | -40 | - | GCTGAATGAAAAATGA |
|  |  |  | Motif-1 | -300 | - | AGCATGTATTATAAT |
| P. infestans | PITG\_05248 | 816 | Motif-1 | -167 | + | TACATGTAC |
| P. infestans | PITG\_05249 | 816 | Motif-1 | -657 | + | TACATGTAC |
| P. infestans | PITG\_05250 | 284 | Motif-18 | -61 | - | TGGTGTGGTGTACGTG |
| P. infestans | PITG\_05251 | 1000 | Motif-0 | -529 | + | CGACTTTTCAATATTC |
|  |  |  | Motif-2 | -425 | + | ACTGCAACTCG |
|  |  |  | Motif-3 | -784 | - | AACTAACTGCCAGTCA |
|  |  |  | Motif-4 | -588 | - | GACCATTGGTTAAAT |
| P. infestans | PITG\_05253 | 1000 | Motif-0 | -47 | - | GAGAAACTGAGAGTGA |
|  |  |  | Motif-1 | -99 | + | TACATGTAC |
| P. infestans | PITG\_05254 | 1000 | Motif-0 | -47 | - | GAGAAACTGAGAGTGA |
|  |  |  | Motif-1 | -99 | + | TACATGTAC |
| P. infestans | PITG\_05255 | 1000 | Motif-0 | -203 | + | TCACTCTCAATTTGCT |
| P. infestans | PITG\_05258 | 1000 | Motif-0 | -57 | - | GTAGGTTGGGGAATGA |
|  |  |  | Motif-1 | -144 | + | AGTACCGGTACAACA |
|  |  |  | Motif-4 | -948 | - | TCTGCTTGGTCGAAA |
|  |  |  | Motif-8 | -807 | + | TTTTTCTAATTTTGA |
|  |  |  | Motif-9 | -145 | + | CAGTACCGGTA |
| P. infestans | PITG\_05260 | 1000 | Motif-1 | -215 | + | AAAACATATACAGGA |
|  |  |  | Motif-4 | -190 | - | TTTGACTGGCTAATA |
|  |  |  | Motif-7 | -116 | + | AAATATTAATA |
| P. infestans | PITG\_05262 | 678 | Motif-0 | -634 | + | GCAAAATGCTGAATGA |
|  |  |  | Motif-2 | -616 | + | GAAGTTGCAGTT |
|  |  |  | Motif-4 | -568 | + | TGTGATTGGCTGAAC |
| P. infestans | PITG\_05263 | 678 | Motif-0 | -60 | - | GCAAAATGCTGAATGA |
|  |  |  | Motif-2 | -73 | + | ACTGCAACTTC |
|  |  |  | Motif-4 | -125 | - | TGTGATTGGCTGAAC |
| P. infestans | PITG\_05264 | 1000 | Motif-0 | -39 | - | GCGAAATGCGAATTGA |
|  |  |  | Motif-2 | -259 | + | GACATTGAAGCTGA |
|  |  |  | Motif-8 | -726 | - | TAAAATTAAGTTTAA |
| P. infestans | PITG\_05265 | 1000 | Motif-1 | -433 | + | TACATGTAC |
|  |  |  | Motif-17 | -549 | - | GATTTAGCCATTTCA |
| P. infestans | PITG\_05267 | 1000 | Motif-0 | -419 | + | TCACTCGACAATTTAT |
|  |  |  | Motif-1 | -661 | + | TACATGTAC |
| P. infestans | PITG\_05271 | 1000 | Motif-0 | -63 | - | CCAAATTGGTGAATGA |
|  |  |  | Motif-1 | -424 | + | TACATGTGTA |
|  |  |  | Motif-7 | -305 | - | CAATATTAATA |
| P. infestans | PITG\_05272 | 1000 | Motif-0 | -55 | + | CATTCCGCAATTTGCG |
|  |  |  | Motif-17 | -518 | - | TCTGTAGCCATCCGG |
|  |  |  | Motif-2 | -141 | + | ACTTCAACTTC |
| P. infestans | PITG\_05273 | 1000 | Motif-4 | -472 | + | GGTGATTGGCTCAAA |
|  |  |  | Motif-8 | -560 | - | TTTTTTTTACTAAAA |
| P. infestans | PITG\_05274 | 1000 | Motif-1 | -136 | + | TACATGTAT |
|  |  |  | Motif-18 | -79 | - | TGATGTGGTGCACATT |
|  |  |  | Motif-2 | -70 | - | TAAGTTGATGTG |
| P. infestans | PITG\_05277 | 1000 | Motif-0 | -227 | + | CATTTTTGAATTCGCT |
| P. infestans | PITG\_05279 | 1000 | Motif-0 | -67 | - | GCCAAAACAGGAGTGA |
| P. infestans | PITG\_05281 | 555 | Motif-16 | -513 | - | GCACCGCCGCAGCAGC |
|  |  |  | Motif-17 | -27 | + | AGTGTAGCCATCTTG |
| P. infestans | PITG\_05282 | 555 | Motif-16 | -58 | + | GCACCGCCGCAGCAGC |
|  |  |  | Motif-17 | -543 | - | AGTGTAGCCATCTTG |
| P. infestans | PITG\_05285 | 1000 | Motif-0 | -322 | + | TCATTCATCGACTTTC |
|  |  |  | Motif-1 | -483 | + | TACATGTAT |
|  |  |  | Motif-7 | -635 | + | AAGTATTAATA |
|  |  |  | Motif-8 | -668 | - | TATTATTTAATAAAA |
| P. infestans | PITG\_05286 | 1000 | Motif-1 | -113 | - | ATTACATTTACAGCTA |
|  |  |  | Motif-6 | -356 | - | CCAGCTCCCCCCTCCT |
| P. infestans | PITG\_05287 | 1000 | Motif-3 | -278 | + | TCTTACGTTTTGTAT |
| P. infestans | PITG\_05288 | 1000 | Motif-4 | -948 | + | AATGATTGGTCAAAA |
| P. infestans | PITG\_05289 | 175 | Motif-4 | -102 | - | TGCGATTGGTCTAAA |
| P. infestans | PITG\_05290 | 175 | Motif-4 | -88 | + | TGCGATTGGTCTAAA |
| P. infestans | PITG\_05291 | 1000 | Motif-4 | -131 | - | TTTGATTGGTTAAGA |
| P. infestans | PITG\_05292 | 436 | Motif-2 | -418 | - | CCACTTCATCTTC |
|  |  |  | Motif-4 | -372 | + | TCTGATTGGTTGAAA |
| P. infestans | PITG\_05293 | 436 | Motif-2 | -31 | + | CCACTTCATCTTC |
|  |  |  | Motif-4 | -79 | - | TCTGATTGGTTGAAA |
| P. infestans | PITG\_05294 | 1000 | Motif-0 | -65 | + | TCATTGTGCGATTAAC |
|  |  |  | Motif-1 | -492 | + | TACATGTAC |
|  |  |  | Motif-9 | -489 | + | ATGTACCGGTA |
| P. infestans | PITG\_05295 | 1000 | Motif-0 | -698 | + | CATTCTGCAACTTCCC |
|  |  |  | Motif-2 | -623 | - | AGCCTGGTTGAAGCCG |
| P. infestans | PITG\_05296 | 401 | Motif-0 | -63 | + | CCACTCCTGCTTCTCC |
| P. infestans | PITG\_05300 | 128 | Motif-17 | -50 | + | GGTGTAGCCATATTT |
| P. infestans | PITG\_05301 | 128 | Motif-17 | -93 | - | GGTGTAGCCATATTT |
| P. infestans | PITG\_05302 | 218 | Motif-0 | -186 | - | GCAGTTTTACTTTCTC |
|  |  |  | Motif-8 | -38 | + | TATTATTTTTTAATT |
| P. infestans | PITG\_05303 | 218 | Motif-0 | -48 | + | GCAGTTTTACTTTCTC |
|  |  |  | Motif-8 | -195 | - | TATTATTTTTTAATT |
| P. infestans | PITG\_05304 | 288 | Motif-8 | -256 | + | TTTTTTTTAATAAAT |
| P. infestans | PITG\_05305 | 1000 | Motif-17 | -534 | - | AATGTAGCCATGTAA |
|  |  |  | Motif-6 | -70 | - | ACACCCACCCACCCAC |
| P. infestans | PITG\_05311 | 1000 | Motif-4 | -70 | - | TATGATTGGCTAAAA |
| P. infestans | PITG\_05312 | 1000 | Motif-0 | -37 | + | TCAGACTTCAATTTTT |
|  |  |  | Motif-4 | -882 | + | CTTCATTGGCTAATA |
| P. infestans | PITG\_05313 | 1000 | Motif-0 | -33 | + | CATTCTCCATTTGACC |
|  |  |  | Motif-2 | -137 | + | CGCGTTGAAGTG |
| P. infestans | PITG\_05314 | 554 | Motif-4 | -284 | - | CATGATTGGCTAAAA |
| P. infestans | PITG\_05315 | 554 | Motif-4 | -285 | + | CATGATTGGCTAAAA |
| P. infestans | PITG\_05317 | 1000 | Motif-3 | -971 | - | ACTTATAAATTGTAT |
| P. infestans | PITG\_05319 | 1000 | Motif-3 | -64 | + | TACAAATTTCTAGTCA |
| P. infestans | PITG\_05320 | 1000 | Motif-0 | -879 | - | ACACTTCAAAATCTGC |
|  |  |  | Motif-2 | -110 | + | CAAATTGAAGTG |
|  |  |  | Motif-4 | -768 | + | TTTGATTTGACAAAA |
| P. infestans | PITG\_05323 | 1000 | Motif-2 | -51 | + | CTCTTCAACTT |
|  |  |  | Motif-4 | -35 | - | TGTGATTGGCTAAGT |
|  |  |  | Motif-8 | -173 | + | TTTTTTTATTTTAAT |
| P. infestans | PITG\_05324 | 438 | Motif-0 | -184 | - | GCAAGTGTTAAAATGA |
|  |  |  | Motif-4 | -78 | - | TGTGATTGGTCTAAA |
| P. infestans | PITG\_05325 | 1000 | Motif-1 | -545 | + | TACAAGTACA |
|  |  |  | Motif-2 | -122 | - | GGTCGAGTTGCTGTC |
| P. infestans | PITG\_05326 | 1000 | Motif-0 | -544 | + | TGACTTTACTACTTGC |
|  |  |  | Motif-1 | -470 | + | TACATGTAT |
|  |  |  | Motif-4 | -59 | - | TACGATTGGTAAAAA |
|  |  |  | Motif-8 | -617 | - | TTTTATTAAGTAATT |
| P. infestans | PITG\_05327 | 1000 | Motif-0 | -24 | - | GCCAGATCAAAACTGA |
|  |  |  | Motif-4 | -70 | + | TCTGATTGGTCATTT |
| P. infestans | PITG\_05328 | 1000 | Motif-2 | -841 | + | CCACTTCACCCAG |
|  |  |  | Motif-3 | -244 | + | AACAAATATCCAGTCA |
|  |  |  | Motif-4 | -510 | - | TCCGATTGGCTTAGA |
|  |  |  | Motif-6 | -58 | + | ACTCCTCCCCCCACCG |
|  |  |  | Motif-9 | -464 | - | GAGTACCGGTA |
| P. infestans | PITG\_05329 | 522 | Motif-17 | -92 | + | TGTGTAGCCATGTGA |
| P. infestans | PITG\_05330 | 812 | Motif-0 | -432 | + | CATTGCCAAATTGGCC |
|  |  |  | Motif-2 | -213 | - | AGCTTCAACGT |
| P. infestans | PITG\_05331 | 812 | Motif-0 | -100 | + | CATTTGCGAACTTGCT |
|  |  |  | Motif-2 | -610 | - | GACGTTGAAGCT |
| P. infestans | PITG\_05332 | 1000 | Motif-1 | -333 | + | TACATGTAT |
| P. infestans | PITG\_05334 | 1000 | Motif-16 | -617 | + | CAACCGTAGCAGCGCC |
|  |  |  | Motif-2 | -671 | - | TCTTCAACATCAGCAA |
| P. infestans | PITG\_05335 | 1000 | Motif-0 | -588 | - | GTAAATTTGGTACTGA |
| P. infestans | PITG\_05338 | 1000 | Motif-1 | -84 | - | TGTGCCGGTACCGGTA |
|  |  |  | Motif-2 | -853 | + | ACTTCAACACG |
|  |  |  | Motif-9 | -84 | - | CGGTACCGGTA |
| P. infestans | PITG\_05339 | 1000 | Motif-1 | -639 | - | TACATGTGCT |
|  |  |  | Motif-7 | -570 | - | TATTATTAATA |
| P. infestans | PITG\_05340 | 1000 | Motif-8 | -140 | - | TTTTATTTATCATAT |
| P. infestans | PITG\_05345 | 693 | Motif-1 | -376 | + | TACATGTAC |
|  |  |  | Motif-2 | -559 | - | CCACATCAACTCGA |
| P. infestans | PITG\_05346 | 693 | Motif-1 | -35 | + | TACATGTAG |
|  |  |  | Motif-2 | -147 | - | CTTCGAGTTGATGTG |
| P. infestans | PITG\_05347 | 1000 | Motif-0 | -39 | - | GCCAACTCGGAAATGA |
|  |  |  | Motif-1 | -308 | + | TACATGTTCAGTATT |
|  |  |  | Motif-2 | -96 | - | TCTCAAGTTGAAGGCG |
|  |  |  | Motif-8 | -880 | - | TTCTTTTTAATAATA |
| P. infestans | PITG\_05348 | 1000 | Motif-0 | -934 | - | CTATTTCTAAATTTG |
|  |  |  | Motif-1 | -877 | + | TACATGTAG |
| P. infestans | PITG\_05354 | 536 | Motif-0 | -96 | + | GCACACTTCAACTTAC |
|  |  |  | Motif-1 | -317 | + | TACATGTAC |
|  |  |  | Motif-2 | -92 | + | ACTTCAACTTACACAT |
|  |  |  | Motif-4 | -79 | + | CATGATTGGCCATAA |
| P. infestans | PITG\_05355 | 536 | Motif-0 | -456 | - | GCACACTTCAACTTAC |
|  |  |  | Motif-1 | -227 | + | TACATGTAA |
|  |  |  | Motif-2 | -454 | - | CACTTCAACTT |
|  |  |  | Motif-4 | -34 | - | TCTGATTGGCTAACC |
| P. infestans | PITG\_05356 | 462 | Motif-0 | -27 | + | CATTTTGCATTTTGCC |
|  |  |  | Motif-9 | -105 | - | GTGTACCGGTA |
| P. infestans | PITG\_05357 | 462 | Motif-0 | -449 | - | TCATTTTGCATTTTG |
|  |  |  | Motif-9 | -368 | + | GTGTACCGGTA |
| P. infestans | PITG\_05361 | 1000 | Motif-0 | -56 | + | TCACTTTGCATTTAGT |
|  |  |  | Motif-1 | -94 | + | AACATGTAGTGATAA |
|  |  |  | Motif-2 | -25 | - | AAAGTTGAAGTC |
| P. infestans | PITG\_05362 | 1000 | Motif-1 | -422 | + | TACATGTAC |
|  |  |  | Motif-2 | -722 | + | ACTGCAACTTG |
| P. infestans | PITG\_05363 | 753 | Motif-1 | -388 | + | AACATGTAC |
|  |  |  | Motif-3 | -697 | + | GCTTGGTTTTTGTAT |
|  |  |  | Motif-9 | -469 | + | CCGTACCGGTA |
| P. infestans | PITG\_05364 | 753 | Motif-1 | -229 | + | AACATGTATCTGATC |
|  |  |  | Motif-3 | -71 | - | AGCTTGGTTTTTGTAT |
|  |  |  | Motif-9 | -295 | - | CCGTACCGGTA |
| P. infestans | PITG\_05366 | 198 | Motif-1 | -102 | + | TACATGCATT |
| P. infestans | PITG\_05367 | 198 | Motif-1 | -104 | + | TGCATGTAC |
| P. infestans | PITG\_05369 | 240 | Motif-16 | -76 | + | CAACAGCAGCAGCAGT |
| P. infestans | PITG\_05370 | 1000 | Motif-0 | -135 | - | ATGAATTCGAGAGTGA |
|  |  |  | Motif-1 | -569 | + | TACATGTAT |
|  |  |  | Motif-2 | -730 | + | AACTCCAACTT |
| P. infestans | PITG\_05372 | 1000 | Motif-0 | -952 | - | TCACTTCTCAAGATGC |
|  |  |  | Motif-4 | -857 | + | TTTGATTGGCTAAAA |
| P. infestans | PITG\_05373 | 1000 | Motif-0 | -46 | + | CCACTTATAAATTGAC |
|  |  |  | Motif-7 | -520 | - | AAATATTAATA |
| P. infestans | PITG\_05374 | 787 | Motif-1 | -88 | + | TACATATAC |
|  |  |  | Motif-2 | -182 | + | CCTTCAACTTCTGTGA |
| P. infestans | PITG\_05375 | 278 | Motif-2 | -118 | - | ACTGCAACACG |
|  |  |  | Motif-4 | -16 | - | CTCGACTGGTTGAAA |
| P. infestans | PITG\_05376 | 298 | Motif-17 | -90 | - | ATTGTAGCCATTTTA |
| P. infestans | PITG\_05379 | 1000 | Motif-0 | -419 | - | GTAGATCAGAGAGTGA |
|  |  |  | Motif-3 | -318 | - | ACTCGGCTATTGTAT |
| P. infestans | PITG\_05380 | 1000 | Motif-0 | -58 | - | GGGAATCAGGAAATGA |
|  |  |  | Motif-1 | -236 | + | TACATATAC |
| P. infestans | PITG\_05381 | 278 | Motif-0 | -50 | + | TCATTTTTCAAGTGGA |
|  |  |  | Motif-1 | -269 | - | ATTACAGGTAT |
|  |  |  | Motif-2 | -90 | - | GCTGCAACATG |
|  |  |  | Motif-8 | -198 | - | TTTTTTTATTTTTTT |
| P. infestans | PITG\_05382 | 471 | Motif-1 | -316 | - | TACGTGTAC |
|  |  |  | Motif-16 | -40 | - | CCGCAGCCGCAGCGAC |
|  |  |  | Motif-2 | -245 | + | GACATTGAAGCTGC |
| P. infestans | PITG\_05385 | 1000 | Motif-0 | -49 | + | CATTCTGCAATTTACG |
|  |  |  | Motif-1 | -283 | + | TACATGTAT |
|  |  |  | Motif-3 | -348 | + | TTTTGCTGTTTGTAT |
|  |  |  | Motif-4 | -685 | - | TGTGATTTGTCAAAT |
| P. infestans | PITG\_05386 | 1000 | Motif-0 | -58 | + | TCATTCGTCATCCTCC |
|  |  |  | Motif-1 | -518 | + | AACATGTAC |
|  |  |  | Motif-7 | -670 | - | TACTACTAATA |
|  |  |  | Motif-9 | -811 | - | ATTTACCGGTA |
| P. infestans | PITG\_05388 | 282 | Motif-0 | -142 | + | TCACATTTGAAGTTGC |
| P. infestans | PITG\_05389 | 282 | Motif-0 | -156 | - | TCACATTTGAAGTTGC |
| P. infestans | PITG\_05391 | 1000 | Motif-4 | -79 | + | CGTGACTGGCAGAAA |
|  |  |  | Motif-8 | -697 | - | TTTGATTTATTTTTT |
| P. infestans | PITG\_05392 | 629 | Motif-0 | -90 | - | GGAAGTTGTGGAATGA |
| P. infestans | PITG\_05394 | 490 | Motif-0 | -69 | + | CCATTCTAAAATCTTC |
| P. infestans | PITG\_05395 | 837 | Motif-0 | -295 | - | CCATTTCTCAAATCG |
|  |  |  | Motif-1 | -631 | - | TCAACATGTAAAGCTC |
|  |  |  | Motif-8 | -757 | - | TATTTTTAAACTTTT |
| P. infestans | PITG\_05396 | 837 | Motif-0 | -96 | + | TTATTTTTAAACTTT |
|  |  |  | Motif-1 | -222 | + | TCAACATGTAAAGCTC |
|  |  |  | Motif-8 | -95 | + | TATTTTTAAACTTTT |
| P. infestans | PITG\_05398 | 1000 | Motif-0 | -138 | - | ACATTTTTAAATTTTA |
| P. infestans | PITG\_05400 | 1000 | Motif-0 | -45 | + | TCATTCCTCGATTTCG |
|  |  |  | Motif-2 | -423 | + | CTGCAAGTTGCTGTC |
| P. infestans | PITG\_05401 | 1000 | Motif-0 | -47 | - | GCAAAAGGTGAAATGA |
|  |  |  | Motif-1 | -239 | + | TACATGTTCCGATAT |
| P. infestans | PITG\_05402 | 1000 | Motif-1 | -591 | + | TACATGTAG |
| P. infestans | PITG\_05403 | 1000 | Motif-0 | -69 | + | CTATTTCTAAATTTG |
| P. infestans | PITG\_05404 | 267 | Motif-17 | -177 | - | AATGTACCCATTCAG |
|  |  |  | Motif-18 | -162 | - | TCTTGTGGTGCACGCA |
| P. infestans | PITG\_05405 | 267 | Motif-17 | -105 | + | AATGTACCCATTCAG |
|  |  |  | Motif-18 | -121 | + | TCTTGTGGTGCACGCA |
| P. infestans | PITG\_05406 | 1000 | Motif-0 | -106 | - | GCAACTTGTGAAATGA |
| P. infestans | PITG\_05409 | 192 | Motif-4 | -119 | + | TCTGATTGGAAGAAA |
| P. infestans | PITG\_05410 | 192 | Motif-4 | -88 | - | TCTGATTGGAAGAAA |
| P. infestans | PITG\_05411 | 214 | Motif-1 | -105 | + | TACATGTAG |
|  |  |  | Motif-17 | -103 | + | CATGTAGCCATTTTG |
| P. infestans | PITG\_05412 | 1000 | Motif-0 | -22 | + | CCACTTCTATATTCAC |
|  |  |  | Motif-1 | -556 | - | ACTACATGCA |
|  |  |  | Motif-2 | -978 | - | AACTTCGACTT |
|  |  |  | Motif-3 | -175 | + | GACTTTGTATTTGAAT |
|  |  |  | Motif-7 | -661 | + | TACTATTAATA |
| P. infestans | PITG\_05413 | 1000 | Motif-0 | -191 | + | TCATTTTCCAGCTTCG |
|  |  |  | Motif-1 | -675 | + | TACATGTAG |
|  |  |  | Motif-2 | -247 | - | ATGGTTGAAGTG |
|  |  |  | Motif-7 | -592 | - | AAGTATTAATA |
| P. infestans | PITG\_05414 | 531 | Motif-7 | -463 | + | AATTATTAATA |
|  |  |  | Motif-8 | -467 | - | TATTAATAATTTAAT |
| P. infestans | PITG\_05415 | 1000 | Motif-0 | -974 | - | TCATTTCCAAATTTG |
|  |  |  | Motif-6 | -167 | + | CCCCCTCCCCCCGAAA |
| P. infestans | PITG\_05419 | 1000 | Motif-8 | -629 | - | TATTATTAATCATAT |
| P. infestans | PITG\_05420 | 1000 | Motif-1 | -818 | - | GTAATTTACCTGTAC |
| P. infestans | PITG\_05421 | 1000 | Motif-0 | -925 | - | TCATTTACCAACGTAC |
|  |  |  | Motif-1 | -735 | + | TACATGTAC |
|  |  |  | Motif-4 | -379 | - | TCCCATTGGCTAAAT |
|  |  |  | Motif-9 | -947 | - | CTGTACCGGTA |
| P. infestans | PITG\_05422 | 1000 | Motif-1 | -888 | + | TACATGTAA |
|  |  |  | Motif-3 | -987 | + | CACTTGGCGTTCGTTT |
|  |  |  | Motif-4 | -22 | + | TTTGATCGGCCAAGT |
| P. infestans | PITG\_05423 | 1000 | Motif-8 | -192 | - | TTTATTTTAATTTTT |
| P. infestans | PITG\_05425 | 1000 | Motif-0 | -645 | + | TCATTCGTCAACTTTT |
|  |  |  | Motif-1 | -801 | + | ATTATACGTGTATT |
|  |  |  | Motif-4 | -337 | - | TTTGATTGGACAAAA |
|  |  |  | Motif-6 | -975 | - | TCCCCTCCCTCCCTCC |
| P. infestans | PITG\_05426 | 1000 | Motif-0 | -71 | + | CACTTGCACTTTTGCT |
|  |  |  | Motif-1 | -705 | + | TACATGTAA |
|  |  |  | Motif-4 | -359 | + | TCCCATTGGTCGATT |
| P. infestans | PITG\_05428 | 539 | Motif-2 | -104 | + | TCACTTCACCCTC |
|  |  |  | Motif-8 | -451 | + | TATTTATAATTTAGT |
| P. infestans | PITG\_05429 | 539 | Motif-2 | -448 | - | TCACTTCACCCTC |
|  |  |  | Motif-8 | -103 | - | TATTTATAATTTAGT |
| P. infestans | PITG\_05430 | 1000 | Motif-0 | -60 | - | GCAAATTGATCAATGA |
|  |  |  | Motif-1 | -128 | + | TACGTGTAC |
|  |  |  | Motif-8 | -90 | - | TTTGTTTTAATTTTA |
|  |  |  | Motif-9 | -125 | + | GTGTACCGGTA |
| P. infestans | PITG\_05434 | 1000 | Motif-0 | -91 | + | CCATTCCCCGTTCCGC |
|  |  |  | Motif-2 | -496 | - | AAAGTTGAAGTC |
|  |  |  | Motif-3 | -189 | + | TTTTGCATTTTGTAT |
|  |  |  | Motif-4 | -259 | + | TGTGATTGGTCAAAA |
| P. infestans | PITG\_05437 | 1000 | Motif-0 | -59 | + | TCATTACTCATTTCGG |
|  |  |  | Motif-3 | -609 | + | TACAGAAGTCAAGTCA |
| P. infestans | PITG\_05440 | 1000 | Motif-0 | -59 | + | TCATTACTCATTTCGG |
|  |  |  | Motif-8 | -568 | - | TATTTTTTTTTTGAA |
| P. infestans | PITG\_05441 | 1000 | Motif-1 | -111 | + | TACATGTAC |
|  |  |  | Motif-16 | -413 | - | CCACAGTAGCAGTAAC |
|  |  |  | Motif-4 | -280 | - | CAGGATTGGTCAAAA |
| P. infestans | PITG\_05446 | 1000 | Motif-0 | -57 | + | CATTTCTCCACTTGCG |
| P. infestans | PITG\_05447 | 155 | Motif-0 | -112 | - | TCATTCTCAAAACCTC |
|  |  |  | Motif-6 | -74 | - | ACCTCCCCCCACCAAT |
| P. infestans | PITG\_05450 | 502 | Motif-1 | -350 | + | TACATGTAC |
| P. infestans | PITG\_05451 | 502 | Motif-1 | -160 | + | TACATGTAT |
| P. infestans | PITG\_05452 | 787 | Motif-7 | -127 | + | CATTATTAATA |
| P. infestans | PITG\_05453 | 744 | Motif-0 | -729 | - | ACAAATATAAAAATGA |
|  |  |  | Motif-1 | -694 | - | ACAACATGTA |
|  |  |  | Motif-4 | -458 | - | TTTGATTGGCCCAAA |
| P. infestans | PITG\_05455 | 153 | Motif-0 | -36 | + | CATTCTCCAAGTTGCC |
|  |  |  | Motif-2 | -99 | + | CGAGTTGAAGTT |
| P. infestans | PITG\_05461 | 1000 | Motif-3 | -444 | + | TAGAAATAGCCAATCA |
| P. infestans | PITG\_05462 | 315 | Motif-18 | -101 | - | TAATGTGGCGCACACG |
| P. infestans | PITG\_05465 | 1000 | Motif-0 | -363 | + | TCACTTTTTGATTCAT |
|  |  |  | Motif-9 | -619 | + | GTGTACCGGTA |
| P. infestans | PITG\_05466 | 1000 | Motif-2 | -981 | + | CATGTTGAATCG |
| P. infestans | PITG\_05470 | 1000 | Motif-8 | -989 | - | TTTGTTTAAATAATA |
| P. infestans | PITG\_05475 | 202 | Motif-9 | -87 | - | GTGTACCGGTA |
| P. infestans | PITG\_05476 | 202 | Motif-9 | -123 | - | GTGTACCGGTA |
| P. infestans | PITG\_05477 | 254 | Motif-1 | -229 | - | GTAGCTTACATGTTT |
| P. infestans | PITG\_05481 | 421 | Motif-0 | -284 | + | TCGTTCTCCATTTAAC |
| P. infestans | PITG\_05482 | 118 | Motif-2 | -45 | + | GAAGTTAAAGTTGA |
| P. infestans | PITG\_05483 | 1000 | Motif-0 | -953 | + | TTAAATTCAAAAGTGA |
| P. infestans | PITG\_05484 | 1000 | Motif-1 | -456 | + | CCTACCGGTACAAGCA |
| P. infestans | PITG\_05485 | 345 | Motif-0 | -65 | - | GAGAATTCACAAGTGA |
|  |  |  | Motif-2 | -123 | + | ACTTCAACTTTTGTAA |
|  |  |  | Motif-3 | -147 | + | AACACTATCCAAGTCA |
| P. infestans | PITG\_05486 | 1000 | Motif-0 | -55 | - | ACAAATTTAAAAATGA |
| P. infestans | PITG\_05487 | 358 | Motif-6 | -23 | - | CCCACTCCCTCCCAAA |
| P. infestans | PITG\_05494 | 1000 | Motif-0 | -15 | + | TCATTTACCATCTTG |
|  |  |  | Motif-7 | -138 | - | CACTATTAATA |
| P. infestans | PITG\_05495 | 158 | Motif-2 | -50 | - | AAAGTTGAAGTG |
| P. infestans | PITG\_05496 | 158 | Motif-2 | -119 | - | CACTTCAACTT |
| P. infestans | PITG\_05497 | 338 | Motif-0 | -308 | - | CCATTTTCTCTTTCAC |
|  |  |  | Motif-4 | -79 | + | CCCGATTGGTTTAAA |
| P. infestans | PITG\_05498 | 338 | Motif-0 | -45 | + | CATTTTCTCTTTCACC |
|  |  |  | Motif-4 | -274 | - | CCCGATTGGTTTAAA |
| P. infestans | PITG\_05502 | 357 | Motif-0 | -213 | + | GCAAAATGCAAACTGC |
|  |  |  | Motif-2 | -92 | + | CAAGTTGAACTG |
| P. infestans | PITG\_05503 | 357 | Motif-0 | -159 | + | CAGTTTGCATTTTGCT |
|  |  |  | Motif-2 | -277 | - | CAAGTTGAACTG |
| P. infestans | PITG\_05504 | 1000 | Motif-0 | -52 | + | CCACTTGCCGTTTTCC |
|  |  |  | Motif-1 | -875 | + | TACATGCATT |
|  |  |  | Motif-2 | -110 | + | CAAGTTCAAGTT |
| P. infestans | PITG\_05505 | 422 | Motif-0 | -78 | + | TCACAAGCCAACTTGC |
| P. infestans | PITG\_05510 | 1000 | Motif-1 | -69 | + | TACATGTAC |
|  |  |  | Motif-4 | -185 | + | TTTGATTGGTCAGCA |
| P. infestans | PITG\_05511 | 1000 | Motif-18 | -627 | - | TTGTGTGGTTTATAAA |
|  |  |  | Motif-2 | -179 | - | TCACATCAACTCGA |
|  |  |  | Motif-3 | -106 | - | AACTTAAATTTTGTAT |
| P. infestans | PITG\_05512 | 305 | Motif-1 | -193 | + | TACATGTAA |
|  |  |  | Motif-2 | -140 | + | ACTTCAACATC |
|  |  |  | Motif-4 | -112 | - | TCTGATTGGATAAAA |
| P. infestans | PITG\_05516 | 1000 | Motif-0 | -107 | + | CACTTTCGAATCTACC |
|  |  |  | Motif-1 | -156 | + | TACATGTAT |
|  |  |  | Motif-7 | -414 | - | TACTATTAATA |
|  |  |  | Motif-8 | -288 | - | TATTTTTTAATTTAA |
| P. infestans | PITG\_05519 | 1000 | Motif-1 | -587 | + | TACATGTAC |
|  |  |  | Motif-2 | -878 | - | AACTTCAACTT |
|  |  |  | Motif-4 | -204 | + | TTTCACTGGCCAAAA |
|  |  |  | Motif-9 | -486 | - | GCGTACCGGTA |
| P. infestans | PITG\_05520 | 1000 | Motif-1 | -441 | + | TACATGTAT |
|  |  |  | Motif-2 | -152 | + | ACTTCAACTTG |
|  |  |  | Motif-4 | -831 | - | TTTCACTGGCCAAAA |
|  |  |  | Motif-9 | -545 | + | GCGTACCGGTA |
| P. infestans | PITG\_05521 | 366 | Motif-2 | -18 | - | GAAGTTCAAGTG |
|  |  |  | Motif-4 | -73 | + | TTTGATTGGCTGAAA |
| P. infestans | PITG\_05522 | 366 | Motif-2 | -360 | + | GAAGTTCAAGTG |
|  |  |  | Motif-4 | -282 | + | CCTGATTGGTTAACA |
| P. infestans | PITG\_05523 | 310 | Motif-2 | -200 | - | CACTTCAACTT |
|  |  |  | Motif-4 | -153 | + | AATGATTGGTCGAAA |
| P. infestans | PITG\_05524 | 822 | Motif-0 | -655 | + | TCATTCTTGCACTGCC |
|  |  |  | Motif-18 | -600 | + | TGGTGTGGCTTACACA |
|  |  |  | Motif-2 | -584 | + | ACTTCAACTTC |
| P. infestans | PITG\_05525 | 822 | Motif-0 | -183 | - | TCATTCTTGCACTGCC |
|  |  |  | Motif-18 | -238 | - | TGGTGTGGCTTACACA |
|  |  |  | Motif-2 | -248 | - | AACTTCAACTT |
| P. infestans | PITG\_05531 | 1000 | Motif-3 | -417 | + | ACTTGATTTTTGTAT |
|  |  |  | Motif-8 | -433 | - | TATTTTTAAGCTGAA |
| P. infestans | PITG\_05532 | 233 | Motif-4 | -120 | - | TGTGATTGGATGAAA |
| P. infestans | PITG\_05533 | 459 | Motif-4 | -300 | - | ACGCATTGGATGAAA |
| P. infestans | PITG\_05534 | 459 | Motif-4 | -174 | + | ACGCATTGGATGAAA |
| P. infestans | PITG\_05536 | 1000 | Motif-2 | -132 | + | GCTTCAACATG |
| P. infestans | PITG\_05537 | 1000 | Motif-1 | -152 | + | TGCATGTAC |
|  |  |  | Motif-2 | -83 | + | GCTTCAACTCG |
|  |  |  | Motif-4 | -118 | - | TCTGATTGGCTGATG |
| P. infestans | PITG\_05538 | 1000 | Motif-1 | -200 | + | TGCATGTAC |
|  |  |  | Motif-2 | -650 | + | ACTTCAACACG |
|  |  |  | Motif-4 | -604 | - | TTTGATAGGCTAATA |
| P. infestans | PITG\_05543 | 1000 | Motif-4 | -104 | + | CCGGATTGGCTCAAA |
|  |  |  | Motif-8 | -206 | - | TATATTTTTTTTAAA |
| P. infestans | PITG\_05545 | 1000 | Motif-0 | -861 | + | CACTTTTCAATTTGAC |
|  |  |  | Motif-1 | -609 | + | TACATGTAG |
|  |  |  | Motif-17 | -739 | - | TGTATAGCCATGTCG |
|  |  |  | Motif-18 | -839 | + | TGGTGTGGTGCACGCA |
| P. infestans | PITG\_05546 | 67 | Motif-4 | -25 | - | GGTGATTGGCTGATT |
| P. infestans | PITG\_05547 | 67 | Motif-4 | -57 | + | GGTGATTGGCTGATT |
| P. infestans | PITG\_05548 | 972 | Motif-1 | -314 | - | ACAATACAAGTACT |
| P. infestans | PITG\_05549 | 1000 | Motif-2 | -15 | + | ACTTCAACATC |
|  |  |  | Motif-4 | -54 | - | GACGATTGGCTGAAA |
| P. infestans | PITG\_05552 | 1000 | Motif-0 | -577 | + | GCAAAATTGGGAATTA |
|  |  |  | Motif-1 | -265 | + | TACATGTAG |
|  |  |  | Motif-4 | -504 | + | TACGATTGGCTCATA |
|  |  |  | Motif-8 | -657 | - | TTTTTTTAAATATTT |
| P. infestans | PITG\_05553 | 1000 | Motif-0 | -30 | + | TCATTCTTCAATCGAT |
|  |  |  | Motif-1 | -298 | - | AATACCGGTACCGTA |
|  |  |  | Motif-7 | -245 | + | TACTATTAATA |
|  |  |  | Motif-9 | -296 | + | CGGTACCGGTA |
| P. infestans | PITG\_05555 | 232 | Motif-2 | -29 | - | GTTGTTGAAGCT |
| P. infestans | PITG\_05556 | 243 | Motif-1 | -38 | + | TACATGCAC |
| P. infestans | PITG\_05557 | 180 | Motif-2 | -140 | + | ACTGCAACTTG |
| P. infestans | PITG\_05560 | 1000 | Motif-4 | -190 | + | TCTGATTGGTCGATA |
| P. infestans | PITG\_05561 | 1000 | Motif-0 | -70 | - | GGAAATTCGAGATTGA |
|  |  |  | Motif-1 | -931 | + | TACATGTAG |
|  |  |  | Motif-2 | -923 | - | ACTGCAACTTC |
|  |  |  | Motif-3 | -317 | + | ACTTGATATTTGTAA |
| P. infestans | PITG\_05562 | 100 | Motif-1 | -94 | + | ACAACATGTAAAATT |
|  |  |  | Motif-4 | -39 | - | TATGATTGGTTTATT |
| P. infestans | PITG\_05563 | 100 | Motif-1 | -16 | - | ACAACATGTA |
|  |  |  | Motif-4 | -53 | - | GTCCATTGGACGAAA |
| P. infestans | PITG\_05565 | 385 | Motif-4 | -269 | + | TTTTATTGGTTATAA |
| P. infestans | PITG\_05567 | 503 | Motif-1 | -144 | - | CTACATTTCATGTAT |
| P. infestans | PITG\_05568 | 503 | Motif-1 | -373 | - | AATACATGAAATGTA |
| P. infestans | PITG\_05572 | 1000 | Motif-0 | -26 | - | GCAAAATGGAGAATCA |
| P. infestans | PITG\_05574 | 1000 | Motif-1 | -649 | - | CGTAGATGTATCA |
|  |  |  | Motif-2 | -350 | - | CAACTTCATCATC |
|  |  |  | Motif-7 | -278 | - | TATTATTAGTA |
| P. infestans | PITG\_05576 | 1000 | Motif-2 | -915 | + | ACTTCAAAATGAGTAA |
| P. infestans | PITG\_05578 | 870 | Motif-0 | -838 | - | ATCACTTTTCGTTTC |
|  |  |  | Motif-1 | -135 | + | TACATGTAT |
|  |  |  | Motif-4 | -451 | + | TTTTATTGGGTGATA |
|  |  |  | Motif-9 | -655 | + | AAGTACCGGTA |
| P. infestans | PITG\_05579 | 870 | Motif-0 | -46 | - | GTGAAACGAAAAGTGA |
|  |  |  | Motif-1 | -209 | + | TACATGTAT |
|  |  |  | Motif-4 | -434 | - | TTTTATTGGGTGATA |
|  |  |  | Motif-9 | -226 | - | AAGTACCGGTA |
| P. infestans | PITG\_05580 | 382 | Motif-0 | -49 | + | CATTTGCGAATTCGCT |
| P. infestans | PITG\_05582 | 350 | Motif-0 | -39 | + | CATTTGCAAATTCGCT |
| P. infestans | PITG\_05583 | 1000 | Motif-1 | -293 | + | TACATGTAT |
|  |  |  | Motif-8 | -416 | - | TTTGTTTAATCAATA |
| P. infestans | PITG\_05584 | 1000 | Motif-3 | -198 | + | ACTTAGGCAATGTAT |
|  |  |  | Motif-4 | -49 | - | CGTGATTGGTTTACA |
| P. infestans | PITG\_05585 | 151 | Motif-4 | -80 | - | TGTGATTGGATGATA |
| P. infestans | PITG\_05586 | 151 | Motif-4 | -55 | + | ATGGATTGGCTAAAA |
| P. infestans | PITG\_05587 | 1000 | Motif-1 | -766 | + | TACATGTAG |
|  |  |  | Motif-2 | -909 | + | GTGGTTGAAGTG |
|  |  |  | Motif-3 | -234 | + | TACGAAATTCAAGTGA |
|  |  |  | Motif-7 | -786 | - | TATTATTAATA |
|  |  |  | Motif-8 | -468 | + | TATTATTTAATAACT |
| P. infestans | PITG\_05589 | 292 | Motif-0 | -120 | + | GCACTATCAAATTTGA |
| P. infestans | PITG\_05590 | 578 | Motif-3 | -182 | + | TACAAAAATAGAGTCA |
| P. infestans | PITG\_05591 | 307 | Motif-0 | -101 | - | GCAACTTGTAGAATGA |
|  |  |  | Motif-18 | -202 | + | TAGTGTGGTAGACACA |
|  |  |  | Motif-2 | -95 | - | GACGGCAACTTGTA |
| P. infestans | PITG\_05592 | 307 | Motif-0 | -222 | + | GCAACTTGTAGAATGA |
|  |  |  | Motif-18 | -121 | - | TAGTGTGGTAGACACA |
|  |  |  | Motif-2 | -226 | + | GACGGCAACTTGTA |
| P. infestans | PITG\_05593 | 252 | Motif-4 | -203 | + | AGTGATTGGTCGAAA |
| P. infestans | PITG\_05594 | 252 | Motif-4 | -64 | - | AGTGATTGGTCGAAA |
| P. infestans | PITG\_05595 | 1000 | Motif-0 | -999 | + | GAAAGTGGAGAAGTGA |
| P. infestans | PITG\_05596 | 1000 | Motif-0 | -123 | - | GCAAATTGCTGAATGA |
|  |  |  | Motif-1 | -646 | + | TACATGTAG |
|  |  |  | Motif-2 | -597 | - | ACTGCAAGTTG |
|  |  |  | Motif-7 | -808 | - | TACTACTAATA |
|  |  |  | Motif-8 | -303 | - | TATTATTAAATATCA |
| P. infestans | PITG\_05597 | 550 | Motif-0 | -511 | - | TCATTTTGCAACTCG |
|  |  |  | Motif-2 | -419 | + | GCGCAACTTGCAGCA |
| P. infestans | PITG\_05598 | 550 | Motif-0 | -53 | + | CATTTTGCAACTCGCC |
|  |  |  | Motif-2 | -146 | - | GCGCAACTTGCAGCA |
| P. infestans | PITG\_05603 | 531 | Motif-0 | -54 | - | CGAAATTTCGAAGTGA |
| P. infestans | PITG\_05604 | 272 | Motif-0 | -40 | + | TCATTCTCCGTTCGTC |
|  |  |  | Motif-17 | -109 | + | AATGTAGCCATATCG |
|  |  |  | Motif-2 | -160 | + | TCTTCAACATCGTCAA |
| P. infestans | PITG\_05606 | 242 | Motif-8 | -240 | - | TTTTATTTATCATAA |
| P. infestans | PITG\_05607 | 920 | Motif-1 | -794 | + | GCTACATATAG |
|  |  |  | Motif-2 | -451 | - | AGCTACGTCGAAGTCG |
|  |  |  | Motif-6 | -332 | + | AGACCCCCCCCCAACG |
| P. infestans | PITG\_05608 | 920 | Motif-1 | -137 | - | GCTACATATAG |
|  |  |  | Motif-2 | -485 | + | AGCTACGTCGAAGTCG |
|  |  |  | Motif-6 | -604 | - | AGACCCCCCCCCAACG |
| P. infestans | PITG\_05609 | 1000 | Motif-0 | -685 | - | GCAAAAACGTAAATGA |
| P. infestans | PITG\_05610 | 1000 | Motif-0 | -541 | + | GCAAAAACGTAAATGA |
|  |  |  | Motif-1 | -79 | + | TACATGTAT |
|  |  |  | Motif-9 | -85 | - | ATGTACCGGTA |
| P. infestans | PITG\_05613 | 1000 | Motif-6 | -886 | - | CCAGCTCCCCCCCCAG |
| P. infestans | PITG\_05614 | 1000 | Motif-1 | -343 | + | TACATGTAT |
|  |  |  | Motif-17 | -314 | + | TGTGTAGCCATTCTT |
|  |  |  | Motif-4 | -76 | - | GCTGATTGGATGATT |
| P. infestans | PITG\_05615 | 1000 | Motif-0 | -43 | + | CATTTCGGATTTTGCC |
|  |  |  | Motif-3 | -108 | - | ACTTGATCTCTCTAT |
| P. infestans | PITG\_05616 | 1000 | Motif-0 | -60 | + | CACTTTGCTATTCACC |
|  |  |  | Motif-1 | -975 | + | TACATGTAC |
|  |  |  | Motif-2 | -386 | + | GGTGTTGAAGTG |
| P. infestans | PITG\_05617 | 623 | Motif-0 | -240 | - | GTAGTTTGCAAAATGA |
|  |  |  | Motif-1 | -256 | + | TACATGTAT |
|  |  |  | Motif-3 | -526 | - | TTGCAAAAGCCAAGCC |
| P. infestans | PITG\_05618 | 1000 | Motif-0 | -208 | + | TCAGTTGGCCATTCTC |
|  |  |  | Motif-1 | -643 | + | TACATGTAT |
|  |  |  | Motif-2 | -949 | - | GACATTGAAGTG |
| P. infestans | PITG\_05619 | 717 | Motif-0 | -684 | - | TCACTTCTCAACTTC |
| P. infestans | PITG\_05620 | 717 | Motif-0 | -48 | - | CGAAGTTGAGAAGTGA |
| P. infestans | PITG\_05621 | 193 | Motif-2 | -181 | + | AACTTCGACGT |
| P. infestans | PITG\_05622 | 193 | Motif-2 | -23 | - | AACTTCGACGT |
| P. infestans | PITG\_05623 | 1000 | Motif-0 | -351 | + | GCAAGTGTAAAACTGA |
|  |  |  | Motif-6 | -144 | + | CCACCGCCCACCACAC |
| P. infestans | PITG\_05628 | 375 | Motif-0 | -39 | + | GCATTTTCATTTTTTC |
| P. infestans | PITG\_05629 | 691 | Motif-0 | -146 | + | GCAGTCGTGAATTTCC |
|  |  |  | Motif-1 | -526 | + | TACATGTAT |
| P. infestans | PITG\_05630 | 405 | Motif-2 | -214 | - | TAGGTTGAAGTG |
| P. infestans | PITG\_05631 | 612 | Motif-4 | -555 | + | TCTGATTGGATGAAA |
| P. infestans | PITG\_05632 | 612 | Motif-4 | -72 | - | TCTGATTGGATGAAA |
| P. infestans | PITG\_05636 | 430 | Motif-0 | -63 | + | CACTTTCGAATCTGCT |
|  |  |  | Motif-2 | -207 | + | CAACTTCATCATC |
| P. infestans | PITG\_05639 | 865 | Motif-18 | -826 | + | TAGTGTGGTGCACACA |
| P. infestans | PITG\_05640 | 865 | Motif-18 | -55 | - | TAGTGTGGTGCACACA |
| P. infestans | PITG\_05649 | 305 | Motif-0 | -27 | + | TCACTTTTGTGTTTTC |
|  |  |  | Motif-2 | -101 | - | CACTTCAACTT |
| P. infestans | PITG\_05652 | 222 | Motif-4 | -26 | + | AACGATTGGCTGAAA |
| P. infestans | PITG\_05653 | 1000 | Motif-0 | -609 | + | TCAGTCTCGTTCTTCC |
|  |  |  | Motif-4 | -452 | - | GGTGATTGGACAATA |
| P. infestans | PITG\_05654 | 1000 | Motif-0 | -638 | - | TCAGTCTCGTTCTTCC |
|  |  |  | Motif-4 | -794 | + | GGTGATTGGACAATA |
| P. infestans | PITG\_05657 | 1000 | Motif-0 | -27 | + | CACTCTCGAATTCGAC |
| P. infestans | PITG\_05658 | 217 | Motif-16 | -41 | + | CCACAGCAGCAGCAAC |
|  |  |  | Motif-18 | -144 | - | TTGTGTGGTACACATA |
|  |  |  | Motif-2 | -196 | - | CAGCTTCAAGTTGA |
| P. infestans | PITG\_05659 | 659 | Motif-0 | -612 | - | ACATTTTCGCTTTTGC |
|  |  |  | Motif-1 | -211 | + | TACATGTAC |
|  |  |  | Motif-2 | -197 | - | CTACTTCAAGTTCA |
|  |  |  | Motif-4 | -50 | + | CACGATTGGCTGAAA |
| P. infestans | PITG\_05660 | 659 | Motif-0 | -62 | + | CATTTTCGCTTTTGCC |
|  |  |  | Motif-1 | -456 | + | TACATGTAC |
|  |  |  | Motif-2 | -476 | + | CTACTTCAAGTTCA |
|  |  |  | Motif-4 | -588 | + | TGTCAGTGGCTGAAA |
| P. infestans | PITG\_05661 | 1000 | Motif-0 | -30 | - | GCAAATGGGAGAATGA |
|  |  |  | Motif-1 | -44 | + | TACATGTAT |
|  |  |  | Motif-2 | -903 | + | CTCGTTGAAGTT |
| P. infestans | PITG\_05664 | 227 | Motif-0 | -50 | + | CATTTGGCAACTTGCC |
|  |  |  | Motif-2 | -107 | + | ACTTCAAGTTG |
| P. infestans | PITG\_05665 | 904 | Motif-1 | -531 | - | TACATGTTGAAGTCA |
|  |  |  | Motif-17 | -869 | - | AATGTACGCATCTGG |
|  |  |  | Motif-2 | -529 | + | ACTTCAACATG |
|  |  |  | Motif-4 | -556 | + | TTTTATTGGACGAAA |
| P. infestans | PITG\_05666 | 904 | Motif-1 | -388 | + | TACATGTTGAAGTCA |
|  |  |  | Motif-17 | -50 | + | AATGTACGCATCTGG |
|  |  |  | Motif-2 | -385 | - | GACTTCAACAT |
|  |  |  | Motif-4 | -336 | + | ATGCATTGGCTAAAA |
| P. infestans | PITG\_05668 | 1000 | Motif-0 | -660 | + | CAATTCTTCAATTTAT |
|  |  |  | Motif-2 | -55 | - | ACTCCAACGCG |
[truncated: 3,455,535 more chars]
