# Supplementary material for: Bioinformatic Inference of Specific and General Transcription Factor Binding Sites in the Plant Pathogen Phytophthora infestans
Source: PLoS One. 2012 Dec 12;7(12):e51295. doi: 10.1371/journal.pone.0051295 (PMC3520976; doi:10.1371/journal.pone.0051295)
Supplement: Data S1 — Overview of the identified motif and their occurrence per gene. (ZIP) [file pone.0051295.s005.zip › Supplementary Data1/overview_per_motif.html]

Overview per Motif


| MotifID | Number of matches (P. infestans/P. sojae/P. ramorum) | Strand + | Strand - | Conservation Score | Motif logo (forward) | Motif Position |
| --- | --- | --- | --- | --- | --- | --- |
| Motif-0 | 18138 (6511/6166/5461) | 10206 | 7932 | 15.1 |  |  |
| Motif-1 | 12070 (5293/3755/3022) | 8765 | 3305 | 9.2 |  |  |
| Motif-10 | 8 (3/4/1) | 4 | 4 | 8.4 |  |  |
| Motif-11 | 13 (5/4/4) | 8 | 5 | 6.9 |  |  |
| Motif-12 | 11 (4/4/3) | 7 | 4 | 7.3 |  |  |
| Motif-13 | 2 (1/0/1) | 2 | 0 | 9.0 |  |  |
| Motif-14 | 9 (3/3/3) | 6 | 3 | 17.5 |  |  |
| Motif-15 | 71 (15/33/23) | 32 | 39 | 16.9 |  |  |
| Motif-16 | 1724 (390/848/486) | 1000 | 724 | 4.2 |  |  |
| Motif-17 | 1938 (613/713/612) | 1011 | 927 | 98.3 |  |  |
| Motif-18 | 1397 (371/556/470) | 630 | 767 | 49.9 |  |  |
| Motif-2 | 12034 (3535/4849/3650) | 6284 | 5750 | 82.2 |  |  |
| Motif-20 | 14 (2/8/4) | 6 | 8 | 14.5 |  |  |
| Motif-21 | 4 (0/0/4) | 3 | 1 | -- |  |  |
| Motif-23 | 7 (2/1/4) | 3 | 4 | 17.5 |  |  |
| Motif-3 | 5249 (1767/2046/1436) | 3323 | 1926 | 110.7 |  |  |
| Motif-4 | 8225 (3418/2645/2162) | 3915 | 4310 | 332.3 |  |  |
| Motif-5 | 27 (8/13/6) | 14 | 13 | 6.6 |  |  |
| Motif-6 | 2377 (624/1112/641) | 1462 | 915 | -- |  |  |
| Motif-7 | 1388 (940/223/225) | 596 | 792 | 6.8 |  |  |
| Motif-8 | 2497 (1777/475/245) | 1181 | 1316 | -- |  |  |
| Motif-9 | 1284 (810/266/208) | 547 | 737 | 3.2 |  |  |
