# Supplementary figures and images for: Bioinformatic Inference of Specific and General Transcription Factor Binding Sites in the Plant Pathogen Phytophthora infestans
Source: PLoS One. 2012 Dec 12;7(12):e51295. doi: 10.1371/journal.pone.0051295 (PMC3520976; doi:10.1371/journal.pone.0051295)

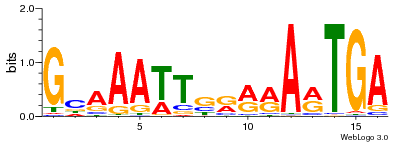

Supplement: Data S1 — Overview of the identified motif and their occurrence per gene. (ZIP) [file pone.0051295.s005.zip › Supplementary Data1/logos/Motif_cluster_0.fa.png]

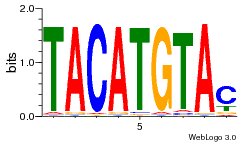

Supplement: Data S1 — Overview of the identified motif and their occurrence per gene. (ZIP) [file pone.0051295.s005.zip › Supplementary Data1/logos/Motif_cluster_1.fa.png]

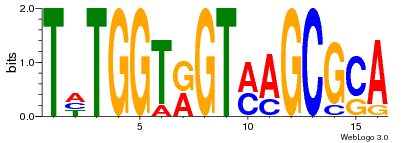

Supplement: Data S1 — Overview of the identified motif and their occurrence per gene. (ZIP) [file pone.0051295.s005.zip › Supplementary Data1/logos/Motif_cluster_10.fa.png]

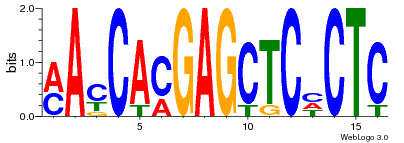

Supplement: Data S1 — Overview of the identified motif and their occurrence per gene. (ZIP) [file pone.0051295.s005.zip › Supplementary Data1/logos/Motif_cluster_11.fa.png]

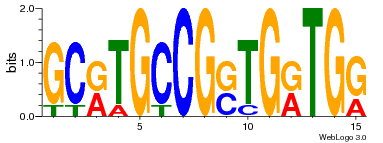

Supplement: Data S1 — Overview of the identified motif and their occurrence per gene. (ZIP) [file pone.0051295.s005.zip › Supplementary Data1/logos/Motif_cluster_12.fa.png]

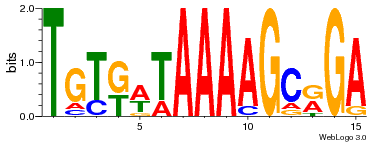

Supplement: Data S1 — Overview of the identified motif and their occurrence per gene. (ZIP) [file pone.0051295.s005.zip › Supplementary Data1/logos/Motif_cluster_13.fa.png]

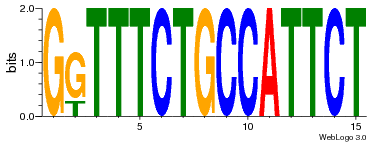

Supplement: Data S1 — Overview of the identified motif and their occurrence per gene. (ZIP) [file pone.0051295.s005.zip › Supplementary Data1/logos/Motif_cluster_14.fa.png]

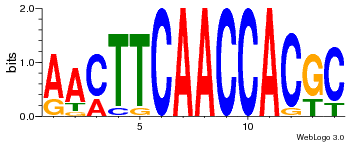

Supplement: Data S1 — Overview of the identified motif and their occurrence per gene. (ZIP) [file pone.0051295.s005.zip › Supplementary Data1/logos/Motif_cluster_15.fa.png]

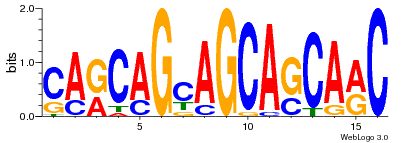

Supplement: Data S1 — Overview of the identified motif and their occurrence per gene. (ZIP) [file pone.0051295.s005.zip › Supplementary Data1/logos/Motif_cluster_16.fa.png]

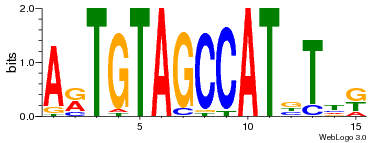

Supplement: Data S1 — Overview of the identified motif and their occurrence per gene. (ZIP) [file pone.0051295.s005.zip › Supplementary Data1/logos/Motif_cluster_17.fa.png]

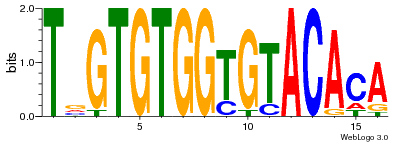

Supplement: Data S1 — Overview of the identified motif and their occurrence per gene. (ZIP) [file pone.0051295.s005.zip › Supplementary Data1/logos/Motif_cluster_18.fa.png]

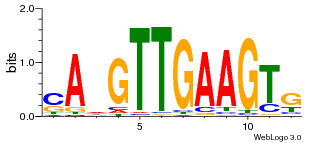

Supplement: Data S1 — Overview of the identified motif and their occurrence per gene. (ZIP) [file pone.0051295.s005.zip › Supplementary Data1/logos/Motif_cluster_2.fa.png]

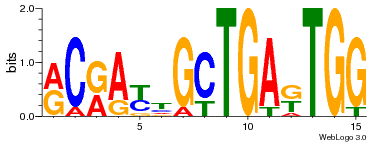

Supplement: Data S1 — Overview of the identified motif and their occurrence per gene. (ZIP) [file pone.0051295.s005.zip › Supplementary Data1/logos/Motif_cluster_20.fa.png]

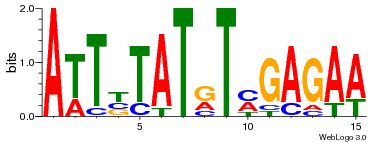

Supplement: Data S1 — Overview of the identified motif and their occurrence per gene. (ZIP) [file pone.0051295.s005.zip › Supplementary Data1/logos/Motif_cluster_21.fa.png]

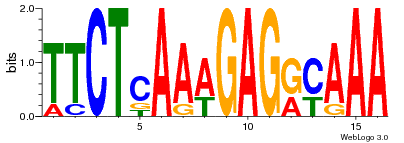

Supplement: Data S1 — Overview of the identified motif and their occurrence per gene. (ZIP) [file pone.0051295.s005.zip › Supplementary Data1/logos/Motif_cluster_23.fa.png]

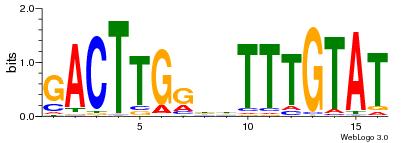

Supplement: Data S1 — Overview of the identified motif and their occurrence per gene. (ZIP) [file pone.0051295.s005.zip › Supplementary Data1/logos/Motif_cluster_3.fa.png]

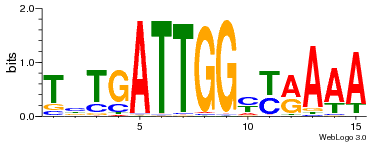

Supplement: Data S1 — Overview of the identified motif and their occurrence per gene. (ZIP) [file pone.0051295.s005.zip › Supplementary Data1/logos/Motif_cluster_4.fa.png]

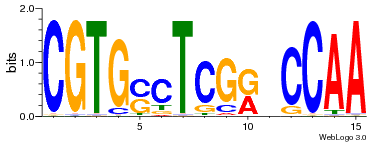

Supplement: Data S1 — Overview of the identified motif and their occurrence per gene. (ZIP) [file pone.0051295.s005.zip › Supplementary Data1/logos/Motif_cluster_5.fa.png]

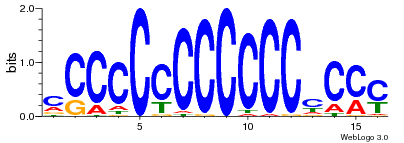

Supplement: Data S1 — Overview of the identified motif and their occurrence per gene. (ZIP) [file pone.0051295.s005.zip › Supplementary Data1/logos/Motif_cluster_6.fa.png]

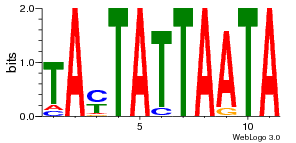

Supplement: Data S1 — Overview of the identified motif and their occurrence per gene. (ZIP) [file pone.0051295.s005.zip › Supplementary Data1/logos/Motif_cluster_7.fa.png]

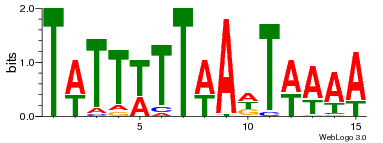

Supplement: Data S1 — Overview of the identified motif and their occurrence per gene. (ZIP) [file pone.0051295.s005.zip › Supplementary Data1/logos/Motif_cluster_8.fa.png]

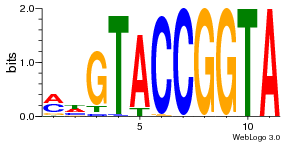

Supplement: Data S1 — Overview of the identified motif and their occurrence per gene. (ZIP) [file pone.0051295.s005.zip › Supplementary Data1/logos/Motif_cluster_9.fa.png]

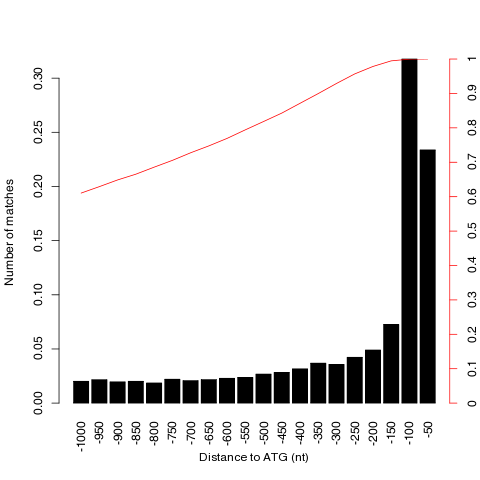

Supplement: Data S1 — Overview of the identified motif and their occurrence per gene. (ZIP) [file pone.0051295.s005.zip › Supplementary Data1/positions/Motif_cluster_0.fimo_position.png]

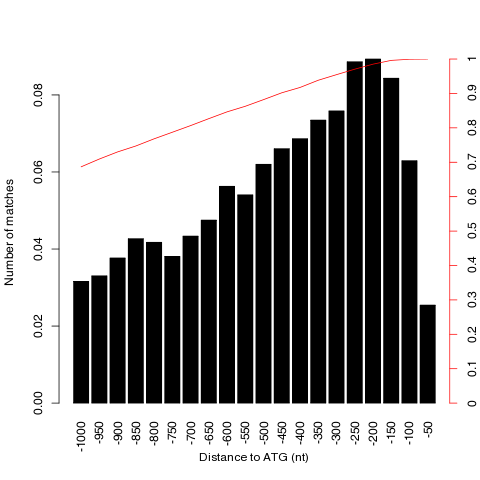

Supplement: Data S1 — Overview of the identified motif and their occurrence per gene. (ZIP) [file pone.0051295.s005.zip › Supplementary Data1/positions/Motif_cluster_1.fimo_position.png]

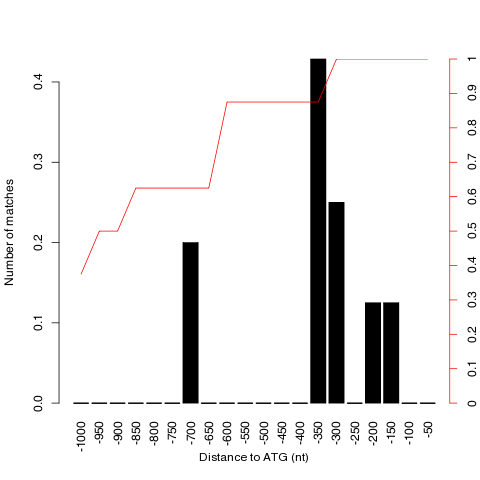

Supplement: Data S1 — Overview of the identified motif and their occurrence per gene. (ZIP) [file pone.0051295.s005.zip › Supplementary Data1/positions/Motif_cluster_10.fimo_position.png]

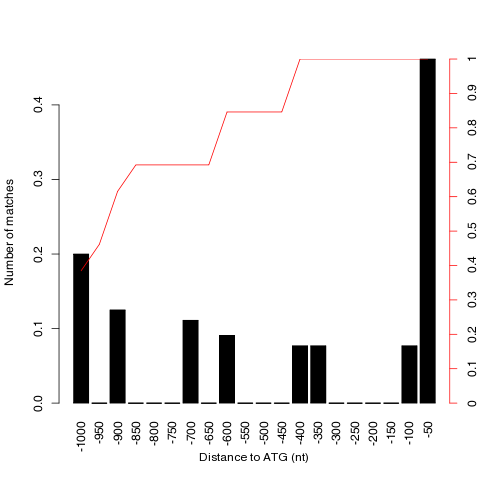

Supplement: Data S1 — Overview of the identified motif and their occurrence per gene. (ZIP) [file pone.0051295.s005.zip › Supplementary Data1/positions/Motif_cluster_11.fimo_position.png]

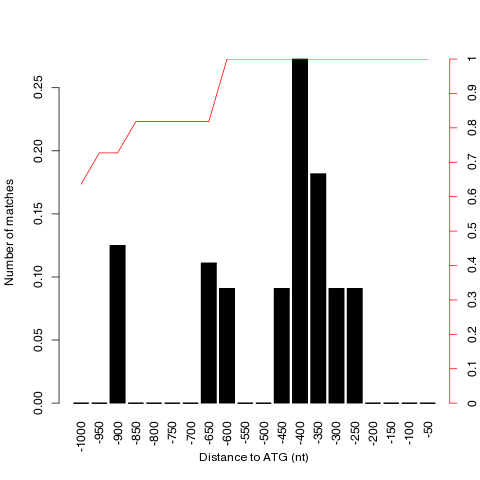

Supplement: Data S1 — Overview of the identified motif and their occurrence per gene. (ZIP) [file pone.0051295.s005.zip › Supplementary Data1/positions/Motif_cluster_12.fimo_position.png]

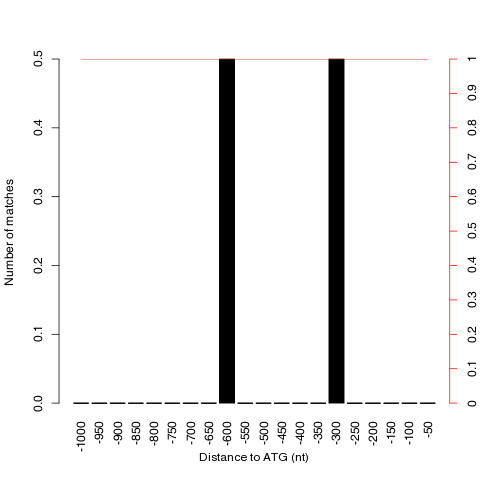

Supplement: Data S1 — Overview of the identified motif and their occurrence per gene. (ZIP) [file pone.0051295.s005.zip › Supplementary Data1/positions/Motif_cluster_13.fimo_position.png]

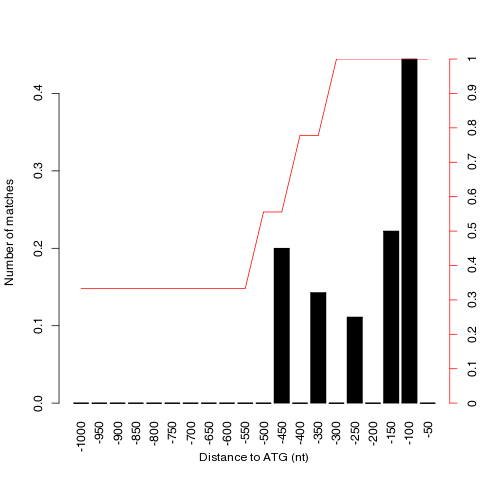

Supplement: Data S1 — Overview of the identified motif and their occurrence per gene. (ZIP) [file pone.0051295.s005.zip › Supplementary Data1/positions/Motif_cluster_14.fimo_position.png]

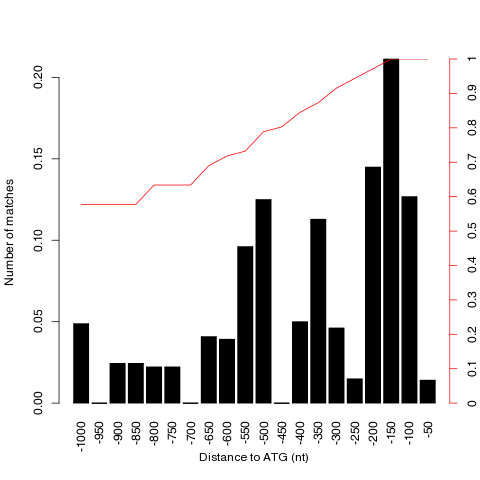

Supplement: Data S1 — Overview of the identified motif and their occurrence per gene. (ZIP) [file pone.0051295.s005.zip › Supplementary Data1/positions/Motif_cluster_15.fimo_position.png]

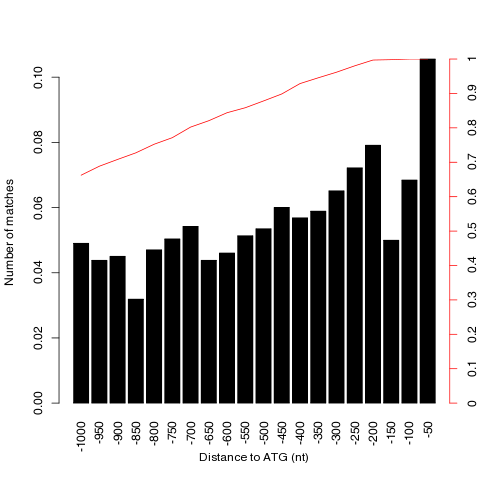

Supplement: Data S1 — Overview of the identified motif and their occurrence per gene. (ZIP) [file pone.0051295.s005.zip › Supplementary Data1/positions/Motif_cluster_16.fimo_position.png]

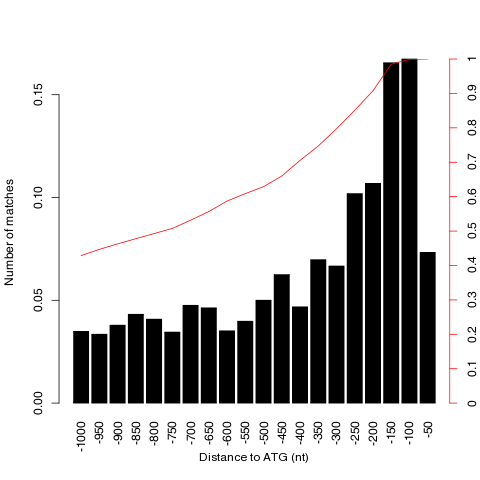

Supplement: Data S1 — Overview of the identified motif and their occurrence per gene. (ZIP) [file pone.0051295.s005.zip › Supplementary Data1/positions/Motif_cluster_17.fimo_position.png]

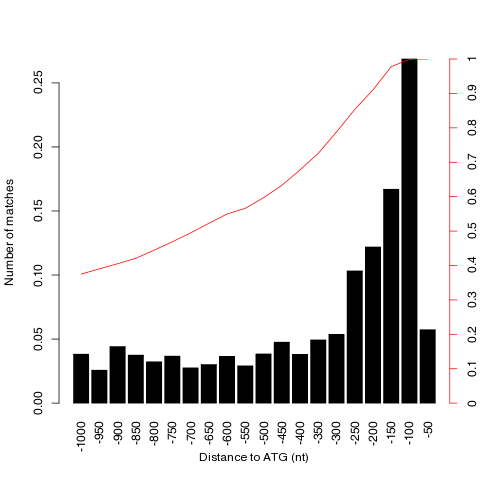

Supplement: Data S1 — Overview of the identified motif and their occurrence per gene. (ZIP) [file pone.0051295.s005.zip › Supplementary Data1/positions/Motif_cluster_18.fimo_position.png]

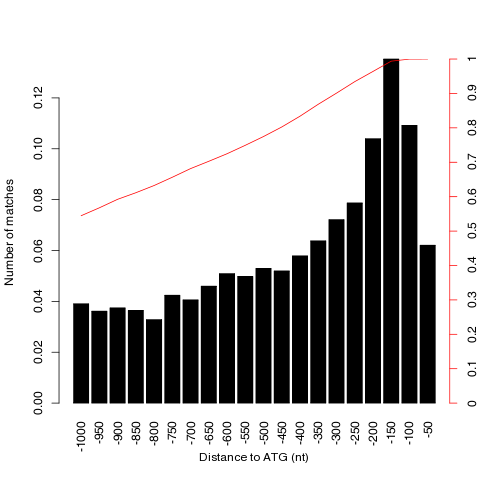

Supplement: Data S1 — Overview of the identified motif and their occurrence per gene. (ZIP) [file pone.0051295.s005.zip › Supplementary Data1/positions/Motif_cluster_2.fimo_position.png]

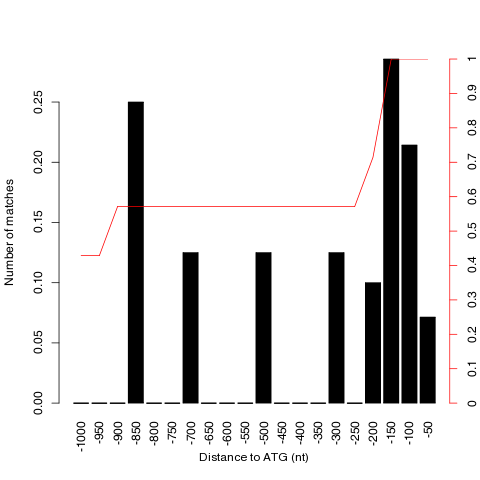

Supplement: Data S1 — Overview of the identified motif and their occurrence per gene. (ZIP) [file pone.0051295.s005.zip › Supplementary Data1/positions/Motif_cluster_20.fimo_position.png]

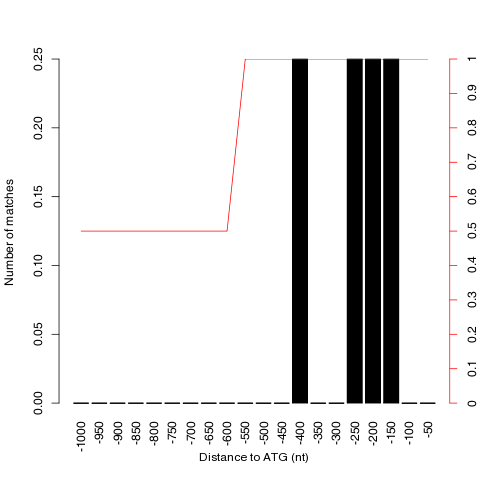

Supplement: Data S1 — Overview of the identified motif and their occurrence per gene. (ZIP) [file pone.0051295.s005.zip › Supplementary Data1/positions/Motif_cluster_21.fimo_position.png]

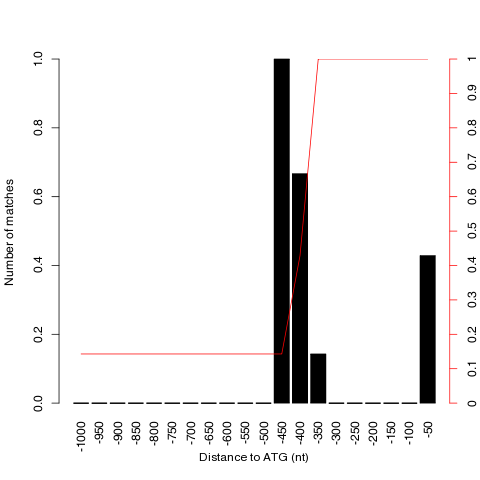

Supplement: Data S1 — Overview of the identified motif and their occurrence per gene. (ZIP) [file pone.0051295.s005.zip › Supplementary Data1/positions/Motif_cluster_23.fimo_position.png]

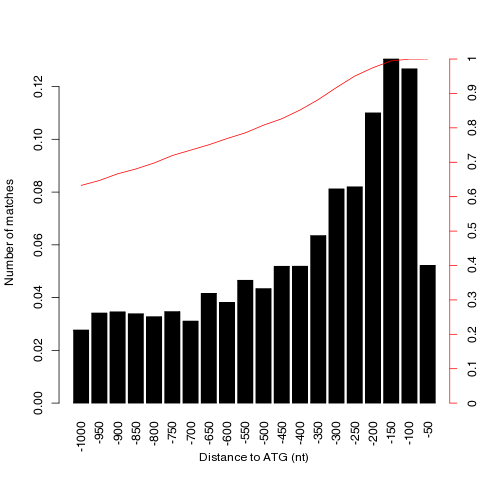

Supplement: Data S1 — Overview of the identified motif and their occurrence per gene. (ZIP) [file pone.0051295.s005.zip › Supplementary Data1/positions/Motif_cluster_3.fimo_position.png]

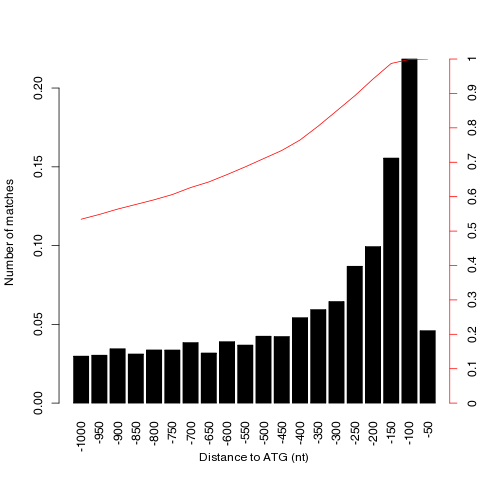

Supplement: Data S1 — Overview of the identified motif and their occurrence per gene. (ZIP) [file pone.0051295.s005.zip › Supplementary Data1/positions/Motif_cluster_4.fimo_position.png]

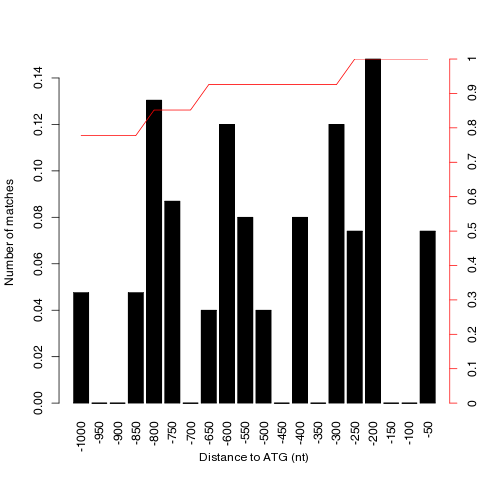

Supplement: Data S1 — Overview of the identified motif and their occurrence per gene. (ZIP) [file pone.0051295.s005.zip › Supplementary Data1/positions/Motif_cluster_5.fimo_position.png]

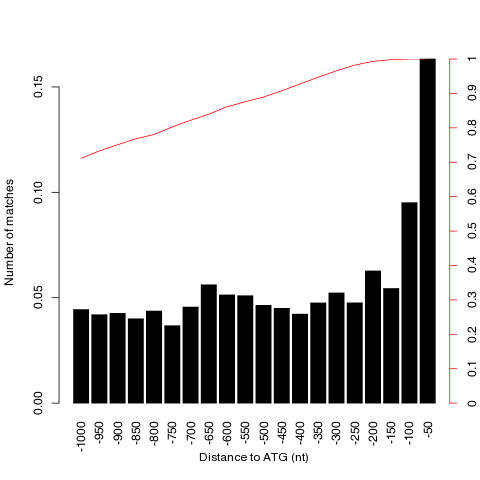

Supplement: Data S1 — Overview of the identified motif and their occurrence per gene. (ZIP) [file pone.0051295.s005.zip › Supplementary Data1/positions/Motif_cluster_6.fimo_position.png]

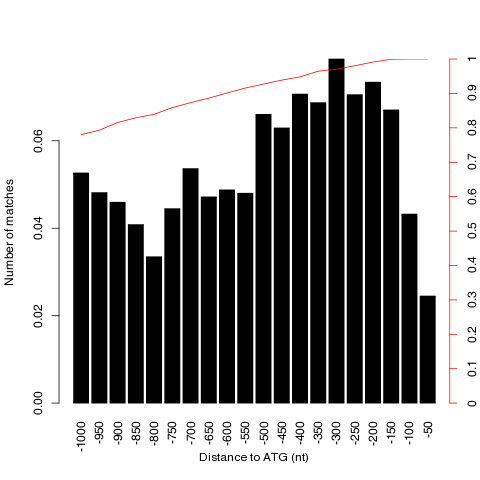

Supplement: Data S1 — Overview of the identified motif and their occurrence per gene. (ZIP) [file pone.0051295.s005.zip › Supplementary Data1/positions/Motif_cluster_7.fimo_position.png]

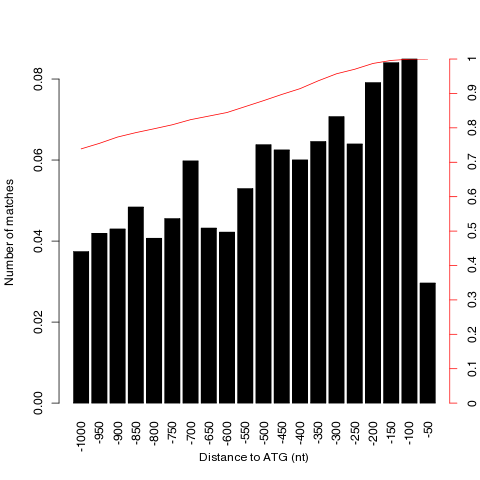

Supplement: Data S1 — Overview of the identified motif and their occurrence per gene. (ZIP) [file pone.0051295.s005.zip › Supplementary Data1/positions/Motif_cluster_8.fimo_position.png]

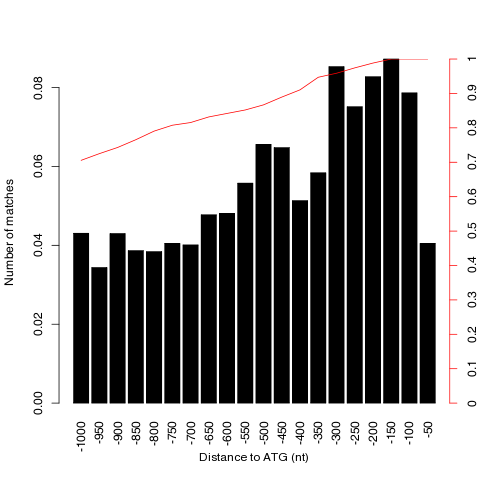

Supplement: Data S1 — Overview of the identified motif and their occurrence per gene. (ZIP) [file pone.0051295.s005.zip › Supplementary Data1/positions/Motif_cluster_9.fimo_position.png]
